# Electrolyte-Guided Selectivity Unlocks Pathway Control in Electrochemical Olefin Functionalization

**DOI:** 10.1021/jacs.5c20366

**Published:** 2026-04-01

**Authors:** Daniel Gordon-Levitan, Dmitrii Bushmin, Jonathan R. Church, Rakesh Mondal, Tsafrir Bohak, Natalia Gloriozova, Moran Feller, Mark A. Iron, Haim Weissman, Michal Leskes, Boris Rybtchinski, Samer Gnaim

**Affiliations:** † Department of Molecular Chemistry and Materials Science, 201485 Weizmann Institute of Science, Rehovot 7610001, Israel; ‡ Department of Chemical Research Support, 34976 Weizmann Institute of Science, Rehovot 7610001, Israel

## Abstract

Organic electrosynthesis offers a direct, electricity-driven strategy for constructing complex molecular structures in a more sustainable and innovative manner. However, even with the precise redox control that electrochemistry affords, steering highly reactive intermediates along a single productive pathway remains a central challenge, particularly when multiple mechanistic manifolds are accessible. Herein, we demonstrate that the identity of the supporting electrolyte dictates the selectivity of electro-reductive olefin coupling, directing the transformation toward either exclusively linear or exclusively branched products. Radical probes, CV, SEM, ssNMR, EPR, and DFT clarify these distinct pathways. Ammonium salts preserve the terminal spin bias of the styrene radical anion, promoting solution-phase radical addition for linear products. Lithium salts instead form a Li-rich interphase that drives benzylic spin localization and channels surface-confined radical coupling to yield branched products. This platform streamlines access to pharmaceutical-relevant scaffolds and reveals previously underexplored polar hydrofunctionalization of conjugated olefins. These findings establish electrolyte-controlled interfacial organization as a powerful lever to control product selectivity in organic electrosynthesis.

## Graphical abstract

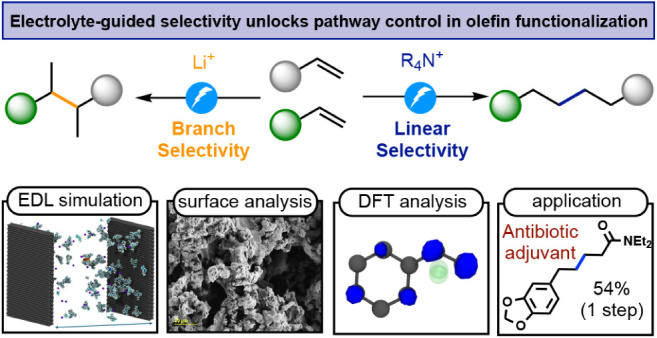

## Introduction

Positioned at the nexus of renewable energy and chemical innovation, electrosynthesis offers a direct pathway to power more innovative and sustainable molecular constructions.^1^ On a fundamental level, the success of any electrosynthetic system hinges on its core components, among which is the supporting electrolyte. While these electrolytes are primarily added to a solution to enhance conductivity and enable efficient charge transport, they also fundamentally shape interfacial behavior through the formation of the electrical double-layer (EDL) (Figure 1A).^2^ This interfacial layer is critical in controlling the kinetics and efficiency of electrochemical processes by influencing the local ion distribution, electric field, and potential drop.

**Figure 1:**
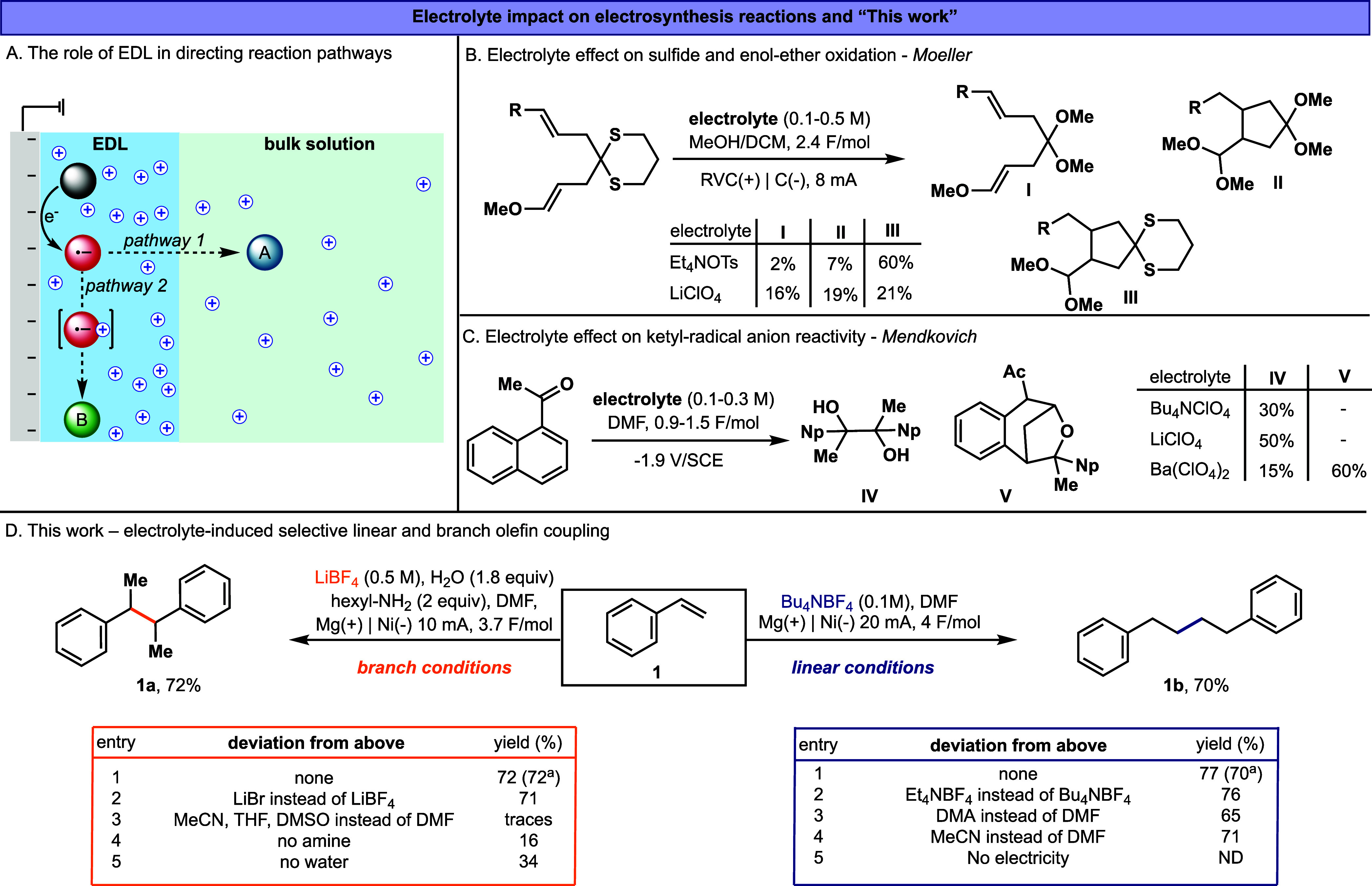
A) Schematic description of the EDL formation and effect on CO_2_ reduction. B) and C) Examples of electrolyte impact on reaction pathways in organic electrosynthesis. D) This work – optimized condition for electrolyte-induced selective linear and branch olefin coupling. ^a^isolated yields.

A clear impact of the electrolyte nature on the reaction outcomes has been widely studied in the context of CO_2_ electrochemical reduction for energy conversion.^3^ Far from being passive, the electrolyte governs the interfacial environment by controlling local pH, electric fields, and cation-specific interactions. As demonstrated by Koper, Strasser, Masel, and others, these factors alter the adsorption energies and stability of key intermediates, which ultimately dictate the selective formation of C_1_ (e.g., CO, formate) or C_2_ products (e.g., ethylene, ethanol).^4^

In organic electrosynthesis, numerous studies reveal that the electrolyte can actively participate in the electrochemical reaction pathway, acting as either a reagent or a mediator.^5^ In parallel scenarios, it is frequently reported that certain electrolytes enhance reaction yields while others suppress the desired reactivity, often for reasons that are not well understood.^6^ However, reports that highlight modulation of reaction pathways through deliberate tuning of the electrolyte’s identity remain uncommon (Figure 1B and C).^7^ In an oxidative diene cyclization manifold, Moeller and coworkers demonstrated that switching between a bulky tetraalkylammonium electrolyte (e.g., TEA^+^) and a small, strongly coordinating alkali-metal cation (Li^+^) can measurably shift product distributions (Figure 1B).^7a^ This behavior is consistent with electrolyte-dependent organization of the interfacial microenvironment (ion pairing/double-layer structure), which can alter the effective polarity and reactivity of the anodically generated radical-cation intermediate and thereby bias the competition between distinct bond-forming/trapping pathways. Mendkovich and coworkers likewise showed that cathodic ketone reductive dimerization is electrolyte-sensitive (Figure 1C). The unique effect of Ba^+^ electrolyte on the charge distribution of ketyl radical-anion intermediates favored a different regiochemical outcome than other coordinating electrolytes.^7^

During our research on the electro-reductive functionalization of olefins, we discovered a notable phenomenon: Under electro-reductive conditions, an activated olefin, for instance, **1**, undergoes selective hydrodimerization depending on the nature of the electrolyte (Figure 1D and see SI, Section 1.5). While alkylammonium salts as electrolytes induce the selective formation of the linear hydrodimerization product **1b**, lithium salts, unexpectedly, induce selective hydrodimerization to give exclusively the branched product **1a.** To our knowledge, formation of **1a** via direct cathodic reduction of styrene has not been reported, and none of the other electrolytes in our study produced it (e.g., Na^+^, K^+^, Cs^+^, Ba^+^, R_4_P^+^, R_3_S^+^, see SI, Section 1.5). Generally, electrochemical reductive coupling of olefins selectively gives the linear products, classically exemplified by the industrial electrosynthesis of adiponitrile from acrylonitrile.^8a^ This chemistry has been extended to alternating-current setups and other olefin classes.^8^ Similarly, activated olefin-based radical anions have been harnessed to react with electrophiles as reported in studies by Shono, Polyzos, and others.^9^

## Results and Discussion

We first examined the electrochemical conditions that lead to the formation of the branched and linear hydrodimerization products, **1a** and **1b** (see SI, Section 1.5, for a detailed analysis of the reaction conditions). Under electroreductive conditions, the reaction selectivity is governed by the identity of the cation in the supporting electrolyte, whereas other reaction components primarily affect the product yield. For the branched product **1a**, LiBF_4_ was used as the supporting electrolyte, with hexylamine and H_2_O serving as proton sources. In contrast, the formation of the linear product **1b** required Bu_4_NBF_4_, which played a dual role as both a supporting electrolyte and a proton source (Figure 1D). In both cases, DMF was employed as the solvent, a magnesium anode/nickel cathode pair was used, and the reactions were performed under identical concentration and temperature. These results demonstrate that the coupling selectivity is exclusively dictated by the nature of the electrolyte in the reaction mixture.

To understand the unexpected electrolyte-dependent outcomes of our electro-reductive olefin coupling reaction (Figure 1D), we examined the underlying mechanism for the formation of the branched and linear products, **1a** and **1b**, respectively. Three questions guided our analysis: (*i*) Does the countercation modulate the electronic structure and stability of the styrene radical anion? (*ii*) What is the operative pathway for the C–C bond-forming step under each electrolyte condition? (*iii*) Are the observed selectivity trends solely dictated by ion-pairing interactions, or do additional interfacial phenomena play a decisive role?

As the first step in the reaction pathway is the styrene reduction to form the corresponding radical anion intermediate,^10^ we postulate that the selectivity may arise from the different charge-spin density localization across the reduced intermediate. To test this hypothesis, we trapped the generated radical anion intermediates using α- and β-cyclopropylstyrene radical-clock probes (Figure 2A). When β-cyclopropyl styrene **2** was subjected to the linear conditions (Bu_4_N^+^), a complex mixture of multiple ring-opened products, including **2b** and **2c** (Figure 2A-i), was obtained, and notably, no detectable linear coupling product of **2** was observed (full conversion of **2** was obtained).^11^ This result suggests that the radical is predominantly positioned on the β-position of the reduced styrene, while the anion is stabilized at the benzylic position, as shown in intermediate **2a**. In contrast, exposing the same substrate, **2**, to the branched conditions (Li^+^) delivered the branched coupling product **2e** in 57% yield with no detectable ring-opening products (Figure 2A-ii). This would indicate that Li^+^ induces a change in the operative radical-anion, favoring **2d** as the dominant species. This is further supported by the reduction of α-cyclopropylstyrene **3** (Figure 2A-iii), a probe that is specifically activated by a benzylic radical.^12^ Under the branched conditions (Li^+^), **3** undergoes complete ring opening, delivering **3a** and **3b** in 20% and 35% yield, respectively (control experiments for the radical-clock probes are detailed in the SI file, Figures S8 and S9). These spin-probe experiments clearly indicate distinct spin distributions in radical anions generated under different electro-reductive conditions. In the presence of Bu_4_N^+^ cations, the radical anion exhibited spin localization dominantly at the homobenzylic carbon. When Li^+^ cations were employed, the unpaired electron density was exclusively localized at the benzylic position. With some activated olefins, this interplay in radical position is reflected by striking color changes in the reaction mixture, signaling different electronic structures and degrees of conjugation with each salt (see Figure 2C-photo).

**Figure 2:**
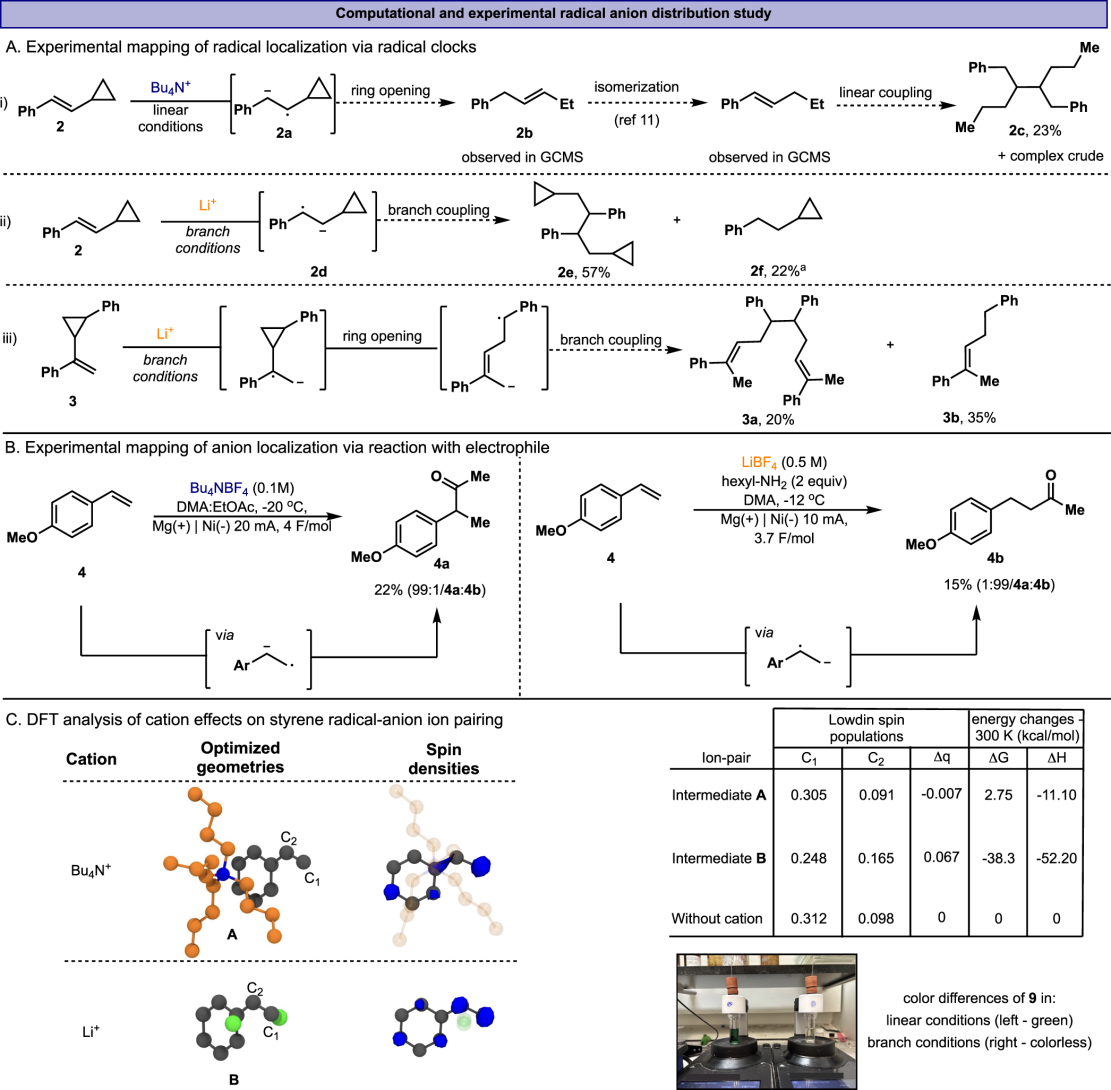
A) Radical clock probes and experiments, and isolated products with their yields. B) Selective electro-reductive acylation of 4-methoxystyrene. C) Computational analysis of spin distribution in styrene radical anion with varied cations. Each structure was optimized at the UKS-ωB97X-D3/cc-pVTZ level of theory and using a Conductor-like Polarizable Continuum Model (CPCM) implicit DMF solvent. Bottom-right - Linear and branched reactions exhibit different colors. ^a^GC yield.

To further validate our hypothesis, we investigated the polar reactivity of the formed olefinic radical anion toward electrophilic acylation with dimethylacetamide (DMA) or ethyl acetate (EtOAc). In this case, it is the anionic center that is expected to attack the electrophile; therefore, one would expect an opposite product distribution (i.e., internal acylation with Li^+^ salts and terminal acylation with Bu_4_N^+^). To suppress competitive anion quenching and dimerization, we slightly modified the reaction conditions. While the same electrode, electrolyte, and electrolysis conditions were retained. For the branched conditions (Li^+^ electrolyte), water was omitted (to prevent rapid protonation), and the solvent was switched from DMF to DMA. In the case of the linear conditions (Bu_4_N^+^ electrolyte), the solvent was modified from DMF to a mixture of DMA/EtOAc. Additionally, to suppress overreduction and improve product yield, the reaction temperature was lowered to −20 °C. Interestingly, these conditions enabled the formation of acylation products with reversed regioselectivity relative to the dimerization. With the Bu_4_N^+^ electrolyte, acylation occurred primarily at the benzylic position, affording **4a**, whereas the Li^+^ electrolyte favored selective acylation at the terminal site, yielding **4b**. This further supports our conclusion that the anion position is predominantly affected by the electrolyte used.

Subsequently, DFT computational analyses were conducted to elucidate the influence of the electrolyte on the electronic structure and spin/charge distribution across the radical-anion skeleton. First, we optimized the lowest-energy ion-paired structures and analyzed the Löwdin spin populations of bare styrene radical anion as well as its complexes with different cations (see SI for the full computational details).^13^ Complexation with a single Bu_4_N^+^ ion, the most stable structure (intermediate **A**, Figure 2C), features cation−π interactions with the arene (Δ*G*_300_ = +2.7 kcal/mol). In contrast, Li^+^ forms a tightly bound, doubly coordinated ion pair (intermediate **B**, Figure 2C) that is strongly favored thermodynamically (Δ*G*_300_ = –38.3 kcal/mol). According to our calculations, switching the cation from tetrabutylammonium to lithium results in an over 80% increase in spin population at C_2_. Moreover, the Li^+^ produced a large perturbation, drawing electron density away from the C_1_ region (a large positive Δ*q*, change in Löwdin atomic charges, relative to the bare styrene radical-anion, see SI, Figures S1 and S2). The sum of Δq indicates that both the negative charge on the radical and the positive charge of Li^+^ are reduced, consistent with a partial charge transfer and polarization effect.

The DFT calculations are consistent with the radical-probe experiments performed under the linear conditions, as they indicate that bulky, weakly coordinating cations such as Bu_4_N^+^ engage in diffuse, outer-sphere cation−π interactions that maintain the intrinsic terminal-biased spin distribution. In contrast, under the Li^+^ conditions, while the DFT results remain consistent with the overall trend, suggesting that the hard Li^+^ cation forms more directional and tightly associated ion pairs, leading to enhanced spin localization at the benzylic (C_2_) position, it does not fully explain the inherent selectivity observed experimentally under the branch conditions. This implies that additional effects beyond simple ion pairing, potentially involving interfacial or medium-dependent processes, contribute to the observed outcome (*vide infra*).

Next, we sought to delineate the pathways responsible for the selective C–C bond formation of the linear versus branched products. Under the linear conditions and based on our assignment of radical-anion localization consistent with intermediate **A** (Figures 2C and 3A) allows us to hypothesize that C–C bond formation proceeds via radical addition of the reduced styrene to a neutral olefin in solution.

**Figure 3:**
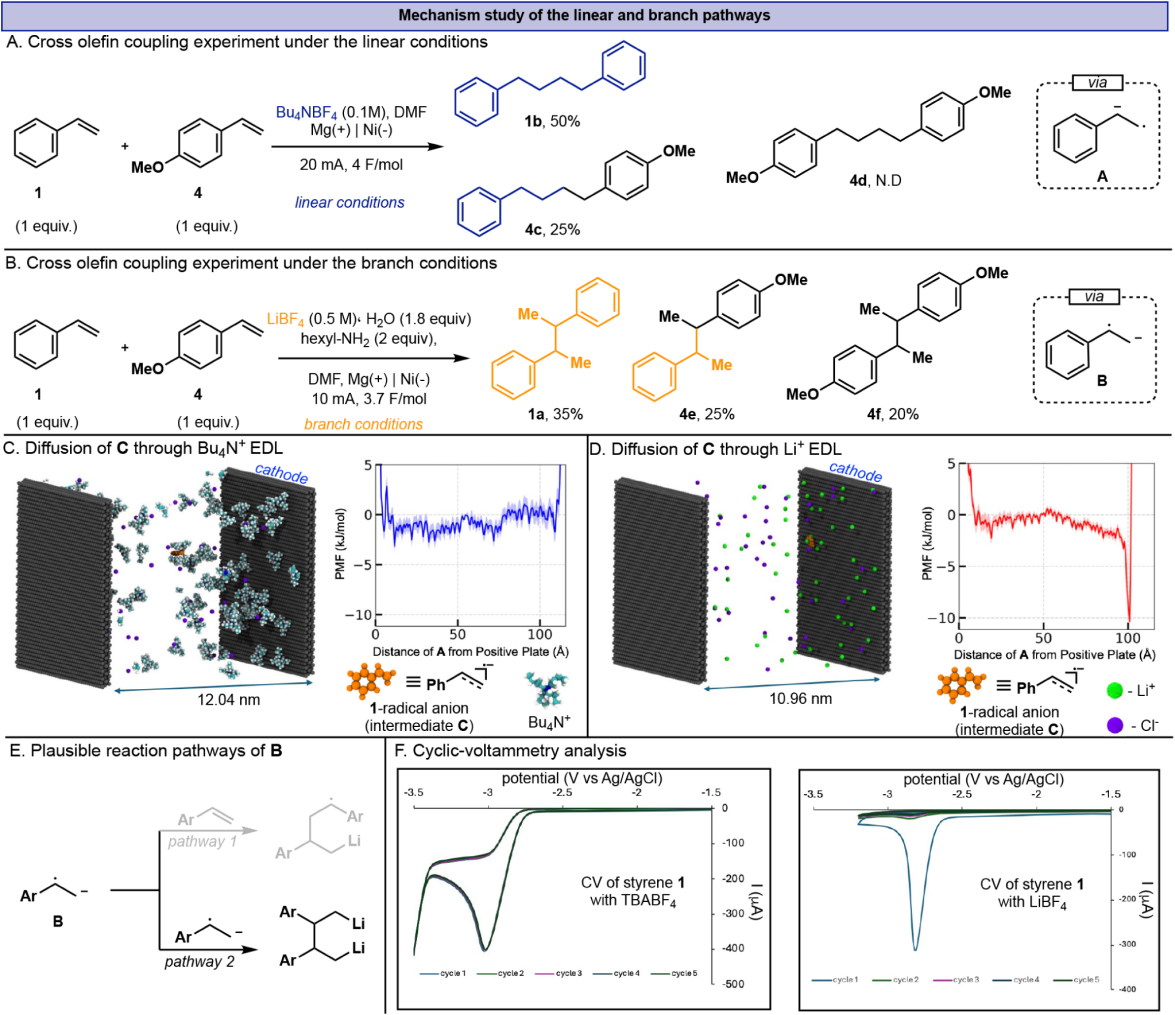
Cross olefin coupling under linear (A) and branched (B) conditions, GC yields. Diffusion simulation of **C** with Bu_4_N^+^ (C) and Li^+^ (D). E) Plausible reaction pathway of the benzylic radical **B**. F) Cyclic voltammetry analysis.

To test this, we performed a cross-olefin coupling using two substrates with distinct electronic natures, **1** and **4** (Figure 3A). Electrolysis at a constant current selectively reduces the olefins, resulting in the product distribution shown in Figure 3A; homocoupling product **1b** is observed at 50%, heterocoupling product **4c** is obtained at 25%, while the homocoupling product **4d** was not detected. This outcome suggests that the initial electron transfer is more favored to the substrate **1** (more electron-deficient), as well as the addition of the nucleophilic radical to electron-rich alkenes such as 4-methoxystyrene is kinetically disfavored.

This conclusion was further supported using constant potential electrolysis (see SI, Additional Data Section, Figures S10 and S11). Consequently, the coupling between radical anions and neutral styrene dominates the reaction pathway. This interpretation is consistent with the established polarity-matching principle in radical chemistry, which predicts that nucleophilic radicals preferentially add to electron-poor π-systems, whereas reactions with electron-rich alkenes proceed slowly or are completely suppressed.^14^ This pathway is presumably enabled by the efficient diffusion of the cathodically generated olefin radical anion to the bulk solution in the electrochemical cell. To probe how the EDL influences the mobility of this reduced species, classical simulations were conducted on the negatively charged radical anion **C** (Figure 3C) in the presence of Bu_4_N^+^ between nickel electrodes.^15^ Umbrella-sampling calculations were performed by constraining the styrene center of mass at 2 Å intervals along the electrochemical cell, with 50 ns trajectories collected for each window to determine the potential of mean force (PMF). For the Bu_4_N^+^ electrolyte, the resulting EDL exhibited a diffuse interfacial layer at the cathode and a nearly flat energy profile without a discernible well (Figure 3C), indicating minimal stabilization of **C** near the electrode surface. For comparison, the simulation with Li^+^ showed a compact EDL and revealed a distinct interfacial minimum of approximately 10 kcal/mol adjacent to the negatively charged electrode, reflecting strong stabilization of **C** by Li^+^ ions (Figure 3D). These findings support the mechanistic picture that bulky ammonium electrolytes promote a predominantly solution-phase radical addition pathway, favoring linear selectivity. This behavior parallels the proposed mechanisms operating in adiponitrile electrosynthesis and related radical-anion-driven systems (see SI, Mechanism Discussion Section, for detailed mechanism).^8^

Under branched conditions, the cross-olefin experiment (Figure 3B) yields all three coupling products **1a**, **4e**, and **4f** in comparable yields that markedly differ from those in the linear regime, indicating a mechanistic divergence. The observation of the near-statistical ratio alongside product **3a** (obtained in the radical probe experiment, Figure 2A-iii) suggests a radical–radical coupling pathway (Figure 3E, pathway 2), wherein the generated radical anions undergo a recombination pathway. Deviations from an ideal 1:1:1 distribution are attributable to slight differences in the reduction rates of the substrates (see SI, Figure S4). This observation raises a key question: if a radical anion such as **B** is generated, why does it not undergo solution-phase radical addition to the excess styrene present (Figure 3E, pathway 1)?^16^ Cyclic voltammetry offers an initial clue. Whereas Bu_4_NBF_4_ affords the expected irreversible reductions for olefin **1** at −2.8 V (vs Ag/AgCl, Figure 3F-left), LiBF_4_ produces a sharp feature at −2.7 V (vs Ag/AgCl, Figure 3F-right) that is characteristic of a deposition process,^17^ implicating the formation of an electrochemically generated interphase (Figure 3F-left). This is further corroborated by the observed progressive decline in current intensity with successive cycles.

Next, we performed postelectrolysis surface analyses to support this interpretation. Interestingly, scanning electron microscopy (SEM) analysis reveals a continuous particulate film on the nickel cathode under the branched conditions, which is absent with the ammonium electrolyte (Figure 4A). To identify the composition of this layer, we performed solid-state NMR analyses (Figure 4B). ^7^Li NMR of the scraped film under argon atmosphere displays two resonances, one at 264 ppm attributable to Li^0^ and another near 0 ppm corresponding to Li^+^ species. To estimate the particle size of metallic lithium, a ^7^Li nutation experiment was performed, demonstrating that the entire lithium volume was efficiently excited and detected by the radio frequency pulses, suggesting Li^0^ domains of a few microns.^18^ The almost symmetric narrow line shape observed in a continuous wave electron paramagnetic resonance (EPR) measurement (Figure 4B) further supports the formation of ∼1 μm-scale lithium particles.^19^ Thus, both solid-state NMR and EPR spectra are consistent with the formation of nano- to micron-sized electronically “hot” Li^0^ embedded in the organics-containing matrix.

**Figure 4:**
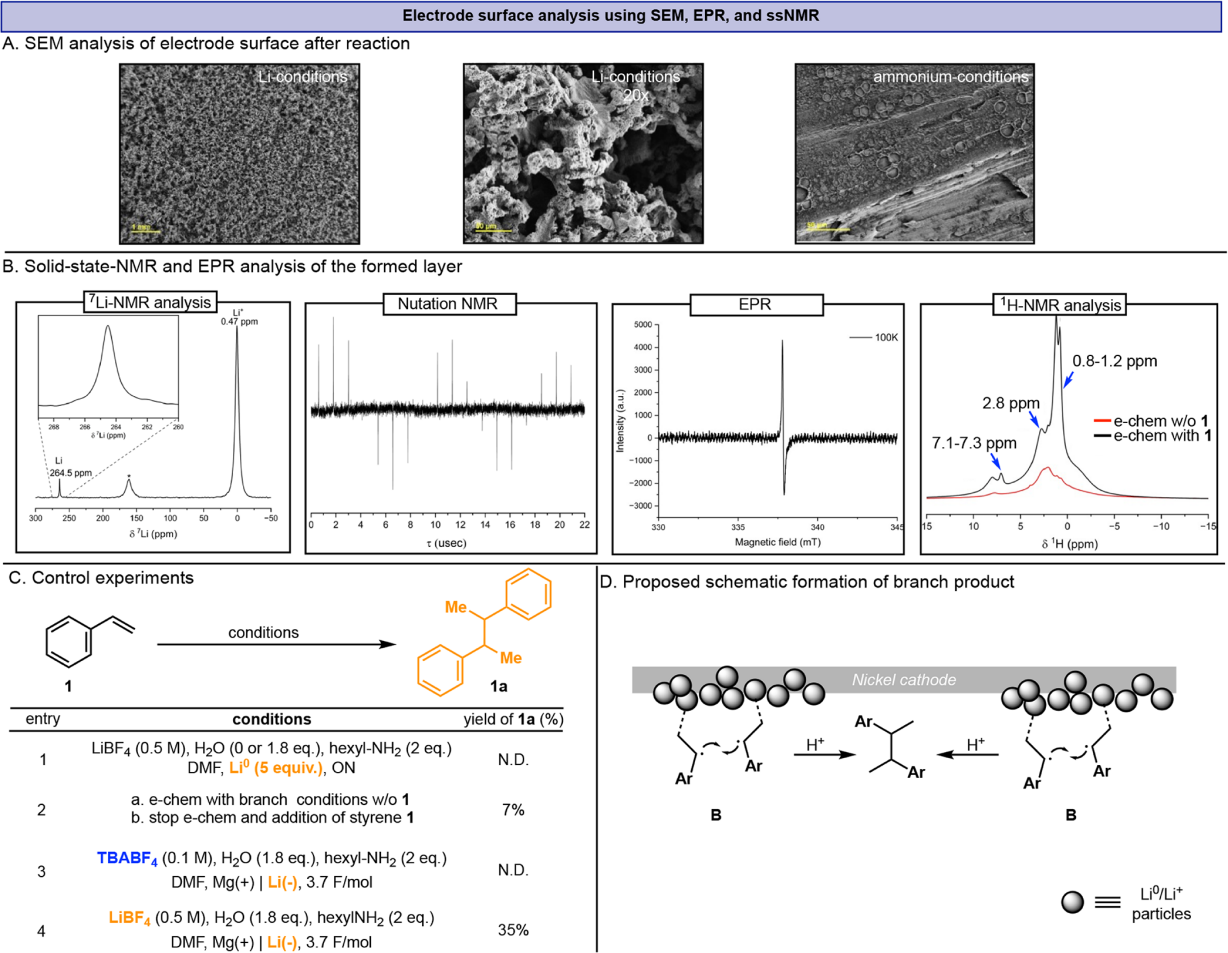
A) Electrode surface SEM analysis. Images were collected using the SE2 detector. Imaging conditions were as follows: aperture size, 30 μm; working distance (WD), 5 mm; electron high tension (EHT), 3.00 kV. B) Magnetic resonance analysis of the sample scraped from the surface of the electrode. Left to right: ^7^Li solid-state-NMR 1D magic angle spinning spectrum, ^7^Li nutation experiment, continuous wave X-band EPR spectrum, and ^1^H Hahn echo magic angle spinning spectra. C) Control experiments. D) Lithium particle-controlled selectivity.

This interfacial Li is not functionally equivalent to bulk Li^0^. Substituting electrolysis with five equivalents of lithium metal (with or without added water) fails to produce **1a** (Figure 4C-entry 1). Furthermore, Yorimitsu and coworkers showed that lithium-mediated styrene dimerizations generally favor a linear outcome rather than the pronounced branched selectivity observed in this case.^20^ Moreover, when the particles are first formed electrochemically in the absence of styrene, the current is then stopped, and only afterward styrene is introduced, **1a** still forms (7%, rest of the balance is attributed to the starting olefin **1**), indicating that the deposited layer both supplies reducing equivalents and imprints selectivity even in the absence of ongoing current (Figure 4C-entry 2). Similarly, olefin **4** furnished a 25% yield of the branched coupling product under similar experimental conditions. When applying a lithium cathode in combination with Bu_4_NBF_4_ electrolyte, no **1a** formation was observed (Figure 4C-entry 3). However, when running the same experiment with LiBF_4_, 35% of **1a** was obtained, accompanied by the formation of a Li layer (Figure 4C-entry 4).

Taken together, these results propose a surface-confined mechanism in which a Li-rich particulate passivation layer, chemically containing Li^0^/Li^+^, presumably creates a microenvironment that adsorbs and partially reduces styrene to a surface-bound radical (anion), increases its local concentration at the interface, and accelerates on-surface radical–radical recombination relative to solution-phase addition. Within this organized interphase, the putative surface-bound intermediate **B** (Figure 4D) accumulates to effectively high interfacial concentrations; short diffusion lengths, Li^+^ coordination, and restricted solvation collectively bias encounters toward bimolecular coupling on the surface rather than reaction in bulk solution. As evidence for the styrene adsorption to the Li-particle layer, we performed solid-state ^1^H NMR after electrolysis, where we observed characteristic peaks of reduced styrene resonating at ∼1, 2.8, and 7.2 ppm (Figure 4B-right). In this view, branch selectivity is not a simple consequence of a more potent reductant, but an emergent property of interfacial organization at a Li-particle layer. This organization channels reactivity through a surface-mediated radical–radical coupling pathway, with both Li^+^ and nanostructured Li^0^ playing crucial roles (see SI, Mechanism Discussion Section, for detailed mechanism).

Collectively, our results show that electrolyte identity governs selectivity probably through two coupled effects: (1) cation-specific interfacial interactions that stabilize and reorganize the styrene radical anion, shifting spin/charge localization and intrinsic reactivity; and (2) modulation of its mobility by the electrical double layer, bulky ammonium cations create a diffuse layer that enables desorption and diffusion into bulk, favoring radical addition and linear coupling. In contrast, Li^+^ generates a compact, Li-rich particulate interphase which facilitates a selective partial reduction of the olefin and channel reactivity through a surface-induced radical–radical coupling mechanism, thereby achieving branched selectivity.

Next, to assess the synthetic utility of the developed methods, we evaluated a representative substrate scope (Figure 5). Under both branched- and linear-selective conditions, mono-, di-, and trisubstituted olefins (α- and β-substituted; **5–7**) are converted to the coupling products with exclusive selectivity. Notably, both reaction conditions were effective for synthesizing adjacent quaternary–quaternary centers, affording the corresponding products (**5a** and **6b**) in good yields. The reaction also accommodates electron-rich arenes (**7**) and heterocycles (**10**) and tolerates common functional groups, including alcohols (**8**) and Bpin esters (**9**). In all cases, the remaining mass balance predominantly corresponds to olefin reduction. Finally, heterocoupling between two distinct olefins is feasible (Figure 5). Using three equivalents of the more electron-rich partner results in the cross-product with high selectivity (see the conditions in the Figure 5 caption). It is worth noting that the branched-selective conditions do not tolerate electron-deficient substituents (e.g., COOR or CN) on the aromatic ring, instead leading to over-reduction product and traces of linear coupling.

**Figure 5:**
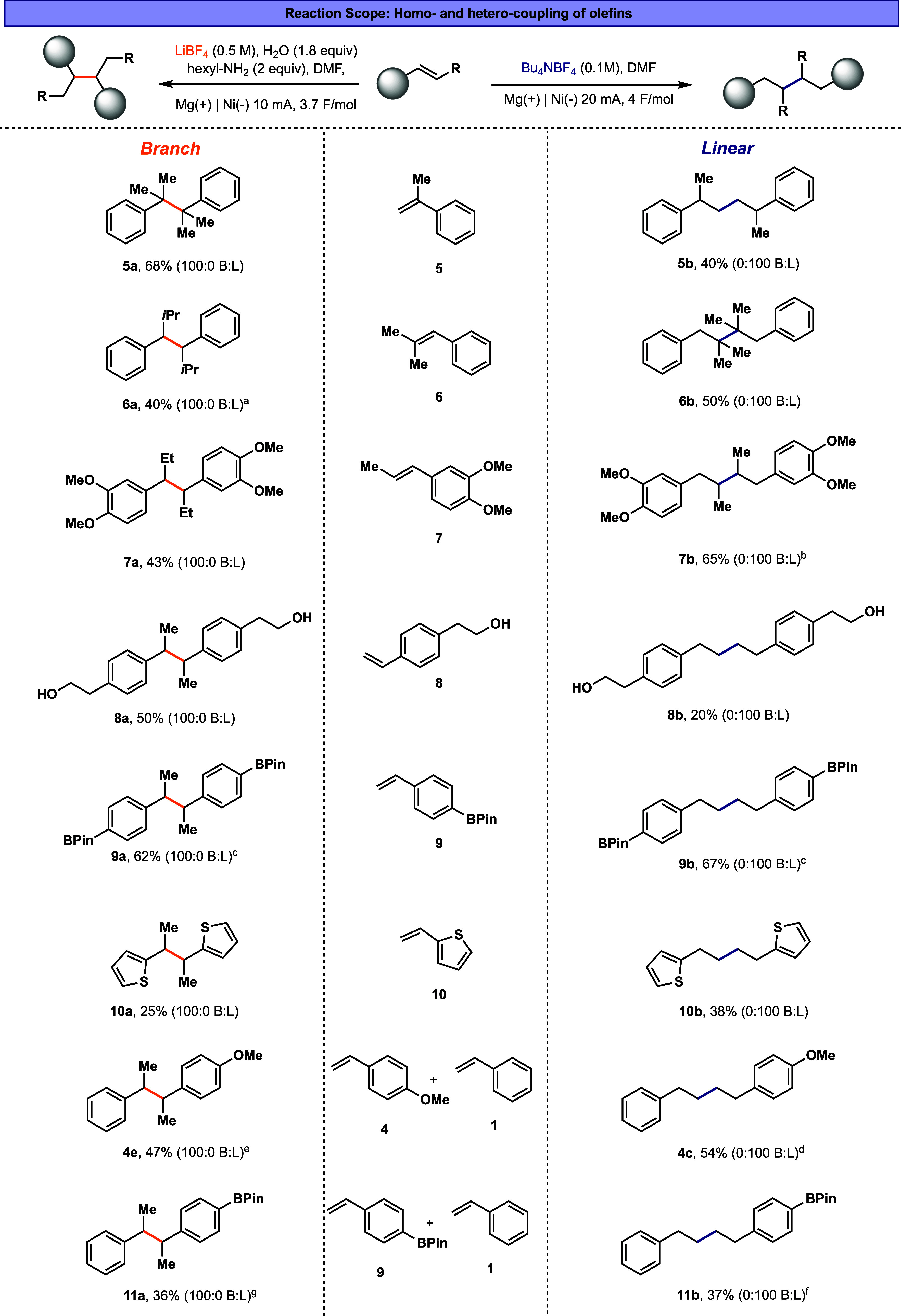
Linear and branched reaction scope. Heterocoupling conditions: linear: electron-rich olefin (3 equiv), TBABF_4_ (0.6 M), DMA (0.5 mL); branched: electron-rich olefin (3 equiv), LiBF_4_ (0.5 M), H_2_O (2 equiv), hexyl-NH_2_ (2 equiv), DMF (3 mL). ^a^d.r – 4:5, ^b^d.r – 1:3, ^c^NMR yield, ^d^24% **1b** product, ^e^40% **1a** product, ^f^24% **1b** product, ^g^32% **1a** product, B:L is branched:linear product ratio.

To further exemplify the potential applicability of the developed methodology, the reactions were applied to the synthesis of pharmaceutically relevant materials (Figure 6A). The efficient construction of quaternary centers with branch selectivity was applied in the synthesis of two antibrain tumor agents, **12** and **13**,^21^ in good selectivity and yield (Figure 6A). Similarly, the linear conditions were applied for the synthesis of an antibiotic adjuvant, **14**,^22^ and an antibreast cancer agent, **15**,^23^ (Figure 6A). In these cases, our methodology enabled a one-step synthesis, replacing the conventional two- and three-step routes, respectively.

**Figure 6:**
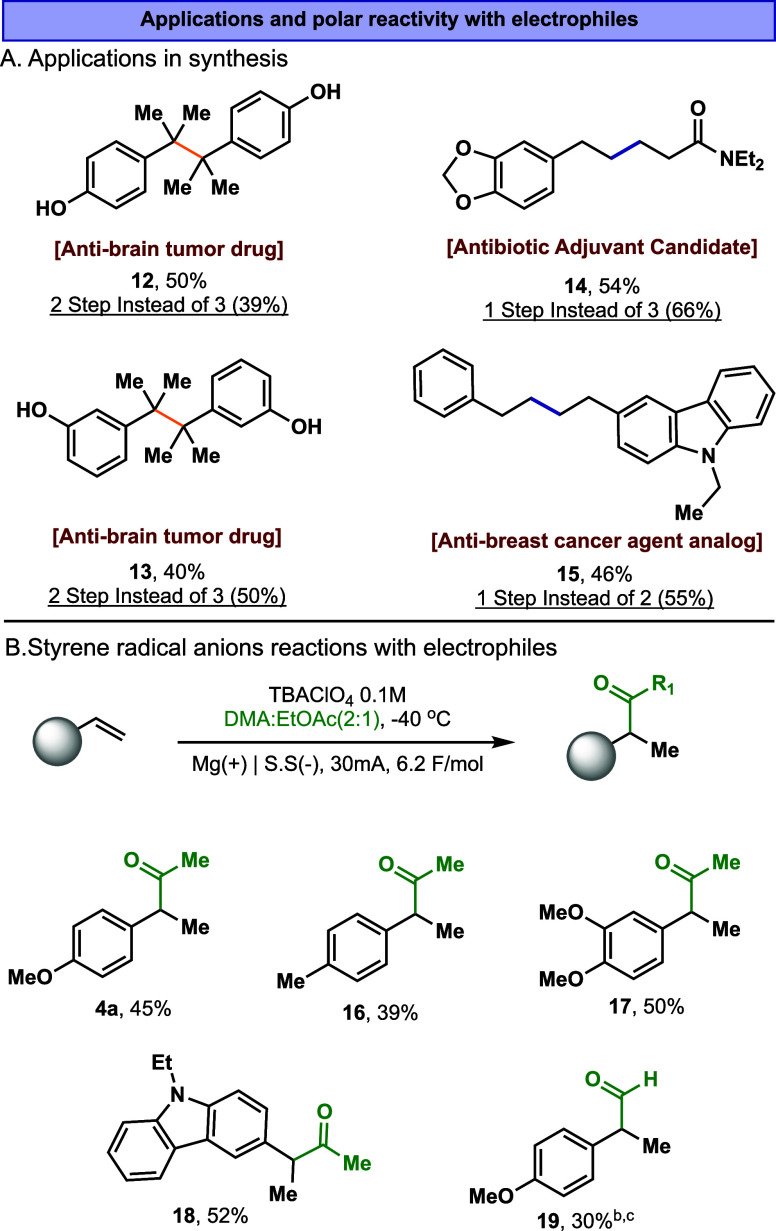
A) Application in the synthesis of biorelevant compounds. B) Internal hydroformylation and hydro-acylation of olefins. ^a^Total yield of two steps, coupling of anisole-styrene and demethylation with BCl_3_. ^b^DMF instead of DMA with 2 equiv of EtOAc. ^c^NMR yield.

Building on the conclusions of this study, we next applied the technique to probe the polar reactivity of the reduced styrene, aiming to promote its direct acylation. While the electrochemical polar hydrofunctionalization of activated olefins is well established,^9b,9c,24^ the internal (benzylic) selectivity of such intermediates has, to our knowledge, not been reported. Guided by these insights, we discovered that employing a Bu_4_N^+^ electrolyte (Bu_4_NClO_4_), in a DMA/EtOAc solvent mixture at −40 °C, led to the selective and efficient internal hydroacylation (Figure 6B). These conditions were applied to a series of activated olefins to form the corresponding hydroacylation products **4a** and **16–18**. Furthermore, these conditions can also be applied to the internal hydroformylation, **19**, using DMF instead of DMA.

## Conclusions

This study demonstrates how the nature of the supporting electrolyte cation is a decisive design element in the selective electro-reductive olefin functionalization. By toggling between bulky ammonium and hard lithium cations, the coupling of olefins can be selectively steered toward either solution-phase linear selectivity or surface-mediated branched products. Primarily, the nature of the electrolyte affects the radical anion distribution in the olefin framework as well as its diffusion behavior in the reaction mixture. The reported methodologies enable concise syntheses of pharmaceutically relevant diaryl scaffolds and unveil new opportunities to exploit electrolyte-controlled interfacial arrangement to guide selectivity in organic electrosynthesis. This level of electrolyte-driven control opens a distinct avenue in synthetic organic electrosynthesis, delivering a practical selectivity handle that conventional reagent manifolds cannot provide. The results presented here merit deeper investigation, both to elucidate the underlying mechanism and to unlock new synthetic avenues.

## Supplementary Material





## Notes

Cartesian coordinates of the optimized structures (PDF)
Experimental procedures for the synthesis of all new substances, together with characterization data, including computational details (PDF)
