# Supplementary material for: Electrolyte-Guided Selectivity Unlocks Pathway Control in Electrochemical Olefin Functionalization
Source: J Am Chem Soc. 2026 Apr 1;148(14):14866–76. doi: 10.1021/jacs.5c20366 (PMC13088248; doi:10.1021/jacs.5c20366)
Supplement: Supplementary file 1 [file ja5c20366_si_001.pdf]

Structures optimized at the ωB97X-D/cc-pVTZ level

Reduced Styrene Radical

C	-7.48524852930658	1.67319246367795	0.31284402680254
C	-6.29397015316809	2.09728424056995	-0.25453284849701
C	-8.78409531604785	1.70916799519569	-0.24369498961449
C	-9.92369544863755	1.24344502638819	0.48984188475830
H	-9.76696558781099	0.85312683246620	1.49179358071619
C	-11.19422935681343	1.27385025538849	-0.02762775593459
H	-12.01952412221693	0.90858439702071	0.57535420510697
C	-11.44477533118050	1.77193749745284	-1.32462429927894
H	-12.44707686686700	1.79569769362268	-1.73189886447408
C	-10.35075398707408	2.23145836493060	-2.06604081329418
H	-10.51299599298668	2.61892465546288	-3.06769389781217
C	-9.06642593754892	2.21014019567434	-1.56330006881896
H	-8.25278104317082	2.57553048262111	-2.17940269435426
H	-7.42928628666110	1.25685467274364	1.31844307479913
H	-6.24432952789122	2.53218848972569	-1.24607222887868
H	-5.36188651261818	2.01101673705901	0.28943168877423

Reduced Styrene Radical with N(Butyl)$_4^+$

C	-7.55225677454726	0.48600476574868	-1.63345054420121
C	-7.22973763986328	0.94280331166249	-2.89961804293482
C	-8.83553057851368	0.32348259756268	-1.06322078800241
C	-9.00260027353605	-0.19389323550136	0.26250619767546
H	-8.11320807710684	-0.44268361963166	0.83502309260493
C	-10.23932792701250	-0.39238193511413	0.82343340562289
H	-10.30470830224246	-0.79469170764976	1.82904041738877
C	-11.42130737910138	-0.08677150714643	0.11431553259988
H	-12.39584463099020	-0.24153452983587	0.55824849356166
C	-11.28939825115184	0.43010894008360	-1.17823034332268
H	-12.18162012224338	0.67464323157643	-1.74657025746346
C	-10.05563498443233	0.63196412316770	-1.76193250228132
H	-10.00783386324632	1.03662460655633	-2.76645544537195
H	-6.72292312631210	0.20632832100612	-0.98431066083307
H	-7.98241571624513	1.24390767559391	-3.61901296160851
H	-6.19597178911701	1.01911132126129	-3.21130741180526
H	-7.52889363222735	-4.56549444489513	-4.33246577250729
H	-7.21734501023503	-5.49186975224795	-2.87465707476139
H	-7.90186904425811	-2.32209144692441	-3.73194159071267
H	-7.18242797760782	-3.44231811290729	-2.10330553005910
C	-7.95065452917716	-5.31888754943513	-3.66497161125750
C	-8.15568640313697	-6.61632344283562	-4.44739080667556
C	-9.35327289709635	-1.39614635313863	-5.86139018651472
H	-9.16708847458330	-3.49841280844298	-5.46217085584665
C	-8.09862179550563	-3.24818779994983	-1.55057647524525
C	-8.98200256280014	-2.38903220773820	-3.61818134951981
C	-6.89090512198306	-3.85399640340430	0.53162974099654
C	-9.60473488755344	-2.62007737133004	-4.98368345329689
H	-8.10735346296238	-2.19063724552681	-1.28767381020328

C	-8.14570083792429	-4.10655185537176	-0.30165453664351
H	-9.31630877891500	-1.44319609359520	-3.19167631697367
C	-9.26267015022372	-4.82668399671234	-3.07556583027309
N	-9.23490560012703	-3.42279901894929	-2.53855816706099
H	-8.20029437382024	-5.16811043781589	-0.55409464683931
H	-10.67748501667344	-2.79338241648717	-4.92006828523704
H	-10.04313326532020	-4.85970443034575	-3.82893944374898
H	-9.58233399031254	-5.46147129826537	-2.25284654675838
H	-9.02258394925128	-3.86833095436351	0.30222745887477
C	-10.52084805883152	-3.11726130634561	-1.79108225512920
H	-10.29609582314905	-2.22823139096394	-1.20362570725703
H	-10.67427984807840	-3.94838278942434	-1.10604725713847
H	-11.66930282257445	-2.02668564621921	-3.26561866491267
C	-11.80235594170180	-2.85324660634369	-2.56534861997466
C	-12.50736000996559	-4.02929388629191	-3.23593652438813
H	-12.47238232867149	-2.45968797172097	-1.79538624588354
H	-6.00736417368260	-4.09579398454449	-0.06532921716037
C	-6.88230966672159	-4.66568335926283	1.81976224233235
H	-6.82534401867478	-2.78762768988544	0.76543263375242
H	-6.92322488078216	-5.73593495779278	1.60818103241778
H	-7.74261884639708	-4.41652898804807	2.44404302458993
H	-5.98050256920549	-4.47338063762744	2.40155085565692
C	-9.91945977014800	-1.57037075208400	-7.26412845093203
H	-8.27803507703800	-1.20374876064729	-5.91546580909377
H	-9.80045874650283	-0.51815366469273	-5.38667937023183
H	-9.46609870927295	-2.42859217096247	-7.76383682228515
H	-9.73284555156170	-0.68856393781516	-7.87746016468793
H	-10.99824945300450	-1.73477003348945	-7.23153317316270
H	-8.88411648699684	-6.44275959707212	-5.24476935275734
C	-8.60006242970644	-7.79374713179774	-3.58576628648634
H	-7.21375129722609	-6.86539918757271	-4.93920780758645
H	-9.58800956747062	-7.63414245213469	-3.15069578313076
H	-7.89787110889947	-7.96045874919009	-2.76603890744734
H	-8.64747440838816	-8.70927842215092	-4.17578937418667
C	-13.92288818766433	-3.65656942483533	-3.65934191990149
H	-12.53568054345846	-4.87612375987614	-2.54466578533797
H	-11.95941124455451	-4.36904760094366	-4.11647131801480
H	-14.52637434852695	-3.36898177255734	-2.79627318286479
H	-14.42006233568898	-4.49037851964623	-4.15549641568848
H	-13.91101054809271	-2.81293176671665	-4.35275846447291

Reduced Styrene Radical with 2Li$^+$

C	-7.83988654627554	3.08022726305222	-0.42668016238508
C	-7.47420442888428	3.32310010777436	-1.77011269692402
C	-8.18576111084127	1.79502827175495	0.10832206172998
C	-8.54908481190366	1.64217019796904	1.47885502323051
H	-8.56001218247012	2.52221471749454	2.11099108881616
C	-8.87886976529267	0.41757745452731	1.99486505538598
H	-9.15014607115985	0.33781438868656	3.04025619292793
C	-8.87015795843809	-0.73500028828457	1.18887764376742

H	-9.13427503017723	-1.69659127731132	1.60732570310916
C	-8.52203816386097	-0.62029496421556	-0.14214459953409
H	-8.51003628737713	-1.49782735358359	-0.77666952721487
C	-8.18257684264859	0.61495877071567	-0.69230560329971
H	-7.91825072583722	0.65145137133408	-1.74503426112479
H	-7.22291294786153	4.32346469095429	-2.08887436339738
H	-7.41856940641560	2.54442780079885	-2.52755713716974
H	-7.86621132516108	3.91914967455858	0.25905354091576
Li	-5.96776598013131	2.05455725783433	-0.68789023746293
Li	-9.61692341526351	2.68939091594020	-1.57202972137029

Reduced Styrene Radical (Compound 9)

H	1.49274053206222	0.08026937560853	2.25077711269294
C	1.00970582434622	0.03220552912547	1.28088325855584
C	-0.35454806943435	0.09659718234523	1.21345358049249
H	-0.91517802447959	0.19275820384509	2.13924162887113
C	-1.07481187214970	0.03831185139793	-0.01379975718031
B	-2.58678432269670	0.10863752626281	-0.06750416264146
C	-0.26828746506577	-0.09217176746902	-1.18797348737869
H	-0.75769387683830	-0.14018543007611	-2.15625527665824
C	1.09571872787702	-0.15528236527459	-1.13573902368729
H	1.66140658252987	-0.25198927160117	-2.05862878653674
C	1.81794745599368	-0.09809832050499	0.09869715926411
C	3.23512243411579	-0.17119000589562	0.12282613870988
C	4.07615351873258	-0.13518687343845	1.20348744950690
H	3.70188702084395	-0.26902409232276	-0.85695468856093
H	3.71305188904894	-0.04146212001851	2.22019858248352
H	5.14791332541409	-0.20413784562410	1.07015543819203
O	-3.41223491977472	0.34639110416721	1.02990396633570
O	-3.34671175164192	-0.05651551907831	-1.22378457059043
C	-4.70367753270174	0.31734379098782	-0.91914897490297
C	-4.77038261220900	0.08958528329478	0.62537763232810
C	-5.64948390799182	-0.54747072024520	-1.73299176910908
C	-4.87003802023643	1.78277328552200	-1.31006999409934
C	-5.08907440346353	-1.35490448957432	0.99926874814165
C	-5.69690022158452	1.03514676058807	1.36844304661523
H	-5.39209232526922	2.07254785514316	1.24504487277994
H	-5.68511281445361	0.79799186269668	2.43263192635300
H	-6.72128029434621	0.92792179212545	1.00826712159414
H	-4.44343384352417	-2.05192432980361	0.46375000780739
H	-6.12759396508352	-1.60463589480131	0.78149879817573
H	-4.92230427200743	-1.48562128572408	2.06862379878586
H	-4.20858355518762	2.42422624914598	-0.72661627604144
H	-5.89675177779959	2.11937139907822	-1.16622908950313
H	-4.61694336078002	1.89655316935910	-2.36430937599194
H	-5.45661813600072	-1.60713240338531	-1.57785098209079
H	-5.52250448211514	-0.32772746008203	-2.79344833521263
H	-6.68621148412894	-0.33787202577400	-1.46493571750017

Reduced Styrene Radical (Compound 9) with $N(Butyl)_4^+$

H	0.27894546279035	0.50475149999968	3.93337285606100
C	0.33429124061559	0.20247332055507	2.89334090037117
C	-0.78573847974752	0.29271749227906	2.11246408158394
H	-1.70085499378110	0.67178673462839	2.55865668491766
C	-0.80399217043339	-0.07802524600419	0.73922910007748
B	-2.05634605878417	0.04112954337219	-0.10615988477762
C	0.43870894476516	-0.55337203592191	0.21570836202657
H	0.48999642907343	-0.84376066260457	-0.82918529687476
C	1.56474490746418	-0.65027100524180	0.98367170698534
H	2.48683441967218	-1.01476071297483	0.53890154070211
C	1.58096975166538	-0.28357993571070	2.36673176707021
C	2.76926477789658	-0.38511376863045	3.13620367285567
C	2.96211498589659	-0.07173543141121	4.45484388068191
H	3.63401040736440	-0.76194113642346	2.59067644312731
H	2.16748626087908	0.31225695439819	5.08358589147922
O	-3.26441282597379	0.56775635189671	0.34441265520068
O	-2.15666332466216	-0.34883384662030	-1.43829766856627
C	-3.40744631822260	0.14961478558401	-1.95286802061299
C	-4.26579428928288	0.29538279977483	-0.65505576588252
C	-3.95527816987982	-0.83766875263590	-2.96735459189796
C	-3.12090049116400	1.48691066101267	-2.62852384484786
C	-4.96514214984326	-1.00016557876564	-0.25567841867241
C	-5.26445284918592	1.43788698194721	-0.67882046320762
H	-4.77146562627963	2.39757472214025	-0.81979930548748
H	-5.80766533948767	1.46717452485142	0.26617569348308
H	-5.98934396024356	1.29622780945066	-1.48197875403471
H	-4.26594490026422	-1.83694364664276	-0.23414838360867
H	-5.77667483296678	-1.24283649174551	-0.94156789339971
H	-5.38560082496892	-0.88003342506553	0.74294827304075
H	-2.73777440417278	2.21682453125933	-1.91473759769102
H	-4.01549875259475	1.89687353080919	-3.09714695916230
H	-2.36669451077899	1.33593683240984	-3.40110088896012
H	-4.02485433339791	-1.84066422973524	-2.55109379515275
H	-3.29964185241516	-0.87106005650138	-3.83799580836505
H	-4.94688003705051	-0.52882791438723	-3.30189685288138
H	3.93490625060946	-0.19147159650881	4.91333987235911
H	3.14907502981457	4.46856575676235	-1.15003931282236
H	1.64134057360513	5.33734878752122	-0.91736999799749
H	3.02175030684596	2.29412385310296	-0.20774992159320
H	1.15153202129251	3.26835354886187	-0.37575902850232
C	2.68099683863695	5.27967547232799	-0.58909428693052
C	3.38792759509677	6.59148010341922	-0.93041848366246
C	5.63518737741627	1.69057901751830	0.41861592122720
H	4.99512101078091	3.69975416811784	0.00919058255606
C	1.02214674857760	3.22095082512076	0.70267291507239
C	3.34183698343769	2.55706340477634	0.79870100425738
C	-1.35792961603291	3.76851219568642	0.22680976696092
C	4.81344565878376	2.93079099211062	0.76280002856826
H	0.85836224860323	2.17690798956142	0.96865142346434
C	-0.16284688138104	4.07013442649604	1.12678183256417
H	3.17740637965323	1.68211545358066	1.42772195982347

C	2.75116206657169	4.99459663484736	0.90267561947030
N	2.36212001075219	3.59774690092036	1.30371112527670
H	0.06909343250965	5.13549986201154	1.06186027292668
H	5.15923655336374	3.33075312324333	1.71508044995907
H	3.76292383507792	5.15203767065522	1.26073919400881
H	2.10348885876006	5.66410515727283	1.46263018485434
H	-0.43251317406750	3.85942260754564	2.16313779636715
C	2.20898678746984	3.50109057929962	2.81019146166574
H	1.70341610637346	2.54923693981453	2.97043731312326
H	1.52731687719970	4.30066000485283	3.09168147579266
H	4.14928188051156	2.76065097834735	3.42678076775654
C	3.44104613055804	3.54337333949512	3.70129008361566
C	4.14789489136772	4.88189924858949	3.89808867504627
H	3.04835387412084	3.22852978190630	4.67266814389001
H	-1.09737235719293	4.01409116173279	-0.80678412642806
C	-2.60627455608436	4.53426325951201	0.64036244937611
H	-1.56125385278149	2.69535180850235	0.24988165186643
H	-2.43470277941453	5.61227368750226	0.61509587034831
H	-2.90956511987419	4.26717227024974	1.65459939055323
H	-3.44029384225360	4.31278665297924	-0.02649388697905
C	7.12251355860446	1.99727530937982	0.31235726614023
H	5.27374029826654	1.26931246106529	-0.52361478519763
H	5.46645659845882	0.92771548591770	1.18396503319364
H	7.31393310753353	2.74108553842453	-0.46351559754840
H	7.69419001848963	1.10207345000136	0.06647061283046
H	7.50774834432073	2.39261827295124	1.25431625719444
H	4.43977276979427	6.51869584248374	-0.63966999491226
C	2.75574124548687	7.81969807133264	-0.28355618167551
H	3.37511703615560	6.70748199999815	-2.01554620979075
H	2.85511103991358	7.80530441658740	0.80297632541170
H	1.69112892056876	7.87904682592898	-0.52025028590024
H	3.22885581869422	8.73369754593555	-0.64307906030922
C	5.09567380637262	4.83863815545780	5.09066046501812
H	3.40047519188099	5.66639163758094	4.04579621811528
H	4.71873119024299	5.16125238147101	3.01093826642369
H	4.55482123533363	4.61291269591474	6.01167924238172
H	5.60607882708678	5.79277115940526	5.22375817322803
H	5.85728075158567	4.06795748307987	4.95397275197966

Reduced Styrene Radical (Compound 9) with 2Li$^+$

H	1.52996747628729	0.12306698850303	2.17454979464155
C	1.05678341074545	0.08995917438965	1.19706905598657
C	-0.33009842909697	0.12427691929915	1.14281094955004
H	-0.88097391074305	0.18248882075116	2.07445172066959
C	-1.02692672089265	0.08362282905387	-0.06198345948889
B	-2.57236243991838	0.12923395913384	-0.09707677013027
C	-0.25851748932096	0.00299696129090	-1.25098165542959
H	-0.77071844817482	-0.03033014374463	-2.20552866884514
C	1.10645488256868	-0.03061854591713	-1.23250505839148

H	1.66483783638033	-0.08933390524675	-2.15924677891442
C	1.83275544609000	0.01337329189899	-0.00286429409746
C	3.26175885147433	-0.01659729048062	0.01356508015576
C	4.06422734400208	0.02097227065885	1.16823686929251
H	3.75641975734232	-0.07319220269146	-0.94941918685627
H	3.66602222956507	0.07638697234164	2.17890725043247
O	-3.35671136513204	0.32436529370289	1.01336626593704
O	-3.31192487583702	-0.01853229348447	-1.24449529123608
C	-4.68586339815618	0.31537677268457	-0.92119622048801
C	-4.73051239206848	0.07058722935750	0.62351215391455
C	-5.60995843516721	-0.57016933953322	-1.73479498839591
C	-4.88724731871582	1.77732949407303	-1.30100986468452
C	-5.03407812886378	-1.37642950305568	0.99238020909677
C	-5.64039058232383	1.01031165070972	1.39108667971256
H	-5.33915255946674	2.04880781383920	1.27051362012852
H	-5.60913971152051	0.76291690835732	2.45214209508840
H	-6.67019492168274	0.90371035326790	1.04754413961397
H	-4.39320679819367	-2.06824616756228	0.44479121902704
H	-6.07406524279303	-1.62635049744924	0.78397308998263
H	-4.85553424092128	-1.51274528007319	2.05878107937699
H	-4.23329287197888	2.43126359999073	-0.72321183950946
H	-5.91948454509108	2.08625481901225	-1.13875861353887
H	-4.65355834980709	1.90289887849204	-2.35801770107433
H	-5.39086734655923	-1.62533126554795	-1.58591422522835
H	-5.49404069350666	-0.34021339456205	-2.79393430101292
H	-6.64920218568369	-0.38619466319151	-1.45904132191071
H	5.13953623402051	-0.00648528162260	1.07844841809890
Li	2.86342855024834	1.93514503630660	0.87299807314752
Li	2.79080138289163	-1.86369626295203	1.04149247537927

Neutral Methylated Styrene

H	2.60804987689843	-2.08291005982121	-3.87208456844160
C	3.41944313755986	-1.37396979736323	-3.99055020283497
C	3.32159780863836	-0.12233463113471	-3.38124163986576
C	2.11117439945311	0.19572380877447	-2.60158596639393
C	4.38339620320020	0.77197295614405	-3.54427219151971
H	4.34643260824084	1.75016910918869	-3.08209950357862
C	5.49534311501622	0.42520985618237	-4.29274312918275
H	6.30487350725435	1.13514871568166	-4.40737391964986
C	5.57675409779774	-0.82416994517305	-4.89646608012233
H	6.44693820258550	-1.09175006488782	-5.48200860545315
C	4.53313970176581	-1.72361775016845	-4.74108850821601
H	4.58329892760407	-2.70073982187962	-5.20475507141540
C	1.82816939461951	1.34749274639463	-1.99887806442290
C	0.57671492819452	1.61143814847480	-1.22585234670019
H	0.80836263336846	1.87786810073705	-0.19203494323881
H	-0.07991555239907	0.74147987173382	-1.21907396591438
H	0.03038917343363	2.45568975529839	-1.65248429107009
H	1.38758615561447	-0.61283177128745	-2.53169159942247
H	2.53134168115394	2.17345077310561	-2.05656540255701

Reduced Methylated Styrene Radical

H	2.62327222444380	-2.12193224147885	-3.88377022469071
C	3.42778326307836	-1.40039055978512	-4.00119119395483
C	3.28593278451415	-0.11843540573255	-3.37385207705730
C	2.13176211994311	0.21383753923589	-2.62486165789895
C	4.39572028977485	0.78364663981708	-3.56880482024004
H	4.36542017226138	1.77186943517656	-3.12426145213228
C	5.49908178337549	0.41821706904090	-4.31099094952551
H	6.30687036779528	1.13495724901307	-4.43067136397411
C	5.60809867077169	-0.84066826044209	-4.91421858892493
H	6.47978301443615	-1.11222609551245	-5.49524159429858
C	4.53418196442532	-1.74471525394999	-4.73540655472067
H	4.58222749420271	-2.73094377981704	-5.18660897450381
C	1.84375898211900	1.39083559481253	-1.96063022016469
C	0.56535488388570	1.62242238789302	-1.20887985504296
H	0.72978223980620	1.85986337263230	-0.15070295677479
H	-0.06988944100287	0.73334408991583	-1.24060146467623
H	-0.02603619579215	2.45257428235044	-1.61509556775002
H	1.37530274533011	-0.57096303453996	-2.56848487719043
H	2.55468263663167	2.21202697137044	-1.97857560647911

Reduced Methylated Styrene Radical+ Neutral Methylated Styrene -TS (R,R)

H	3.80324752870696	-1.77527985756595	-1.88285971119560
C	3.57892710473611	-0.73883967131881	-1.65220584124609
C	2.49967555862520	-0.46006078672680	-0.77547518106128
C	1.73044001628302	-1.52148288864441	-0.20854996641491
C	2.25390581051068	0.91162010424575	-0.51423448545418
H	1.44712680086144	1.19064028589996	0.15212123977608
C	3.02373283356352	1.90909371235724	-1.08660337359908
H	2.79768468365276	2.94501597926595	-0.85865470546596
C	4.07659726832115	1.60588692486548	-1.94486736102953
H	4.67301538847406	2.39114000386991	-2.39091814192659
C	4.34187852787276	0.26253567906391	-2.21699235008156
H	5.15587629297451	-0.00155780361838	-2.88292776753963
C	0.57966360222796	-1.38195998834971	0.56559994001265
C	0.03795939825567	-2.57325531181321	1.30594222866970
H	0.58523096955467	-2.75296143526318	2.23679806234839
H	0.11673168292647	-3.48131503252871	0.70282355932284
H	-1.01315944534661	-2.42861083230332	1.56581013304356
H	-4.78091452773975	1.75997390363478	2.99952639674151
H	-3.29728270871408	3.75762877428742	2.90370491382406
C	-3.89856678917362	1.75198683343986	2.36923484855199
C	-3.07091173122553	2.87532527280629	2.31924041138915
C	-3.60869187175683	0.62534167984476	1.62763519561493
C	-1.94807691613092	2.83074499128723	1.49796378721974
H	-4.26523221049821	-0.23671258945364	1.68691162750839
H	-1.29010486955323	3.69107038003678	1.44177302414486
C	-2.47226737533085	0.55093635894360	0.78221082488378

C	-1.64991836507983	1.70555951894482	0.74867862791678
H	-1.63451234401677	-1.83564359756296	-2.61444939308745
C	-2.20879757114707	-0.63260453659020	0.02787288758496
C	-1.08213221997314	-0.88429451233318	-0.75327289286000
H	-0.76692340024277	1.71380527432172	0.12151837326454
C	-1.08781409989761	-2.05863915618879	-1.69254682441631
H	-1.56881615763502	-2.92593361712145	-1.23311456137359
H	-0.07170820244167	-2.34348703892404	-1.97413751883595
H	2.00738911065054	-2.52578700500212	-0.52269604347404
H	-2.90658765213876	-1.45311827059230	0.18300362887472
H	-0.51695794884571	-0.02951975299324	-1.11505350239891
H	0.43029382869057	-0.43724199222106	1.08118991076810

Reduced Methylated Styrene Radical+ Neutral Methylated Styrene -TS (R,S)

H	3.31832445330535	-0.38332067674140	-2.84552681297995
C	2.99930703167333	-0.94603391955912	-1.97410438424183
C	2.06323111835327	-0.35032003262155	-1.09023424951606
C	1.57104426679514	0.96662487416473	-1.34142330887402
C	1.67932959245399	-1.13568865271134	0.02630515905207
H	0.94965948943204	-0.74442261558131	0.72440424609316
C	2.20301282116760	-2.39891850424079	0.23774724170319
H	1.88198043632164	-2.96271374380091	1.10706234146434
C	3.12474111864112	-2.95757345734941	-0.64475989132260
H	3.52733625431198	-3.94782535193994	-0.47515695233485
C	3.51246283383075	-2.20840136548444	-1.75673168850128
H	4.22665695816212	-2.61919024223447	-2.46181041714714
C	0.64000459865391	1.66313200892665	-0.58131357272045
H	0.47884194600124	1.34093411494841	0.44288462289005
H	-2.45648633484683	-2.45900991981263	3.65069927918026
H	-2.55424100702233	-0.37017702406316	5.00217913873231
C	-2.32879263771984	-1.49508384295673	3.17062733370391
C	-2.38381063754913	-0.32752323329499	3.93428632551931
C	-2.10887566071810	-1.44242887467405	1.80930544536633
C	-2.20748124560906	0.89236867455429	3.28668825253456
H	-2.06811711661169	-2.36484489924717	1.23898899889692
H	-2.23900424784437	1.81331133054422	3.85877896169521
C	-1.93071356972649	-0.21435754510135	1.12233207300732
C	-1.98320609005195	0.95613215327025	1.92276118691058
H	-2.31943011199019	0.64531756586278	-2.99917577974064
C	-1.68900201165965	-0.19595059617754	-0.28307648519148
C	-1.42163728091608	0.93381669364927	-1.04989411308922
H	-1.84113599454388	1.92538465050982	1.45993125919119
C	-1.33697890411358	0.81088822609191	-2.54480152519127
H	-0.69878305926330	-0.03226832450065	-2.82188459947149
H	-0.91146784768166	1.70649790559821	-3.00043236363517
H	1.90869271201919	1.42468703315948	-2.26921260159668
H	-1.56948550516324	-1.16878502501146	-0.75582220957126
H	-1.79347491506617	1.88773653886649	-0.68721485204450
C	0.46840895655341	3.14297021632400	-0.80687575609519
H	1.28278777146858	3.71354112770605	-0.34814522185034

H	-0.46430738861656	3.52082302760983	-0.38366957896137
H	0.47060920756938	3.38067170531801	-1.87371550186397

Reduced Methylated Styrene Radical+ Neutral Methylated Styrene - Product (R,R)

H	2.51690582122844	-2.17560681665699	-4.26718636021283
C	3.28112412826186	-1.41338945849479	-4.14042768207838
C	2.87950949295741	-0.13355349754874	-3.59707116775973
C	1.57093677714171	0.14283209474332	-3.25081532255117
C	3.97428282163548	0.80170610420327	-3.46245105262651
H	3.78290140485856	1.78292172666643	-3.04289168357402
C	5.26367199719983	0.49027302770490	-3.84635881523324
H	6.03497883583554	1.24509842695120	-3.71750044690539
C	5.60818957201881	-0.75394150753994	-4.37768331169443
H	6.62496962297609	-0.98680376607689	-4.66538288860118
C	4.57358989187341	-1.69488599389694	-4.50861135271094
H	4.79574956375619	-2.67888354096069	-4.91242549157742
C	1.10873474434403	1.44621919648971	-2.65752737712895
C	1.36906066716533	1.55177395665171	-1.14711578902414
H	2.42902771309173	1.39947964713088	-0.93531103385782
H	0.81005521190389	0.78527832691883	-0.60469954361022
H	1.07950343038141	2.52901386488545	-0.74660549079236
H	-4.37836678779064	-1.09902428479650	0.43356355743019
H	-4.40370190529336	0.90666021635165	1.89557119060755
C	-3.75933999956852	-0.24400454514432	0.19099579018634
C	-3.77650497852222	0.88224580952818	1.01398679112032
C	-2.95769295135834	-0.27767493587938	-0.92958734259594
C	-2.97471094739797	1.97477639077302	0.68932676621814
H	-2.94934732752192	-1.15844251828128	-1.56132770970477
H	-2.98153551430591	2.85417849114570	1.32180778480872
C	-2.12853848312092	0.81979725559674	-1.28371259494184
C	-2.16714179133130	1.95225990785752	-0.42994929839396
H	-1.63041017386266	2.29412140619158	-4.67259612949355
C	-1.32039168646473	0.73631062172230	-2.43569991073947
C	-0.38305782586408	1.77121773376471	-2.96527416695967
H	-1.55147019884294	2.81272094960276	-0.65451390491284
C	-0.60980231451150	1.96626224156819	-4.46693877890408
H	-0.43615531232294	1.03620027669817	-5.00994827220521
H	0.07652574498509	2.71818286947554	-4.86195024786968
H	0.83818323646383	-0.65115148528246	-3.36529354482093
H	-1.38723465121882	-0.18878798867397	-3.00065907194543
H	-0.59445364636628	2.72545131759865	-2.47514991797241
H	1.66986581758641	2.26783847901242	-3.12589617897257

Reduced Methylated Styrene Radical+ Neutral Methylated Styrene - Product (R,S)

H	2.20643718436660	-1.17527310985864	-5.76700685453717
C	2.96690987365159	-1.00833557192460	-5.00885023235186
C	2.71778124563800	0.00799835558427	-4.00968214004628
C	1.54672527088769	0.74459842419936	-3.98749908916769
C	3.79841843498259	0.15927354926601	-3.06209816234647

H	3.72152195172814	0.91581588027674	-2.28937972712151
C	4.93349900857612	-0.62633484190225	-3.10369132966421
H	5.70238321062694	-0.45990004491862	-2.35384787956054
C	5.12832622107526	-1.60987428493337	-4.07521259556605
H	6.02632234523776	-2.21318391148995	-4.09931166616224
C	4.10949111966262	-1.77003731551917	-5.02839228020458
H	4.22333138295823	-2.51804470579141	-5.80823620725807
C	1.25474544861622	1.84527722898011	-3.00473541864525
H	1.73196413691429	1.58901864981123	-2.04900305903335
H	-3.43601347017051	-1.44567264933962	1.03535653642126
H	-4.75160785993951	0.58864517345671	1.57141593988410
C	-3.19241052166679	-0.50286402156646	0.56116081838576
C	-3.93329310069001	0.63961590532065	0.86511416761201
C	-2.15036135283250	-0.44054693536291	-0.33862093263283
C	-3.60845552821626	1.84641607326619	0.24929277134867
H	-1.57889015381559	-1.33232044422391	-0.56878440662296
H	-4.17896730109615	2.73773451421963	0.48027502507005
C	-1.79802934524180	0.77471080093241	-0.98190338684718
C	-2.56681309603604	1.92104710044977	-0.65412380924023
H	-2.16707166851138	2.45028713511400	-3.58503513663378
C	-0.72472000708089	0.80089337450210	-1.89496745401918
C	-0.25003718234539	1.99135363663226	-2.66235527743961
H	-2.33424006180816	2.87096770836387	-1.11787141503304
C	-1.14048167631526	2.24288959692990	-3.89069704922579
H	-1.16118288225136	1.37265578935059	-4.54739270529090
H	-0.78547348085428	3.09829762117104	-4.46784036241347
H	0.83760590570036	0.58320613700063	-4.79436937970119
H	-0.20490175579305	-0.13395393257397	-2.08178186357007
H	-0.34433021680231	2.87519097614703	-2.02017225263936
C	1.84490258915043	3.20325386472645	-3.41889281623883
H	2.92365498457031	3.11211619192951	-3.55965059168531
H	1.66695207636324	3.97669282870118	-2.66428068160022
H	1.42179827076083	3.54982525307321	-4.36452909622252

Reduced Methylated Styrene Radical+ Reduced Methylated Styrene Radical - Product (R,R)

H	2.51283603819912	-2.10060879535997	-4.58283702768495
C	3.29920802753165	-1.35632382549859	-4.48800487505339
C	3.01114817441917	-0.15692605150981	-3.72485142428324
C	1.78634206994817	0.06652297925121	-3.13322607517633
C	4.13183147521407	0.76022237620357	-3.66005038166958
H	4.02733734955450	1.68135023758555	-3.09819354499978
C	5.33391502690678	0.50546649951551	-4.28852079961586
H	6.12715885808236	1.24312192038302	-4.19685260560730
C	5.56778134403370	-0.66070165775886	-5.02178038893763
H	6.51816022286575	-0.84789119435474	-5.50406249898002
C	4.50801324372859	-1.58128357117504	-5.09769514657535
H	4.64283347818657	-2.50437004087507	-5.65522721216408
C	1.43497653313986	1.29399244812268	-2.32915123562003
C	1.98682174056864	1.24446756172622	-0.89565378892349
H	3.06740040710074	1.08517424557313	-0.90247202402294

H	1.53053706532284	0.42292195270433	-0.33963759438542
H	1.78644015981498	2.17288857442474	-0.34919222926112
H	-4.58744629687173	-0.93174670861358	0.67523156603571
H	-5.62510423570900	1.34453432344505	0.80207778692329
C	-4.08761885102941	-0.08184653977451	0.21812420828298
C	-4.67907922234838	1.19093683705706	0.29971560405497
C	-2.89112365416387	-0.29143400143674	-0.42007518192134
C	-3.99135074415743	2.24329698100435	-0.31025630732232
H	-2.47485344877018	-1.29497579366840	-0.44966230618243
H	-4.41688978173342	3.24311860269125	-0.27847328335175
C	-2.13819128709250	0.77351753333679	-1.05439585230737
C	-2.78749347548093	2.06596565055674	-0.96194146817131
H	-1.60859611101871	2.29621543354690	-3.71115946400283
C	-0.92418755205651	0.55177753913556	-1.66856591392460
C	-0.07900043665223	1.62138893437157	-2.31452891648597
H	-2.31497038974274	2.92657977275656	-1.42180991860272
C	-0.55786607144658	1.99769567529705	-3.72555913732387
H	-0.46062241477109	1.14678499533882	-4.40308191876727
H	0.02236268381014	2.82808712265870	-4.14315865769002
H	1.02349230095450	-0.69890128386203	-3.24707365564992
H	-0.54001232999303	-0.46466074086383	-1.67314277088621
H	-0.16972638871441	2.53872815337593	-1.71182910764772
H	1.91344649236997	2.16358385468884	-2.80633645209865

Reduced Methylated Styrene Radical+ Reduced Methylated Styrene Radical - Product (R,S)

H	2.25009122967050	-1.21102311801182	-5.76749068374581
C	3.02201288592936	-1.01580976057837	-5.02762438529350
C	2.75997261020467	-0.00311066705314	-4.02312886738161
C	1.56983843231153	0.69180966877399	-3.96995498102635
C	3.86155476484069	0.18499523757548	-3.10059641961667
H	3.77402721935693	0.93634600284918	-2.32389641038621
C	5.02144080410884	-0.55910548887772	-3.17183169112897
H	5.80156313910185	-0.36630788992710	-2.43974234872837
C	5.22822274205434	-1.53772073566804	-4.14803013118331
H	6.14610269091084	-2.10897242371608	-4.19428534453081
C	4.19035296606933	-1.73439918667214	-5.07531886781846
H	4.31065491169134	-2.47968104538990	-5.85717998248143
C	1.25574389748324	1.78046942695204	-2.97556603708444
H	1.72699373361564	1.51697905923875	-2.01827546178278
H	-3.55901088708259	-1.35435517790885	1.12536768703072
H	-4.88127209268514	0.72549731996521	1.57311705734551
C	-3.30380916664806	-0.42968573643413	0.61445189143175
C	-4.05125563408835	0.73123430423696	0.87891578359539
C	-2.25272017765982	-0.43727122186487	-0.26783193363771
C	-3.67791904233083	1.88848403498224	0.19027348643589
H	-1.70467308613293	-1.36162250572468	-0.43023082556525
H	-4.23226755571162	2.80824302891225	0.35887639365809
C	-1.82825206117620	0.74149964029820	-0.99870158061066
C	-2.62824202826279	1.91414502279827	-0.70538799326291
H	-2.14472084403296	2.41850991308855	-3.57355527201614

C	-0.75842852470718	0.72584729474147	-1.86746789300339
C	-0.25056385127601	1.91825254551041	-2.63654566656597
H	-2.39970008876109	2.84609293592931	-1.21009316275752
C	-1.10892220008101	2.24289968651355	-3.87156716073591
H	-1.11131831355008	1.40865864460334	-4.57588208149909
H	-0.76337312771128	3.13505804526236	-4.40257917462017
H	0.83089786895534	0.47853252803073	-4.73776984361344
H	-0.23424264200300	-0.21449178545428	-2.02167388660020
H	-0.32080280571729	2.80320548773975	-1.98478389003160
C	1.85847023225937	3.13607111850814	-3.38356153139696
H	2.93807398225527	3.04419063134248	-3.52279296943619
H	1.68137332122953	3.90798608375292	-2.62651751159980
H	1.43959669756959	3.48998908167547	-4.32873831035556
