# Supplementary material for: Electrolyte-Guided Selectivity Unlocks Pathway Control in Electrochemical Olefin Functionalization
Source: J Am Chem Soc. 2026 Apr 1;148(14):14866–76. doi: 10.1021/jacs.5c20366 (PMC13088248; doi:10.1021/jacs.5c20366)
Supplement: Supplementary file 2 [file ja5c20366_si_002.pdf]

SUPPORTING INFORMATION

Electrolyte-Guided Selectivity Unlocks Pathway Control in Electrochemical Olefin Functionalization

Daniel Gordon-Levitan[#a], Dmitrii Bushmin[#a], Jonathan R. Church[#b], Rakesh Mondal[a], Tsafrir Bohak[a], Natalia Gloriozova[a], Moran Feller[a], Mark A. Iron[b], Haim Weissman[a], Michal Leskes[a], Boris Rybtchinski[a], and Samer Gnaim[*a]

[a] Department of Molecular Chemistry and Materials Science, Weizmann Institute of Science, Rehovot 7610001, Israel.

[b] Department of Chemical Research Support, Weizmann Institute of Science, Rehovot 7610001, Israel.

# These authors contributed equally to this study.

TABLE OF CONTENTS

**1.**	**Experimental details**		**S4 – S78**
	1.1.	Methods and materials	S5 – S6
	1.2.	Electrochemical equipment	S7
	1.3.	Synthetic Procedures of starting materials	S8
	Synthetic procedure and chemical analysis data of starting material (Compound **2**)		S9
	Synthetic procedure and chemical analysis data of starting material (Compound **3**)		S9
	Synthetic procedure and chemical analysis data of starting material (Compound **5**)		S10
	Synthetic procedure and chemical analysis data of starting material (Compound **6**)		S10
	Synthetic procedure and chemical analysis data of starting material (Compound **8**)		S11
	Synthetic procedure and chemical analysis data of starting material (Compound **9**)		S12
	Synthetic procedure and chemical analysis data of starting material (Compound **10**)		S12
	Synthetic procedure and chemical analysis data of starting material (Compound **S1**)		S13
	Synthetic procedure and chemical analysis data of starting material (Compound **S2**)		S13
	Synthetic procedure and chemical analysis data of starting material (Compound **S3**)		S13
	Synthetic procedure and chemical analysis data of starting material (Compound **S4**)		S14
	Synthetic procedure and chemical analysis data of starting material (Compound **S5**)		S14
	Synthetic procedure and chemical analysis data of starting material (Compound **S7**)		S15
	1.4	General procedures of electrochemical reactions	S16
	**General procedure A**: Linear homo-coupling general procedure		S16
	**General procedure B**: Linear hetero-coupling general procedure		S17
	Graphical guide for **A, B** electrochemical reaction setup		S18-S20
	**General procedure C**: Branched homo-coupling general procedure		S21
	**General procedure D**: Branched hetero-coupling general procedure		S22
	Graphical guide for **C, D** electrochemical reaction set-up		S23-S26
	**General procedure E**: α-hydrocarbonylation procedure		S27
	**General procedure F**: β-hydrocarbonylation procedure		S28
	**General procedure G**: α-hydroformylation procedure		S29
	1.5	Reaction study and optimization campaign	S30
	Electrolyte screening		S30

	Optimization of **procedure A**		S31-S34
	Optimization of **procedure B**		S35
	Optimization of **procedure C**		S36-S43
	Optimization of **procedure E**		S44-S48
	1.6	Synthetic procedures of electrochemical reactions	S49
	Reaction procedure and chemical analysis data of branch coupling product (Compound **1a**)		S49
	Reaction procedure and chemical analysis data of linear coupling product (Compound **1b**)		S50
	Reaction procedure and chemical analysis data of α-hydrocarbonylation (Compound **4a**)		S51
	Reaction procedure and chemical analysis data of β-hydrocarbonylation (Compound **4b**)		S52
	Reaction procedure and chemical analysis data of linear coupling product (Compound **4c**)		S53
	Reaction procedure and chemical analysis data of branch coupling product (Compound **4e**)		S54
	Reaction procedure and chemical analysis data of branch coupling product (Compound **5a**)		S55
	Reaction procedure and chemical analysis data of linear coupling product (Compound **5b**)		S56
	Reaction procedure and chemical analysis data of branch coupling product (Compound **6a**)		S57
	Reaction procedure and chemical analysis data of linear coupling product (Compound **6b**)		S58
	Reaction procedure and chemical analysis data of branch coupling product (Compound **7a**)		S59
	Reaction procedure and chemical analysis data of linear coupling product (Compound **7b**)		S60
	Reaction procedure and chemical analysis data of branch coupling product (Compound **8a**)		S61
	Reaction procedure and chemical analysis data of linear coupling product (Compound **8b**)		S62
	Reaction procedure and chemical analysis data of branch coupling product (Compound **9a**)		S63
	Reaction procedure and chemical analysis data of linear coupling product (Compound **9b**)		S64
	Reaction procedure and chemical analysis data of branch coupling product (Compound **10a**)		S65
	Reaction procedure and chemical analysis data of linear coupling product (Compound **10b**)		S66
	Reaction procedure and chemical analysis data of branch coupling product (Compound **11a**)		S67
	Reaction procedure and chemical analysis data of linear coupling product (Compound **11b**)		S68
	Reaction procedure and chemical analysis data of branch coupling product (Compound **12**)		S69
	Reaction procedure and chemical analysis data of branch coupling product (Compound **13**)		S70
	Reaction procedure and chemical analysis data of linear coupling product (Compound **14**)		S71
	Reaction procedure and chemical analysis data of linear coupling product (Compound **15**)		S72
	Reaction procedure and chemical analysis data of α-hydrocarbonylation (Compound **16**)		S73

	Reaction procedure and chemical analysis data of α-hydrocarbonylation (Compound **17**)		S74
	Reaction procedure and chemical analysis data of α-hydrocarbonylation (Compound **18**)		S75
	Reaction procedure and chemical analysis data of α-formylation (Compound **19**)		S76
	1.7	Synthetic procedures of electrochemical radical-clock reaction products	S77
	Reaction procedure and chemical analysis data of radical-clock, α-position (Compound **3a**)		S77
	Reaction procedure and chemical analysis data of radical-clock, α-position (Compound **3b**)		S77
	Reaction procedure and chemical analysis data of radical-clock, β-position (Compound **2e**)		S78
	Reaction procedure and chemical analysis data of radical-clock, β-position (Compound **2c**)		S79
**2.**	**Solid-state NMR**		**80**
**3.**	**Computational details**		**S81 – S84**
**4.**	**Additional data**		**S85 – S93**
**5.**	**Mechanism discussions**		**S94 - S98**
**6.**	**NMR, GC, HRMS spectra**		**S99 –S200**
**7.**	**References**		**S201 –S205**

1. Experimental details

1.1. Materials and Methods

Reagents were purchased at the highest commercial quality and used without further purification unless otherwise stated. Isolated yields refer to chromatographically and spectroscopically (^{1}H NMR) homogeneous material unless otherwise stated. Acetonitrile (MeCN), chloroform ($CHCl_3$), dichloromethane (DCM), *N,N*-dimethylformamide (DMF), *n*-pentane, *n*-hexane, benzene, toluene, dimethylsulfoxide (DMSO), diethyl ether, and tetrahydrofuran (THF) were obtained by passing the previously degassed solvents through an activated alumina column. For the determination of ^{1}H NMR yields, nitromethane or 1,3,5-trimethoxybenzene was used as an internal standard (automatic baseline correction was applied). Reactions were monitored by thin-layer chromatography (TLC) on 0.25 mm E. Merck silica plates (60 F_{254}), using short-wave UV light (254 nm) for visualization and *p*-anisaldehyde or potassium permanganate as developing agents. Flash column chromatography was performed using E. Merck silica gel (60, particle size 0.043–0.063 mm) or basic Al_2O_3. NMR spectra were recorded on Bruker Avance NEO-300, Avance NEO-400, Avance III HD-500, Avance NEO-600, and Avance III-800, and chemical shifts for ^{1}H- and ^{13}C NMR are reported relative to the solvent peaks (7.26 ppm for ^{1}H NMR in $CDCl_3$, 77.16 ppm for ^{13}C NMR in $CDCl_3$). The following abbreviations were used to explain NMR peak multiplicities: s = singlet, d = doublet, t = triplet, q = quartet, p = pentet, m = multiplet, br = broad. High-resolution mass spectra (HRMS) were recorded on an Agilent LC/MSD TOF mass spectrometer using an ESI ion source, a Waters LC-TOF (I-Class and G2-XS) mass spectrometer using ESI or APCI ion sources, and a Thermo Fisher Scientific LTQ Orbitrap XL mass spectrometer using an ESI ion source. GC-MS (EI) was performed on an Agilent 7820A GC system and a 5975 Series MSD, using *n*-decane as an internal standard.

Solid-state NMR measurements were performed on a 9.4T, 400 MHz Bruker Avance Neo solid-state NMR spectrometer. The measurements were performed using a 2.5 mm triple-resonance magic-angle-spinning probe with a sample spinning frequency of 25kHz.

For ^{7}Li (Larmor frequency of 155 MHz), direct excitation was used with radio-frequency pulses of 125 kHz amplitude and a relaxation delay of 20s. This delay was chosen following optimization to obtain a quantitative spectrum of both metallic and diamagnetic lithium environments. ^{7}Li shifts were calibrated relative to LiF at -1 ppm.

^{1}H NMR spectra were acquired with a rotor-synchronized Hahn echo using radio-frequency pulses

with 133 kHz amplitude and a 5s relaxation delay (during which the resonances were fully relaxed). The spectra were referenced to adamantane at 1.8 ppm.

Continuous-wave (CW) EPR measurements in the X-band (~9.4 GHz) were performed on a Bruker Magnettech ESR5000 spectrometer, with a modulation frequency of 100 kHz. Spectra were acquired at 100 K.

SEM images were obtained using a Zeiss Sigma 500 microscope. Unless otherwise specified, imaging conditions were as follows: aperture size, 30 μm; working distance (WD), 5 mm; electron high tension (EHT), 3.00 kV. Images were collected using the SE2 detector unless stated otherwise. Elemental analysis was performed using an EDS Bruker XFlash / 60 mm^2.

Benchtop cyclic voltammetry experiments were performed in a one-compartment glass cell. A homemade Ag/AgCl reference electrode (-0.45 V *vs.* ferrocene in MeCN; -0.54 V *vs.* ferrocene in acetone) and a coiled Pt wire counter electrode were used. All 3-mm glassy carbon (GC) electrodes were purchased from CH Instruments. Prior to the experiment, electrodes were polished using alumina paste. Before beginning electrochemical testing, the solution was purged with N_2 gas for at least 10 minutes to remove dissolved oxygen. Cyclic voltammetry experiments were conducted using a Biologic SP150 potentiostat.

1.2. Electrochemistry equipment

The electrochemical experiments were performed using an ElectraSyn 2.0 purchased from IKA (catalog number 0020008980). The nickel electrode (catalog number: 0040002859), nickel micro-electrode (catalog number: 0040004023), magnesium electrode (catalog number: 0040002848), and magnesium micro-electrode (catalog number: 0040004025) used in this work were bought from IKA (for a 0.3 mmol scale). Other electrodes used or tested in this work were obtained from IKA (https://www.ika.com/en). For experiments using a 5 mL ElectraSyn vial (catalog number: 0040003171), the electrode dimensions were approximately W8 x H52.5 x D2 mm, unless otherwise stated. For experiments using a 1 mL ElectraSyn micro-vial (catalog number: 0040003294), the electrode dimensions were approximately W3 × H56 × D1 mm, unless otherwise stated.

1.3. Synthetic procedures

Starting materials synthesis

The following substrates were purchased from various vendors:

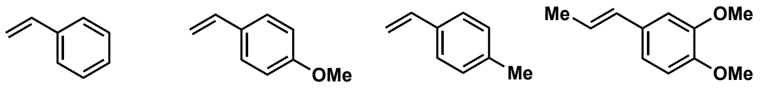

1
Sigma-Aldrich
[cat: S4972]

4
Aaron Chemicals
[cat: AR0034SK]

S6
Sigma-Aldrich
[cat: M80806]

7
Sigma-Aldrich
[cat: W246830]

General Wittig Olefination procedure[1]

An oven-dried 100 mL round-bottom flask was charged with the alkyl triphenyl phosphonium halide salt (1.5 equiv.) and a stirring bar, and the flask was dried under high vacuum for 1 h. Upon the removal from the high vacuum, the septum was removed to allow the quick addition of 20 mL of dry THF, along with 1.5 equiv. of potassium *tert*-butoxide. The septum was then replaced, the N_2 balloon was inserted with a needle, and the mixture was stirred and left at 0 °C in an ice bath for 30 minutes. After 30 minutes, 1 g of the aldehyde was added, and after another 30 minutes, the ice bath was removed, and the reaction was left overnight. Upon completion of the reaction (as determined by TLC and GC/MS), the reaction mixture was quenched with a saturated NH_4Cl solution and extracted three times using 30 mL of ethyl acetate. The combined organic layer was then washed with 20 mL of water and brine and dried over sodium sulfate. The solvent was removed under reduced pressure, and the product was purified by flash column chromatography.

General Suzuki cross-coupling procedure[2]

A crimp top sealed vial was charged with aryl bromide/halide substrate (1 equiv, 2.4 mmol) and vinyl boronic acid pinacol ester (1.1 equiv, 2.6 mmol), $Pd(OAc)_2$ (21.6 mg, 0.04 equiv, 0.096 mmol), SPhos (78.8 mg, 0.08 equiv, 0.192 mmol), and K_3PO_4 (1.53 g, 3 equiv, 7.21 mmol). The tube was purged with nitrogen, and 1,4-dioxane (9.6 mL) and water (0.216 mL) were added. The reaction tube was then sealed and heated to 80 °C for 2 h. The resulting solution was filtered through Celite® with EtOAc. The crude was washed with water (20 mL) and brine (20 mL). The combined organic extracts were dried over Na_2SO_4, filtered, and concentrated in vacuo. The product was purified by flash column chromatography.

Compound 2

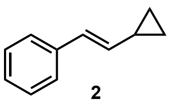

2

Following the general procedure for Suzuki coupling, iodobenzene (490 mg, 1 equiv, 2.4 mmol) was reacted with with (*E*)-2-(2-cyclopropylvinyl)-4,4,5,5-tetramethyl-1,3,2-dioxaborolane (527 mg, 1.1 equiv., 2.64 mmol), $Pd(OAc)_2$ (21.6 mg, 0.04 equiv., 0.096 mmol), SPhos (78.8 mg, 0.08 equiv., 0.192 mmol) and K_3PO_4 (1.53 g, 3 equiv., 7.21 mmol) for 2 h in 80 °C. Compound **2** was purified by flash column chromatography (SiO_2, 100% hexane), yielding 65% (255 mg, 1.77 mmol). ^{1}H NMR is consistent with the literature data.[3]

^{1}H NMR (400 MHz, $CDCl_3$): δ 7.40 – 7.11 (m, 5H), 6.49 (d, *J* = 15.8 Hz, 1H), 5.76 (dd, *J* = 15.8, 8.9 Hz, 1H), 1.68 – 1.50 (m, 1H), 0.88 – 0.74 (m, 2H), 0.57 – 0.49 (m, 2H).

Compound 3

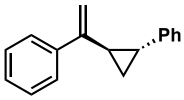

3

Following the general procedure for Wittig olefination, phenyl(2-phenylcyclopropyl)methanone (1 g, 1 equiv., 4.5 mmol) was reacted with methyltriphenylphosphonium bromide (2.41 g, 1.5 equiv., 6.75 mmol) and potassium *tert*-butoxide (757 mg, 1.5 equiv., 6.75 mmol) in dry THF (20 mL). Compound **3** was purified by flash column chromatography (SiO_2, 100% hexane), yielding 91% (900 mg, 4.09 mmol). ^{1}H NMR is consistent with the literature data.[4]

^{1}H NMR (300 MHz, $CDCl_3$): δ 7.62 – 7.46 (m, 2H), 7.40 – 7.12 (m, 8H), 5.39 (s, 1H), 5.09 (s, 1H), 2.09 – 1.92 (m, 2H), 1.43 (ddd, J = 8.6, 6.3, 5.0 Hz, 1H), 1.37 – 1.23 (m, 1H).

Compound 5

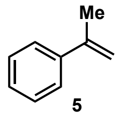

5

Following the general procedure for Wittig olefination, acetophenone (1 g, 1 equiv., 8.32 mmol) was reacted with methyltriphenylphosphonium bromide (4.46 g, 1.5 equiv., 12.48 mmol) and potassium *tert*-butoxide (1.4 g, 1.5 equiv., 12.48 mmol) in dry THF (20 mL). Compound **5** was purified by flash column chromatography (SiO_2, 100% hexane), yielding 91% (900 mg, 7.62 mmol). ^{1}H NMR is consistent with the literature data.[5]

^{1}H NMR (300 MHz, CDCl3): δ 7.60 – 7.23 (s 5H), 5.40 (s, 1H), 5.12 (h, J = 1.5 Hz, 1H), 2.23 – 2.16 (s, 3H).

Compound 6

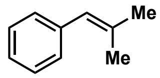

6

Following the general procedure for Wittig olefination, benzaldehyde (1 g, 1 equiv., 6.42 mmol) was reacted with *iso*-propyltriphenylphosphonium iodide (6.11 g, 1.5 equiv., 14.13 mmol) and potassium *tert*-butoxide (1.59 g, 1.5 equiv., 14.13 mmol) in dry THF (20 mL). Compound **6** was purified by flash column chromatography (SiO_2, 100% hexane), yielding 65% (815 mg, 6.16 mmol). ^{1}H NMR is consistent with the literature data.[6]

^{1}H NMR (300 MHz, $CDCl_3$): δ 7.39 – 7.13 (m, 5H), 6.27 (s, 1H), 1.91 (s, 3H), 1.87 (s, 3H).

Compound 8

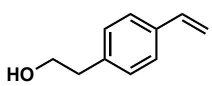

8

Following the general procedure for Suzuki coupling, 2-(4-bromophenyl)ethan-1-ol (482 mg, 1 equiv., 2.4 mmol) was reacted with with (*E*)-2-(2-cyclopropylvinyl)-4,4,5,5-tetramethyl-1,3,2-dioxaborolane (527 mg, 1.1 equiv., 2.64 mmol), $Pd(OAc)_2$ (21.6 mg, 0.04 equiv., 0.096 mmol), SPhos (78.8 mg, 0.08 equiv., 0.192 mmol) and K_3PO_4 (1.53 g, 3 equiv., 7.21 mmol) for 2 h in 80 °C. Compound **8** was purified by flash column chromatography (SiO_2, hexane:EtOAc 4:1), yielding 76% (270 mg, 1.82 mmol). ^{1}H NMR is consistent with the literature data.[7]

^{1}H NMR (300 MHz, $CDCl_3$): δ 7.47 – 7.07 (m, 4H), 6.73 (dd, *J* = 17.6, 10.9 Hz, 1H), 5.75 (dd, *J* = 17.6, 1.0 Hz, 1H), 5.24 (dd, *J* = 10.9, 1.0 Hz, 1H), 3.89 (q, *J* = 6.3 Hz, 2H), 2.89 (t, *J* = 6.5 Hz, 2H).

Compound 9

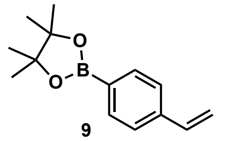

9

A mixture of (4-vinylphenyl)boronic acid (1.00 g, 1 equiv., 6.76 mmol), pinacol (800 mg, 1 equiv., 6.76 mmol), and DCM (33 mL) was combined in a 100 mL round-bottom flask. Anhydrous $MgSO_4$ (1.63 g, 2 equiv., 13.5 mmol) was added, and the resulting suspension was stirred at room temperature under nitrogen for 18 h. The mixture was then filtered, and the filtrate was concentrated under reduced pressure to afford **9** in 100% yield (1.53 g, 6.67 mmol). ^{1}H NMR is consistent with the literature data.[8]

^{1}H NMR (600 MHz, $CDCl_3$): δ 7.80 – 7.78 (m, 2H), 7.44 – 7.41 (m, 2H), 6.75 (dd, *J* = 17.6, 10.9 Hz, 1H), 5.84 (dd, *J* = 17.6, 0.9 Hz, 1H), 5.31 (dd, *J* = 10.9, 0.9 Hz, 1H), 1.37 (s, 12H)

Compound 10

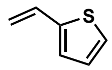

10

Following the general procedure for Wittig olefination, thiophene-2-carbaldehyde (1 g, 1 equiv., 8.92 mmol) was reacted with methyltriphenylphosphonium bromide (4.78 g, 1.5 equiv., 13.38 mmol) and potassium *tert*-butoxide (1.5 g, 1.5 equiv., 13.38 mmol) in dry THF (20 mL). Compound **10** was purified by flash column chromatography (SiO_2, 100% hexane), yielding 88% (865 mg, 7.85 mmol). ^{1}H NMR is consistent with the literature data.[9]

^{1}H NMR (300 MHz, $CDCl_3$): δ 7.24 – 7.14 (m, 1H), 7.08 – 6.94 (m, 2H), 6.84 (ddd, *J* = 17.4, 10.8, 0.9 Hz, 1H), 5.59 (dd, *J* = 17.3, 0.7 Hz, 1H), 5.16 (d, *J* = 10.8 Hz, 1H).

Compound S1

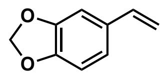

S1

Following the general procedure for Wittig olefination, benzo[*d*][1,3]dioxole-5-carbaldehyde (1 g, 1 equiv., 6.66 mmol) was reacted with methyltriphenylphosphonium bromide (3.57 g, 1.5 equiv., 9.99 mmol) and potassium *tert*-butoxide (1.12 g, 1.5 equiv., 9.99 mmol) in dry THF (20 mL). Compound **S1** was purified by flash column chromatography (SiO_2, hexane:EtOAc 4:1), yielding 95% (950 mg, 6.41 mmol). ^{1}H NMR is consistent with the literature data.[10]

^{1}H NMR (300 MHz, $CDCl_3$): δ 7.01 – 6.74 (m, 3H), 6.65 (dd, *J* = 17.5, 10.9 Hz, 1H), 5.98 (s, 2H), 5.60 (dd, *J* = 17.5, 0.9 Hz, 1H), 5.15 (dd, *J* = 10.8, 0.9 Hz, 1H).

Compound S2

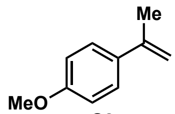

S2

Following the general procedure for Wittig olefination, 1-(4-methoxyphenyl)ethan-1-one (1 g, 1 equiv., 6.66 mmol) was reacted with methyltriphenylphosphonium bromide (3.57 g, 1.5 equiv., 9.99 mmol) and potassium *tert*-butoxide (1.12 g, 1.5 equiv., 9.99 mmol) in dry THF (20 mL). Compound **S2** was purified by flash column chromatography (SiO_2, hexane:EtOAc 4:1), yielding 97% (960 mg, 6.48 mmol). ^{1}H NMR is consistent with the literature data.[11]

^{1}H NMR (300 MHz, $CDCl_3$): δ 7.50 – 7.39 (m, 2H), 6.94 – 6.85 (m, 2H), 5.32 (s, 1H), 5.02 (s, 1H), 3.84 (s, 3H), 2.16 (s, 3H).

Compound S3

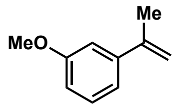

S3

Following the general procedure for Wittig olefination, 3-hydroxybenzaldehyde (510 mg, 1 equiv, 4.18 mmol) was reacted with methyltriphenylphosphonium bromide (2 g, 1.5 equiv., 8.12 mmol) and 50% sodium hydride in mineral oil (390 mg, 1.9 equiv., 8.12 mmol) in dry THF (20 mL). The product, 3-vinylphenol, was purified by flash column chromatography (SiO_2, 1:19 hexane:EtOAc), yielding 44% (222 mg, 1.65 mmol). Then, 3-vinylphenol (182 mg, 1 equiv., 1.5 mmol) was protected with a methyl group by reacting it with iodomethane (160 µL, 1.7 equiv., 2.6 mmol) and 50% sodium hydride in mineral oil (73 mg, 0.6 equiv., 73 mg), in 10 mL of dry THF. Compound **S3** was purified by flash column chromatography (SiO_2, hexane:DCM 9:1), with a yield of 84% (162 mg, 1.21 mmol). ^{1}H NMR is consistent with the literature data.[12]

^{1}H NMR (300 MHz, $CDCl_3$): δ 7.29 (t, J = 7.9 Hz, 1H), 7.16 – 7.00 (m, 2H), 6.87 (ddd, J = 8.2, 2.6, 1.0 Hz, 1H), 5.41 (s, 1H), 5.13 (s, 1H), 3.87 (s, 3H), 2.19 (s, 3H).

Compound S4

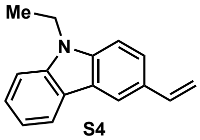

S4

Following the general procedure for Wittig olefination, 9-ethyl-9*H*-carbazole-3-carbaldehyde (1 g, 1 equiv., 4.48 mmol) was reacted with methyltriphenylphosphonium bromide (2.4 g, 1.5 equiv., 6.72 mmol) and potassium *tert*-butoxide (750 mg, 1.5 equiv., 6.72 mmol) in dry THF (20 mL). Compound **S4** was purified by flash column chromatography (SiO_2, hexane:EtOAc 4:1), yielding 75% (743 mg, 3.36 mmol). ^{1}H NMR is consistent with the literature data.[13]

^{1}H NMR (300 MHz, $CDCl_3$): δ 8.13 (dd, J = 9.7, 1.5 Hz, 2H), 7.63 – 7.55 (m, 2H), 7.44 – 7.20 (m, 3H), 6.94 (dd, J = 17.5, 10.9 Hz, 1H), 5.79 (dd, J = 17.6, 1.0 Hz, 1H), 5.22 (dd, J = 10.9, 1.1 Hz, 1H), 4.39 (q, J = 7.2 Hz, 2H), 1.46 (t, J = 7.2 Hz, 3H).

Compound S5

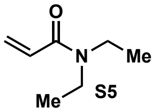

S5

A solution of diethylamine (2.24 mL, 21.5 mmol) and triethylamine (3.01 mL, 21.7 mmol) in DCM (70 mL) was stirred in an ice bath under a N_2 atmosphere. A solution of acryloyl chloride (2.00 g, 1.80 mL, 22.1 mmol) in DCM (15 mL) was added dropwise to the cold, stirring amine solution over 75 minutes using an addition funnel. The ice bath was removed, and the mixture was stirred overnight at room temperature under N_2. The reaction mixture was washed successively with saturated aqueous NH_4Cl (40 mL), water (40 mL), and saturated aqueous $NaHCO_3$ (40 mL). The combined aqueous layers were extracted with DCM (2 × 30 mL), and the combined organic

extracts were washed with brine (40 mL), dried over Na_2SO_4, filtered, and concentrated under reduced pressure to afford an orange oil. The crude product was purified by flash column chromatography (hexane:EtOAc 1:1) to afford **S5** in an 80% yield (2.25 g, 17.69 mmol). ^{1}H NMR is consistent with the literature data.[14]

^{1}H NMR (300 MHz, $CDCl_3$): δ 6.57 (dd, *J* = 16.8, 1.8 Hz, 1H), 6.36 (dd, *J* = 16.7, 3.0 Hz, 1H), 5.68 (dd, *J* = 10.3, 2.2, 1H), 3.43 (q, 2H), 3.42 (q, 2H), 1.21 (t, 3H), 1.19 (t, 3H).

Compound S7

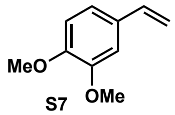

S7

Following the general procedure for Wittig olefination, 3,4-dimethoxybenzaldehyde (1 g, 1 equiv., 6.02 mmol) was reacted with methyltriphenylphosphonium bromide (2.4 g, 1.5 equiv., 9.03 mmol) and potassium *tert*-butoxide (1 g, 1.5 equiv., 9.03 mmol) in dry THF (20 mL). Compound **S7** was purified by flash column chromatography (SiO_2, hexane:EtOAc 4:1), yielding 90% (890 mg, 5.42 mmol). ^{1}H NMR is consistent with the literature data.[15]

^{1}H NMR (300 MHz, $CDCl_3$): δ 7.04 – 6.78 (m, 3H), 6.68 (dd, *J* = 17.6, 10.9 Hz, 1H), 5.64 (dd, *J* = 17.5, 0.9 Hz, 1H), 5.17 (dd, *J* = 10.8, 0.9 Hz, 1H), 3.92 (s, 3H), 3.91 (s, 3H).

General procedure A: Linear homo-coupling general procedure

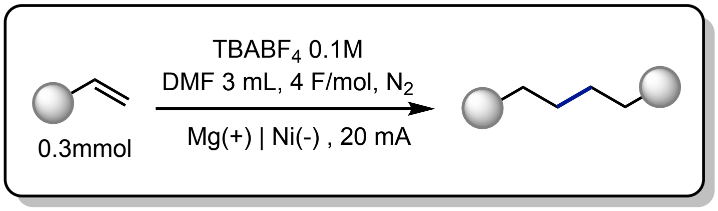

A 5 mL Electrosyn vial was wrapped with Teflon tape on the screw thread, then charged with $TBABF_4$ (0.3 mmol, 98.78 mg), 3 mL of dry DMF to form a 0.1 M solution, and 0.3 mmol of the olefin substrate. The vial was then sealed with a septum-equipped Electrosyn cap, carried with a nickel cathode and magnesium anode (which was polished ahead). Using an N_2 balloon and a long needle inserted through the septum, the reaction mixture was purged for 10 minutes, then maintained under an N_2 atmosphere throughout electrolysis. The electrolysis was set to 20 mA constant current, 1500 rpm stirring, and usually for 4 F/mol, or until the voltage suddenly rose to 30 V, depending on the substrate. Upon completion, the crude was diluted with 1 M aqueous HCl (30 mL) and extracted with Et_2O (3 × 30 mL). The organic layers were combined, washed with water (20 mL), brine (20 mL), and dried over Na_2SO_4. The organic mixture was concentrated under vacuum, and the product was purified using PTLC (hexane:EtOAc).

Note: The N_2 degassing step is crucial, as any oxygen present can significantly harm the reaction outcome by increasing the proportion of side products. Similarly, using a well-dried, degassed solvent is crucial. Seeing gas evolution during electrolysis usually indicates that the steps above were not carried out correctly.

General procedure B: Linear hetero-coupling general procedure

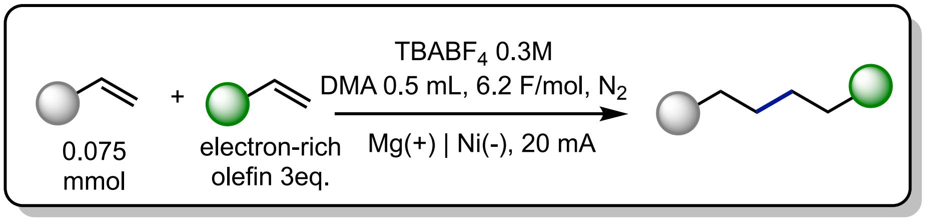

A 1 mL Electrosyn micro-vial was wrapped with Teflon tape on the screw thread, charged with $TBABF_4$ (0.15 mmol, 49.38 mg), and 0.5 mL of dry DMA to form a 0.3 M solution. 0.075 mmol of the limiting substrate was then added to the vial, along with 3 equiv. of the electron-rich olefin. The vial was partially sealed with an Electrosyn cap equipped with a micro-nickel cathode and a micro-magnesium anode (which had been polished beforehand). Using an N_2 balloon and a long needle, the reaction mixture was purged for 10 minutes. Then, the cap was sealed, and the reaction mixture was kept under a N_2 atmosphere throughout the electrolysis. The electrolysis was set to 20 mA constant current, 1500 rpm stirring, and usually ran for 6.2 F/mol or until the voltage suddenly rose to 30 V, depending on the substrate. Upon completion, the crude was diluted with 1 M aqueous HCl (30 mL) and extracted with Et_2O (3 × 30 mL). The organic layers were combined, washed with water (20 mL), brine (20 mL), and dried over Na_2SO_4. The organic mixture was concentrated under vacuum, and the product was purified using PTLC (hexane:EtOAc).

Note: The N_2 degassing step is crucial, as any oxygen present can significantly harm the reaction outcome by increasing the proportion of side products. Similarly, using a well-dried, degassed solvent is crucial. Seeing gas evolution during electrolysis usually indicates that the steps above were not carried out correctly.

__General graphical guide of procedures A and B__

1. Wrap the screw thread of the Electrosyn 5 mL or 1 mL vial with Teflon tape, and equip the Electrosyn cap with a nickel cathode and a polished magnesium anode (for the 1 mL vial, use Electrosyn micro-electrodes).

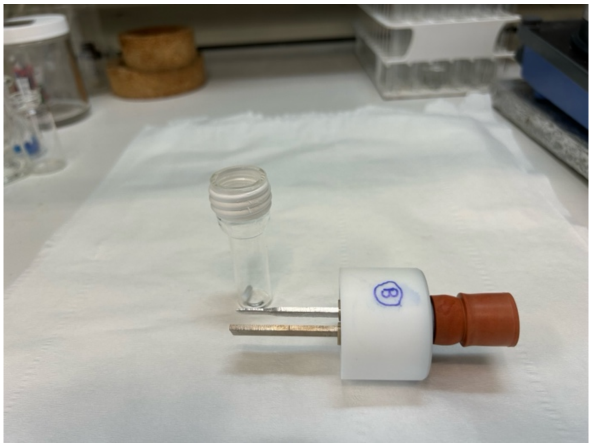

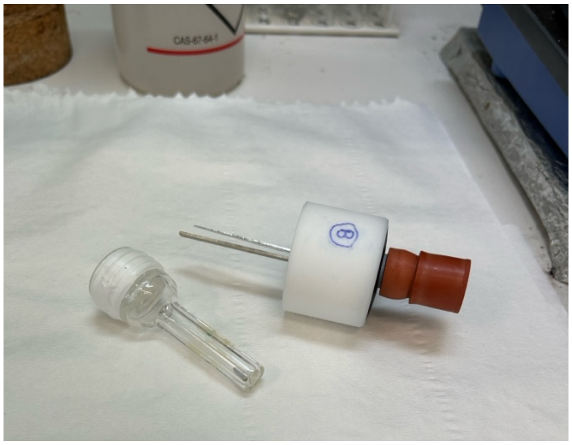

2. Charge the electrolyte into the reaction vial, which is pre-equipped with a magnetic stirring bar. If one of the substrates is solid, it will also be charged to the vial at this point.

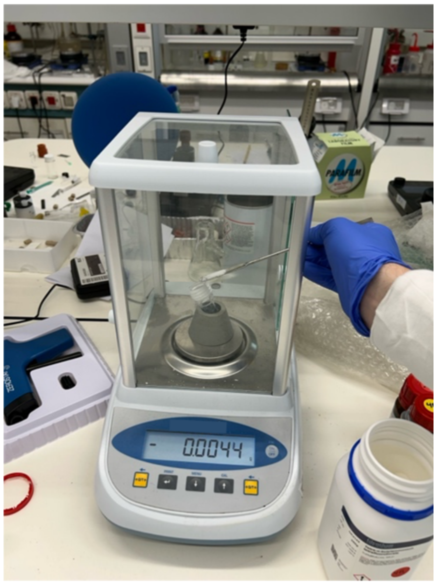

3. The relevant dry solvent is syringed, degassed with a N_2 balloon, and directly transferred to the reaction vessel.

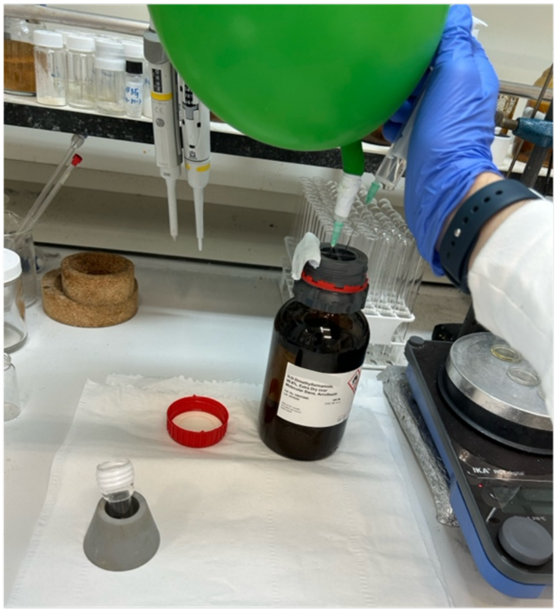

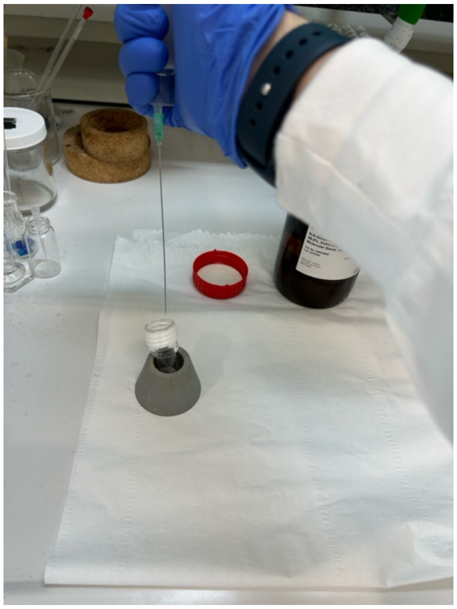

4. The liquid substrates are charged into the vial using a pipette.

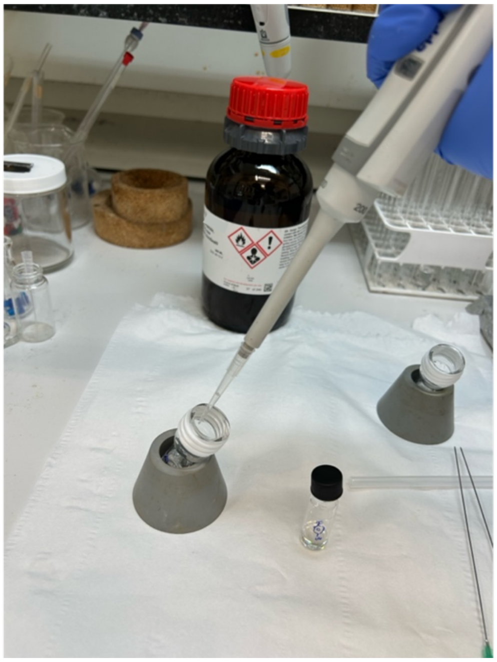

5. The vial is partially screwed onto the cap, which carries the electrodes and a septum. N_2 is purged for 10 minutes using a balloon and a long needle whose tip reaches the bottom of the vial.

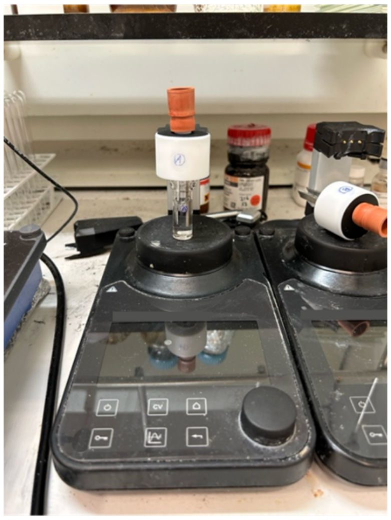

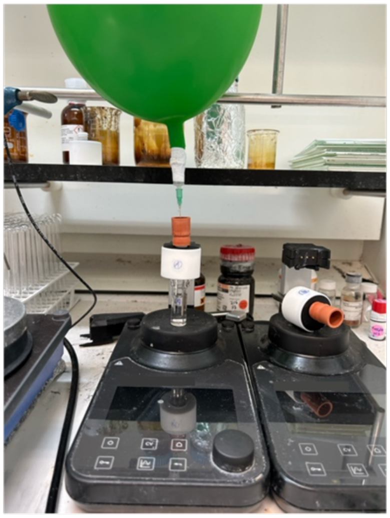

6. After 10 minutes, the cap is screwed fully up until no bubbles are coming from the needle. Then, the needle tip is pulled out of the solvent, and electrolysis is initiated at 20 mA, 1500 rpm, and for the relevant time. Usually, the signal for reaction completion is a sudden rise in voltage to 30 Volts.

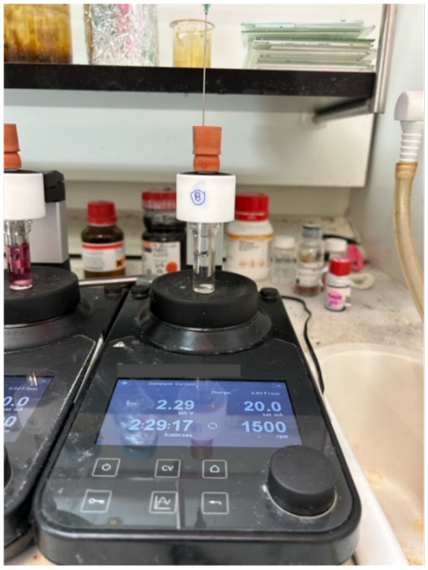

General procedure C: Branched Homo-coupling procedure

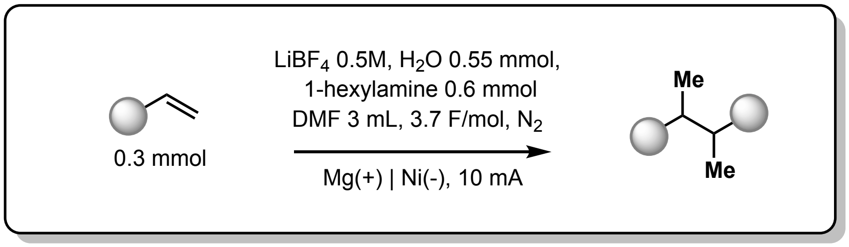

A 5 mL ElectraSyn vial was equipped with $LiBF_4$ (1.5 mmol, 140.6 mg, 5 equiv.) and a magnetic stir bar, dried under high vacuum at 155 °C for 1 h. Then it was cooled down to room temperature and filled with N_2. A separate dry glass vial was equipped with 0.3 mmol of the olefin substrate, 3 mL of dry DMF, water (10 µL, 0.55 mmol, 1.8 equiv), and hexylamine (0.6 mmol, 79.3 µL, 2 equiv.). The resulting mixture was transferred to the ElectraSyn vial with dried $LiBF_4$ under N_2. The vial thread was wrapped with Teflon tape and capped with an ElectraSyn cap equipped with a nickel cathode, magnesium anode, and septum. Then the reaction mixture was purged with N_2, while stirring to dissolve the salt, for 10 minutes. The electrolysis was carried out under constant current conditions at 10 mA for 3.7 F/mol (3 h), with stirring at 1000 rpm using an IKA ElectraSyn. Upon completion, the crude was diluted with 1 M aqueous HCl (30 mL) and extracted with Et_2O (3 × 30 mL). The organic layers were combined, washed with water (20 mL), brine (20 mL), and dried over Na_2SO_4. The organic mixture was concentrated under vacuum, and the product was purified using PTLC (hexane:EtOAc).

Note: LiBr can also be used as an alternative to $LiBF_4$. Drying the salts at higher temperatures leads to their decomposition. It is important to minimize the reaction mixture's interaction with the atmosphere and to seal the reaction on the first attempt. Repeated exposure to the atmosphere leads to the absorption of atmospheric moisture. Based on our experience, water does not negatively affect the reaction itself; however, at high concentrations, it causes rapid anode degradation, a sharp increase in resistance, and halts the reaction (especially with $LiBF_4$). For purging, we prefer to slightly open the vial head to allow 2-3 bubbles/second for 10 minutes.

<u>**General procedure D: Branched Hetero-coupling procedure**</u>

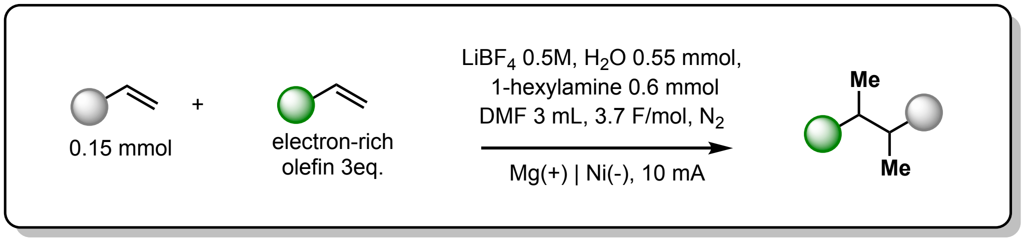

A 5 mL ElectraSyn vial was equipped with $LiBF_4$ (1.5 mmol, 140.6 mg, 5 equiv) and a magnetic stir bar, dried under high vacuum at 155 °C for 1 h. Then it was cooled down to room temperature and filled with N_2. A separate dry glass vial was equipped with 0.15 mmol of the olefin substrate and 3 equiv. of the electron-rich olefin, 3 mL of dry DMF, water (10 µL, 0.55 mmol, 1.8 equiv.), and hexylamine (0.6 mmol, 79.3 µL, 2 equiv.). The resulting mixture was transferred to the ElectraSyn vial with dried $LiBF_4$ under N_2. The vial thread was wrapped with Teflon tape and capped with an ElectraSyn cap equipped with a nickel cathode, magnesium anode, and septum. Then the reaction mixture was purged with N_2, while stirring to dissolve the salt, for 10 minutes. The electrolysis was carried out under constant current conditions at 10 mA for 3.7 F/mol (3 h), with stirring at 1000 rpm using an IKA ElectraSyn. Upon completion, the crude was diluted with 1 M aqueous HCl (30 mL) and extracted with Et_2O (3 × 30 mL). The organic layers were combined, washed with water (20 mL), brine (20 mL), and dried over Na_2SO_4. The organic mixture was concentrated under vacuum, and the product was purified using PTLC (hexane:EtOAc). Same Notes as for procedure C.

General graphical guide of procedures C and D

1. Wrap the screw thread of the Electrosyn 5 mL or 1 mL vial with Teflon tape, and equip the Electrosyn cap with a nickel cathode and a polished magnesium anode (for the 1 mL vial, use Electrosyn micro-electrodes).

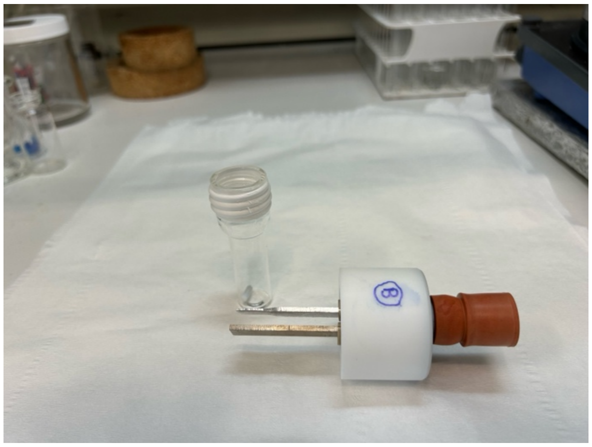

2. Charge the lithium electrolyte into the reaction vial, which is pre-equipped with a magnetic stirring bar.

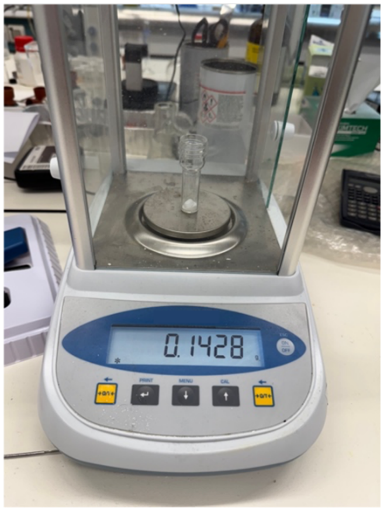

3. Equip the vial with a septum and dry under high vacuum at 155 °C for 1 h.

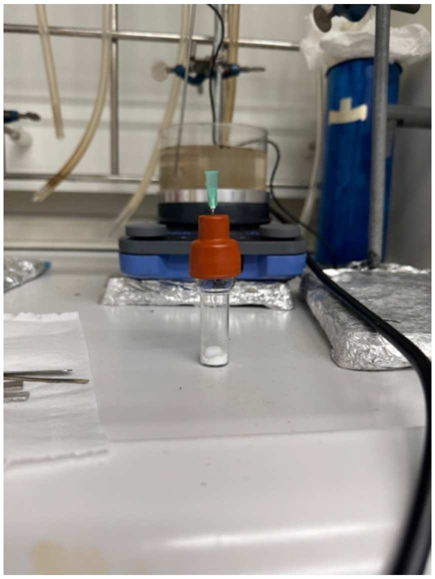

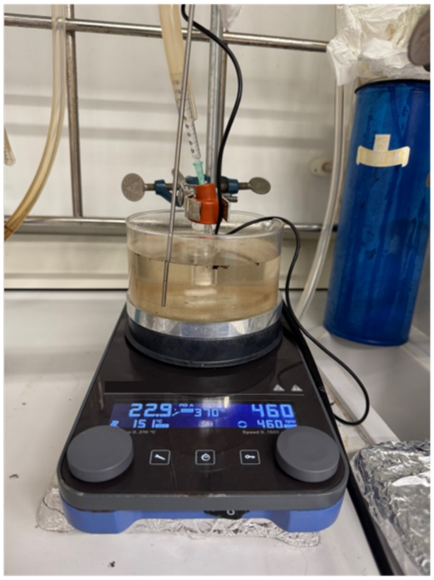

4. The relevant dry solvent is syringed, degassed with a N_2 balloon, and directly transferred to the reaction vessel.

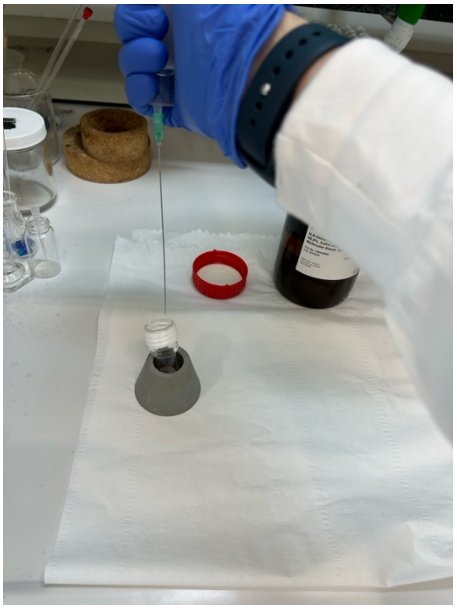

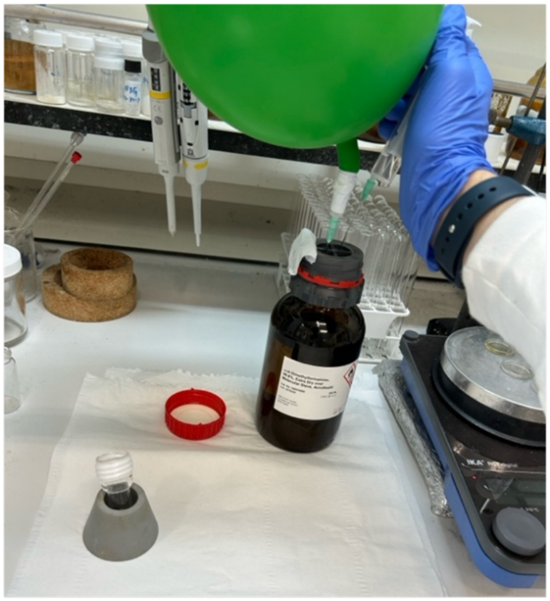

5. The liquid substrates and the solvent were pre-prepared and charged into a separate vial.

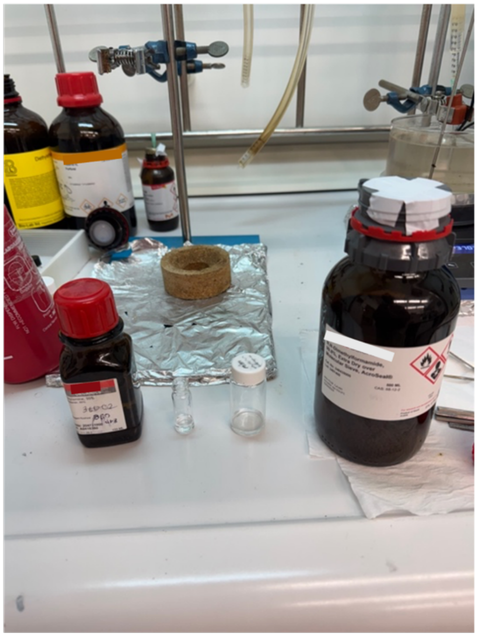

6. When the Electrasyn vial containing the electrolyte is dry, the pre-prepared reaction mixture is transferred to it through a septum using a syringe.

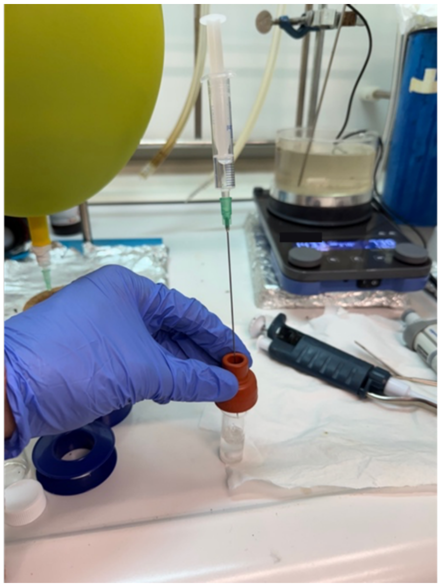

7. The vial is partially screwed onto the cap, which carries the electrodes and a septum. N_2 is purged for 10 minutes using a balloon and a long needle whose tip reaches the bottom of the vial, while stirring to dissolve the electrolyte.

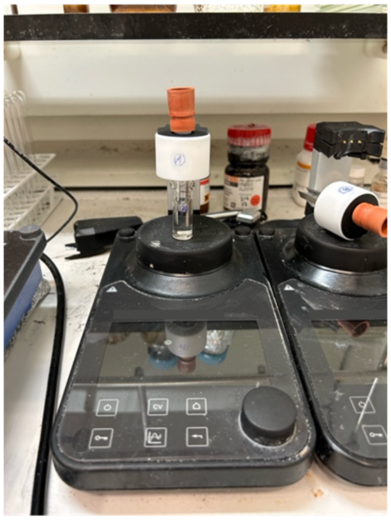

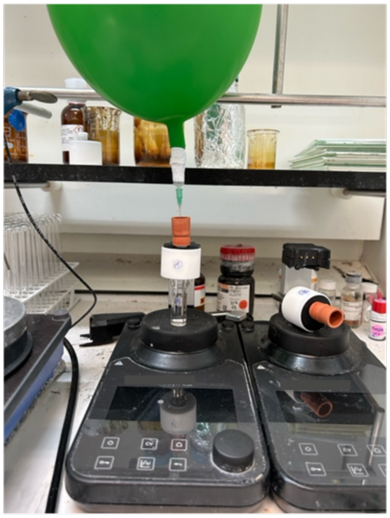

8. After 10 minutes, the cap is screwed fully up until no bubbles are coming from the needle. Then, the needle tip is pulled out of the solvent, and electrolysis is initiated at 10 mA for 3.7 F/mol (3 h) at 1000 rpm.

General procedure E: α-hydrocarbonylation procedure

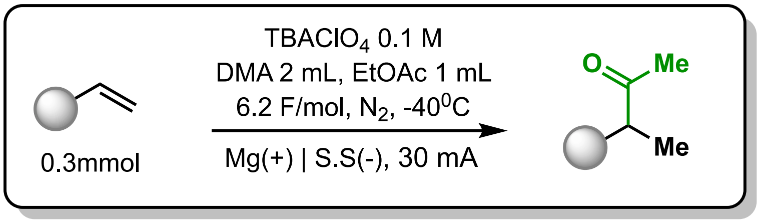

A 5 mL Electrosyn vial was wrapped with Teflon tape on the screw thread, then charged with $TBAClO_4$ (102.9 mg, 0.3 mmol, 2 equiv.), dry DMA (2 mL), and EtOAc (1 mL). The substrate (0.3 mmol, 1 equiv.) was then introduced, and the vial was partially sealed with a septum-equipped Electrosyn cap fitted with a stainless-steel cathode and a magnesium anode (which had been polished beforehand). Using a N_2 balloon and a long needle inserted through the septum, the reaction mixture was purged for 10 minutes. The cap was then sealed completely, ensuring no bubbles appeared at the needle tip. Electrolysis was performed at a constant current of 30 mA with stirring at 1500 rpm, typically for 6.2 F/mol, while maintaining the reaction temperature at approximately -40 °C using a MeCN/dry ice bath. Upon completion, the crude was diluted with 1 M aqueous HCl (30 mL) and extracted with Et_2O (3 × 30 mL). The organic layers were combined, washed with water (20 mL), brine (20 mL), and dried over Na_2SO_4. The organic mixture was concentrated under vacuum, and the product was purified using PTLC (hexane:EtOAc). The N_2 degassing step was critical, as the presence of oxygen significantly affected the reaction outcome by promoting the formation of side products. Likewise, the use of well-dried, degassed solvent was essential; noticeable gas evolution during electrolysis typically indicated incomplete degassing or insufficient solvent drying.

General procedure F: β-hydrocarbonylation procedure

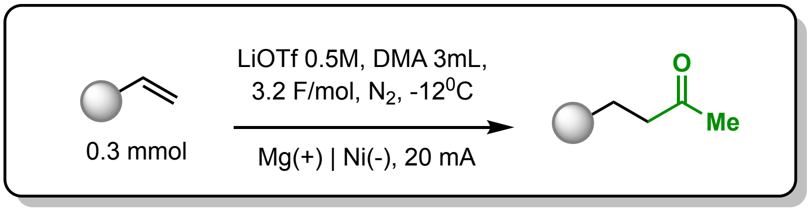

A 5 mL Electrosyn vial was wrapped with Teflon tape on the screw thread, then charged with LiOTf (234 mg, 1.5 mmol, 5 equiv.) and 3 mL of dry DMA. The substrate (0.3 mmol, 1 equiv.) was then added, and the vial was partially sealed with a septum-equipped Electrosyn cap fitted with a nickel cathode and a magnesium anode (pre-polished). Using a N_2 balloon and a long needle inserted through the septum, the reaction mixture was purged for 10 minutes. The cap was then sealed completely, ensuring no bubbles appeared at the needle tip. Electrolysis was performed at a constant current of 10 mA with stirring at 1500 rpm, typically for 3.2 F/mol, while maintaining the reaction temperature at approximately -12 °C using an ethylene glycol/dry ice bath. Upon completion, the crude was diluted with 1 M aqueous HCl (30 mL) and extracted with Et_2O (3 × 30 mL). The organic layers were combined, washed with water (20 mL), brine (20 mL), and dried over Na_2SO_4. The organic mixture was concentrated under vacuum, and the product was purified using PTLC (hexane:EtOAc). The N_2 degassing step was critical, as the presence of oxygen significantly affected the reaction outcome by promoting the formation of side products. Likewise, the use of well-dried, degassed solvent was essential; noticeable gas evolution during electrolysis typically indicated incomplete degassing or insufficient solvent drying.

General procedure G: α-hydroformylation procedure

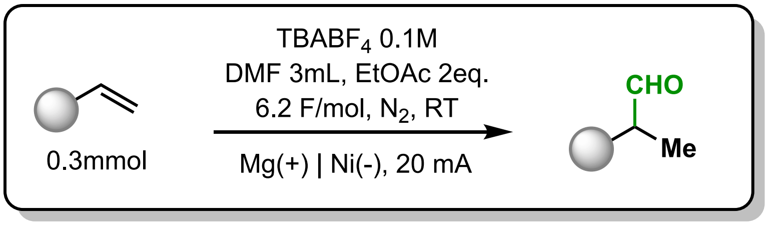

A 5 mL Electrosyn vial was wrapped with Teflon tape on the screw thread, then charged with $TBABF_4$ (0.3 mmol, 98.78 mg), and 3 mL of dry DMF was added by syringe to form a 0.1 M electrolyte solution. Then, EtOAc (0.6 mmol, 58.83 µL, 2 equiv.) was added, along with the substrate (0.3 mmol), and the vial was partially sealed with a septum-equipped Electrosyn cap fitted with a nickel cathode and a magnesium anode (previously polished). Using a N_2 balloon and a long needle inserted through the septum, the reaction mixture was purged for 10 minutes. The cap was then sealed completely, ensuring no bubbles appeared at the needle tip. Electrolysis was performed at a constant current of 20 mA with stirring at 1500 rpm, typically for 6.2 F/mol, at room temperature. Upon completion, the crude was diluted with an aqueous NH_4Cl solution (30 mL) and extracted with Et_2O (3 × 30 mL). The organic layers were combined, washed with water (20 mL), brine (20 mL), and dried over Na_2SO_4. The organic mixture was concentrated under vacuum, and the product was purified using PTLC (hexane:EtOAc). The N_2 degassing step was critical, as the presence of oxygen significantly affected the reaction outcome by promoting the formation of side products.

1.5. Reaction study and optimization campaign

Electrolyte screening

All reactions were carried out on a 0.3 mmol scale. The crude reaction mixture was analyzed by GC/MS using *n*-decane as an internal standard.

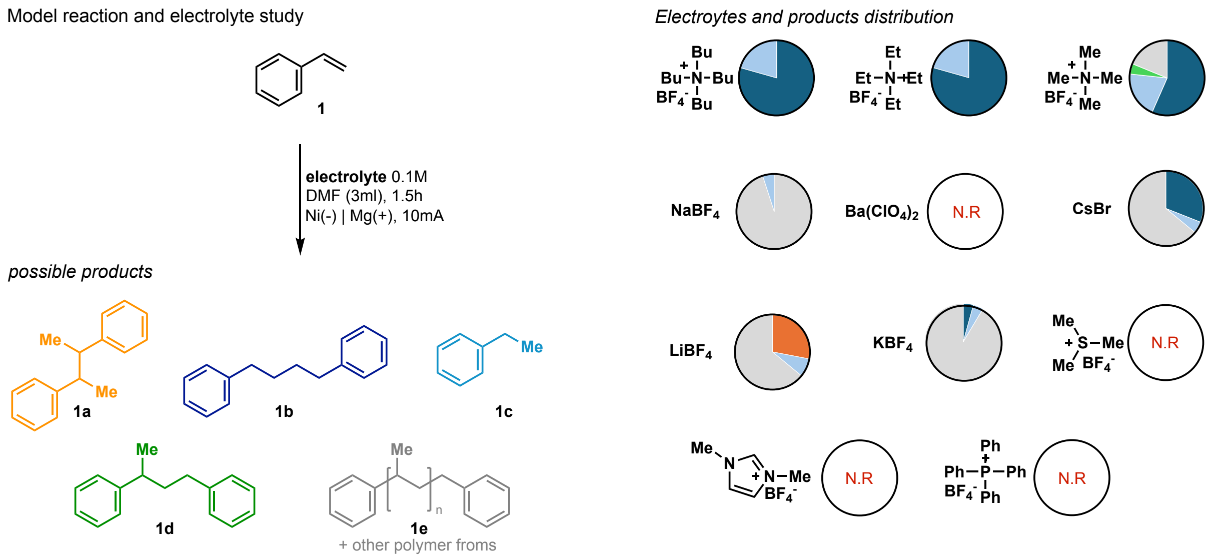

We selected styrene **1** as a simple model substrate. Reactions were conducted under constant current (10 mA) with a sacrificial magnesium anode, a nickel cathode, and DMF as solvent. With these conditions in hand, we surveyed a range of cations and mapped the possible outcomes: (i) branched dimer formation **1a**; (ii) linear dimerization to **1b**; (iii) hydrogenation to ethylbenzene **1c**; (iv) semi-branched coupling **1d**; and (v) reductive polymerization of styrene to **1e** (or related polymeric material), a well-established process under strongly reducing conditions.

With organic ammonium salts, the reaction predominantly afforded the linear dimer **1b**, with reduced styrene **1c** as the main by-product. Notably, tetramethylammonium (Me_4N^+) uniquely promoted substantial polymer formation together with a minor amount of the semi-branched coupling product **1d**, the only condition under which **1d** was detected. In contrast, other organic electrolytes (sulfonium, phosphonium, and imidazolium) gave no reaction, returning starting styrene in full, which we attribute to rapid reduction/decomposition under the electrolysis conditions. Shifting to metal-based electrolytes led to markedly greater polymerization, especially with Na^+ and K^+. Two cation effects stood out: with Cs^+ we observed 33% linear **1b** and 4% reduction (**1c**), with the remainder of the mass balance as polymer; with Li^+, the branched dimer **1a** was obtained in 25% alongside 4% **1c**. Overall, electrolyte identity exerts decisive control over

chemoselectivity, steering the outcome among branched vs linear dimerization and polymerization.

Linear homo-coupling (Procedure A)

All optimization reactions were carried out on a 0.3 mmol scale. The crude reaction mixture was analyzed by GC/MS using *n*-decane as an internal standard.

Evaluation of solvent (Table S1)

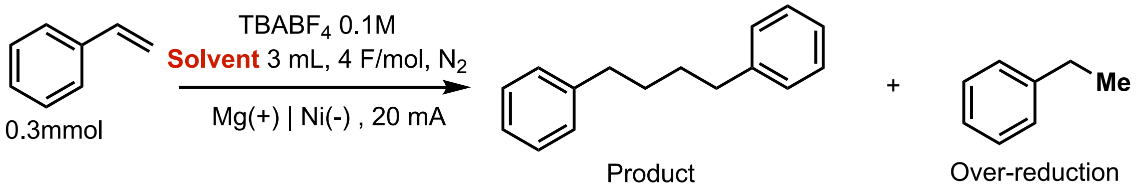

**Solvent**	Product	Over-reduction
NMP	ND	ND
DMSO	ND	ND
Acetone	ND	8.5%
DMA	65%	34%
THF	65%	33%
MeCN	71%	25%
DMF	77%	20%

The impact of the solvent on the reaction performance was tested; thus, a variety of solvents were screened. The reaction worked with MeCN and THF, and no product was formed with dimethylsulfoxide (DMSO) or 1-methylpyrrolidin-2-one (NMP). In the case of acetone as a solvent, a ketone-olefin coupling product 2-methyl-4-phenylbutan-2-ol was obtained. The optimal solvent was found to be an amidic-based solvent. In this instance, dry DMF gave the optimal yield, and *N,N*-dimethylformamide (DMA) gave a slightly lower yield (Table S1).

Evaluation of the cathode (Table S2)

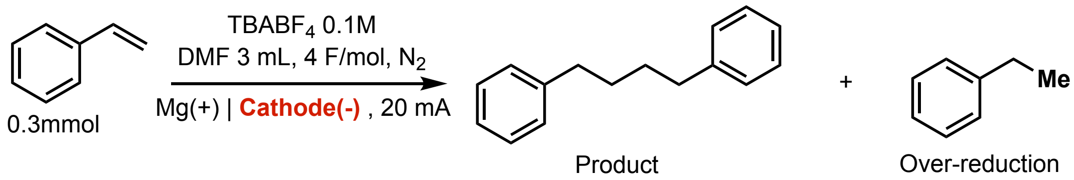

**Cathode**	Product	Over-reduction
Sn	15%	54%
RVC	5%	77%
Mg	25%	44%
Cu	65%	21%
Graphite	35%	54%
Ni	77%	20%
Stainless steel	55%	33%
Ni foam	61%	32%

Different cathode materials were screened to identify the optimal material for the optimized product. Notably, metallic electrodes, such as copper, nickel, and stainless steel, yielded the best results, with nickel being the superior. Porous cathodes, such as RVC (Reticulated vitreous carbon), was problematic because plaque formed during the reaction, leading to inconsistent yields and lower overall yields (Table S2).

Evaluation of the anode (Table S3)

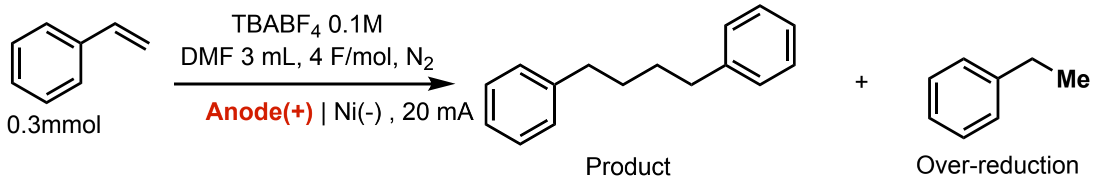

**Anode**	Product	Over-reduction
Al	8%	80%
Ni	60%	38%
Mg	77%	20%
Zn	66%	30%

As with the cathodes, different sacrificial anode materials were checked. The aluminum sacrificial anode directed the reaction almost exclusively towards the over-reduction. Other anodes, such as zinc and nickel, increased the yield of the coupling product, making it the major product, while the optimal result was obtained with magnesium (Table S3).

Evaluation of the counter-ion (Table S4)

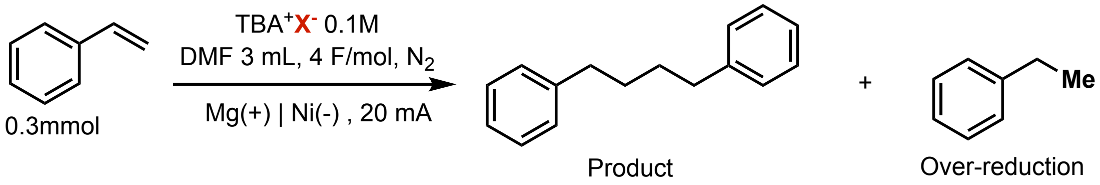

**Counter-ion**	Product	Over-reduction
$TBAClO_4$	31%	60%
$TBAPF_6$	37%	51%
TBABr	43%	38%
$TBABF_4$	77%	20%

Different counteranions of the same cation were screened to evaluate their effect on the reaction outcome. Unlike with other cations, the anions mainly affected the ratio between the coupling and the over-reduction product. ClO_4^-, PF_6^-, and Br^- anions yielded results below 50%, with considerable over-reduction. The BF_4^- salt was found to be the superior candidate, with the highest yield of 77% and consistent results (Table S4).

Linear hetero-coupling (Procedure B)

The crude reaction mixture was analyzed to isolate the heterocoupling product and determine its yield.

Evaluation of regular conditions (5 mL vial, regular electrodes) vs. micro conditions (1 mL vial, micro electrodes) (Table S5)

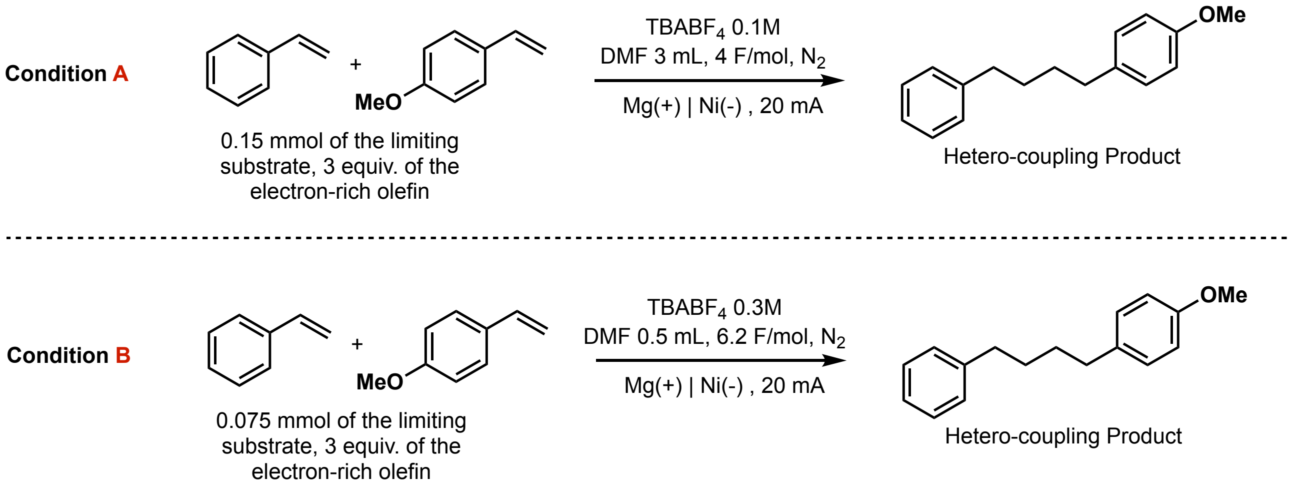

**Condition**	Hetero-coupling product
A	21%
B	54%

While optimizing the hetero-coupling reaction, it was found that increasing the yield of the hetero-product requires 3 equiv of the electron-rich olefin. Additionally, all previously optimized conditions were verified as the most suitable for this reaction. Interestingly, increasing the concentration (0.15 M instead of 0.05 M) and current density (12 mA/cm^2 instead of 5 mA/cm^2) were different, increased the yield of the hetero-coupling product from 21% to 54%.

Branched homo-coupling (Procedure C)

All optimization reactions were carried out on a 0.3 mmol scale. The crude reaction mixture was analyzed by GC/MS using *n*-decane as an internal standard.

Evaluation of solvent (Table S6)

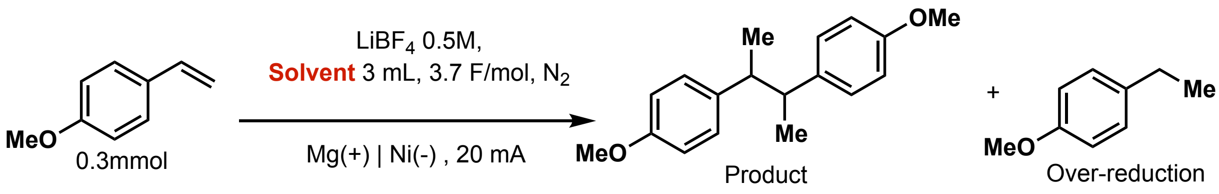

**Solvent**[a]	Product	Over-reduction
DMF	25%	4%
DMA	9%	12%
DMSO	traces	traces
NMP[b]	traces	traces
EtOAc[b]	N.D.	N.D.
MeCN	N.D.	12%
THF	N.D.	N.D.

[a] Commercial $LiBF_4$, containing adventitious moisture (adsorbed from the atmosphere), was used

[b] Lower reaction time due to fast reaching of 30 V (poor conductivity).

At the first stage of optimization, we examined the effect of solvent nature on the yield of the methoxystyrene hydrodimerization product (Table S6). Among all the solvents tested, only DMF and DMA afforded the desired product. For DMSO and NMP, only trace amounts of the product were obtained, whereas with other solvents, only side products were obtained.

Evaluation of proton source (Table S7)

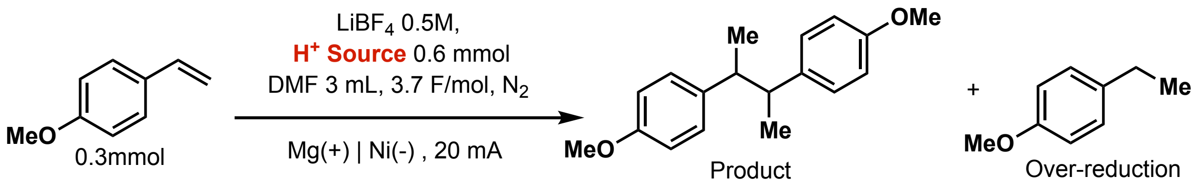

**Proton source**[a]	Product	Over-reduction
No proton source	25%	4%
HFIP	6%	69%
H_2O	23%	4%
i-BuOH	22%	18%
Et_3HBF_4	17%	5%
$TBABF_4$	13%	6%
EtSH	37%	16%
PhSH	24%	26%
Urea	23%	20%
DMU	6%	3%
DPrU	5%	4%
Urea (3 equiv)	42%	10%
Urea (3 equiv) + HMPA	26%	7%
**$BuNH_2$**	**61%**	**15%**
Bu_2NH	56%	12%
CH_3CN	30%	6%
PhOH	0%	50%
**$C_6H_{13}NH_2$**	**64%**	**16%**
**$C_8H_{17}NH_2$**	**64%**	**13%**
$^{i}Pr_2NH$ (3 equiv)	44%	14%
$(TMS)_2NH$ (3 equiv)	N.D.	N.D.
Cyclohexylamine	44%	11%
Piperidine	35%	7%

Ethylenediamine	58%	11%
Acetamide	38%	18%
Cyclohexylacetylene	23%	6%
EtOAc	23%	5%
Cyclohexadyene	13%	29%

[a] Commercial $LiBF_4$, containing adventitious moisture (adsorbed from the atmosphere), was used

Conducting the reaction without an external proton source yielded only 25% (Table S7, Entry 1). During the reaction, we observed the formation of transparent precipitate, which we identified as the polymerization product. To suppress this pathway, we tested several proton sources with pK_a ranging from 6 to 35. In addition, several H atom sources were evaluated (Table S7, Entries 2-27). Best yields (61-64%) were achieved using linear aliphatic primary amines ($BuNH_2$, hexyl-NH_2, and octyl-NH_2). When secondary amines (piperidine, dibutylamine, diisopropylamine), ethylenediamine, and cyclohexylamine were used, moderate yields (35–56%) were also obtained; however, they were still lower than those observed for primary linear amines, regardless of the number of equivalents added. Other proton sources yielded significantly lower amounts of the hydrodimerization product and led primarily to over-reduction reactions or polymerization.

Evaluation of the additives (Table S8)

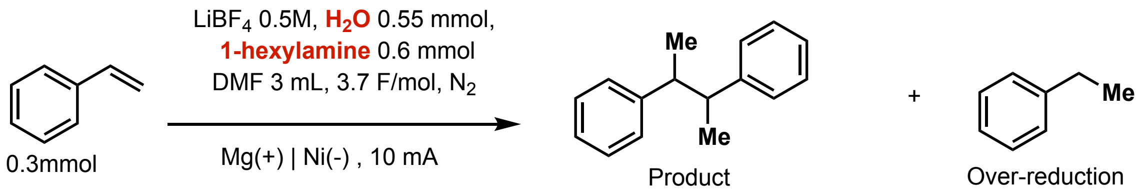

**Additive**	Product	Over-reduction
No H_2O	34%	21%
No 1-hexylamine	16%	1%
No both	12%	1%
**With both**	**72%**	**14%**

Even with the most suitable proton source at hand, we noticed inconsistent reaction yields. Given that, these issues mostly occur during the humid season, we hypothesize that they may be related to water concentration within our system. Although we did not intentionally add water to the reaction, $LiBF_4$ is highly hygroscopic and can affect its outcome. To test this, we dried $LiBF_4$ at 155 °C for 1 h before the reaction and used the resulting salt, avoiding exposure to the atmosphere (see "General graphical guide of the procedure C and D"). Nevertheless, the reaction yield remained poor, affording only 34% of the product, mainly due to side-product formation (Table S8, Entry 1). Removing hexyl-NH_2 (while adding a controlled amount of water) or running the reaction without both gave only a small amount of product (16% and 12%, respectively). Only when both hexylamine and a controlled amount of water were added to the reaction (with dried $LiBF_4$ used in all cases) were we able to achieve high and consistent coupling yields (72%).

Evaluation of the current (Table S9)

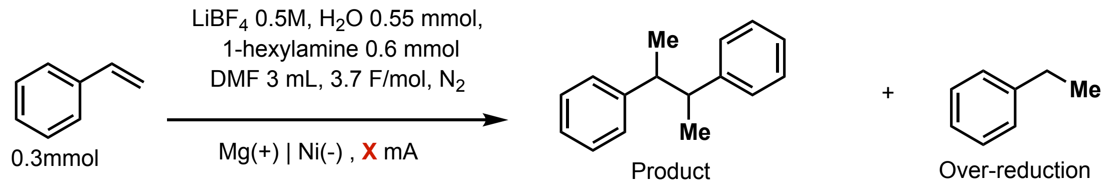

**Current**	Product	Over-reduction
5 mA	72%	14%
**10 mA**	**72%**	**14%**
20 mA	67%	16%

During the optimization of the current, we found that this parameter had almost no effect on the reaction outcome (provided that the total charge passed was the same). In all cases, the yield remained between 67% and 72%.

Evaluation of the cathode (Table S10)

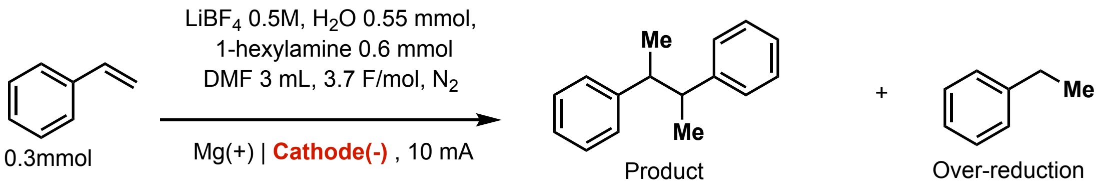

**Cathode**	Product	Over-reduction
Sn	16%	24%
Graphite	67%	21%
**Ni**	**72%**	**14%**
Stainless steel	68%	10%

During screening of cathode materials, we found that Ni cathode results in the highest yield of the coupling product, followed by stainless steel (68%), graphite (67%), and tin (16%; Table S10).

Evaluation of the anode (Table S11)

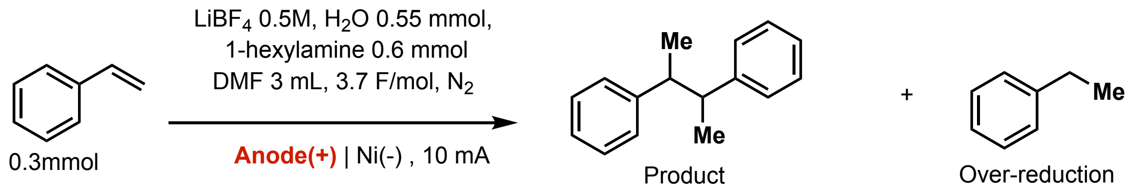

**Anode**	Product	Over-reduction
Zn	12%	1%
Al	N.D.	N.D.
**Mg**	**72%**	**14%**

During screening of anode materials, only the magnesium anode was found to be suitable for this reaction; zinc yielded 12%, and no product was observed with the aluminum anode (Table S11).

Evaluation of the counter-ion (Table S12)

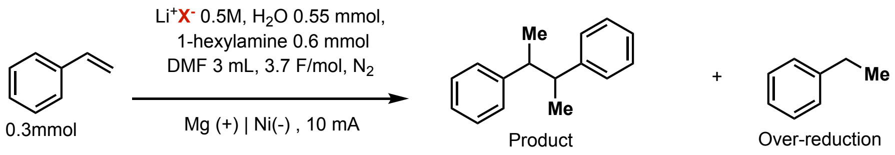

**Counter-ion**	Product	Over-reduction
$LiBF_4$	72%	14%
$LiClO_4$	71%	21%
LiBr	71%	21%
LiOTf	70%	19

Other counter-ions besides BF_4^- were tested, such as Br^-, OTf^- (triflate), and ClO_4- (all salts were dried before the reaction). All gave almost identical yields of around 70% (Table S12), indicating that this parameter does not affect the reaction outcome.

General procedure E: α-hydrocarbonylation procedure

All optimization reactions were carried out on a 0.3 mmol scale. The crude reaction mixture was analyzed by ^{1}H NMR, using nitromethane as an internal standard, unless otherwise stated.

Evaluation of temperature (Table S13)

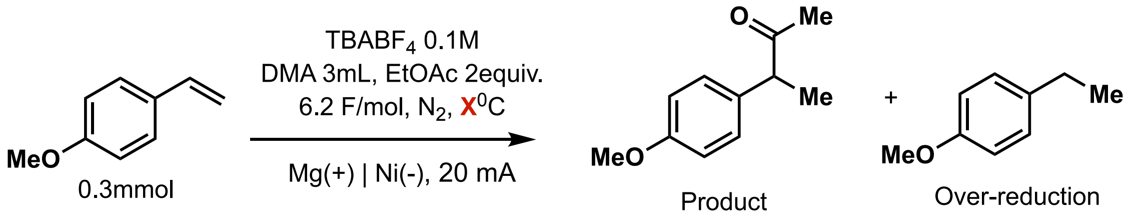

**Temperature**	Product	Over-reduction
RT	10%	76%
-20 °C	22%	60%

In the initial stages of the optimization, the impact of temperature was evaluated with 2 equiv. of ethyl acetate (EtOAc). It was observed that cooling the reaction decreased over-reduction and moderately increased the yield of the hydrocarbonylation product. Lowering the temperature below -20 °C during this stage was limited by the DMA solvent's melting point (Table S13).

Evaluation of EtOAc equiv (Table S14)

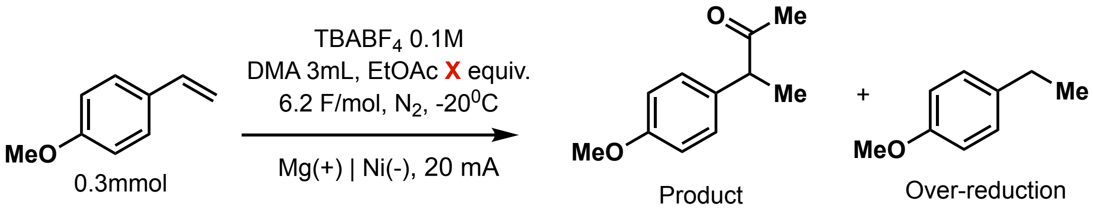

**EtOAc equiv**	Product	Over-reduction
2	22%	60%
4	10%*	60%*
8	22%	70%
16	14%*	70%*
32	33%	60%
DMA:EtOAc 2:1 (3 mL total)	22%*	58%*

*GC/MS yield, using *n*-decane as an internal standard

The effect of EtOAc equivalents on hydrocarbonylation formation was analyzed, as it is a crucial additive that dictates the product formation. A clear correlation between the equiv and the product yield was not observed. The optimization continued with the DMA:EtOAc 2:1 ratio, which yielded results comparable to those at 2 equiv conditions and allowed for reducing the temperature below -20 °C (Table S14).

Evaluation of the constant current. (Table S15)

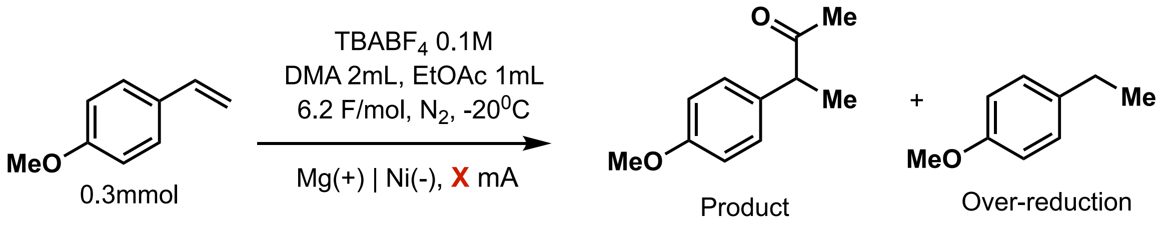

**Current (mA)**	Product	Over-reduction
10	10%*	60%*
20	22%*	58%*
30	25%*	49%*

*GC/MS yield, using *n*-decane as an internal standard

The effect of the electrical current was examined. Lower current (10 mA, down from 20 mA) decreased the yield to 10%. A higher current, 30 mA, slightly increased the product yield to 25%, making it the optimal current for the reaction (Table S15).

Further evaluation of temperature (Table S16)

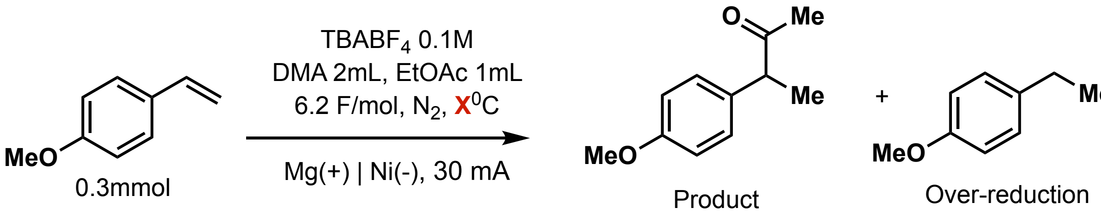

**Temperature**	Product	Over-reduction
RT	12%	80%
-20 °C	25%*	49%*
-40 °C	41%	45%

*GC/MS yield, using *n*-decane as an internal standard

Encouraged by the results shown in Table 12, the reaction with a DMA:EtOAc 2:1 solvent ratio (3 mL total) was cooled to -40 °C. As the temperature decreased, the product yield increased, rising from 12% at RT to 41% at -40 °C. Based on these results, the following reaction optimization kept the solvent ratio and temperature constant (Table S16).

Evaluation of counter-ion (Table S17)

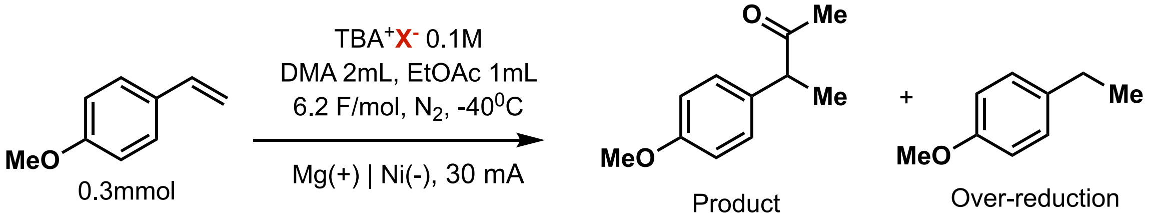

**Counter-ion**	Product	Over-reduction
$TBABF_4$	41%	45%
TBAI	35%	36%
$TBAPF_6$	29%	35%
TBASCN	34%	39%

$TBAClO_4$	50%	26%
TBAOTf	28%	44%

As in other reactions, counteranions were screened. Here, $TBAClO_4$ improved the yield compared to the original electrolyte, $TBABF_4$, increasing it from 41% to 50%. The triflate salt, along with TBAI and TBASCN, decreased the yield. Hence, the perchlorate salt was chosen as the standard one for this reaction (Table S17).

Evaluation of the cathode (Table S18)

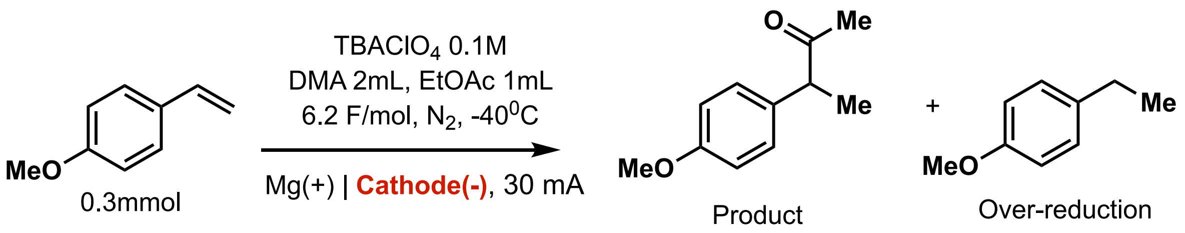

**Cathode**	Product	Over-reduction
Cu	47%	45%
Ni	50%	26%
Sn	62%	33%
Stainless steel	66%	29%

Finally, a range of cathodes was screened. Stainless steel was found to be optimal, yielding the best 66% yield, followed by Sn, Ni, and Cu (Table S18).

Compound 1a

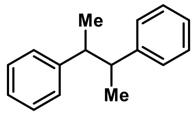

Following the **general procedure C** on a 0.3 mmol scale with **1**. After passing 3.7 F/mol of charge (3 h) and standard workup, the product was purified by PTLC (SiO_2, 100% hexane) to afford 22.7 mg (72% yield) of **1a** as a white solid (mixture of 2 diastereomers 1:1, determined by GC-MS). Note: during purification, only the purest fractions were collected, resulting in a change in the diastereomer ratio to 7:10 in the NMR spectra.

^{1}H NMR (300 MHz, CDCl3-*d*) δ 7.45 – 6.95 (m, 10H), 3.05 – 2.92 (m, 1H), 2.90 – 2.77 (m, 1H), 1.32 (s, 3H), 1.06 (d, 3H).

^{13}C NMR (75 MHz, $CDCl_3$) δ 146.5, 145.9, 128.3, 127.8, 127.8, 127.6, 126.1, 125.7, 47.3, 46.5, 21.1, 18.0.

Physical State: White solid

GC/MS(EI): 105.1 (100%), 104.1 (23.75%), 77 (12.95%)

TLC: R_f=0.5 (100% hexane)

The spectroscopic data are consistent with those reported in the literature.[16]

Compound 1b

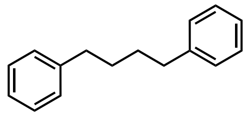

Following the **general procedure A** on a 0.3 mmol scale with **1**. After passing 6.2 F/mol of charge (2.5 h) and standard workup, the product was purified by PTLC (SiO_2, 100% hexane) to afford 22 mg (70% yield) of **1b** as a pale yellow oil.

^{1}H NMR (300 MHz, CDCl3): δ 7.37 – 7.14 (m, 10H), 2.73 – 2.61 (m, 4H), 1.80 – 1.61 (m, 4H)
^{13}C NMR (126 MHz, $CDCl_3$): δ 142.6, 128.4, 128.3, 125.7, 35.8, 31.1.
Physical State: Pale yellow oil
GC/MS(EI): 104 (13.32%), 210 (71.46%), 91 (100%)
TLC: R_f=0.6 (100% hexane)
The spectroscopic data are consistent with those reported in the literature.[17]

Compound 4a

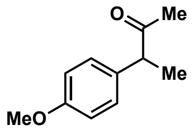

Following the **general procedure E** on a 0.3 mmol scale with **4**. After passing 6.2 F/mol of charge (2.5 h) and standard workup, the product was purified by PTLC (SiO_2, hexane:EtOAc 6:1) to afford 24 mg (45% yield) of **4a** as a yellow oil.

^{1}H NMR (400 MHz, $CDCl_3$): δ 7.15 (d, J = 8.7 Hz, 2H), 6.89 (d, J = 8.8 Hz, 2H), 3.82 (s, 3H), 3.71 (q, J = 7.1 Hz, 1H), 2.06 (s, 3H), 1.38 (d, J = 7.0 Hz, 3H)
^{13}C NMR (151 MHz, $CDCl_3$): δ 209.4, 158.8, 132.7, 128.9, 114.4, 55.4, 52.9, 28.3, 17.4.
GC/MS(EI): 178 (8.7%), 105 (18.99%), 135 (100%)
Physical State: yellow oil
TLC: R_f= 0.6 (hexane:EtOAc 6:1)
The spectroscopic data are consistent with those reported in the literature.[18]

Compound 4b

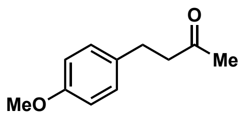

Following the **general procedure F** on a 0.3 mmol scale with **4**. After passing 3.1 F/mol of charge (2.5 h) and standard workup, the product was purified by PTLC (SiO_2, hexane:EtOAc 5:1) to afford 16.6 mg (31% yield) of **4b** as a yellow oil.

^{1}H NMR (400 MHz, $CDCl_3$): δ 7.12 (d, J = 8.7 Hz, 2H), 6.84 (d, J = 8.7 Hz, 2H), 3.80 (s, 3H), 2.86 (t, J = 7.1 Hz, 2H), 2.78 – 2.72 (m, 2H), 2.15 (s, 3H).
^{13}C NMR (201 MHz, $CDCl_3$): δ 208.5, 158.2, 133.3, 129.5, 114.2, 55.6, 45.8, 30.4, 30.0.
GC/MS(EI): 43 (19.48%), 178 (38.5%), 121 (100%)
Physical State: yellow oil
TLC: R_f= 0.4 (hexane:EtOAc 5:1)
The spectroscopic data are consistent with those reported in the literature.[19]

Compound 4c

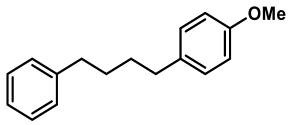

Following the **general procedure B** on a 0.075 mmol scale of **1** and 3 equiv of **4**. After passing 3.1 F/mol of charge (1.25 h) and standard workup, the product was purified by PTLC (SiO_2, hexane:EtOAc 19:1) to afford 9.8 mg (54%) of **4c** as a pale-yellow oil.

^{1}H NMR (300 MHz, $CDCl_3$): δ 7.39 – 6.76 (m, 9H), 3.81 (s, 3H), 2.77 – 2.49 (m, 4H), 1.68 (dd, J = 9.1, 5.5 Hz, 4H).

^{13}C NMR (151 MHz, $CDCl_3$): δ 157.7, 142.8, 134.8, 129.4, 128.6, 128.4, 125.8, 113.8, 55.4, 36.0, 35.0, 31.5, 31.2.

Physical State: pale-yellow oil

HRMS (ASAP-TOF): calc'd for $C_{17}H_{21}O$ $[M+H]^+$: 241.1587; found: 241.1602

TLC: R_f=0.5 (hexane:EtOAc 19:1)

Compound 4e

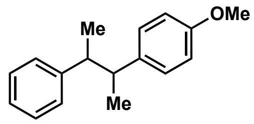

Following the **general procedure D** on a 0.15 mmol scale of **1** and 3 equiv of **4**. After passing 3.7 F/mol of charge (3 h) and standard workup, the product was purified by PTLC (SiO_2, hexane:EtOAc 19:1) to afford 17.6 mg (47% yield) of **4e** as a white solid (mixture of 2 diastereomers 1:1, determined by GC-MS).

^{1}H NMR (600 MHz, $CDCl_3$): δ 7.33 (t, *J* = 7.6 Hz, 2H), 7.26 – 7.20 (m, 2H), 7.15 (d, *J* = 8.6 Hz, 3H), 6.88 (d, *J* = 8.6 Hz, 2H), 3.83 (s, 3H), 2.82 – 2.73 (m, 2H), 1.04 (d, *J* = 6.6 Hz, 3H), 1.02 (d, *J* = 6.5 Hz, 3H).

^{13}C NMR (151 MHz, $CDCl_3$): δ 158.0, 146.7, 138.7, 128.6, 128.4, 127.7, 126.1, 113.8, 55.4, 47.6, 46.5, 21.3, 21.1.

Physical State: white solid

GC/MS(EI): 240 (1.74%), 105 (10.77%), 135 (100%)

TLC: R_f=0.5 (hexane:EtOAc 19:1)

The spectroscopic data are consistent with those reported in the literature.[20]

Compound 5a

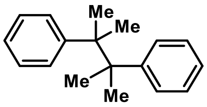

Following the **general procedure C** on a 0.3 mmol scale with **5**. After passing 3.7 F/mol of charge (3 h) and standard workup, the product was purified by PTLC (SiO_2, 100% hexane) to afford 24.6 mg (68% yield) of **5a** as a white solid.

^{1}H NMR (300 MHz, $CDCl_3$): δ 7.23 – 7.20 (m, 1H), 7.13 – 7.08 (m, 1H), 1.36 (s, 3H).
^{13}C NMR (151 MHz, $CDCl_3$): δ 146.8, 128.7, 126.7, 125.5, 43.7, 25.2
Physical State: White solid
GC/MS(EI): 119.1(100%), 91(36.89%), 120(10.32%)
TLC: R_f= 0.9 (100% hexane)
The spectroscopic data are consistent with those reported in the literature.[21]

Compound 5b

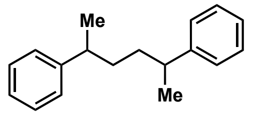

Following the **general procedure A** on a 0.3 mmol scale with **5**. After passing 6.2 F/mol of charge (2.5 h) and standard workup, the product was purified by PTLC (SiO_2, 100% hexane) to afford 14.3 mg (40% yield) of **5b** as a yellow oil (mixture of 2 diastereomers 1:1, determined by GC-MS).

^{1}H NMR (300 MHz, $CDCl_3$): δ 7.33 – 7.24 (m, 5H), 7.22 – 7.10 (m, 5H), 2.66 (q, J = 7.1 Hz, 2H), 1.54 – 1.40 (m, 4H), 1.21 (d, J = 7.0 Hz, 6H).

^{13}C NMR (151 MHz, $CDCl_3$): δ 147.8, 128.4, 127.1, 125.9, 40.2, 36.6, 36.3, 22.7, 22.5.

Physical State: Yellow oil

GC/MS(EI): 106 (12.03%), 238 (23.98%), 105 (100%)

TLC: R_f=0.6 (100% hexane)

The spectroscopic data are consistent with those reported in the literature.[21]

Compound 6a

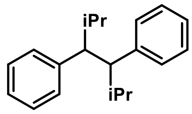

Following the **general procedure C** on a 0.3 mmol scale with **6**. After passing 3.7 F/mol of charge (3 h) and standard workup, the product was purified by PTLC (SiO_2, 100% hexane) to afford 15.8 mg (40% yield) of **6a** as a white solid (mixture of 2 diastereomers 4:5, determined by GC-MS and ^{1}H NMR).

^{1}H NMR (400 MHz, $CDCl_3$) δ 7.42 –6.81 (m, 10H), 3.13 (m, 1H), 3.03 (m, 1H), 2.18 – 2.06 (m, 1H), 1.77 – 1.64 (m, 1H), 1.00 (d, *J* = 6.7 Hz, 3H), 0.79 (d, *J* = 6.7 Hz, 3H), 0.69 (d, *J* = 6.9 Hz, 3H), 0.62 (d, *J* = 6.9 Hz, 3H).

^{13}C NMR (151 MHz, $CDCl_3$) δ 140.5, 140.3, 127.6, 126.9, 126.0, 125.5, 52.8, 52.7, 28.6, 27.9, 22.5, 22.4, 18.4, 15.6.

Physical State: White solid

GC-MS (EI): 105 (21.67%), 133 (87.11%), 91 (100%)

TLC: R_f= 0.65 (100% hexane)

Compound 6b

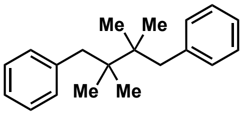

Following the **general procedure A** on a 0.3 mmol scale with **6**. After passing 6.2 F/mol of charge (2.5 h) and standard workup, the product was purified by PTLC (SiO_2, 100% hexane) to afford 20.1 mg (50% yield) of **6b** as a white solid.

^{1}H NMR (400 MHz, $CDCl_3$): δ 7.37 – 7.17 (m, 10H), 2.74 (s, 4H), 0.88 (s, 12H).
^{13}C NMR (101 MHz, $CDCl_3$): δ 140.1, 131.3, 127.5, 125.7, 42.4, 39.5, 21.5.
Physical State: White solid
GC/MS(EI): 133 (11.98%), 105 (28.45%), 91 (100%)
TLC: R_f=0.6 (100% hexane)

Compound 7a

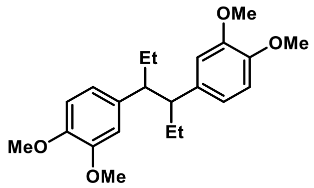

Following the **general procedure C** on a 0.3 mmol scale with **7**. After passing 3.7 F/mol of charge (3 h) and standard workup, the product was purified by PTLC (SiO_2, hexane:EtOAc 3:1) to afford 23.0 mg (43% yield) of **7a** as a yellow solid (mixture of 2 diastereomers 1:1, determined by GC-MS and ^{1}H NMR).

^{1}H NMR (500 MHz, CDCl$_3$) δ 6.84 (d, *J* = 8.1 Hz, 1H), 6.73 (dd, *J* = 8.1, 1.8 Hz, 1H), 6.68 (d, *J* = 8.2 Hz, 1H), 6.66 (d, *J* = 1.7 Hz, 1H), 6.46 (dd, *J* = 8.2, 1.9 Hz, 1H), 6.29 (m, 1H), 2.69 – 2.63 (m, 1H), 2.51 – 2.45 (m, 1H), 1.88 – 1.78 (m, 1H), 1.61-1.51 (m, 1H), 1.50 – 1.41 (m, 1H), 1.38 – 1.24 (m, 1H)., 0.79 (t, *J* = 7.3 Hz, 3H), 0.58 (t, *J* = 7.3 Hz, 3H).

^{13}C NMR (151 MHz, CDCl$_3$) δ 148.7, 147.8, 147.2, 146.9, 137.1, 135.2, 121.6, 112.3, 110.8, 110.1, 55.9, 55.8, 55.8, 55.7, 54.1, 52.7, 27.3, 26.4, 12.5, 12.4.

Physical State: Yellow solid

HRMS: calc'd for $C_{22}H_{30}O_4Na$ [M+Na]$^+$: 381.2045; found: 381.2042

TLC: R_f= 0.5 (hexane:EtOAc 3:1)

Compound 7b

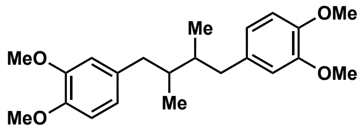

Following the **general procedure A**, but with DMA instead of DMF, on a 0.3 mmol scale with **7**. After passing 3.7 F/mol of charge (1.5 h) and standard workup, the product was purified by PTLC (SiO_2, hexane:EtOAc 7:1) to afford 35.1 mg (65% yield) of **7b** as a white solid (mixture of 2 diastereomers 1:3, determined by ^{1}H NMR and GC-MS).

^{1}H NMR (300 MHz, $CDCl_3$): δ 6.86 – 6.69 (m, 3H), 6.69 – 6.57 (m, 3H), 3.88 (s, 6H), 3.84 (s, 6H), 2.85 – 2.51 (m, 2H), 2.48 – 2.26 (m, 2H), 1.78 (q, *J* = 6.5 Hz, 2H), 0.86 (d, *J* = 6.7 Hz, 6H).
^{13}C NMR (75 MHz, $CDCl_3$): δ 148.6, 147.0, 134.4, 134.2, 112.2, 112.1, 111.0, 110.9, 55.9, 55.8, 55.7, 41.0, 39.1, 38.8, 37.6, 16.2, 13.3
Physical State: White solid
HRMS (ESI-TOF): calc'd for $C_{22}H_{30}O_4Na$ $[M+Na]^+$: 381.2086; found: 381.2028
TLC: R_f=0.4 (hexane:EtOAc 7:1)

Compound 8a

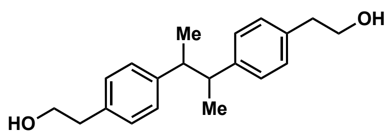

Following the **general procedure C** on a 0.3 mmol scale with **8**. After passing 3.7 F/mol of charge (3 h) and standard workup, the product was purified by PTLC (SiO_2, hexane:EtOAc 1:1) to afford 22.4 mg (50% yield) of **8a** as a white solid (mixture of two diastereomers 1:1, determined by crude ^{1}H NMR). To obtain clean NMR spectra, the compound was further purified by preparative HPLC (MeCN/Water). Only one diastereomer was isolated in pure form.

^{1}H NMR (300 MHz, $CDCl_3$): δ 7.28-7.12 (m, 4H), δ 7.04 (d, J = 8.4 Hz, 2H), 6.98 (d, J = 8.2 Hz, 2H), 3.89 (t, J = 5.8 Hz, 2H), 3.82 (t, J = 5.8 Hz, 2H), 2.97 – 2.90 (m, 1H), 2.92 (t, J = 6.5 Hz, 2H), 2.87 – 2.81 (m, 1H), 2.80 (t, J = 6.5 Hz, 2H), 1.29 (d, J = 6.9 Hz, 3H), 1.03 (d, J = 6.9 Hz, 3H).
^{13}C NMR (151 MHz, $CDCl_3$): δ 144.85, 144.33, 136.12, 135.74, 129.08, 128.61, 128.18, 127.97, 63.88, 63.74, 47.02, 46.29, 38.95, 38.81, 21.29, 18.08.
Physical State: white solid
HRMS (ESI-TOF): calc'd for $C_{20}H_{26}O_2Na$ $[M+Na]^+$: 321.1825; found: 321.1827
TLC: R_f=0.4 (hexane:EtOAc 1:1)

Compound 8b

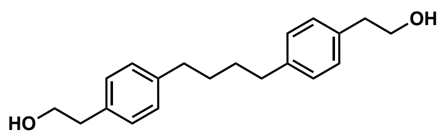

Following the **general procedure A** on a 0.3 mmol scale with **8**. After passing 6.2 F/mol of charge (2.5 h) and standard workup, the product was purified by PTLC (SiO_2, DCM:EtOAc 1:1) to afford 9 mg (20%) of **8b** as a white powder.

^{1}H NMR (800 MHz, $CDCl_3$): δ 7.15 – 7.10 (m, 8H), 3.85 (t, J = 6.2 Hz, 4H), 2.84 (t, J = 6.5 Hz, 4H), 2.63 – 2.59 (m, 4H), 1.67 – 1.62 (m, 4H).
^{13}C NMR (201 MHz, $CDCl_3$): δ 141.1, 135.9, 129.2, 129.0, 64.1, 39.1, 35.7, 31.4.
Physical State: White solid
HRMS (ESI-TOF): calc'd for $C_{20}H_{26}O_2Na$ $[M+Na]^+$: 321.1823; found: 321.1826.
TLC: R_f=0.6 (DCM:EtOAc 1:1)

Compound 9a

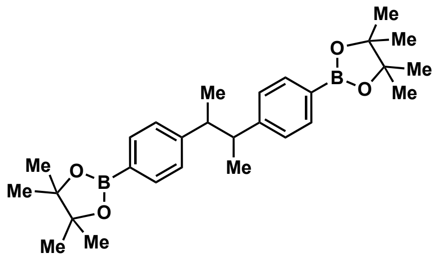

Following the **general procedure C** on a 0.3 mmol scale with **9**. After passing 3.7 F/mol of charge (3 h) and standard workup, the product was purified by PTLC (SiO_2, hexane:EtOAc 9:1) to afford **9a** (62% yield, determined by ^{1}H NMR) as a white solid (mixture of 2 diastereomers 1:1, determined by ^{1}H NMR).

^{1}H NMR (800 MHz, $CDCl_3$) δ 7.77 (d, J = 7.8 Hz, 2H), 7.60 (d, J = 7.8 Hz, 2H), 7.24 (d, J = 7.9 Hz, 2H), 7.03 (d, J = 7.9 Hz, 2H), 3.01 – 2.95 (m, 1H), 2.85 – 2.80 (m, 1H), 1.35 (s, 12H), 1.32 (s, 12H), 1.26 (d, J = 6.6 Hz, 3H), 1.00 (d, J = 6.5 Hz, 3H).
^{13}C NMR (201 MHz, $CDCl_3$): δ 150.2, 149.5, 135.2, 134.7, 127.6, 127.4, 84.0, 83.9, 47.6, 46.7, 25.2, 21.3, 18.4.
^{11}B NMR (96 MHz, $CDCl_3$): δ 32.4
Physical State: white solid
HRMS (ESI-TOF): calc'd for $C_{28}H_{41}O_4B_2$ $[M+H]^+$: 463.3185; found: 463.3189
TLC: R_f=0.5 (hexane:EtOAc 9:1)

Compound 9b

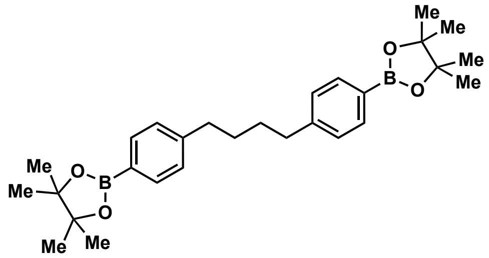

Following the **general procedure A** on a 0.3 mmol scale with **9**. After passing 6.2 F/mol of charge (2.5 h) and standard workup, the product was purified by PTLC (SiO_2, hexane:EtOAc 9:1) to afford **9b (**67% yield, determined by ^{1}H NMR).

^{1}H NMR (800 MHz, $CDCl_3$) δ 7.71 (d, *J* = 8.0 Hz, 4H), 7.17 (d, *J* = 7.9 Hz, 4H), 2.65 – 2.61 (m, 4H), 1.67 – 1.62 (m, 4H), 1.33 (s, 24H).

^{13}C NMR (151 MHz, $CDCl_3$) δ 146.1, 134.9, 128.05, 83.7, 36.1, 31.0, 24.9

^{11}B NMR (96 MHz, $CDCl_3$) δ 31.8

Physical State: White solid

HRMS (ESI-TOF): calc'd for $C_{28}H_{40}O_4B_2Na$ $[M+Na]^+$: 485.3005; found: 485.3010

TLC: R_f=0.4 (hexane:EtOAc 9:1)

Compound 10a

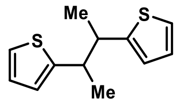

Following the **general procedure C** on a 0.3 mmol scale with **10**. After passing 3.7 F/mol of charge (3 h) and standard workup, the product was purified by PTLC (SiO_2, 100% hexane) to afford **10a** (25% yield) as a yellow oil (a mixture of 2 diastereomers, 1:1, determined by ^{1}H NMR). NMR determined yield with an internal standard (nitromethane).

^{1}H NMR (400 MHz, CDCl$_3$) δ 7.17 (dd, *J* = 5.0, 1.0 Hz, 1H), 7.13 (dd, *J* = 5.1, 1.1 Hz, 1H), 6.95 (dd, *J* = 5.1, 3.5 Hz, 1H), 6.91 (dd, *J* = 5.1, 3.5 Hz, 1H), 6.80 (dd, *J* = 3.4, 0.8 Hz, 1H), 6.74 (d, *J* = 3.4 Hz, 1H), 3.38 – 3.32 (m, 1H), 3.22 – 3.16 (m, 1H), 1.35 (d, *J* = 6.8 Hz, 3H), 1.25 (dd, *J* = 4.8, 1.8 Hz, 3H).

13C NMR (151 MHz, CDCl$_3$) δ 149.4, 149.3, 126.4, 126.3, 123.9, 123.8, 122.9, 122.8, 43.4, 42.4, 21.2, 17.8.

Physical State: Yellow oil

GC/MS(EI): 222 (2.91%), 77 (7.49%), 11 (100%)

TLC: R_f=0.6 (100% hexane)

Compound 10b

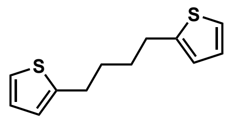

Following the **general procedure A** on a 0.3 mmol scale with **10**. After passing 6.2 F/mol of charge (2.5 h) and standard workup, the product was purified by PTLC (SiO_2, 100% hexane) to afford 12.65 mg (38% yield) of **10b** as a yellow oil.

^{1}H NMR (500 MHz, CDCl3): δ 7.13 (dd, J = 5.1, 1.2 Hz, 2H), 6.93 (dd, J = 5.1, 3.4 Hz, 2H), 6.80 (t, J = 3.3, 1.1 Hz, 2H), 2.92 – 2.84 (m, 4H), 1.83 – 1.73 (m, 4H).
^{13}C NMR (151 MHz, $CDCl_3$): δ 145.4, 126.8, 124.2, 123.0, 31.3, 29.8.
Physical State: yellow oil
GC/MS(EI): 124 (20.38%), 222 (45.3%), 97 (100%)
TLC: R_f=0.4 (100% hexane)
The spectroscopic data are consistent with those reported in the literature.[20]

Compound 11a

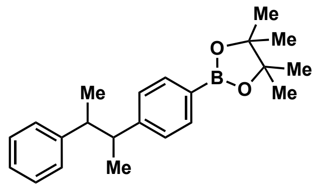

Following the **general procedure D** on a 0.15 mmol scale of **1** and 3 equiv of **9**. After passing 3.7 F/mol of charge (3 h) and standard workup, the product was purified by PTLC (SiO_2, hexane:EtOAc 19:1) to afford 18.2 mg (36% yield) of **11a** as a white solid (mixture of 2 diastereomers 1:1, determined by GC-MS).

^{1}H NMR (600 MHz, $CDCl_3$): δ 7.79 (d, J = 7.9 Hz, 1H), 7.63 (d, J = 8.0 Hz, 1H), 7.33 (t, J = 7.6 Hz, 1H), 7.27 – 7.01 (m, 6H), 3.01 – 2.94 (m, 1H), 2.89 – 2.78 (m, 1H), 1.37 (s, 6H), 1.34 (s, 6H), 1.28 (d, J = 9.2 Hz, 3H), 1.03 (d, J = 6.3 Hz, 3H).

^{13}C NMR (151 MHz, $CDCl_3$): δ 150.0, 149.4, 146.4, 145.7, 134.9, 134.4, 128.3, 127.9, 127.8, 127.6, 127.3, 127.1, 126.1, 125.7, 83.7, 83.6, 47.5, 47.1, 46.6, 46.2, 24.9, 24.9, 24.86, 21.05, 21.0, 18.1, 18.0.

^{11}B NMR (96 MHz, $CDCl_3$): δ 32.54 (br).

Physical State: White solid

HRMS (ASAP-TOF): calc'd for $C_{22}H_{30}BO_2$ $[M+H]^+$: 337.2333; found: 337.2416

TLC: R_f=0.4 (Hexane:EtOAc, 19:1)

Compound 11b

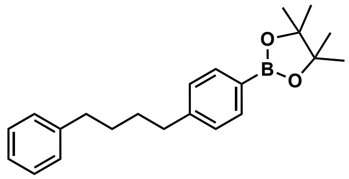

Following **general procedure B** on a 0.075 mmol scale of **1** and 3 equiv of **9**. After passing 3.7 F/mol of charge (1.5 h) and standard workup, the product was purified by PTLC (SiO_2, hexane:EtOAc 19:1) to afford 9.4 mg (37%) of **11b** as a yellow oil.

^{1}H NMR (600 MHz, CDCl3): δ 7.74 (d, J = 8.1 Hz, 2H), 7.28 (s, 1H), 7.22 – 7.17 (m, 6H), 2.66 (p, J = 7.3 Hz, 4H), 1.68 (p, J = 3.3 Hz, 4H), 1.36 (s, 12H).

^{13}C NMR (151 MHz, CDCl3): δ 146.2, 142.7, 135.0, 128.6, 128.4, 128.1, 125.8, 83.8, 36.1, 35.9, 31.2, 25.0.

^{11}B NMR (96 MHz, $CDCl_3$): δ 32.4.

GC/MS(EI): 336 (43.23%), 117 (70.23%), 91 (100%)

HRMS (ASAP-TOF): calc'd for $C_{22}H_{30}BO_2$ $[M+H]^+$: 337.2333; found: 337.2491

Physical State: Yellow oil

TLC: R_f= 0.5 (hexane:EtOAc 19:1)

Compound 12

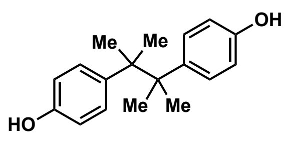

Following the **general procedure C** on a 0.3 mmol scale with **S2**. After passing 4.9 F/mol of charge (4 h) and standard workup, the product was purified by PTLC (SiO_2, hexane:EtOAc 19:1) to afford 4,4'-(2,3-dimethylbutane-2,3-diyl)bis(methoxybenzene) in 50% yield (22.4 mg) as a white solid. The obtained compound was demethylated following the literature procedure[22]. The final product **12** was obtained in an overall 50% yield (20 mg).

^{1}H NMR (400 MHz, $CDCl_3$): δ 6.91 (d, J = 8.8 Hz, 4H), 6.67 (d, J = 8.9 Hz, 4H), 1.29 (s, 12H).
^{13}C NMR (151 MHz, $CDCl_3$): δ 153.3, 139.2, 129.9, 113.4, 43.2, 25.3.
Physical State: White solid
HRMS (ESI-TOF): calc'd for $C_{18}H_{21}O_2$ [M-H]$^-$: 269.1542; found: 269.1542
TLC: R_f=0.4 (hexane:EtOAc 4:1)

Compound 13

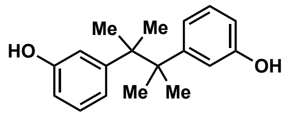

Following the **general procedure C** on a 0.3 mmol scale with **S3**. After passing 4.9 F/mol of charge (4 h) and standard workup, the product was purified by PTLC (SiO_2, hexane:EtOAc 19:1) to afford 3,3'-(2,3-dimethylbutane-2,3-diyl)bis(methoxybenzene) in 40% yield (18 mg) as a white solid. The obtained compound was demethylated following the literature procedure[22]. The final product **13** was obtained in an overall 40% yield (16.3 mg).

^{1}H NMR (400 MHz, $CDCl_3$/MeOD): δ 7.09 (t, J = 8.0 Hz, 2H), 6.76 – 6.62 (m, 4H), 6.54 (d, J = 2.2 Hz, 2H), 1.31 (s, 12H).
^{13}C NMR (151 MHz, $CDCl_3$/MeOD): δ 154.9, 148.8, 127.48, 120.6, 116.2, 112.2, 43.62, 25.2.
Physical State: White solid
HRMS: calc'd for $C_{18}H_{21}O_2$ [M-H]: 269.1542; found: 269.1539
TLC: R_f=0.4 (hexane:EtOAc 4:1)

Compound 14

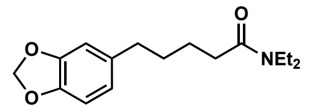

Following **general procedure B** on a 0.075 mmol scale of **S5** and 3 equiv of **S1**. After passing 3.7 F/mol of charge (1.5 h) and standard workup, the product was purified by PTLC (SiO_2, hexane:EtOAc 3:2) to afford 11.2 mg (54% yield) of **14** as a white solid.

^{1}H NMR (600 MHz, CDCl3): 6.76 – 6.62 (m, 3H), 5.93 (s, 5H), 3.38 (q, J = 7.1 Hz, 2H), 3.30 (q, J = 7.1 Hz, 2H), 2.58 (t, J = 7.4 Hz, 2H), 2.32 (d, J=14.7 Hz, 2H), 1.74 – 1.60 (m, 4H), 1.17 (t, J = 7.1 Hz, 3H), 1.12 (t, J = 7.1 Hz, 3H).

^{13}C NMR (151 MHz, CDCl3): δ 172.1, 136.4, 121.2, 109.0, 108.2, 100.8, 42.1, 40.2, 35.7, 33.1, 31.7, 25.2, 22.8, 14.6, 14.3, 13.3.

GC/MS(EI): 277 (59.94%), 135 (63.16%), 115 (100%)

Physical State: white solid

TLC: R_f= 0.3 (hexane:EtOAc 3:2)

The spectroscopic data are consistent with those reported in the literature.[23]

Compound 15

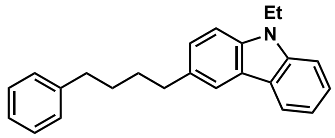

Following **general procedure B** on a 0.075 mmol scale of **1** and 3 equiv of **S4**. After passing 3.7 F/mol of charge (1.5 h) and standard workup, the product was purified by PTLC (SiO_2, hexane:EtOAc 20:1) to afford **15** as a yellow oil in 46% yield.

^{1}H NMR (300 MHz, $CDCl_3$): δ 8.09 (d, *J* = 7.8 Hz, 1H), 7.91 (s, 1H), 7.51 – 7.17 (m, 10H), 4.37 (q, *J* = 7.2 Hz, 1H), 2.85 (t, *J* = 7.3 Hz, 2H), 2.70 (t, *J* = 7.3 Hz, 2H), 1.88 – 1.66 (m, 4H), 1.45 (t, *J* = 7.2 Hz, 3H).

^{13}C NMR (126 MHz, $CDCl_3$): δ 142.7, 140.2, 138.5, 133.0, 128.5, 128.3, 126.4, 125.6, 125.4, 123.0, 122.8, 120.3, 119.8, 118.5, 108.4, 108.1, 37.5, 35.9, 35.9, 32.01, 31.17, 13.8.

HR-MS: measured: 328.1922 $(M+H)^+$ calc'd: 328.1987

HRMS (ASAP-TOF): calc'd for $C_{24}H_{26}N$ $[M+H]^+$: 328.2060; found: 328.1922

Physical State: White solid

TLC: R_f= 0.5 (hexane:EtOAc 20:1)

Compound 16

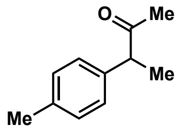

Following the **general procedure E** on a 0.3 mmol scale with **S6**. After passing 6.2 F/mol of charge (2.5 h) and standard workup, the product was purified by PTLC (SiO_2, hexane:EtOAc 5:1) to afford 19 mg (39% yield) of **16** as a yellow oil.

^{1}H NMR (400 MHz, $CDCl_3$): δ 7.20 – 7.09 (m, 3H), 3.73 (q, *J* = 6.9 Hz, 1H), 2.36 (s, 3H), 2.06 (s, 3H), 1.39 (d, *J* = 7.0 Hz, 3H).
^{13}C NMR (151 MHz, $CDCl_3$): δ 209.3, 137.7, 137.0, 129.7, 127.8, 53.4, 28.4, 21.1, 17.3.
GC/MS(EI): 162 (10.46%), 91 (18.89%), 119 (100%)
Physical State: yellow oil
TLC: R_f= 0.7 (hexane:EtOAc 5:1)
The spectroscopic data are consistent with those reported in the literature.[24]

Compound 17

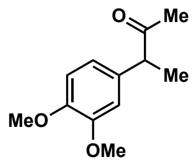

Following the **general procedure E** on a 0.3 mmol scale with **S7**. After passing 6.2 F/mol of charge (2.5 h) and standard workup, the product was purified by PTLC (SiO_2, hexane:EtOAc 6:1) to afford 31 mg (50% yield) of **17** as a yellow oil.

^{1}H NMR (400 MHz, $CDCl_3$): δ 6.87 – 6.67 (m, 3H), 3.89 (s, 6H), 3.70 (q, *J* = 7.0 Hz, 1H), 2.07 (s, 3H), 1.39 (d, *J* = 7.0 Hz, 3H).
^{13}C NMR (151 MHz, $CDCl_3$): δ 209.3, 149.3, 148.2, 133.1, 120.1, 111.4, 110.6, 56.0, 53.3, 28.3, 17.3.
GC/MS(EI): 208 (15.22%), 105 (17.57%), 165 (100%)
Physical State: yellow oil
TLC: R_f= 0.6 (hexane:EtOAc 6:1)
The spectroscopic data are consistent with those reported in the literature.[25]

Compound 18

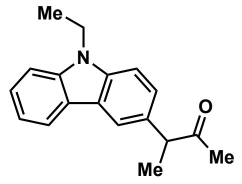

Following the **general procedure E** on a 0.3 mmol scale with **S4**. After passing 6.2 F/mol of charge (2.5 h) and standard workup, the product was purified by PTLC (SiO_2, hexane:EtOAc 9:1) to afford 41.5 mg (52% yield) of **18** as a white solid.

^{1}H NMR (600 MHz, CDCl$_3$): δ 8.11 (d, *J* = 8.8 Hz, 1H), 7.96 (d, *J* = 1.8 Hz, 1H), 7.52 – 7.22 (m, 6H), 4.39 (q, *J* = 7.3 Hz, 2H), 3.95 (q, *J* = 7.0 Hz, 1H), 2.10 (s, 3H), 1.52 (d, *J* = 7.0 Hz, 3H), 1.46 (t, *J* = 7.3 Hz, 3H).

^{13}C NMR (151 MHz, CDCl$_3$): δ 209.85, 140.40, 139.30, 131.17, 126.01, 125.56, 123.51, 122.71, 120.60, 119.72, 119.04, 109.01, 108.68, 53.90, 37.74, 28.51, 17.90, 13.98.

GC/MS(EI): 265 (16.60%), 193 (16.92%), 222 (100%)

Physical State: white solid

TLC: R$_f$= 0.5 (hexane:EtOAc 9:1)

Compound 19

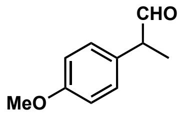

Following the **general procedure G** on a 0.3 mmol scale with 4-vinylanisole. After passing 6.2 F/mol of charge (2.5 h) and standard workup (NH_4Cl aqueous sol. instead of HCl 1M sol.), the product was purified by PTLC (SiO_2, hexane:EtOAc 19:1) to afford **19** (30% yield, determined by ^{1}H NMR) as a white solid.

^{1}H NMR (800 MHz, $CDCl_3$): δ 9.64 (d, J = 1.5 Hz, 1H), 7.13 (d, J = 8.6 Hz, 2H), 6.91 (d, J = 8.8 Hz, 2H), 3.81 (s, 3H), 3.61 – 3.56 (m, 1H), 1.41 (d, J = 7.0 Hz, 3H).

^{13}C NMR (201 MHz, $CDCl_3$): δ 203.90, 161.67, 132.21, 132.03, 117.16, 79.84, 79.68, 79.52, 57.97, 54.82, 17.33.

Physical State: white solid

GC/MS(EI): 164 (9.32%), 105 (19.3%), 135 (100%)

TLC: R_f=0.4 (19:1 Hexane : EtOAc)

The spectroscopic data are consistent with those reported in the literature.[26]

Radical Clock Experiments

α Position – cyclopropyl with a phenyl ring (Branched)

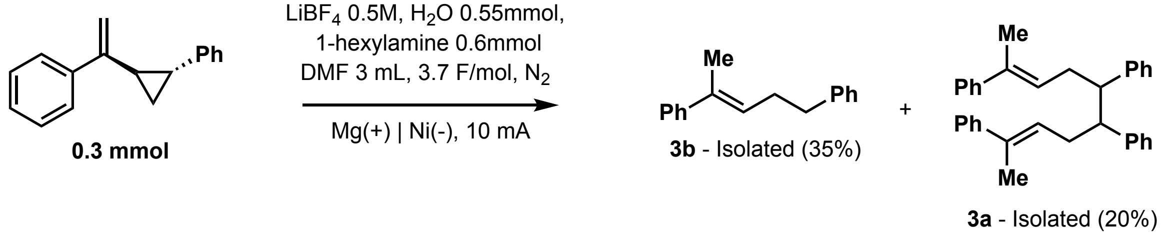

Following the **general procedure C** on a 0.3 mmol scale with **3**. After passing 3.7 F/mol of charge (3 h) and standard workup, products were purified by PTLC (SiO_2, 100% hexane) to afford 23.3 mg (35% yield) of **3b** as a yellow oil and 13.3 mg (20% yield) of **3a** as a white solid, for obtaining clean NMR spectra compounds after PTLC were purified with preparative HPLC (MeCN:water). For **3a**, only a single isomer was isolated in pure form.

Product **3a** characterization

^{1}H NMR (500 MHz, $CDCl_3$): δ 7.39 – 7.08 (m, 20H), 5.46 (t, *J* = 7.5 Hz, 2H), 3.05 – 2.98 (m, 2H), 2.40 – 2.23 (m, 4H), 1.67 (s, 6H).

^{13}C NMR (151 MHz, $CDCl_3$): δ 144.2, 144.0, 135.4, 128.5, 128.1, 126.6, 126.5, 125.7, 52.1, 34.1, 29.9, 15.8.

Physical State: White solid

HR-MS: calc'd for $C_{34}H_{35}$: 443.6535, measured: 443.2737 $(M+H)^+$

TLC: R_f=0.1 (100% hexane)

Product **3b** characterization

^{1}H NMR (800 MHz, $CDCl_3$): δ 7.40 – 7.17 (m, 10H), 5.81 (td, *J* = 7.2, 1.4 Hz, 1H), 2.79 – 2.73 (m, 2H), 2.52 (q, *J* = 7.5 Hz, 2H), 1.97 (s, 3H).

^{13}C NMR (201 MHz, $CDCl_3$): δ 144.2, 142.3, 135.7, 128.8, 128.6, 128.5, 127.7, 126.9, 126.1, 125.9, 36.1, 31.1, 16.1.

Physical State: Yellow oil

GC/MS(EI): 222 (11.95%), 91 (31.78%), 131 (100%)

TLC: R_f=0.5 (100% hexane)

The spectroscopic data are consistent with those reported in the literature.[27]

β Position – cyclopropyl with a phenyl ring (Branched)

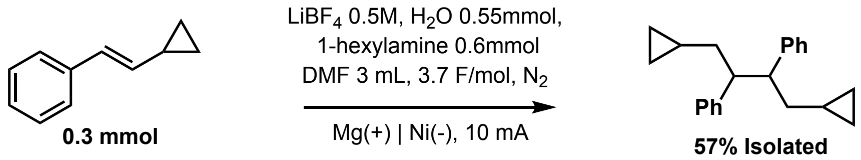

Following the **general procedure C** on a 0.3 mmol scale with **2**. After passing 3.7 F/mol of charge (3 h) and standard workup, the product was purified by PTLC (SiO_2, 100% hexane) to afford 17.6 mg (57% yield) of **2e** as a white solid (mixture of 2 diastereomers 1:6, determined by GC-MS).

^{1}H NMR (600 MHz, $CDCl_3$): δ 7.35 – 7.17 (m, 10H), 2.88 – 2.80 (m, 2H), 1.34 (m, 4H), 0.93 – 0.82 (m, 2H), 0.28 – 0.14 (m, 4H), -0.25 – -0.33 (m, 4H).

^{13}C NMR (151 MHz, $CDCl_3$): δ 145.1, 128.5, 128.3, 126.1, 52.5, 40.0, 9.5, 5.1, 4.1.

Physical State: White solid

GC/MS(EI): 145 (27.0%), 144 (32.9%), 91 (100%)

TLC: R_f=0.5 (100% hexane)

β Position – cyclopropyl with a phenyl ring (Linear)

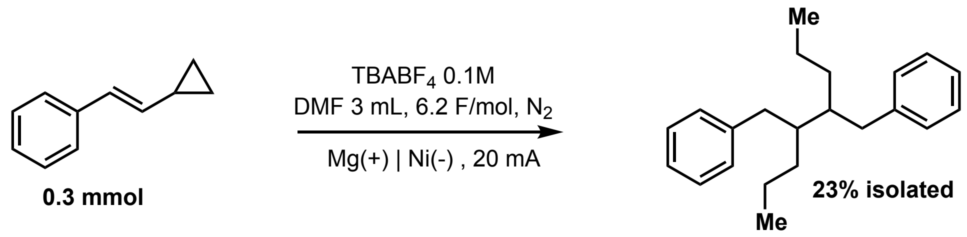

Following the **general procedure A** on a 0.3 mmol scale with **2**. After passing 6.2 F/mol of charge (2.5 h) and standard workup, the product was purified by PTLC (SiO_2, 100% hexane) to afford 9.6 mg (23% yield) of **2c** as a yellow oil.

^{1}H NMR (300 MHz, $CDCl_3$): δ 7.27 – 6.97 (m, 10H), 2.53 (ddd, J = 83.3, 13.7, 7.1 Hz, 4H), 1.75 – 1.67 (m, 2H), 1.37 – 1.15 (m, 8H), 0.86 (t, J = 7.2 Hz, 6H).

^{13}C NMR (151 MHz, $CDCl_3$): δ 142.1, 129.2, 128.2, 125.6, 40.4, 37.1, 32.3, 21.1, 14.5.

Physical State: yellow oil

GC/MS(EI): 294 (2.35%), 105 (12.86%), 91 (100%)

TLC: R_f=0.5 (100% hexane)

The spectroscopic data are consistent with those reported in the literature.[28]

2. Solid-state NMR

The layer that was analyzed in the solid-state NMR was prepared by running a reaction in standard procedure **C**, inside a nitrogen-filled glovebox. Upon the completion of the reaction, the electrasyn cap was unscrewed, and the Ni cathode was washed six times with DMF. Later, the electrode was sealed in a septum-equipped vial and taken outside the glovebox. Immediately after, the vial was purged with argon, using a balloon, and taken to an argon-filled glovebox, where the NMR sample was prepared by scraping the deposited Li layer.

Solid-state NMR measurements were performed on a 9.4 T 400MHz solid-state NMR Bruker Avance Neo spectrometer. The measurements were performed using a 2.5 mm triple-resonance magic-angle-spinning probe with a sample spinning frequency of 25kHz.

For ^{7}Li (Larmor frequency of 155 MHz), direct excitation was used with radio-frequency pulses of 125 kHz amplitude and a relaxation delay of 20 s. This delay was chosen following optimization to obtain a quantitative spectrum of both metallic and diamagnetic lithium environments. ^{7}Li shifts were calibrated relative to LiF at -1 ppm.

^{1}H spectra were acquired with a rotor-synchronized Hahn echo using radio-frequency pulses with an amplitude of 133 kHz and a 5 s relaxation delay (during which the resonances were fully relaxed). The spectra were referenced to adamantane set at 1.8 ppm.

Continuous-wave (CW) EPR measurements in the X-band (~9.4 GHz) were performed on a Bruker Magnettech ESR5000 spectrometer, with a modulation frequency of 100 kHz. Spectra were acquired at 100 K.

3. Computational Details

Density Functional Theory

Styrene was optimized as a negatively charged open-shell doublet using the CPCM implicit solvent model with DMF at the UKS-ωB97X-D3[29]/cc-pVTZ[30–33] level of theory with Orca 6.1.0.[34] Styrene radical anion-ion complexes of styrene with lithium and tetrabutylammonium ions were optimized at the same level of theory. An additional set of structures with two ions, one on either side of the styrene radical, were also optimized. Vibrational frequencies of each optimized structure were obtained at the same level of theory, and a two-point complete basis set (CBS) energy extrapolation was performed using cc-pVXZ, where X=T, Q, with a power law expression given in Equation 1.[35]

$$E_X = E_{CBS} + AX^{-3} \quad (1$$

These resulting CBS electronic energies and vibrational frequencies were then used to estimate the Gibbs free energy of binding for each complex at 300 K relative to the asymptotic reactants.

Classical Simulations

To study the impact of the electrical double layer on the behavior of styrene, classical simulations of a single radical styrene molecule under dilute conditions in DMF were performed with LiCl or $N(Bu)_4Cl$ using GROMACS 2025.3.[36,37] For styrene, fixed atomic charges were generated from a RESP charge fitting performed using Psi4[38], while the bonding parameters were generated using GAFF2.[39] Charges and parameters for DMF and $N(Bu)_4^+$ were also generated using the GAFF2 force field. The Lennard-Jones parameters for the nickel electrodes were taken from Heinz et al.[40], while lithium and chloride were obtained from Li et al.[41] Radical styrene was first solvated in a box of DMF (10.22 nm x 10.36 nm x 10.0 nm). The X and Y dimensions were chosen to match the final X/Y dimensions of the nickel electrodes. Separate simulations were performed for LiCl and $N(Bu)_4Cl$ using an ion concentration of 0.1 mM. For each system, an additional ion was added to balance the charge of the negatively charged styrene radical. Each system was first minimized using a 1000 $kJ*mol^{-1}*nm^{-1}$ convergence criterion, a maximum step size of 0.01 nm, and up to 50,000 steps.

The minimized structures were then thermally equilibrated for 10 ns using the NVT ensemble at 300 K with the Nose–Hoover thermostat (τ_T = 0.1 ps), followed by 50 ns of NPT simulation at 1 bar using the Parrinello–Rahman barostat (τ_p = 10 ps) with semiisotropic coupling and a compressibility factor of 4.5×10^{-5} in the Z-direction and no compressibility in X, Y directions to maintain the same dimensionality as the nickel electrodes. Bonds involving hydrogen atoms were constrained during each phase of the simulations using the LINCS algorithm, allowing a 2 fs time step for dynamics. Electrostatic interactions were calculated using the Particle Mesh Ewald (PME) method with a cutoff of 1.2 nm, while Lennard–Jones interactions were truncated at the same cutoff distance. Periodic boundary conditions were used during both NVT and NPT equilibration steps. Next, the equilibrated simulation boxes were placed between (111) nickel electrodes at the ends of the Z-direction. The nickel electrodes themselves were composed of 5049 atoms generated from the CHARMM-GUI website[42]. When combining the solvent box with the nickel electrodes, small spacings were added between the Z-directional ends of the solvent box to prevent overlap between protruding molecules and the electrodes of 5 Å for Li^+ and 9 Å for $N(Bu)_4{}^+$. The partial atomic charge of each nickel atom was set to 0.003157 a.u. (-0.003157 a.u. for the cathode) to produce a -2.5 V drop across the simulation box. The final simulation box was then thermally equilibrated with the NVT ensemble using two-dimensional periodic boundary conditions and PME electrostatics with the slab correction (the Yeh–Berkowitz scheme) to suppress spurious image interactions along the Z-direction. For these and subsequent simulations, the metal electrodes were frozen in place.

Table S19 - Spin Populations on C1 and C2 carbons from DFT optimized structures, along with the corresponding changes in the Gibbs free energy, enthalpy and entropy upon complex formation in kcal/mol at 300 K.

**Model**	**C1**	**C2**	**ΔG**	**ΔH**	**TΔS**
Li^+	0.253	0.147	-24.51	-32.24	-7.64
$[N(Bu)_4]^+$	0.305	0.091	2.75	-11.10	-13.85
$2Li^+$	0.248	0.165	-38.3	-52.20	-13.90
$2[N(Bu)_4]^+$	0.303	0.090	4.89	-20.99	-25.88
Without Ions	0.312	0.098	0	0	0

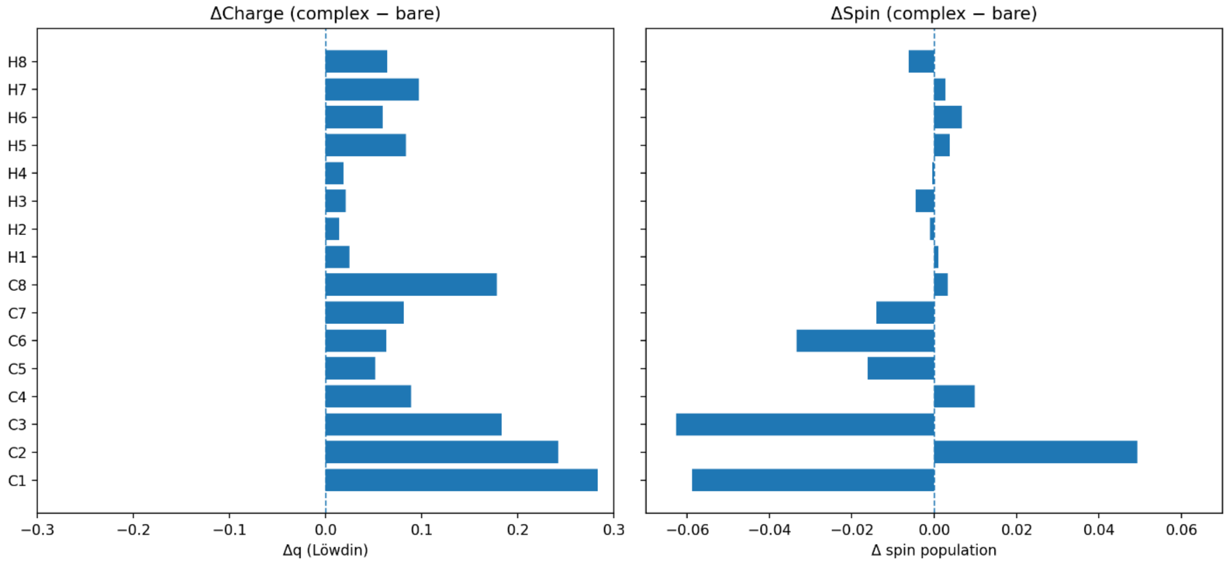

Figure S1. Change in the Löwdin charges and spin population for the hydrogen atoms and heavy atoms of styrene in complex with Li^+.

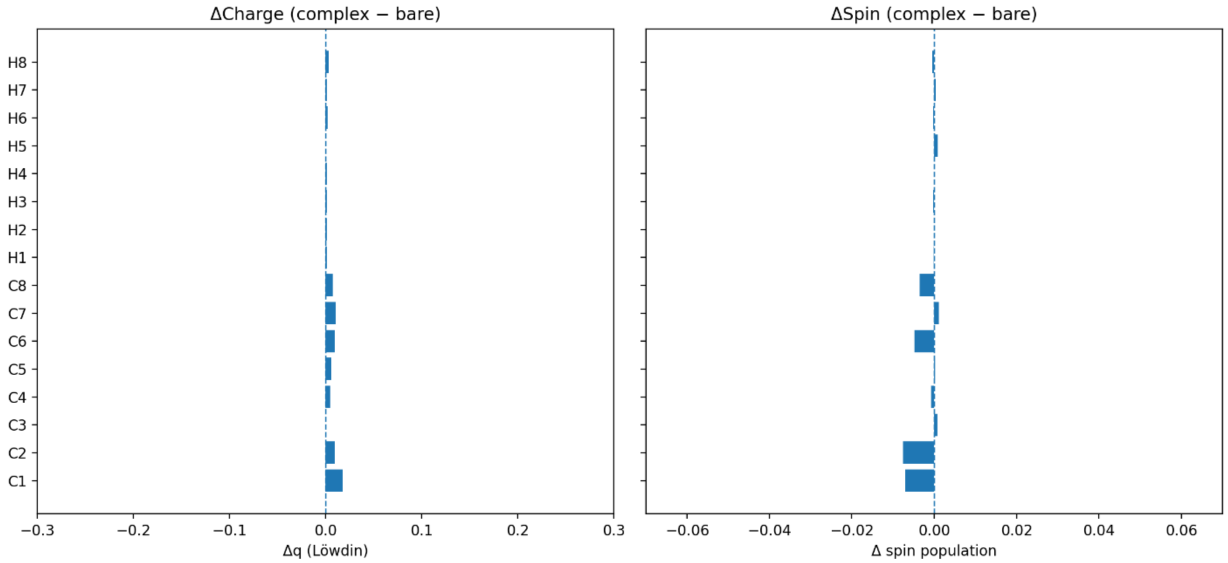

Figure S2. Change in the Löwdin charges and spin population for the hydrogen atoms and heavy atoms of styrene in complex with $N(Bu)_4^+$.

Umbrella Sampling

The thermally equilibrated systems were then used for umbrella sampling simulations. Here, initial configurations were generated from two 50 ns steered molecular dynamics (MD) simulations

pulled in both Z-directions. During these simulations, the center-of-mass (COM) of styrene was pulled towards and away from the negative electrode, with a harmonic restraint (1250 kJ*mol^{-1} *nm^{-2}) applied to the COM along the Z-direction. Evenly spaced sampling windows were generated at 2 Å intervals along the Z-direction, each employing the same harmonic restraints as above. Each window was simulated for 50 ns in the NVT ensemble, using the same simulation settings as previously outlined. The final 25 ns of each trajectory were then used to calculate the potential of mean force (PMFs) using the weighted histogram analysis method (WHAM) implemented in GROMACS. Bayesian bootstrapping was applied with 100 resamples to estimate PMF uncertainties.

4. Additional data

Kinetics experiment of hetero-coupling

Figure S3 – Monitoring of the reaction kinetics of styrene and 4-vinylanisole heterocoupling under $TBABF_4$ conditions (procedure **A**)

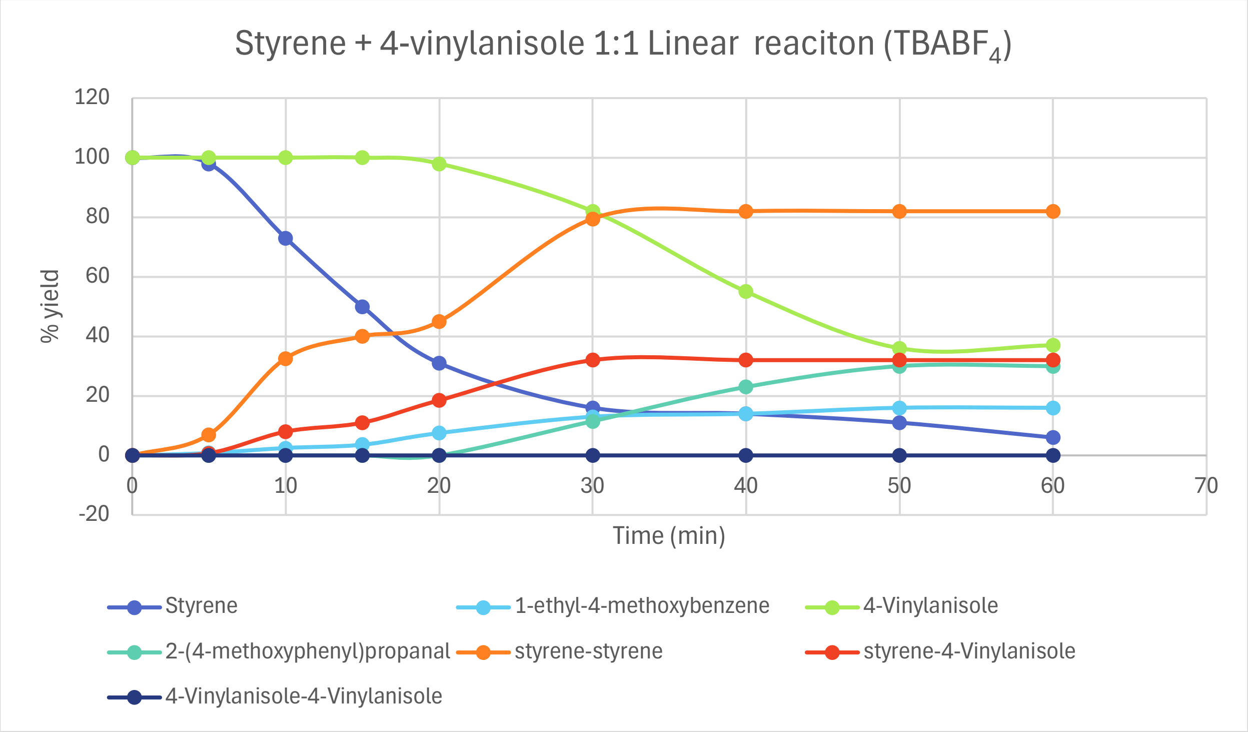

Figure S4 – Monitoring of the reaction kinetics of styrene and 4-vinylanisole heterocoupling under $LiBF_4$ conditions (procedure **C**)

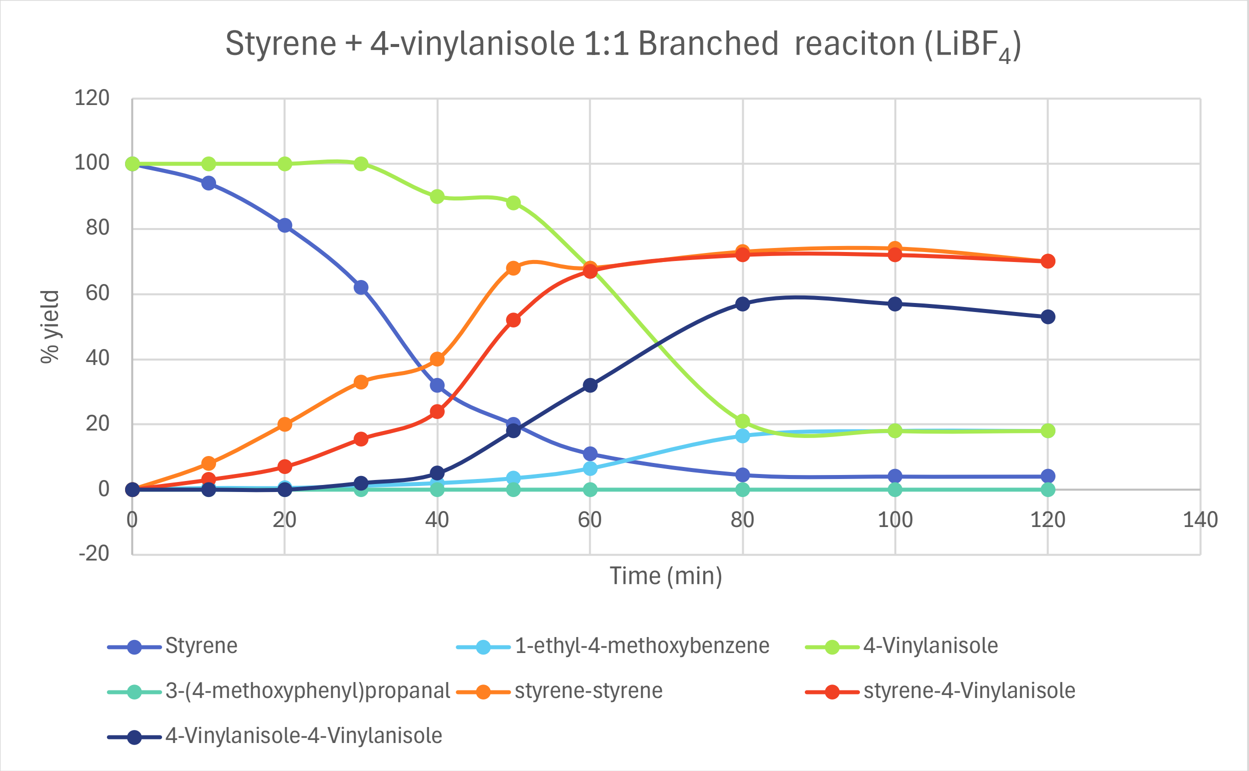

SEM imaging of a blank nickel electrode

Figure S5

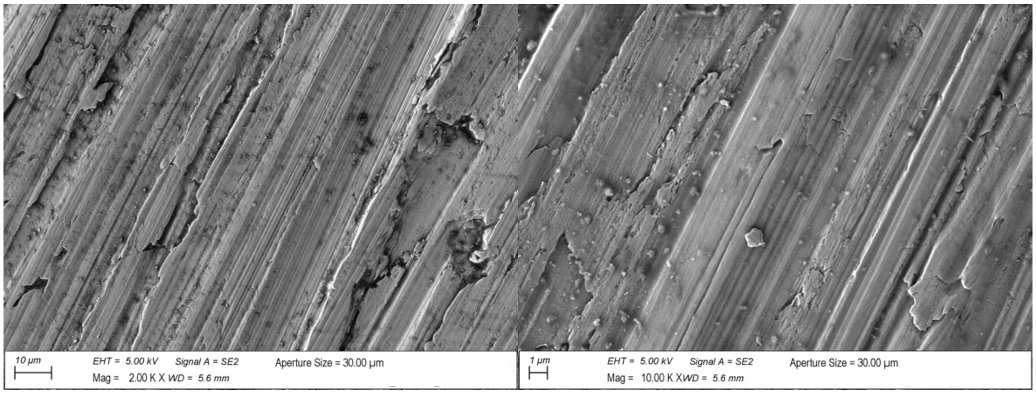

Constant Current Electrolysis Experiment

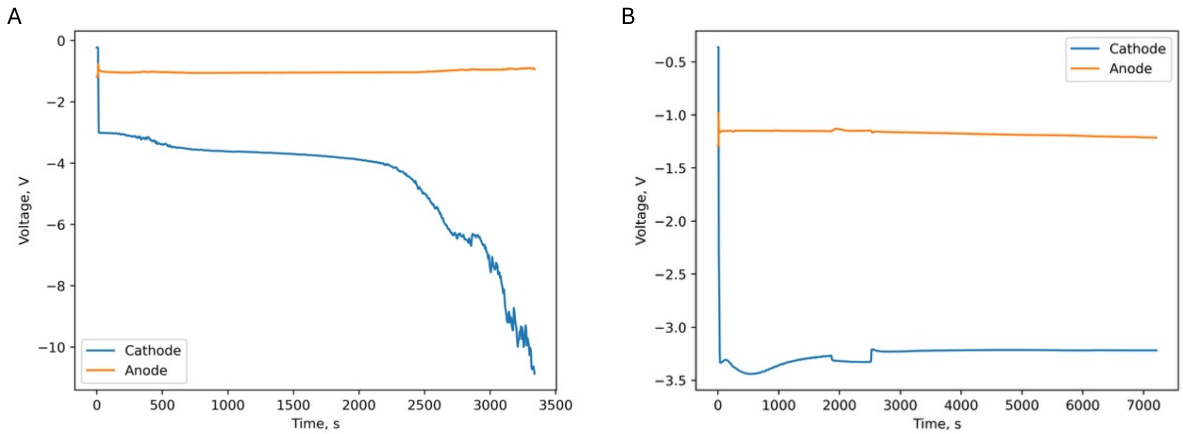

Figure S6 – Voltage on cathode and anode during a reduction of styrene in (A) linear conditions with $TBABF_4$ as electrolyte, (B) branched conditions with Li as electrolyte.

Predicted UV-vis spectra of styrene 9 radical anion with Bu_4N^+ and Li^+

We propose that the distinct colors observed in the reaction mixtures arise from the different electronic nature of the species participating in delocalization with the aromatic ring: specifically, the anionic character of intermediate **A** versus the radical character of intermediate **B**. This phenomenon occurs because the non-bonding orbital of the anion is typically higher in energy than the singly occupied molecular orbital of the corresponding radical. This energy difference narrows the HOMO–LUMO gap, resulting in a bathochromic shift. This effect is supported by comparing the absorption maxima of benzylic radicals and anions derived from toluene (~315 nm for the radical vs. ~350 nm for the anion) and diphenylmethane (~325 nm for the radical vs. ~450 nm for the anion).[43-46] Together, these studies motivate a conservative mechanistic interpretation relevant here: changes in charge delocalization and counterion interactions can tune absorption band profiles, and the observed color can therefore report on which electronically distinct intermediate is populated under a given set of reaction conditions.

To further support this conclusion, we calculated absoprtion spectra of the isolated radical anion (of compound **9**) as well as in complex with Bu_4N^+ and $2Li^+$ species from the 10 lowest electronic states calculated with TD-DFT calculations at the ωB97X-D/cc-pVTZ level of theory with CPCM implicit solvent model (DMF). As shown in Figure S7B (blue), the negatively charged open-shell doublet displays a dominant π–π* transition in the visible region, with absorption maxima at 495 and 668 nm. Consistent with the conclusions of this study, complexation with a Bu_4N^+ counterion largely preserves the spectral signature of the reduced doublet, giving comparable absorbance features (Figure S7B, green). In contrast, coordination with Li^+ markedly suppresses the radical-anion absorption, leading to a pronounced blue shift and substantially lower normalized intensities (Figure S7B, orange).

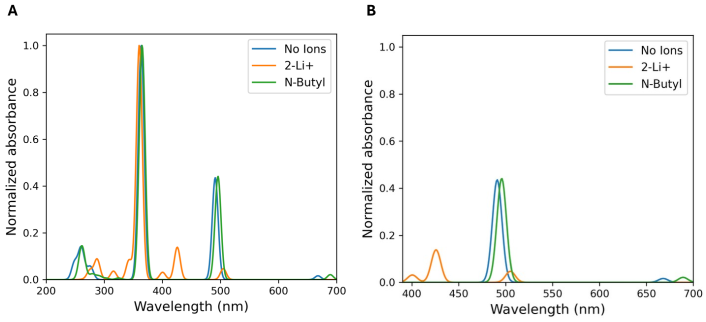

Figure S7: A) TD-DFT predicted absorption spectrumabsorbance spectrum of **9**-radical anion species. B) Visible light region of the **9**-radical anion species absorbance spectrum.

Control experiments for radical clock analysis

It is well established that, under electroreductive conditions, strained cyclopropyl rings can undergo ring opening.[6,7] Therefore, throughout this study, we took care to distinguish ring opening that arises from radical intermediacy from ring opening that could occur via direct reduction of the cyclopropane. To this end, for each radical-clock experiment, we performed an appropriate control under closely matched conditions. For substrate **3**, we prepared control compound **3y**, which contains a polarized cyclopropyl group but bears an inactive, non-conjugated olefin that is not expected to undergo reduction. Compound **3y** was evaluated under both the branched and linear conditions. Under the branched conditions, we observed quantitative recovery of the starting material (Figure S8A). In contrast, subjecting **3y** to the linear conditions led to extensive background ring opening, affording 86% ring-opened products (Figure S8B). To further corroborate this behavior, we examined a second control, **3z** (Figure S8C), a polarized and activated cyclopropane lacking an olefin. Under the linear conditions, **3z** likewise underwent substantial ring opening (88%).

Taken together, these results indicate that **3** functions as a reliable radical probe under the **branched conditions** (Figure 2A, Manuscript), but it is **not an appropriate negative control under the linear conditions**, where cyclopropane ring opening proceeds readily as a background process independent of radical formation.

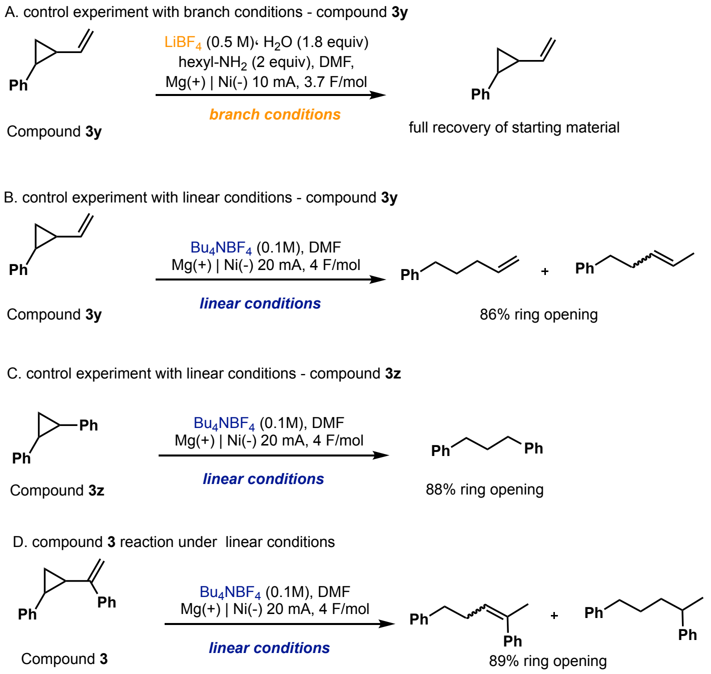

Figure S8: Radical clock control experiments of compounds **3y** and **3z**.

We also performed the same set of control experiments for radical probe **2**. In this case, the corresponding control substrate **2y** showed quantitative recovery of the starting material under the reaction conditions, and no ring-opening products were detected (Figure S9).

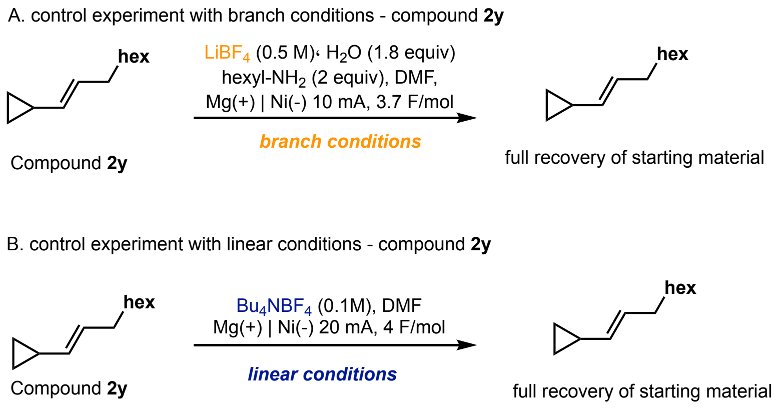

Figure S9: Radical clock control experiments of compound **2y**.

Constant potential experiment under the linear conditions

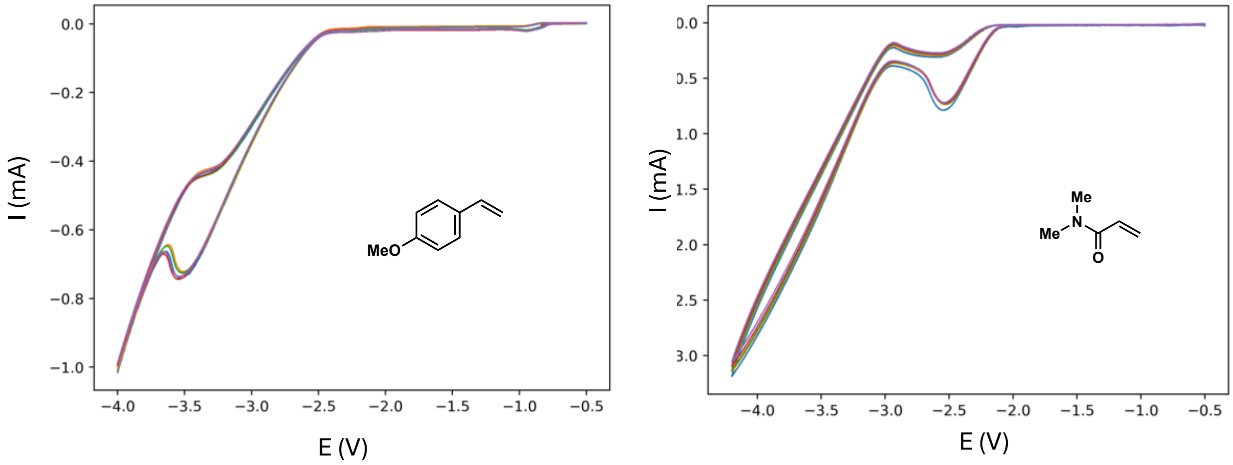

Figure S10: CV plots of methoxystyrene and acrylamide.

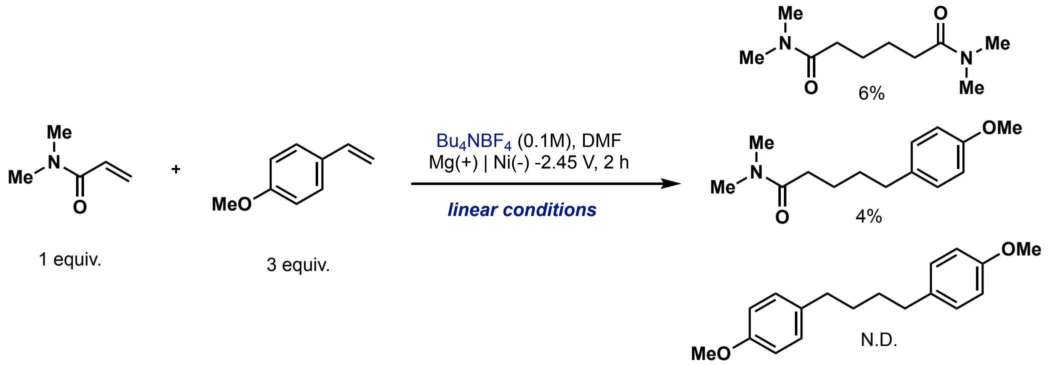

Figure S11: Constant potential reaction of methoxystyrene and acrylamide.

CV analysis of EtOAC and acylation reaction

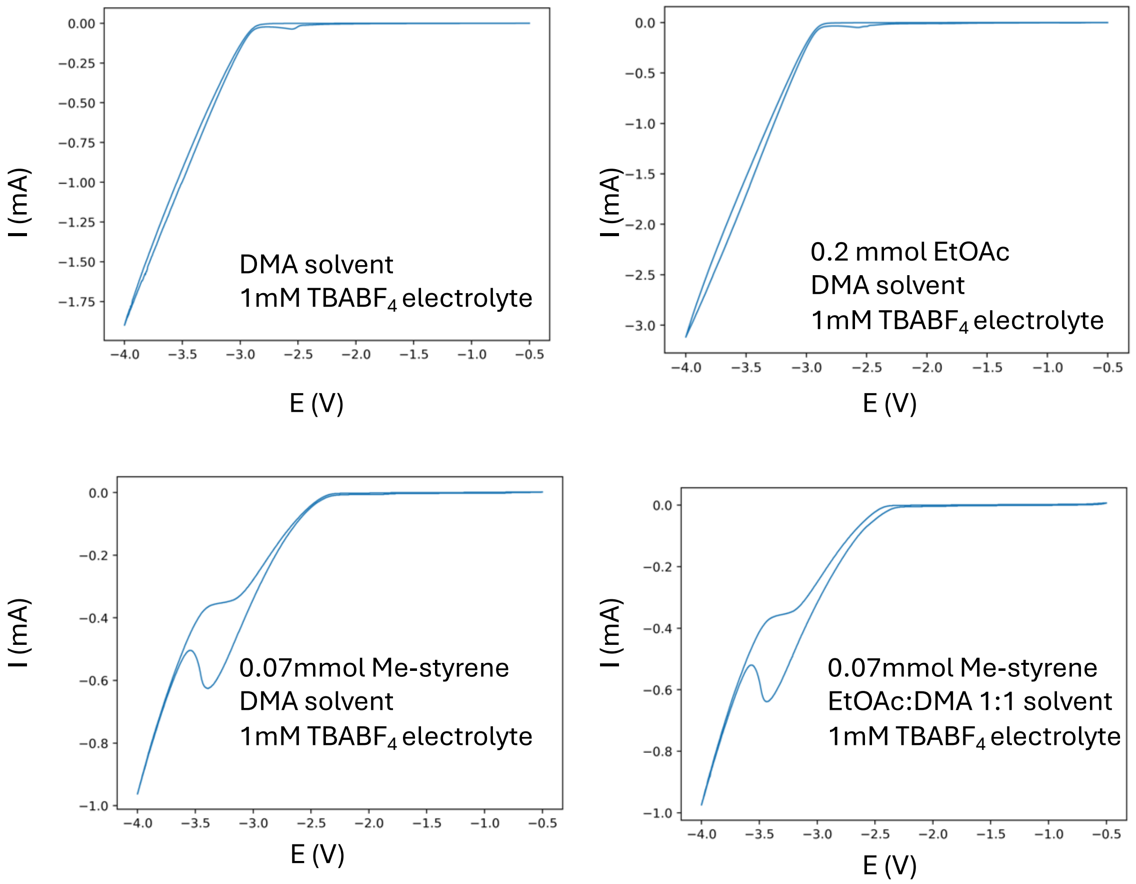

Figure S12: CV analysis of DMA, EtOAc, and styrene in DMA or DMA:EtOAc mixture.

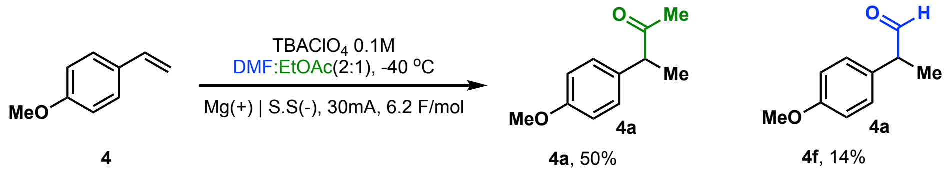

Figure S13: Formylation and acylation reaction with a mixture of EtOAc and DMF.

5. Mechanism Discussions

Linear coupling

A full proposed mechanism for the formation of the linear homocoupling product is demonstrated below (Figure S14).

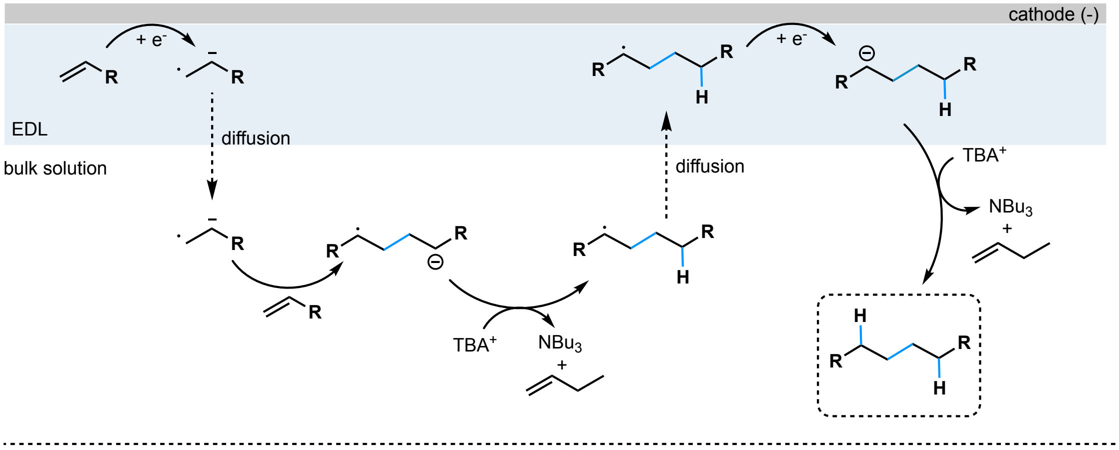

Figure S14: Proposed mechanism for the formation of linear homocoupling products

<u>Another option for the mechanism and the contradictions with it</u>

a) Proton source

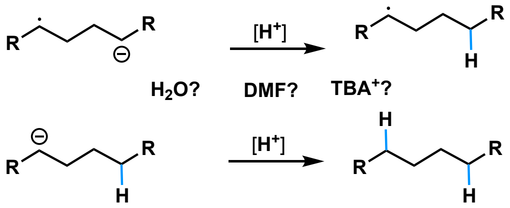

Figure S15: Stages requiring a proton source and potential "candidates" for it

A proton source is essential for material balance in the reaction (Figure S15), with potential sources including the electrolyte (TBA+ salt), residual water, and the solvent (DMF). As DMF is considered an insufficiently weak proton donor for this transformation, the active source is hypothesized to be the electrolyte, supported by two key observations: GC-MS analysis of the crude mixture shows a peak corresponding to tributylamine whose concentration increases with reaction time (implying TBA+ consumption), and a rapid increase in cell voltage is noted during the final reaction stages, which is attributed to electrolyte depletion and a consequent rise in ohmic resistance.

Branched coupling

A full proposed mechanism for the formation of the branched homocoupling product is demonstrated below (Figure S16).

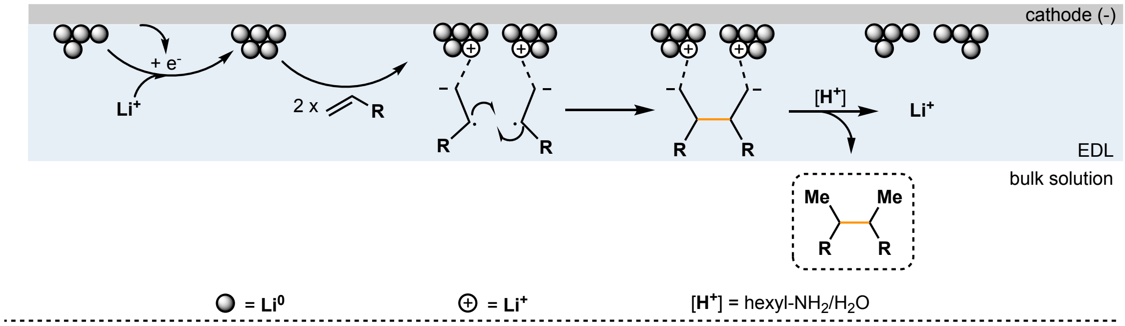

Figure S16: Proposed mechanism for the formation of branched homocoupling products

<u>Possible deviations from proposed mechanisms:</u>

a) Direct reduction vs mediated

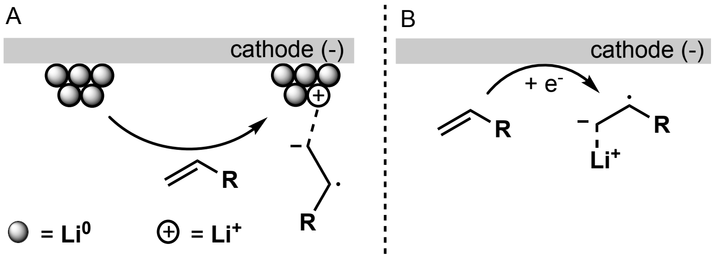

Figure S17: Possible pathways for olefin reduction. A) Li-mediated reduction, B) Direct reduction

Olefin reduction may proceed via (a) mediation by in situ-generated lithium nanoparticles or (b) direct electrode electron transfer (Figure S17). Direct transfer cannot be fully excluded; however, a single CV reduction peak (assigned to Li deposition) and product formation with a pre-electrolyzed Li layer (Figure 4C, entry 2) allow us to propose lithium-mediated reduction as the dominant pathway.

b) Radical-radical coupling vs radical addition

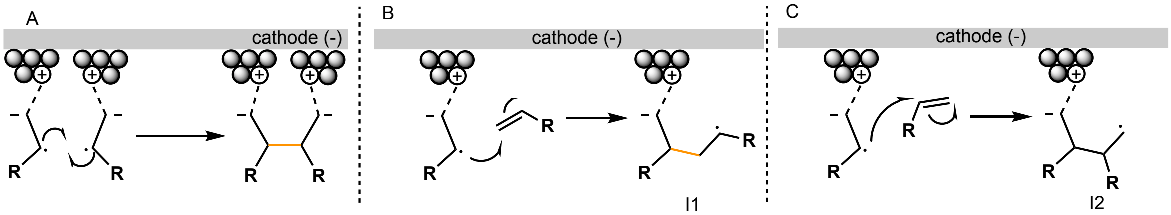

Figure S18: Possible pathways for the formation of the C-C bond. A) radical-radical recombination, B) radical attack to the terminal carbon, C) radical attack to the internal carbon

C–C bond formation may proceed via (a) radical–radical coupling or (b) radical addition, yielding branched or "semi-branched" regioisomers (Figure S18). The near-statistical distribution in the cross-olefin coupling experiment (Figure 3B) supports radical-radical recombination. Moreover, a radical addition mechanism would favor the "semi-branched" intermediate **I1** compared to **I2** due to benzylic radical stability and reduced steric hindrance at the terminal carbon; however, no product with such a skeleton was observed.

Diastereoselectivity differences between Linear and Branch reactions:

Across our dataset, the branch-selective conditions consistently deliver statistical diastereoselectivity (d.r. ≈ 1:1, meso:racemic), as illustrated by a representative example in Figure 19. In contrast, when disubstituted olefins were subjected to the linear-selective conditions (e.g., substrate **20**, Figure 19), we observed measurable diastereoselectivity in the newly formed C–C bond of the linear product. This divergence suggests that the bond-forming event under linear conditions is distinct from that operating in the branch-selective manifold.

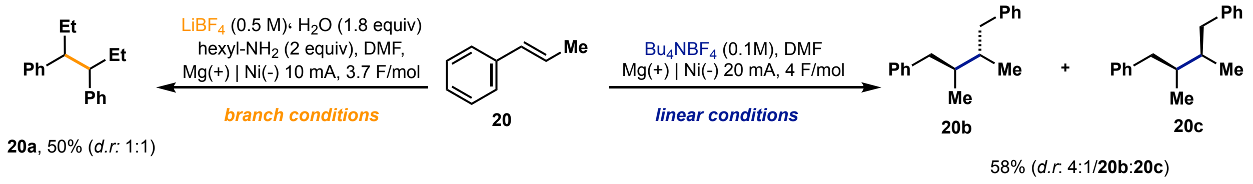

Figure S19: Reaction outcome of styrene **20** and diastereoselectivity.

For the branch-selective pathway, our mechanistic data strongly support a radical–radical coupling process. Importantly, the observed d.r. ≈ 1:1 is also consistent with this proposal, since radical–radical couplings are typically effectively barrierless and therefore provide little intrinsic basis for stereochemical discrimination. If the linear-selective reaction proceeded through the same radical–radical coupling manifold, a similar statistical diastereochemical outcome would be expected.

To probe the origin of this diastereoselectivity, we examined which diastereomer predominates. Analysis of the product mixture (chiral GC; Figure 20) shows that **20b** (racemic) is the major product, whereas **20c** (meso) is minor. This trend is consistent with kinetic stereocontrol at the C–C bond-forming step, where the lower-energy transition state, presumably with reduced steric repulsion, forms more readily. We therefore computed the free-energy profiles for the radical addition pathways leading to each diastereomer, Figure 21. In contrast to radical–radical coupling, both trajectories proceed through well-defined transition states. The computed activation barriers are close but distinct: formation of **B** (intermediate of **20b**) proceeds with a barrier of $\Delta G^{\ddagger} = 17.8$ kcal/mol, whereas formation of **A** (intermediate of **20c**) requires $\Delta G^{\ddagger} = 18.2$ kcal/mol. The resulting $\Delta\Delta G^{\ddagger} = 0.4$ kcal/mol corresponds to a predicted ratio of roughly 65:35 at 300 K. While this slightly underestimates the experimentally observed selectivity (80:20), it captures the correct direction and supports a mechanism in which diastereoselectivity arises from a differentiated,

barriered radical-addition step, rather than an unselective, barrierless radical–radical coupling event.

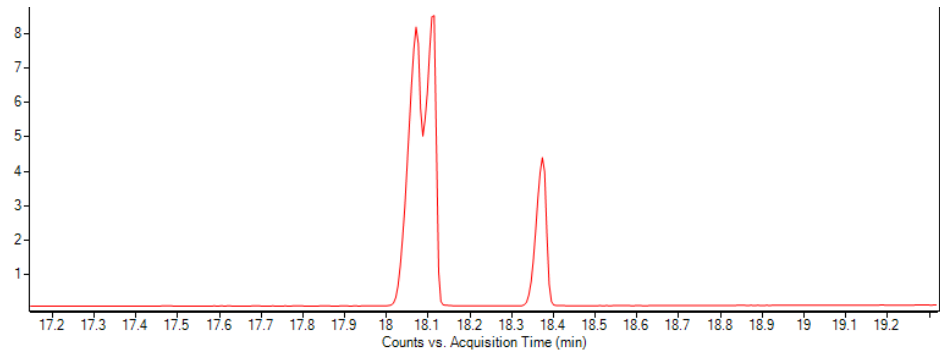

Figure S20: GC trace (chiral column) of **20** linear coupling products, **20b** and **20c**.

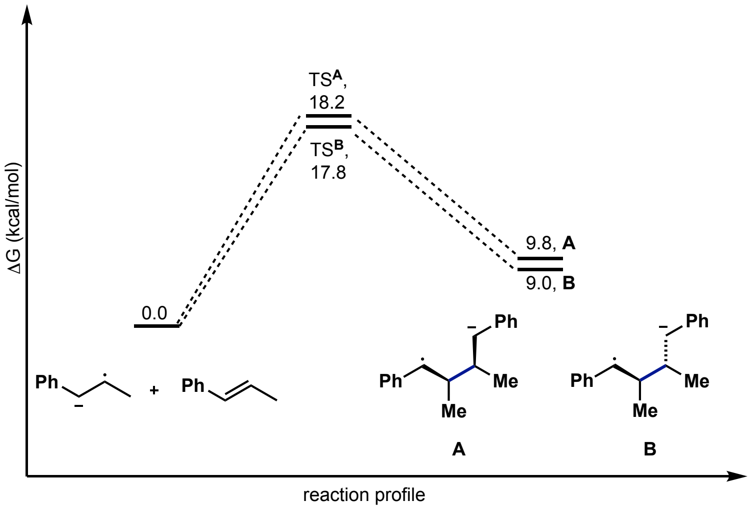

Figure S21: DFT computation of the radical addition pathway of the compound **20** coupling process.

<u>Note</u>: We computed free energy profiles for both pathways of each set of reaction conditions using ORCA 6.1.0 at the ωB97X-D level with implicit DMF solvent included from the conductor-like polarizable continuum model (CPCM). Here, optimized geometries and vibrational frequencies were calculated using the cc-pVTZ basis, while electronic energies were refined to the complete basis set (CBS) limit using a two-point (TZ, QZ) extrapolation. For each reaction, the change in the Gibbs free energy along the reaction coordinate was then determined at 300 K.

6. NMR, GC, HRMS spectra

^{1}H NMR (300 MHz, $CDCl_3$) of compound **1a**

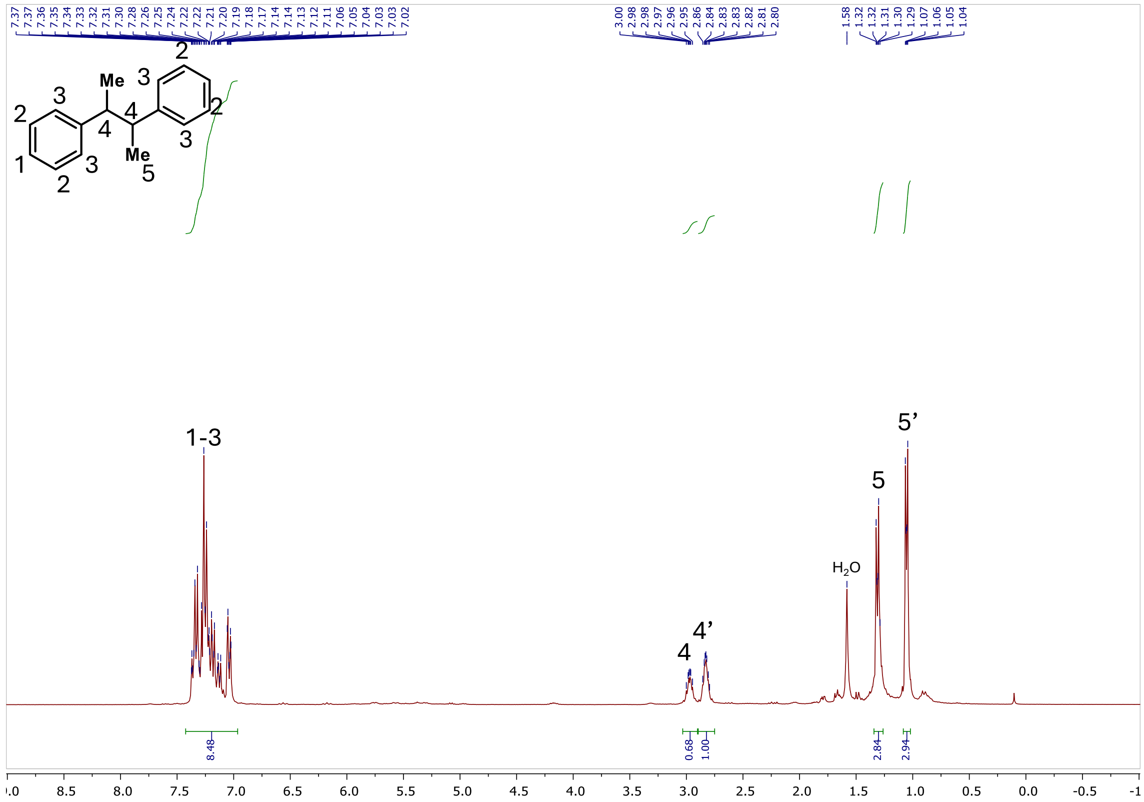

^{13}C NMR (75 MHz, $CDCl_3$) of compound **1a**

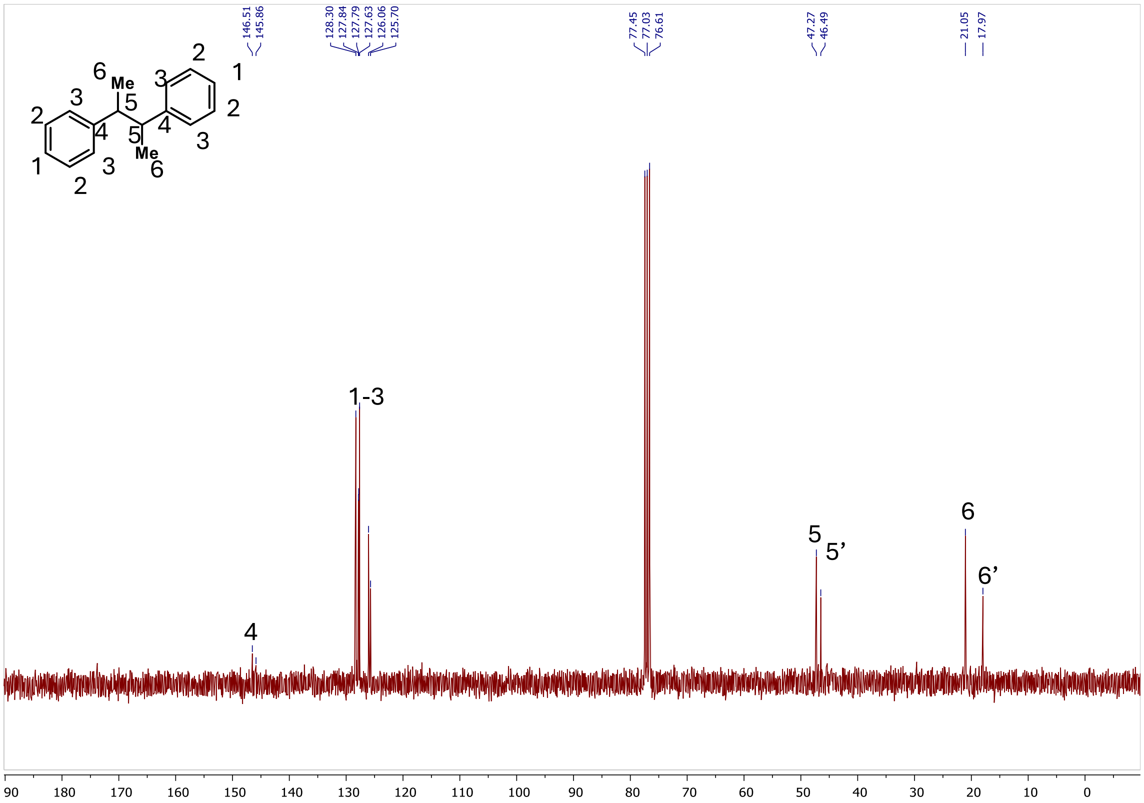

GC-MS (EI) of compound **1a**

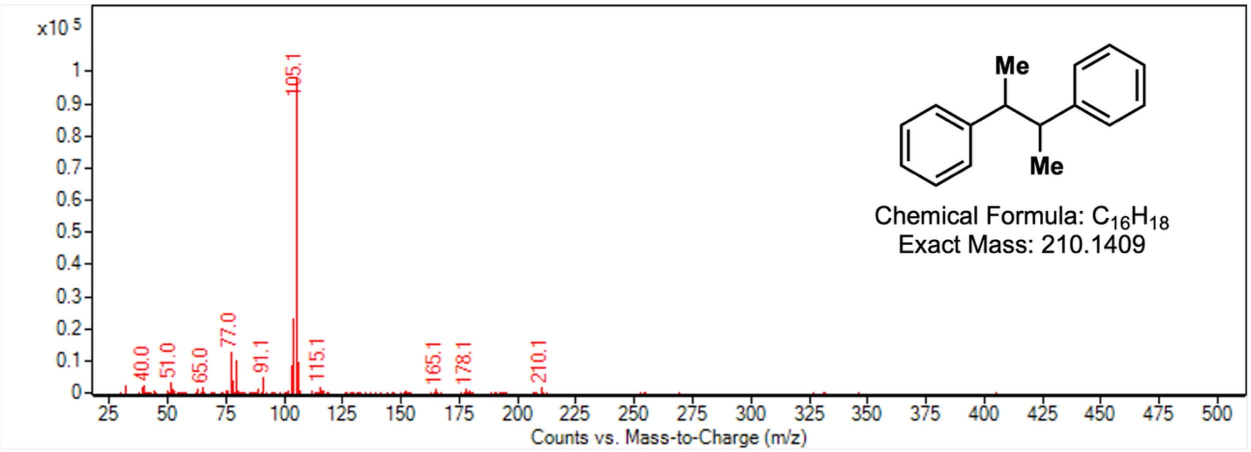

^{1}H NMR (300 MHz, $CDCl_3$) of compound **1b**

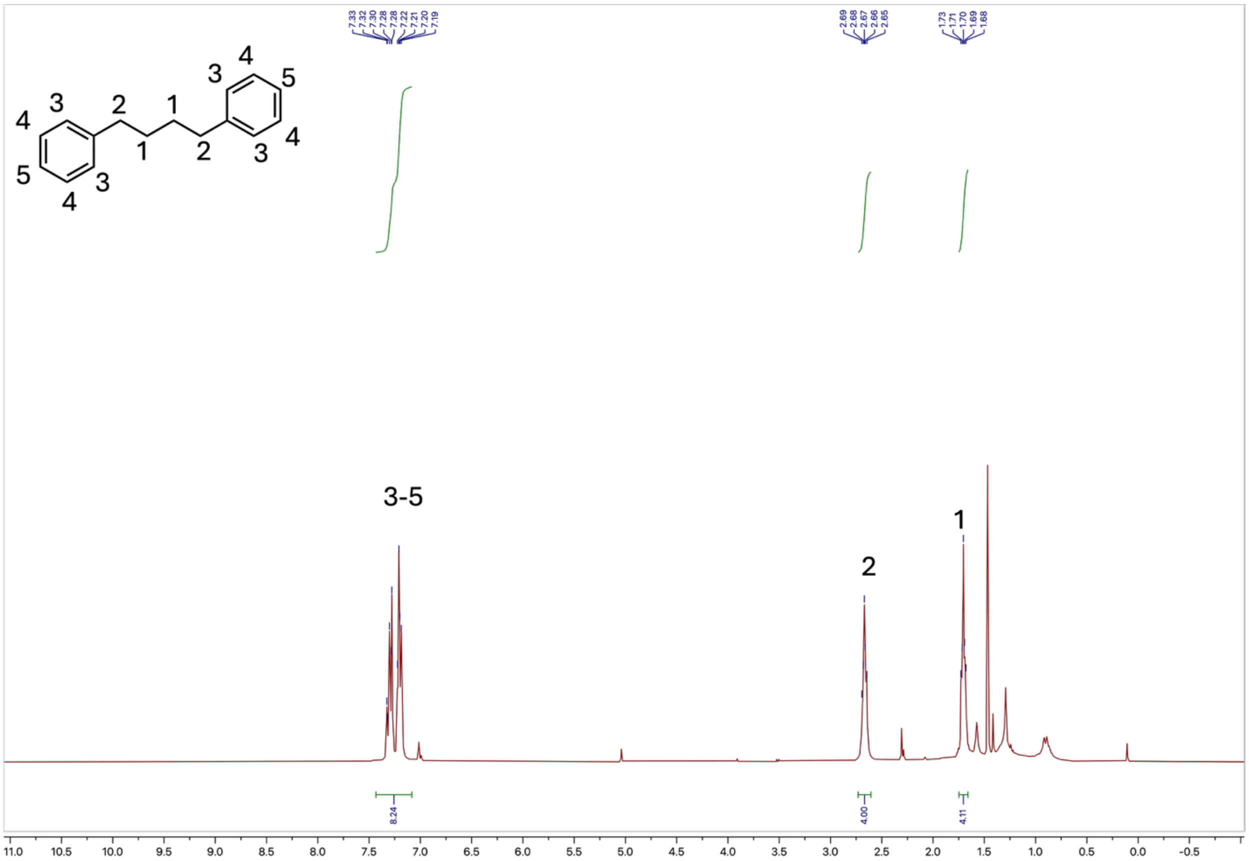

^{13}C NMR (126 MHz, $CDCl_3$) of compound **1b**

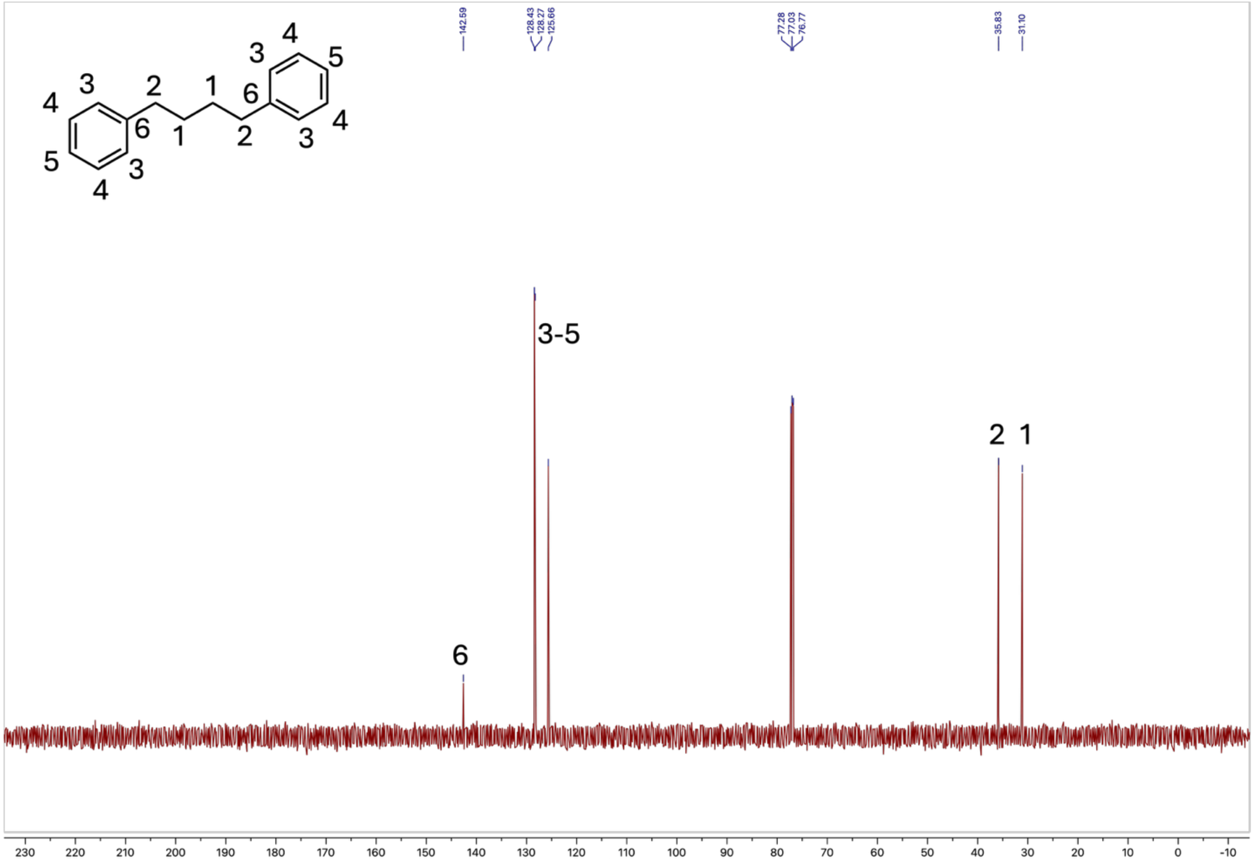

GC-MS (EI) of compound **1b**

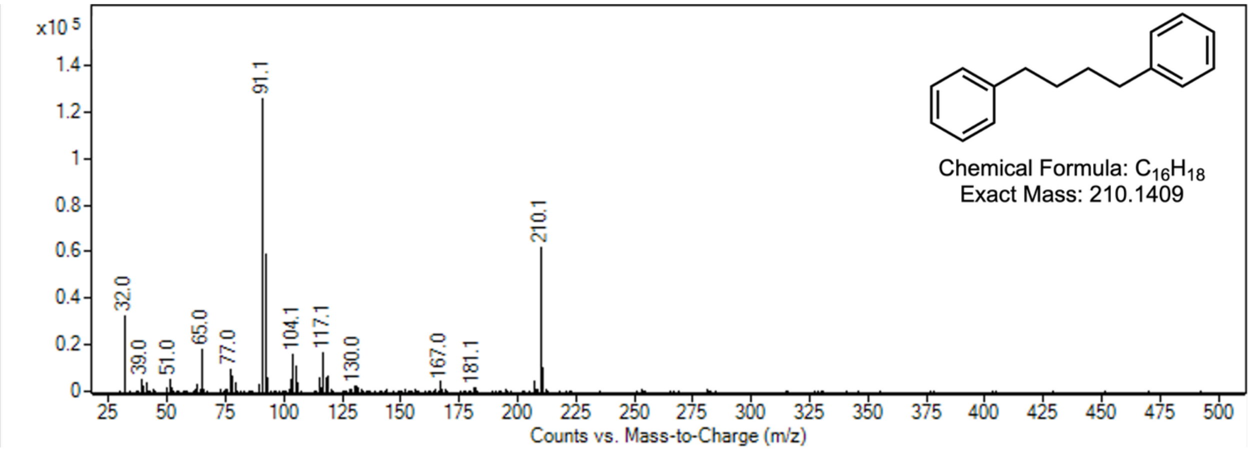

GC/MS crude comparison of **1a** and **1b** – branched crude (bottom) vs linear crude (top)

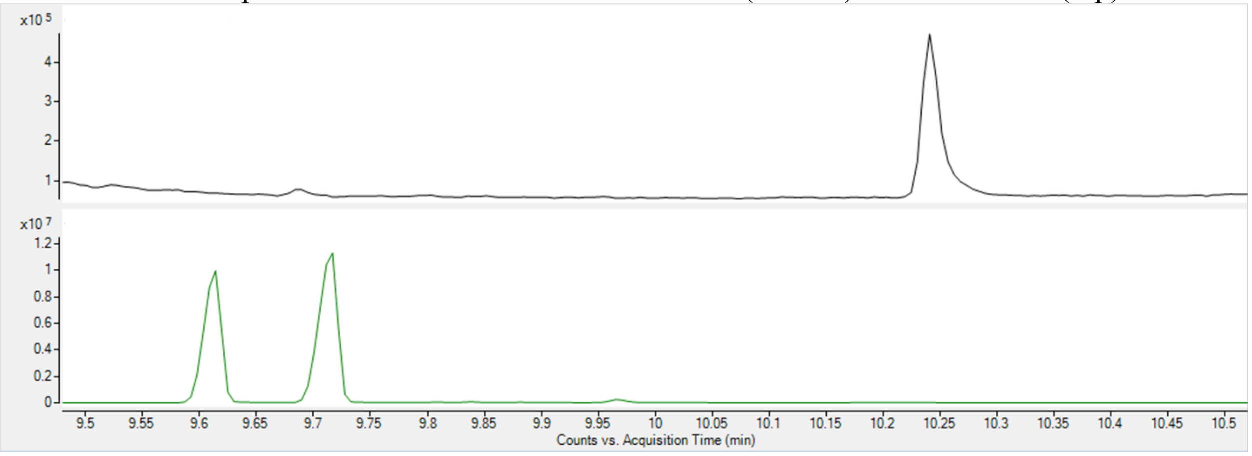

^{1}H NMR (400 MHz, $CDCl_3$) of compound **4a**

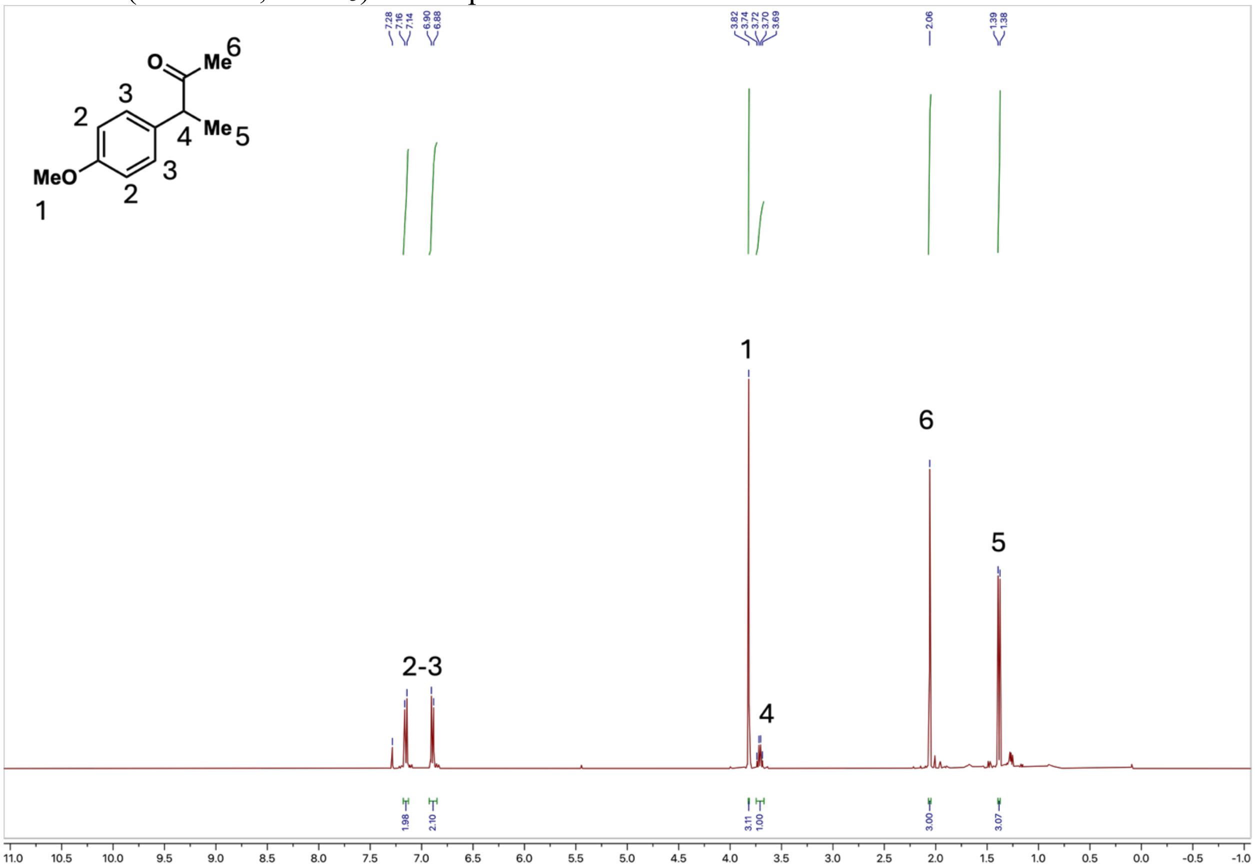

^{13}C NMR (151 MHz, $CDCl_3$) of compound **4a**

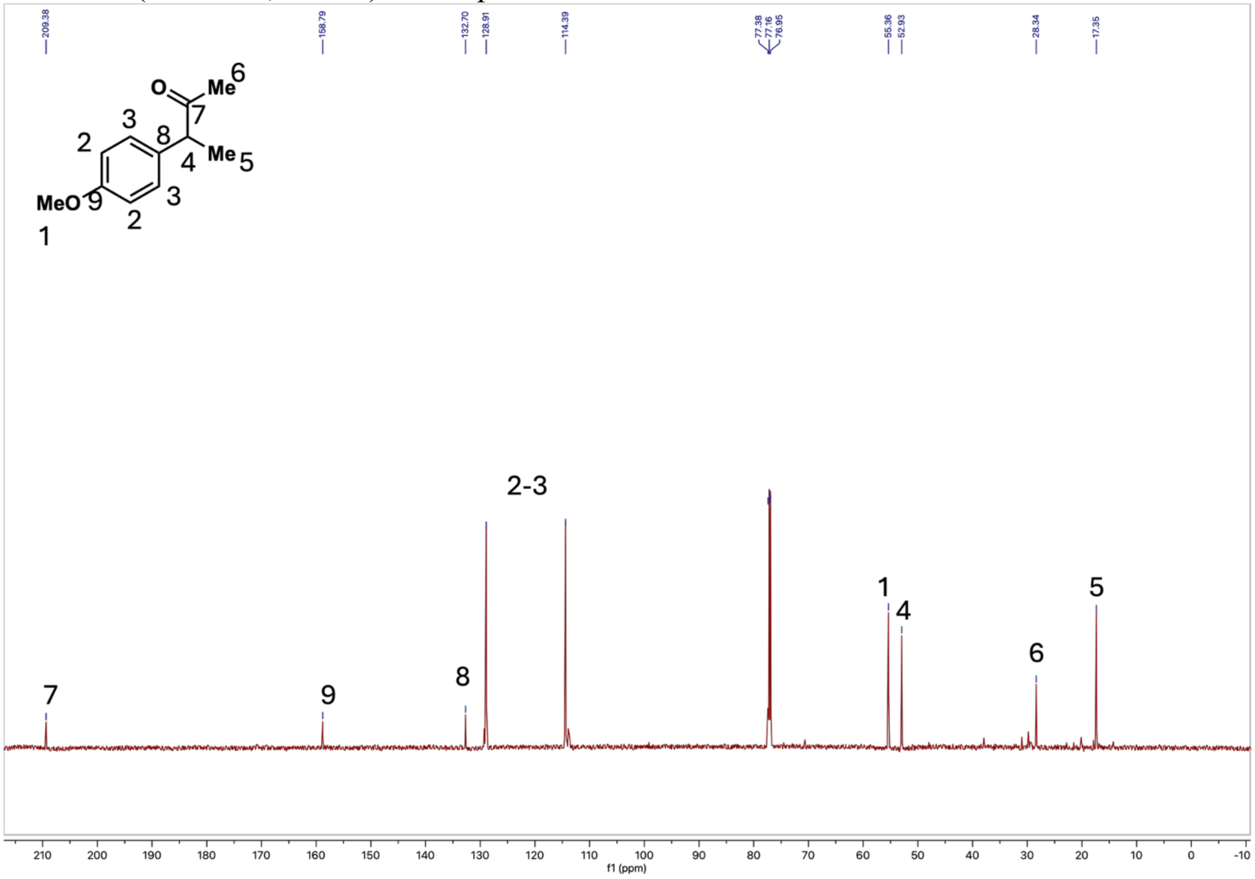

GC-MS (EI) of compound **4a**

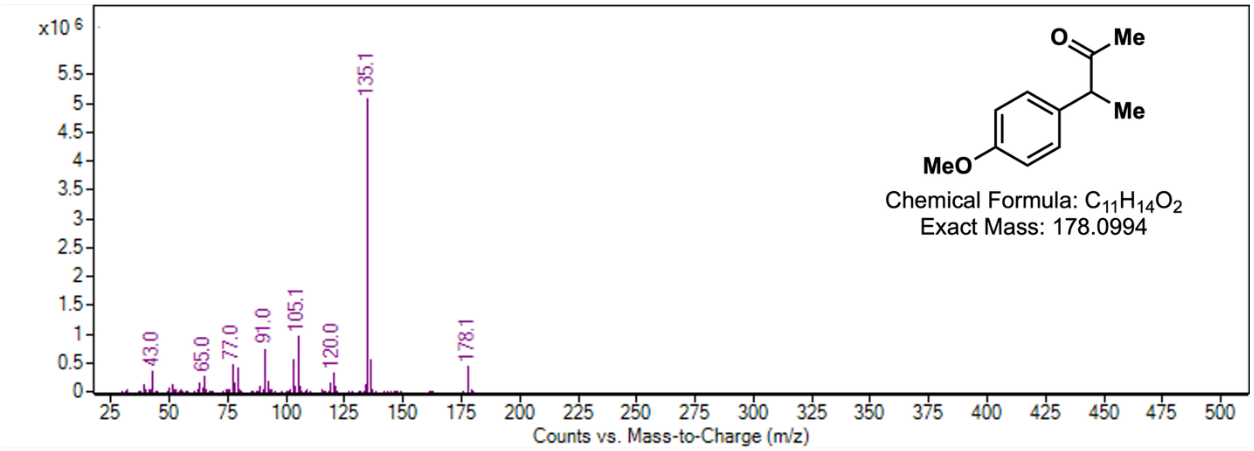

^{1}H NMR (400 MHz, $CDCl_3$) of compound **4b**

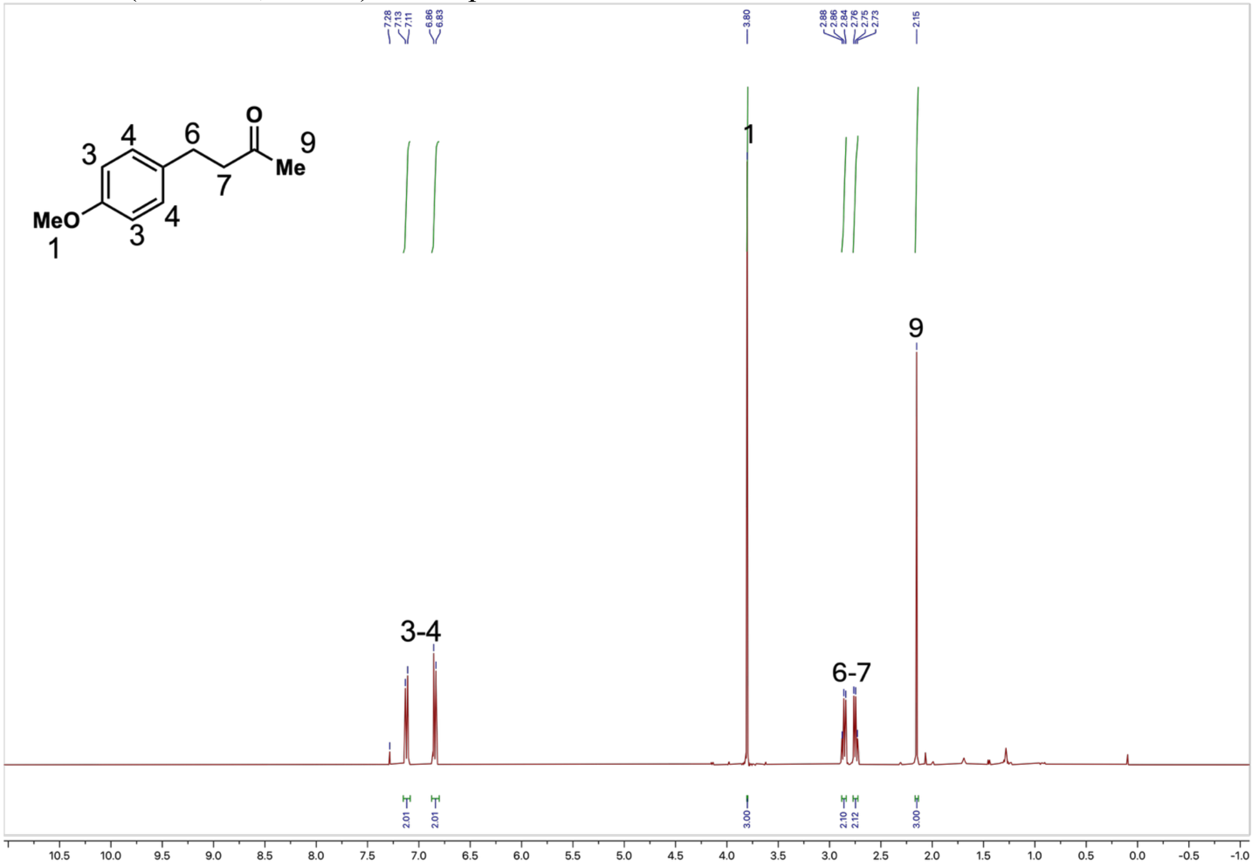

^{13}C NMR (151 MHz, $CDCl_3$) of compound **4b**

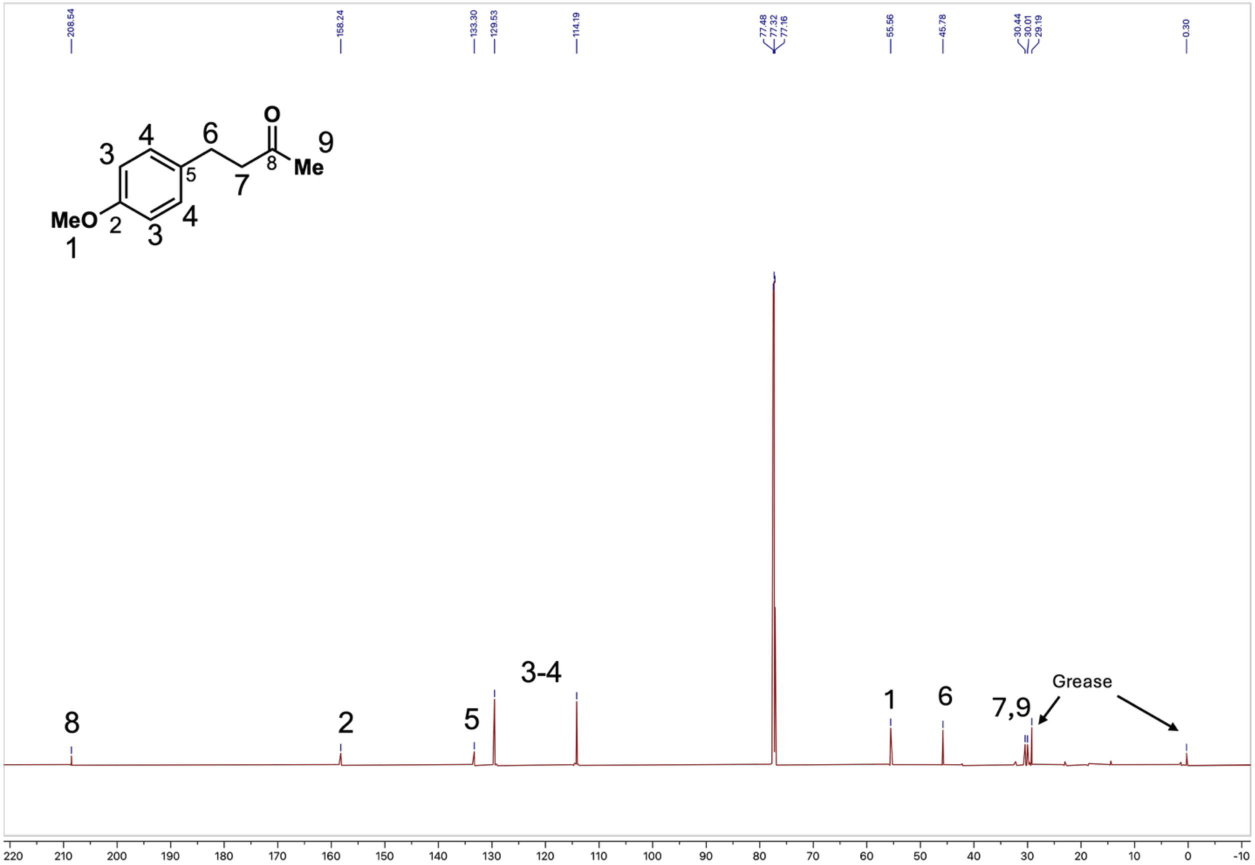

GC-MS (EI) of compound **4b**

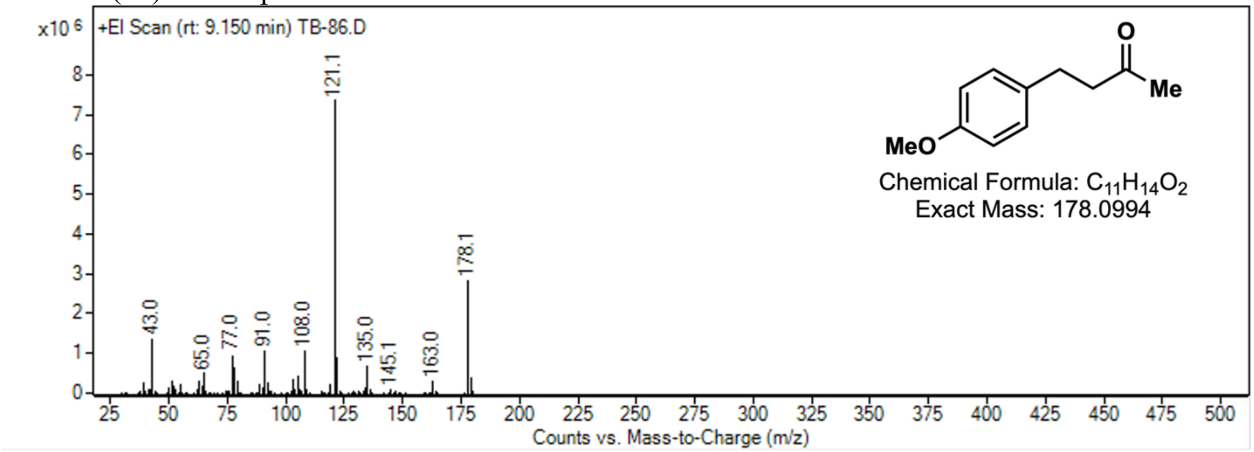

GC comparison between alpha (bottom) vs beta (top) hydrocarbonylation

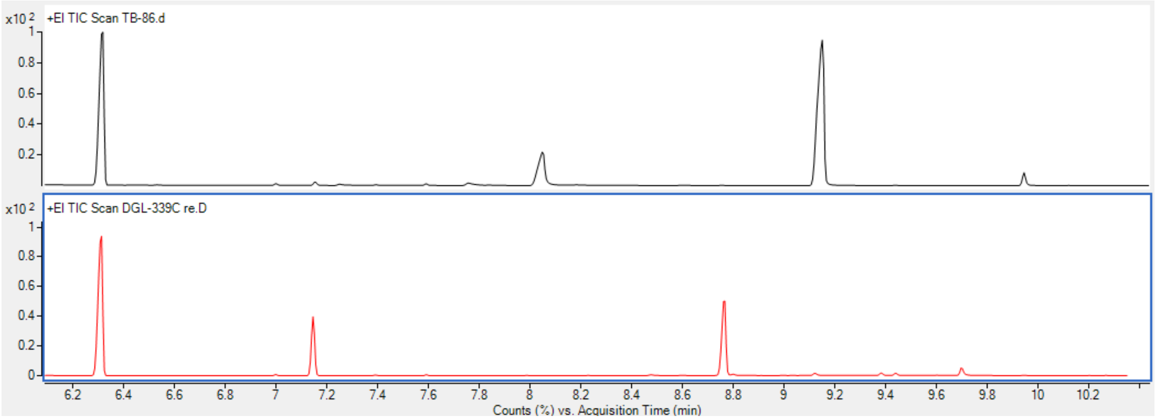

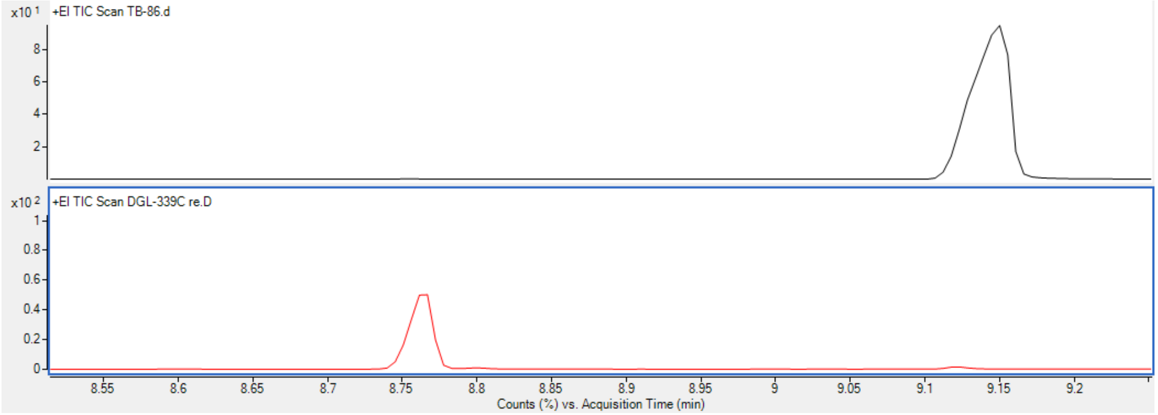

^{1}H NMR (300 MHz, $CDCl_3$) of compound **4c**

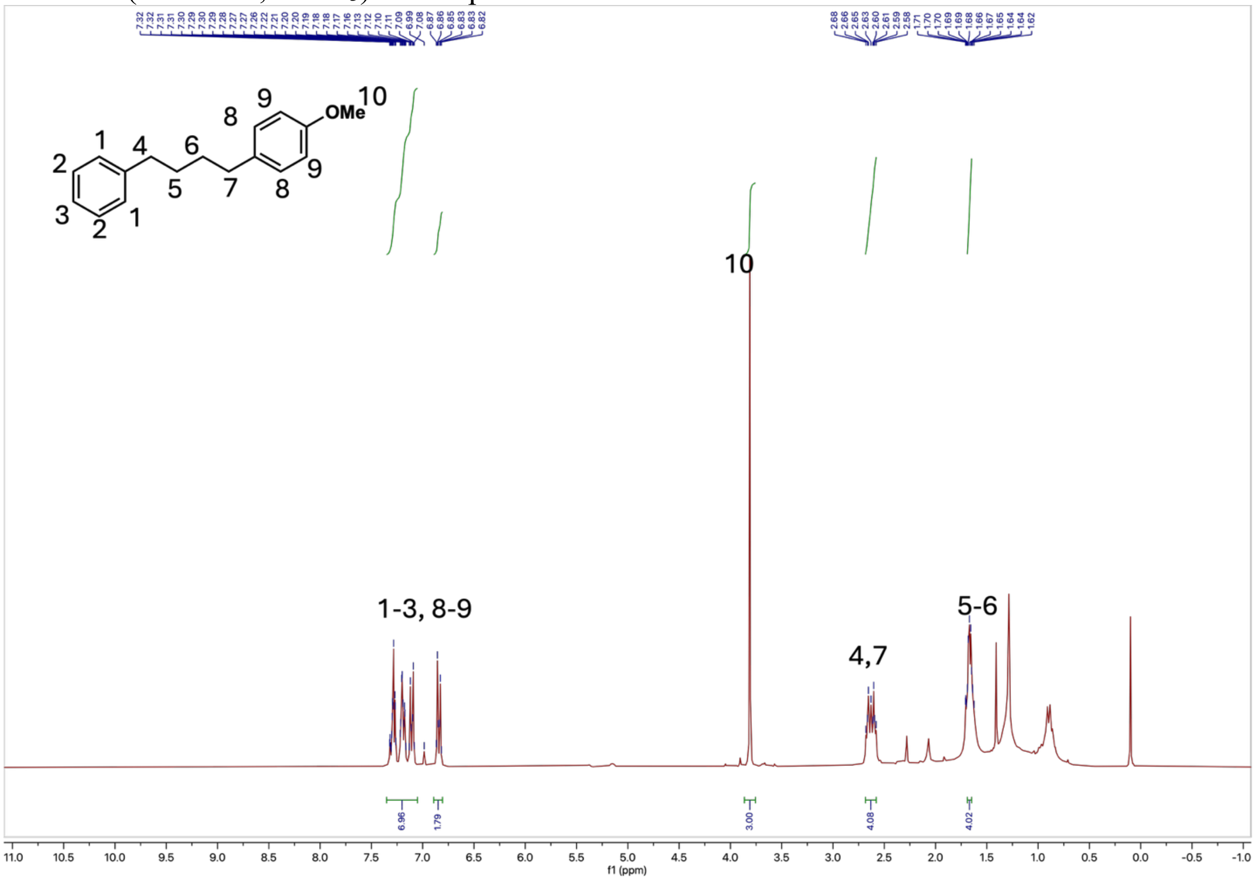

^{13}C NMR (151 MHz, $CDCl_3$) of compound **4c**

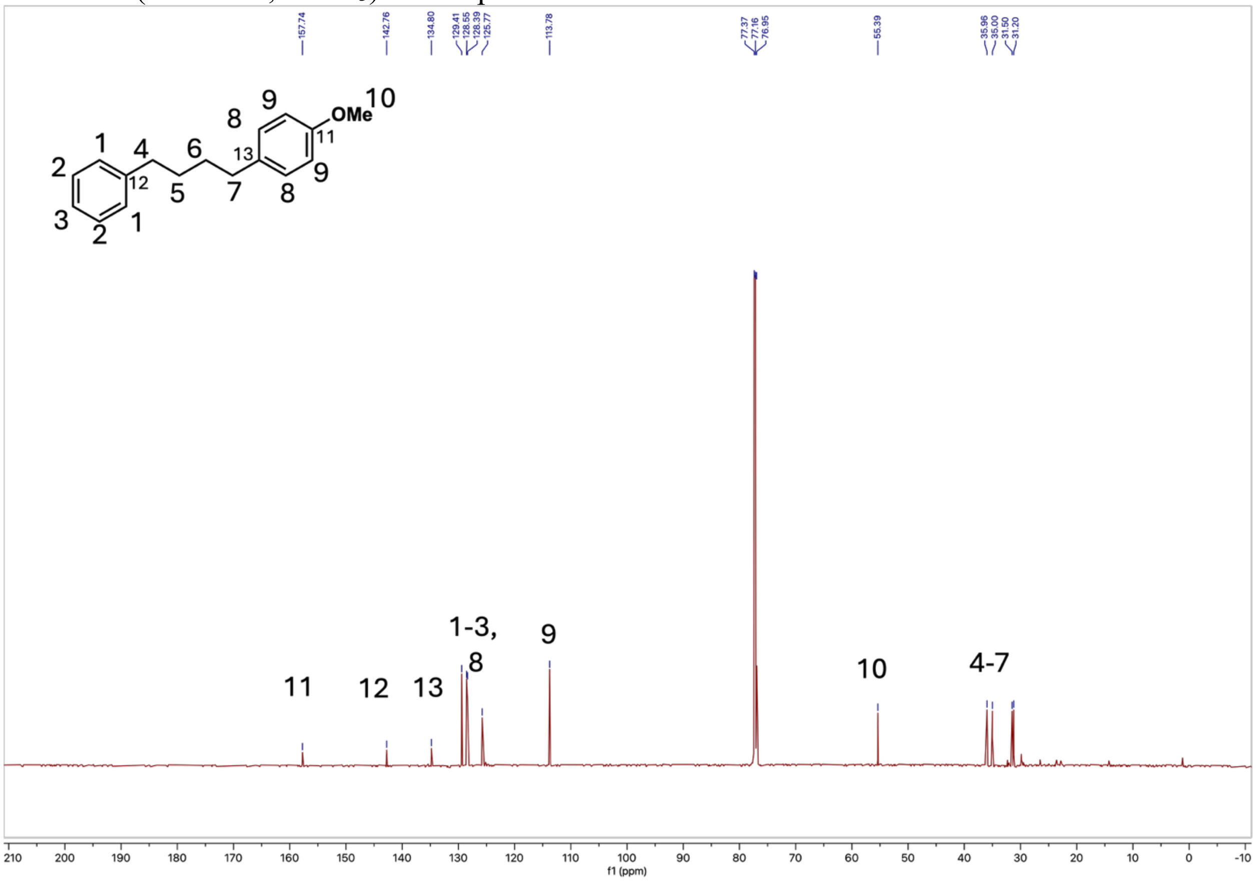

HRMS (ASAP-TOF) of compound **4c**

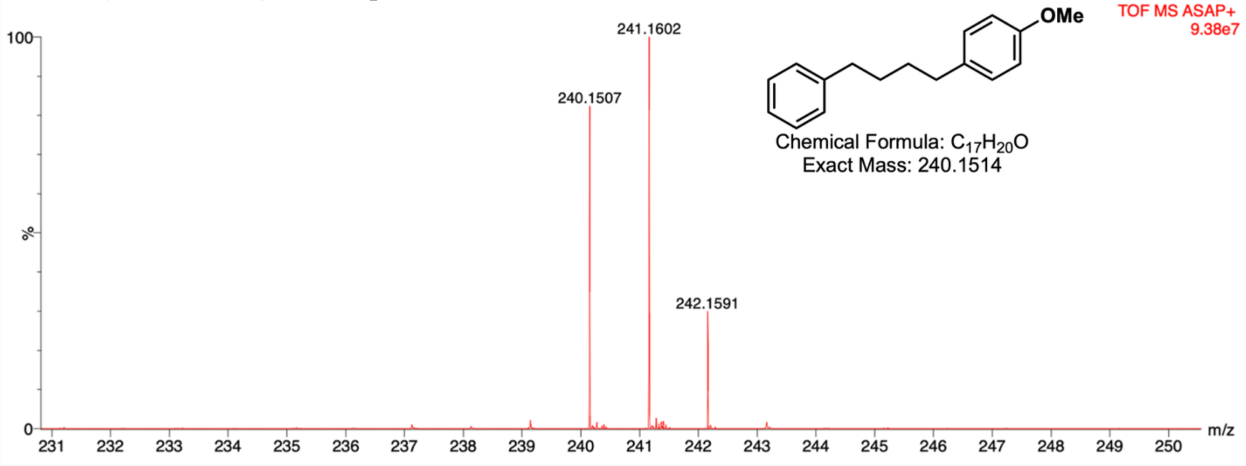

^{1}H NMR (600 MHz, $CDCl_3$) of compound **4e**

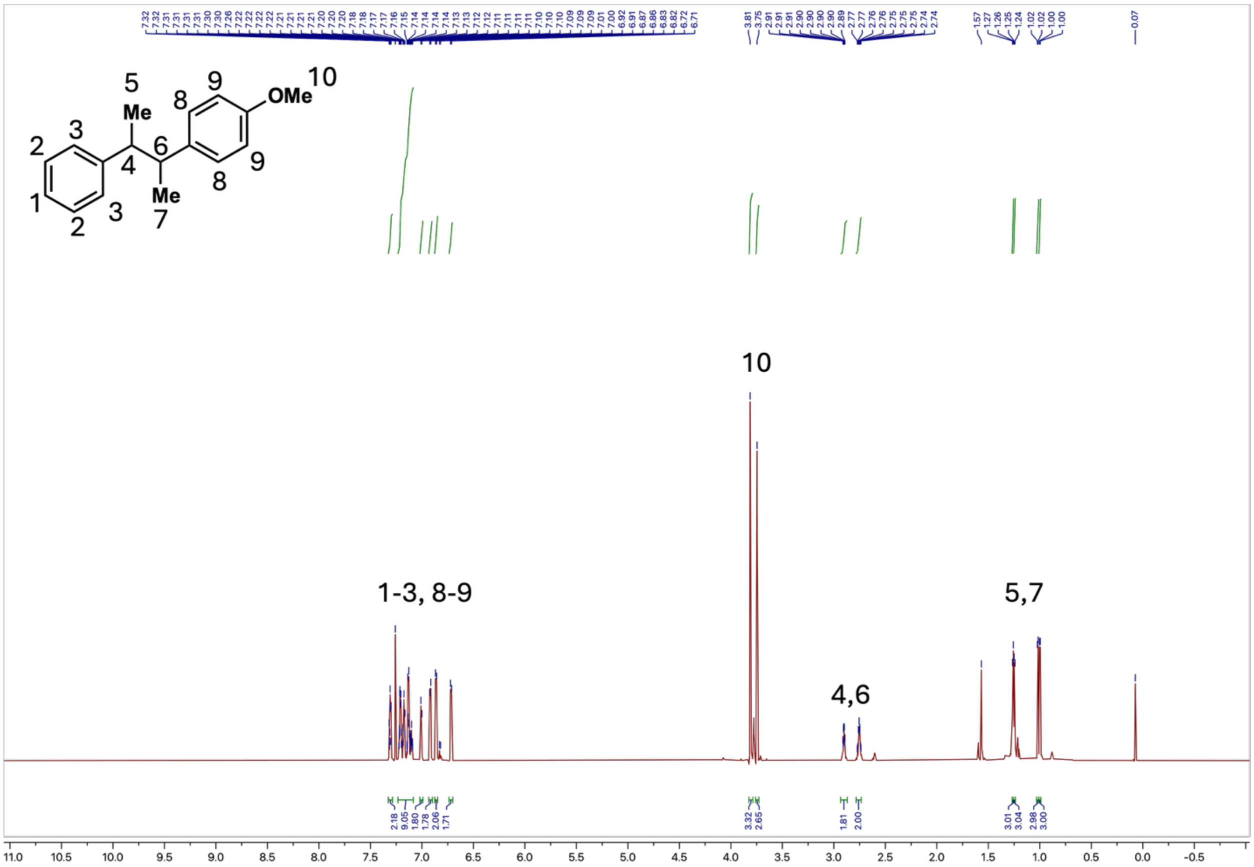

^{13}C NMR (151 MHz, $CDCl_3$) of compound **4e**

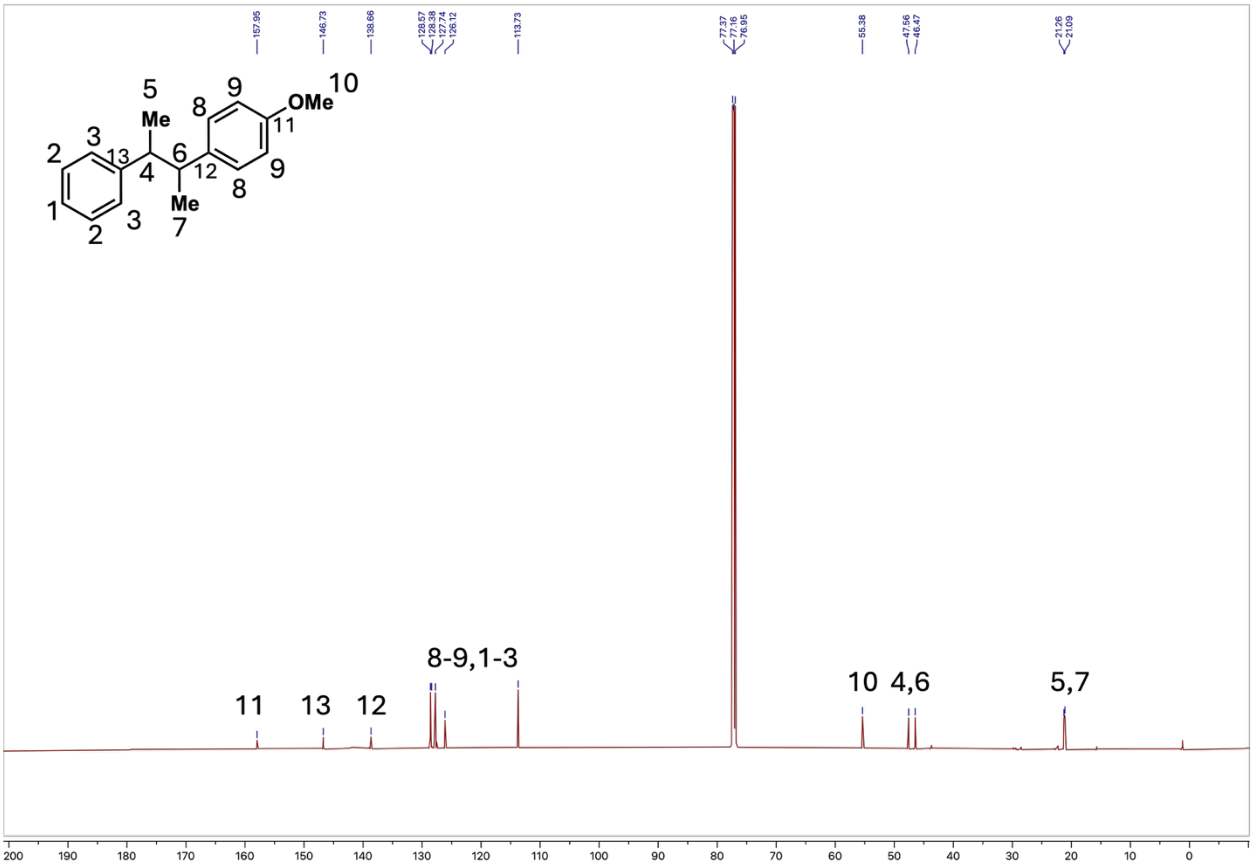

GC-MS (EI) of compound **4e**

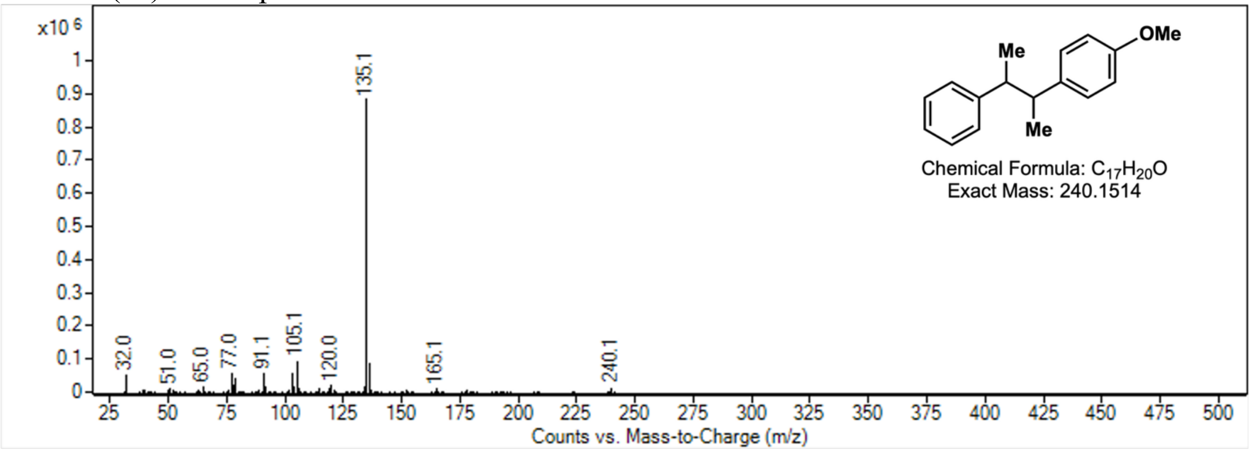

GC/MS comparison linear (top) vs branched (bottom)

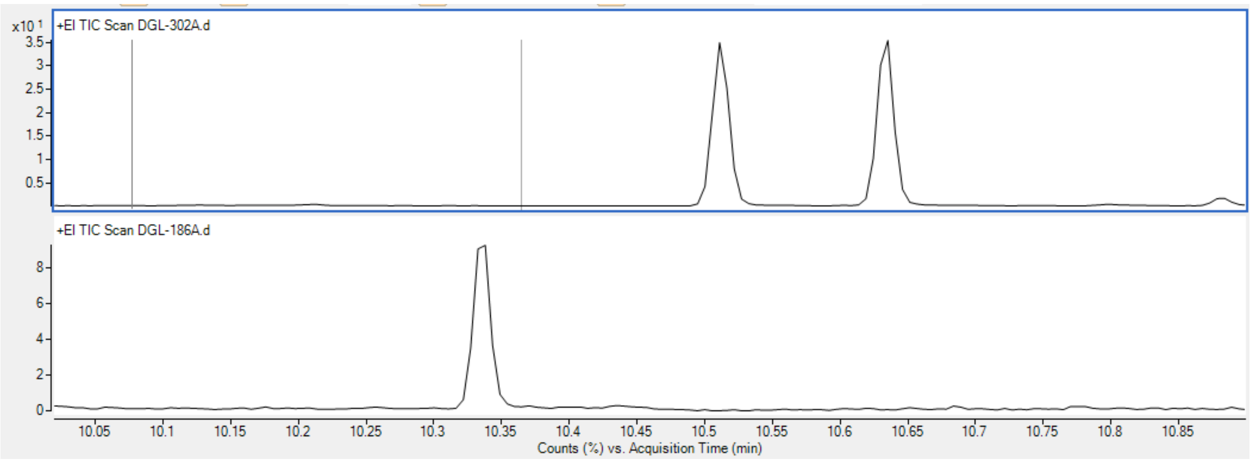

^{1}H NMR (600 MHz, $CDCl_3$) of compound **5a**

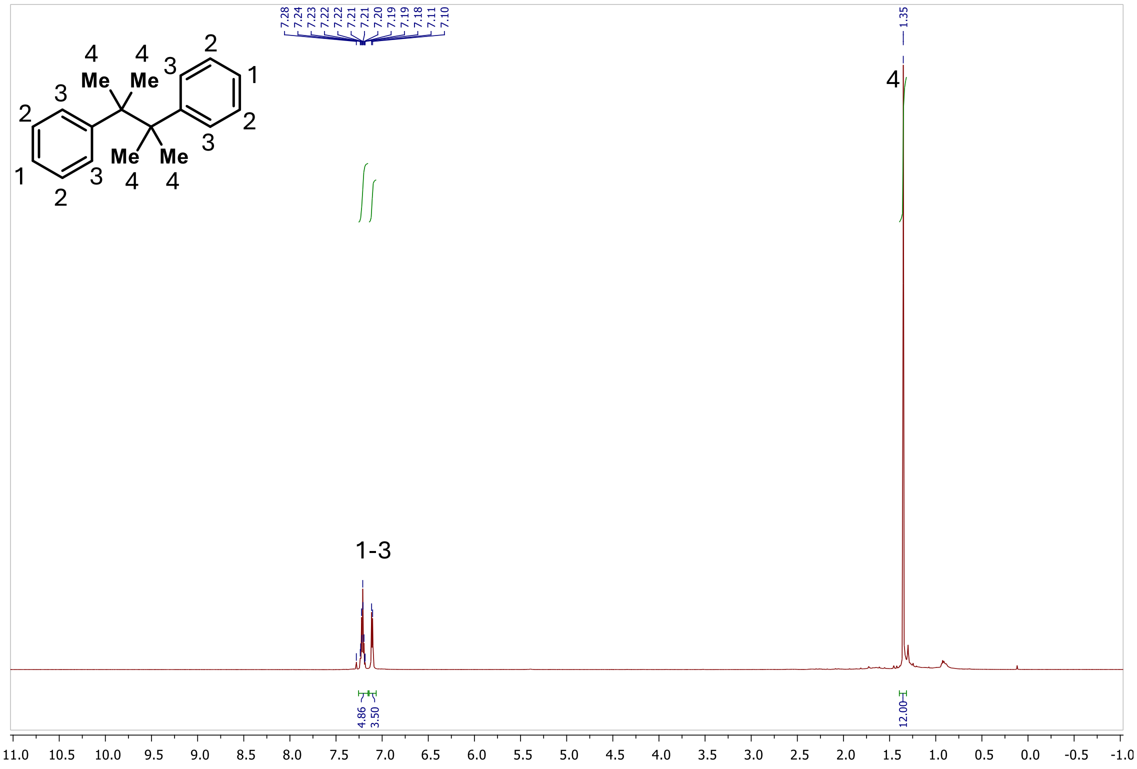

^{13}C NMR (151 MHz, $CDCl_3$) of compound **5a**

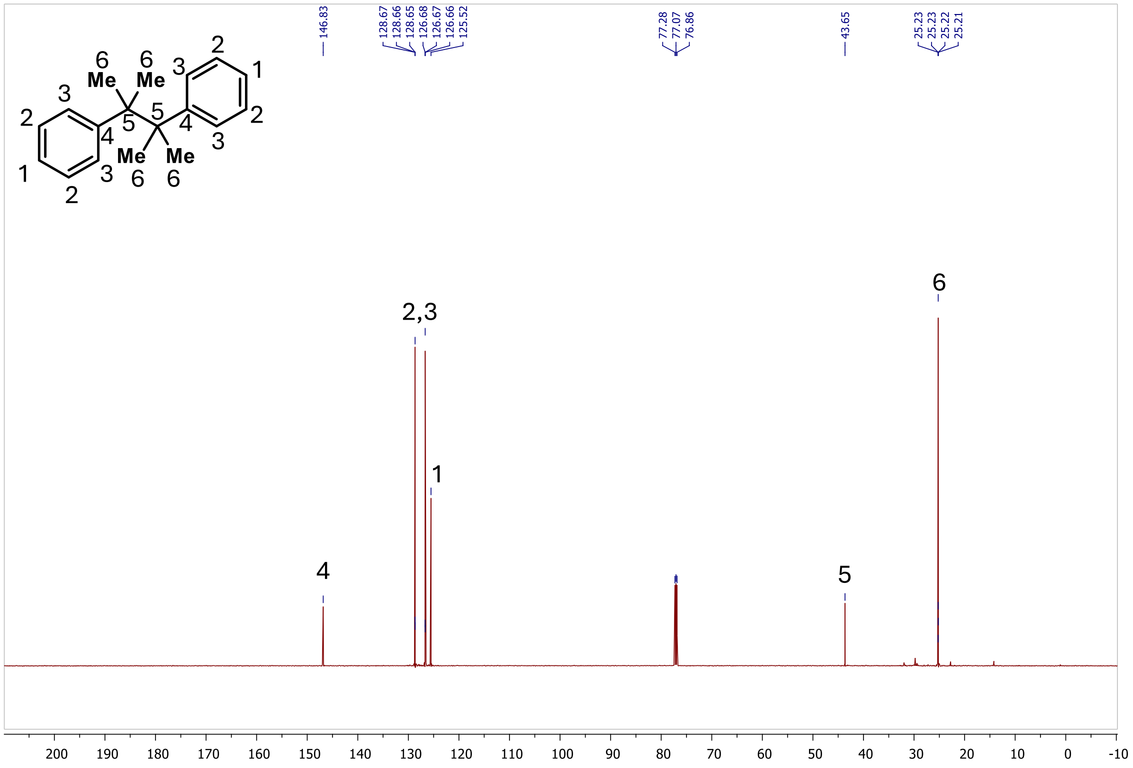

GC-MS (EI) of compound **5a**

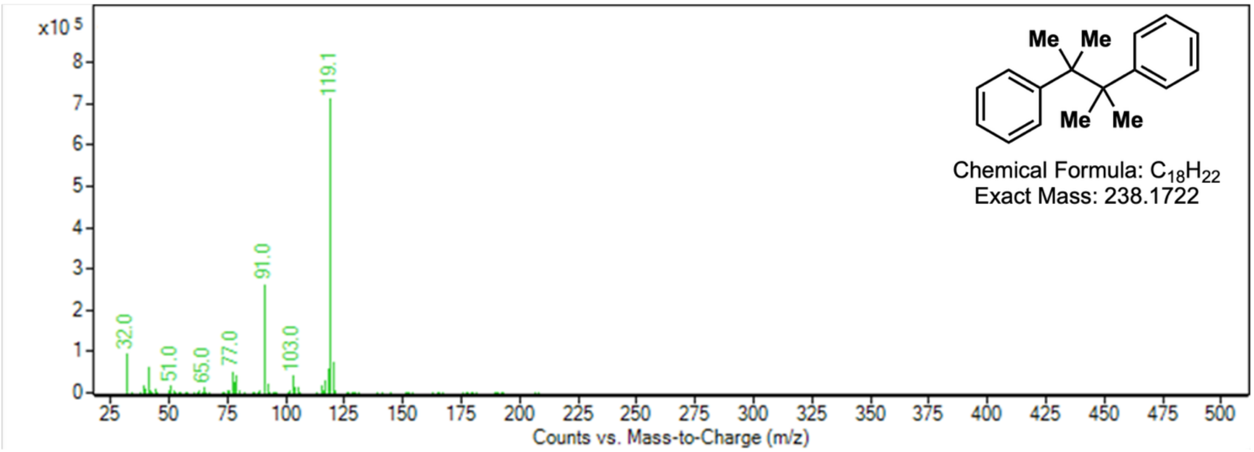

^{1}H NMR (300 MHz, $CDCl_3$) of compound **5b**

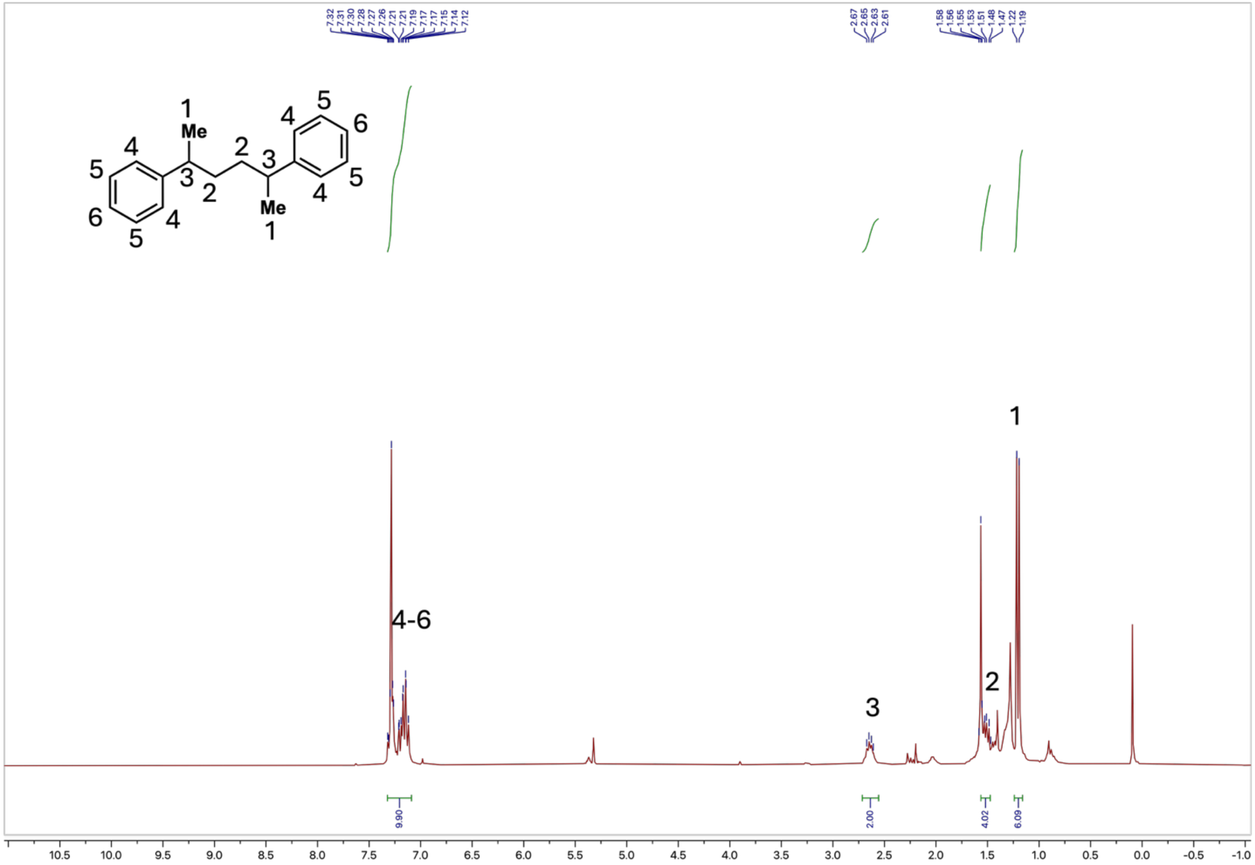

^{13}C NMR (151 MHz, $CDCl_3$) of compound **5b**

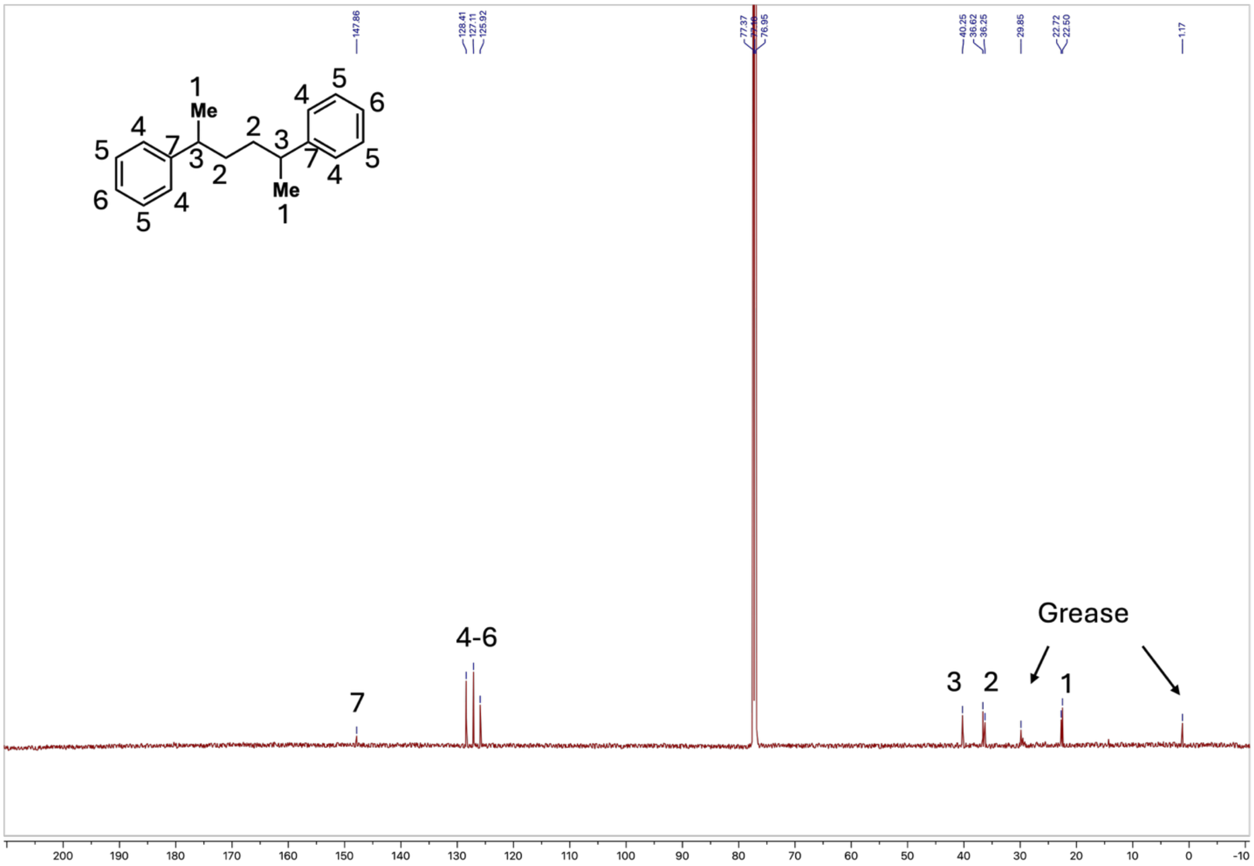

GC-MS (EI) of compound **5b**

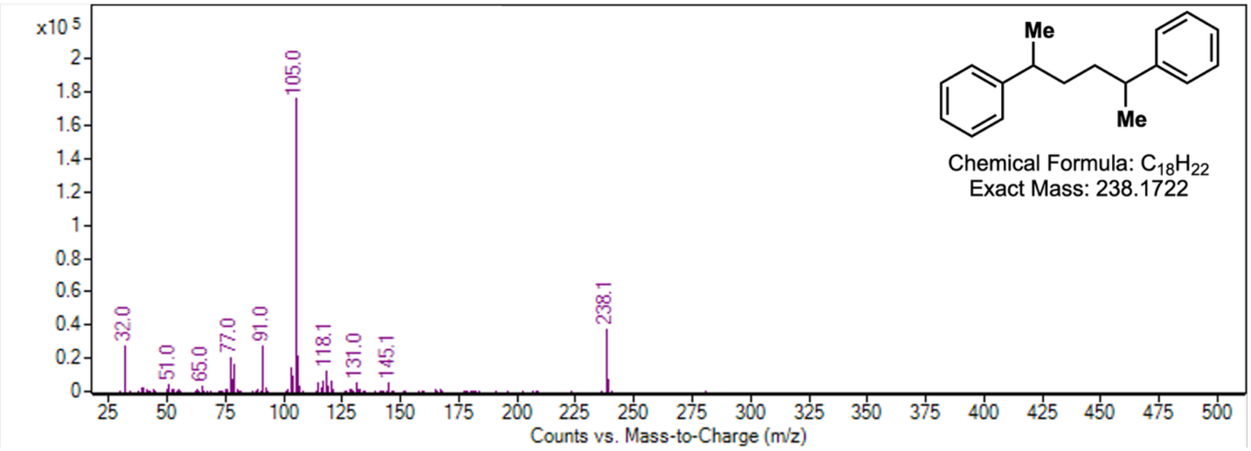

GC/MS crude d.r. of **5b** (1:1)

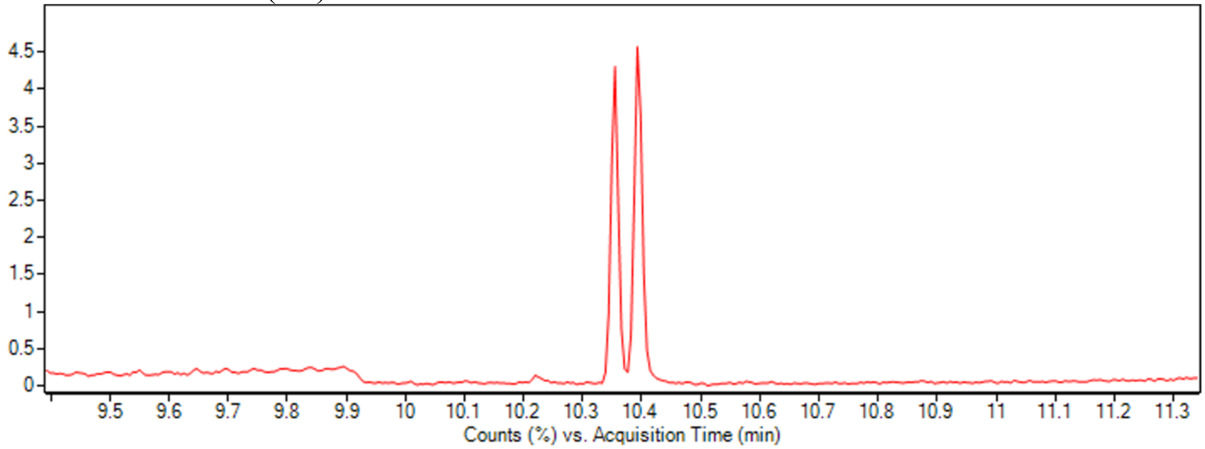

GC/MS crude comparison of **5a** and **5b** – branched crude (bottom) vs linear crude (top)

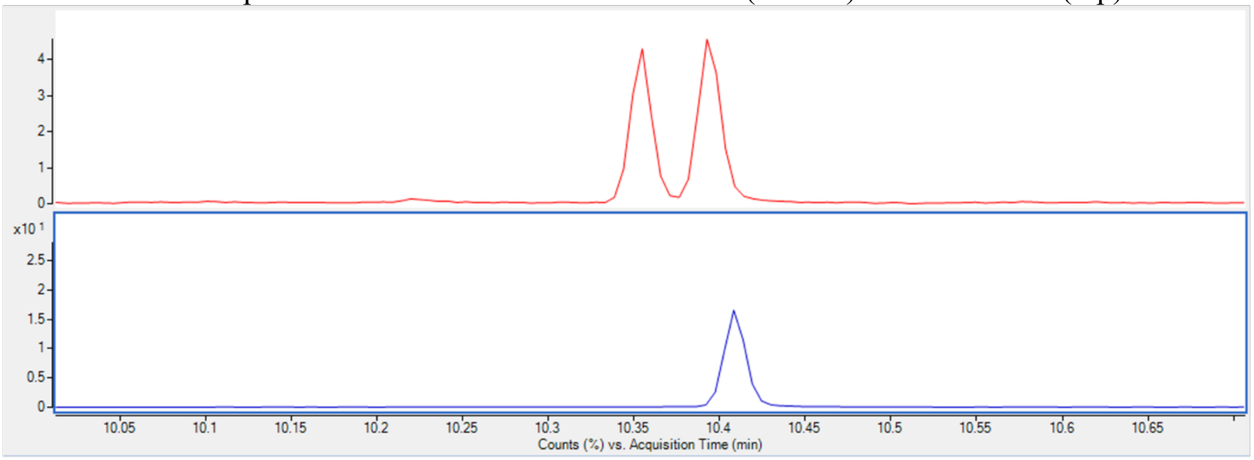

^{1}H NMR (600 MHz, $CDCl_3$) of compound **6a**

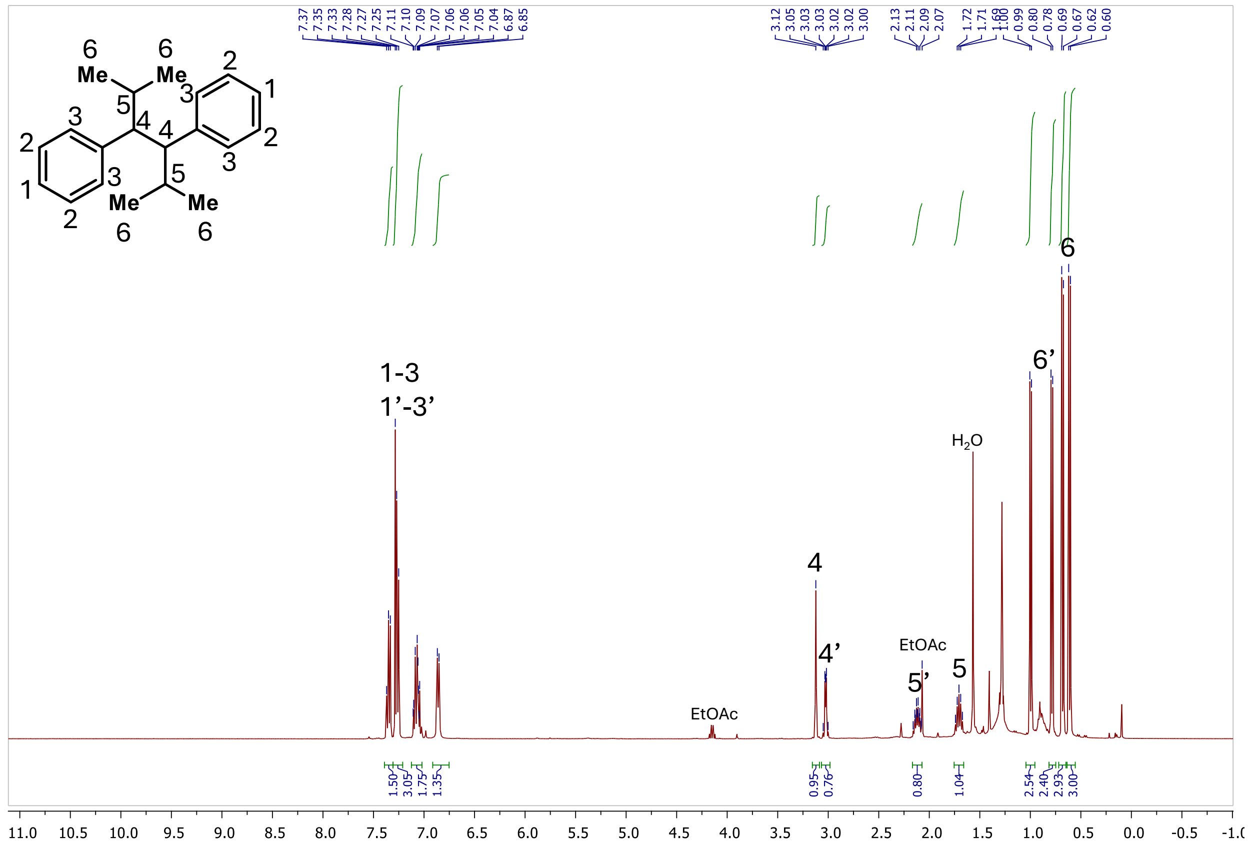

^{13}C NMR (151 MHz, $CDCl_3$) of compound **6a**

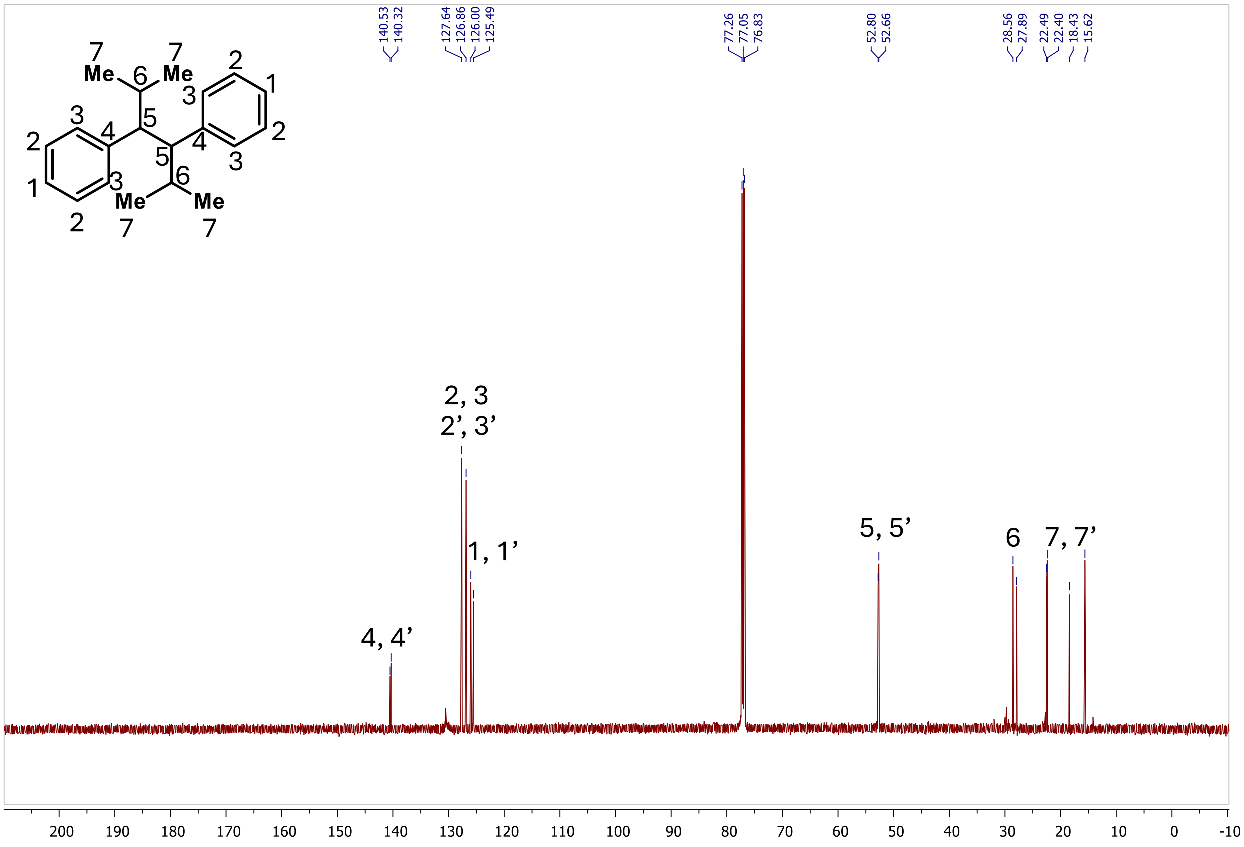

GC-MS (EI) of compound **6a**

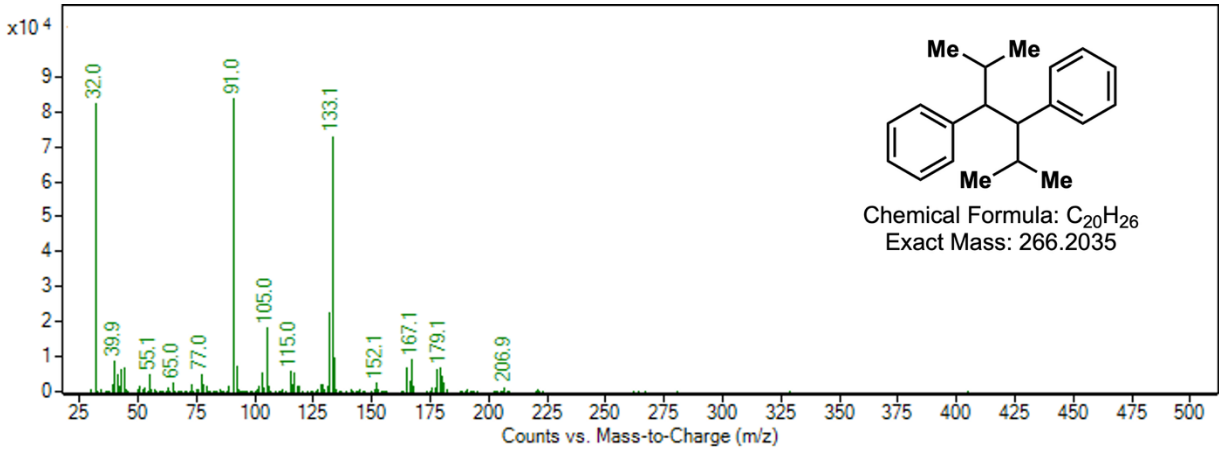

^{1}H NMR (400 MHz, $CDCl_3$) of compound **6b**

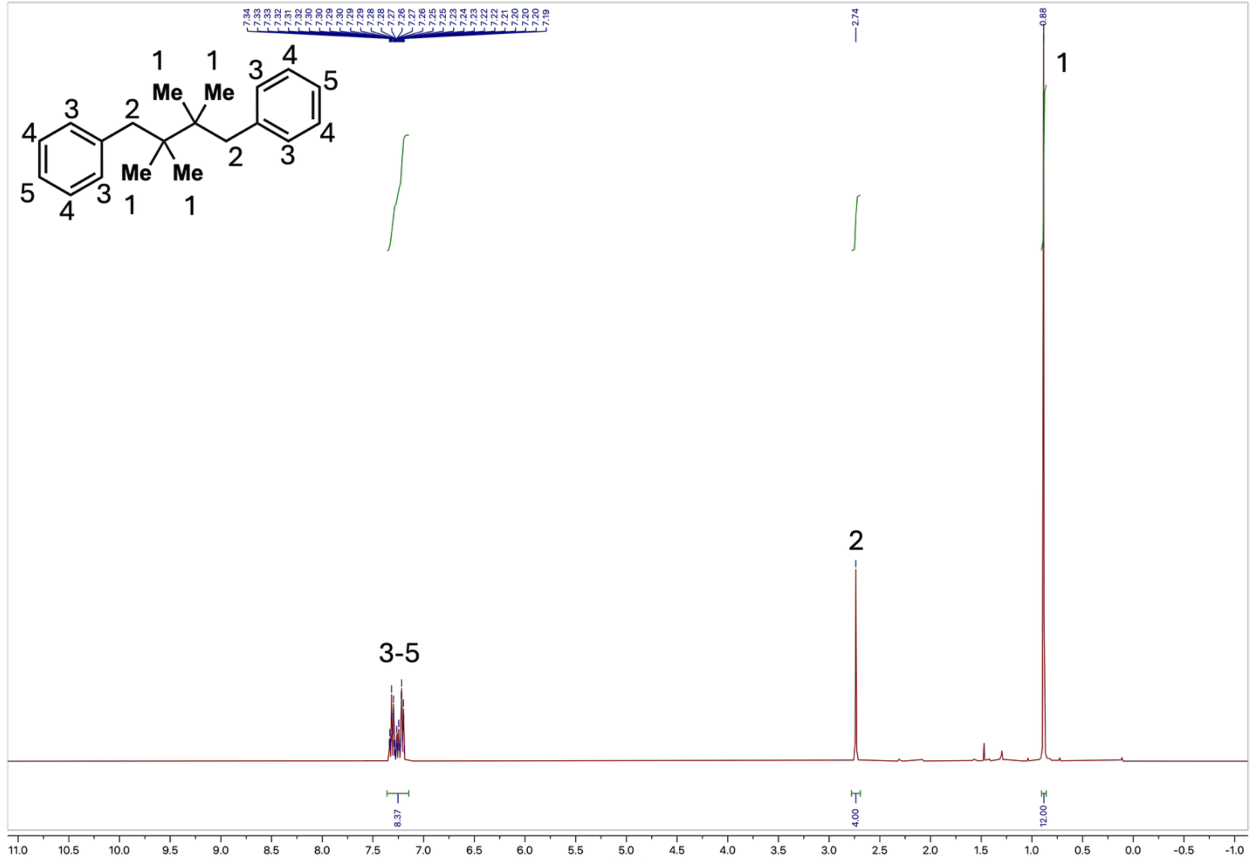

^{13}C NMR (101 MHz, $CDCl_3$) of compound **6b**

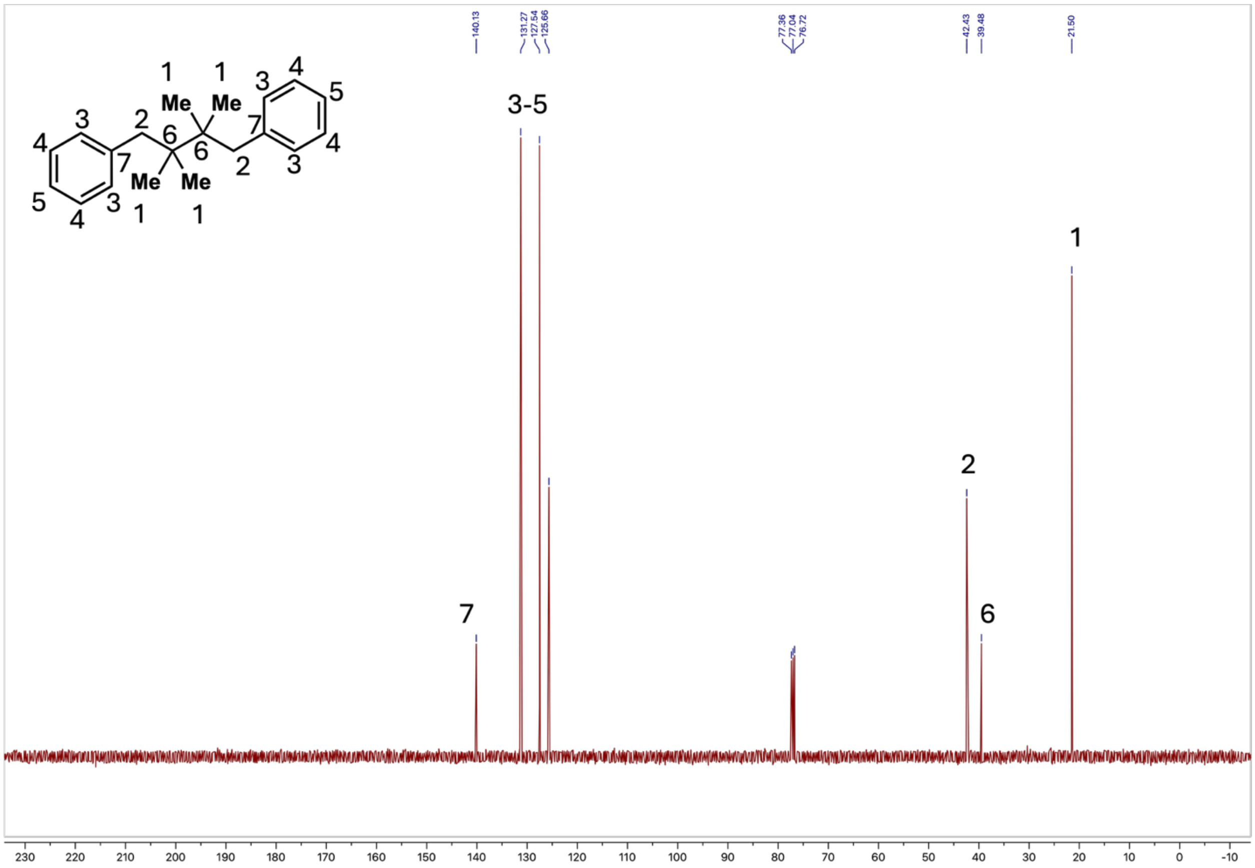

GC-MS (EI) of compound **6b**

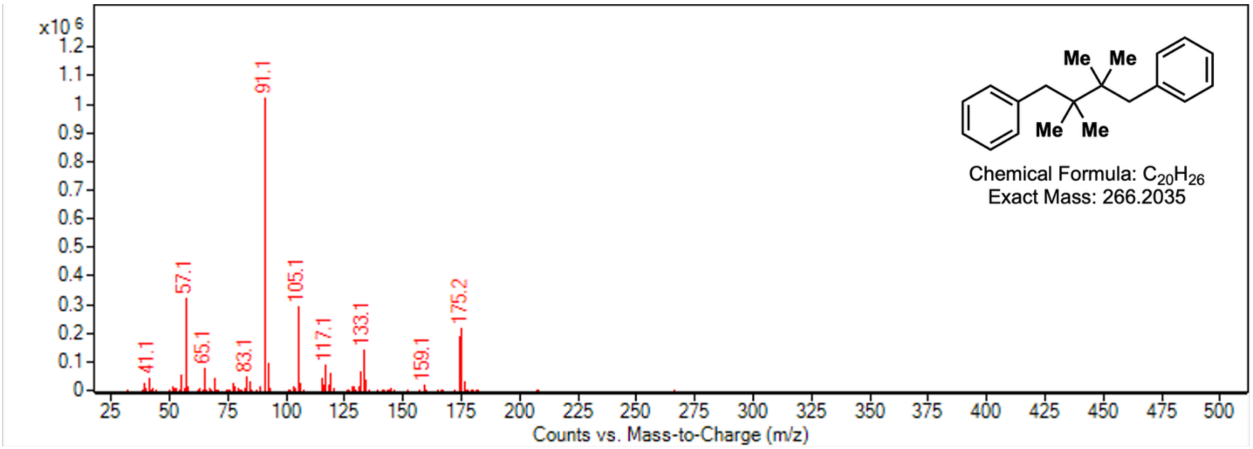

GC/MS crude comparison of **6a** and **6b** – branched crude **6a** (bottom) vs linear crude **6b** (top)

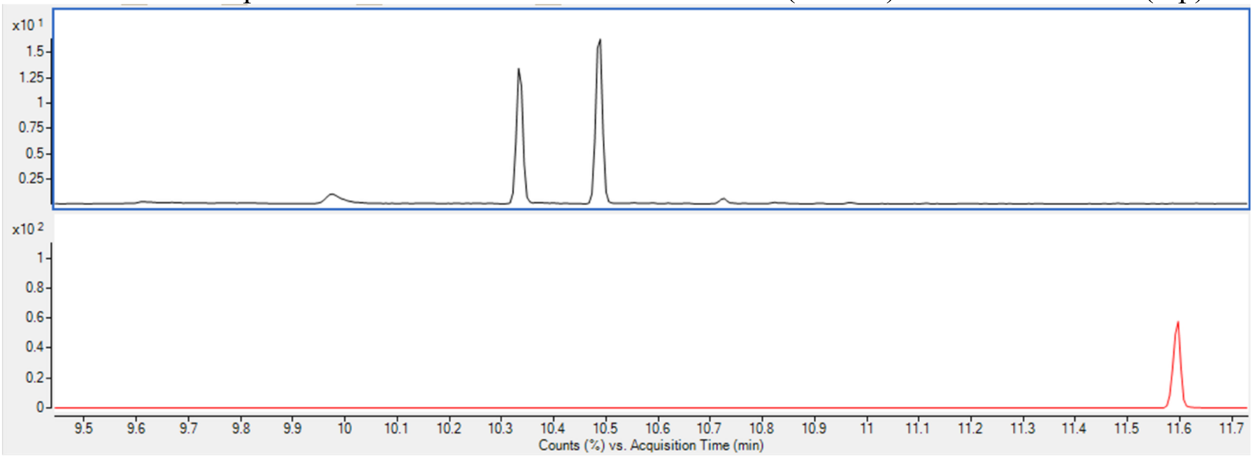

^{1}H NMR (600 MHz, $CDCl_3$) of compound **7a**

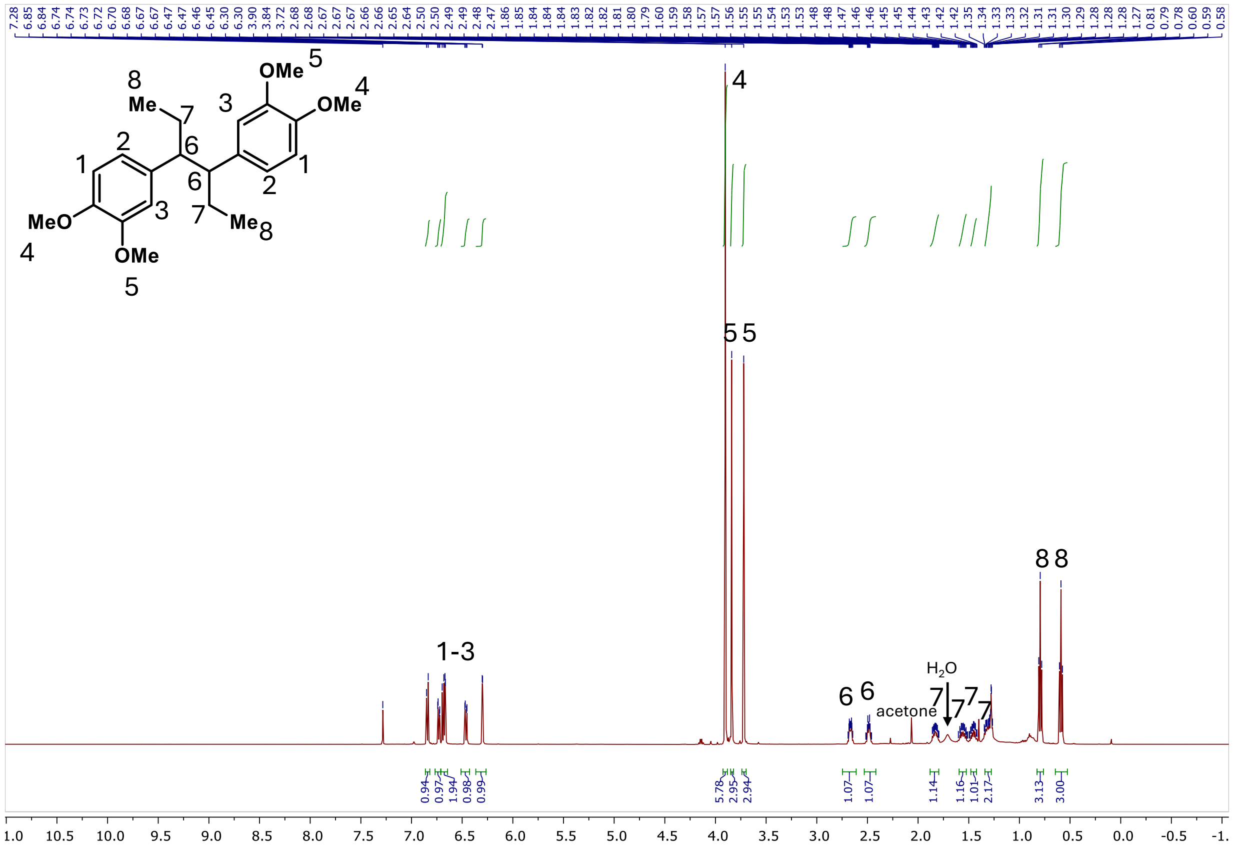

^{13}C NMR (151 MHz, $CDCl_3$) of compound **7a**

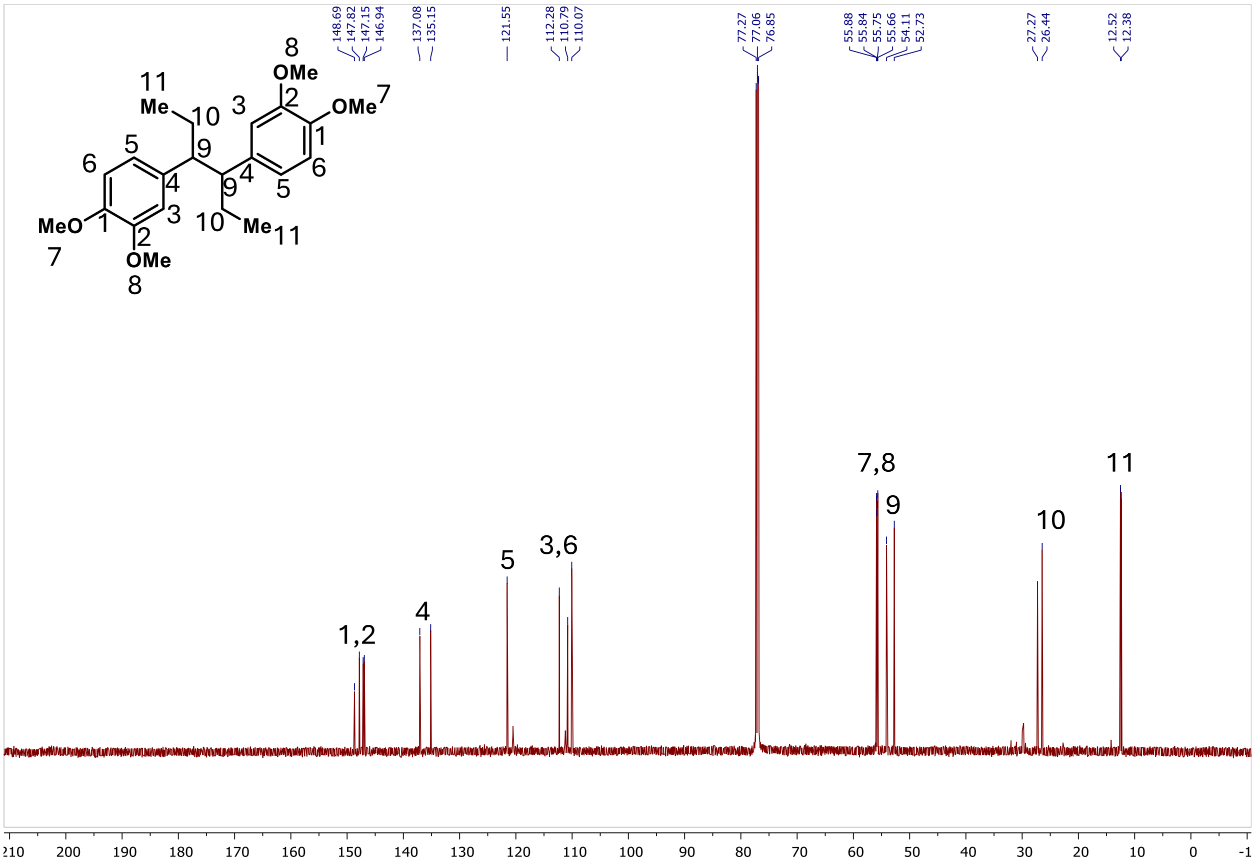

HRMS (ESI-TOF) of compound **7a**

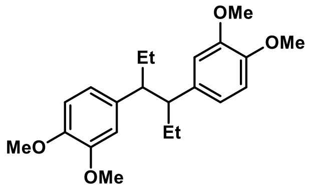

Chemical Formula: $C_{22}H_{30}O_4$
Molecular Weight: 358.4780

15-Oct-2025 12:12:30 BD-SI_dimethoxy1 Bushmin Dmitrii

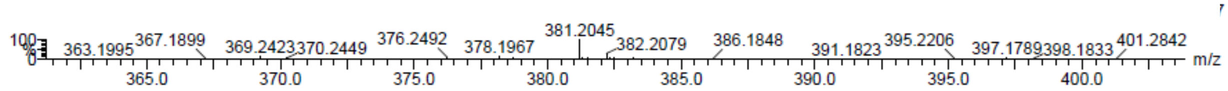

Minimum:				-1.5				
Maximum:		5.0	5.0	50.0				

Mass	Calc. Mass	mDa	PPM	DBE	i-FIT	Norm	Conf(%)	Formula
381.2045	381.2042	0.3	0.8	7.5	726.7	n/a	n/a	C22 H30 O4 Na

^{1}H NMR (300 MHz, $CDCl_3$) of compound **7b**

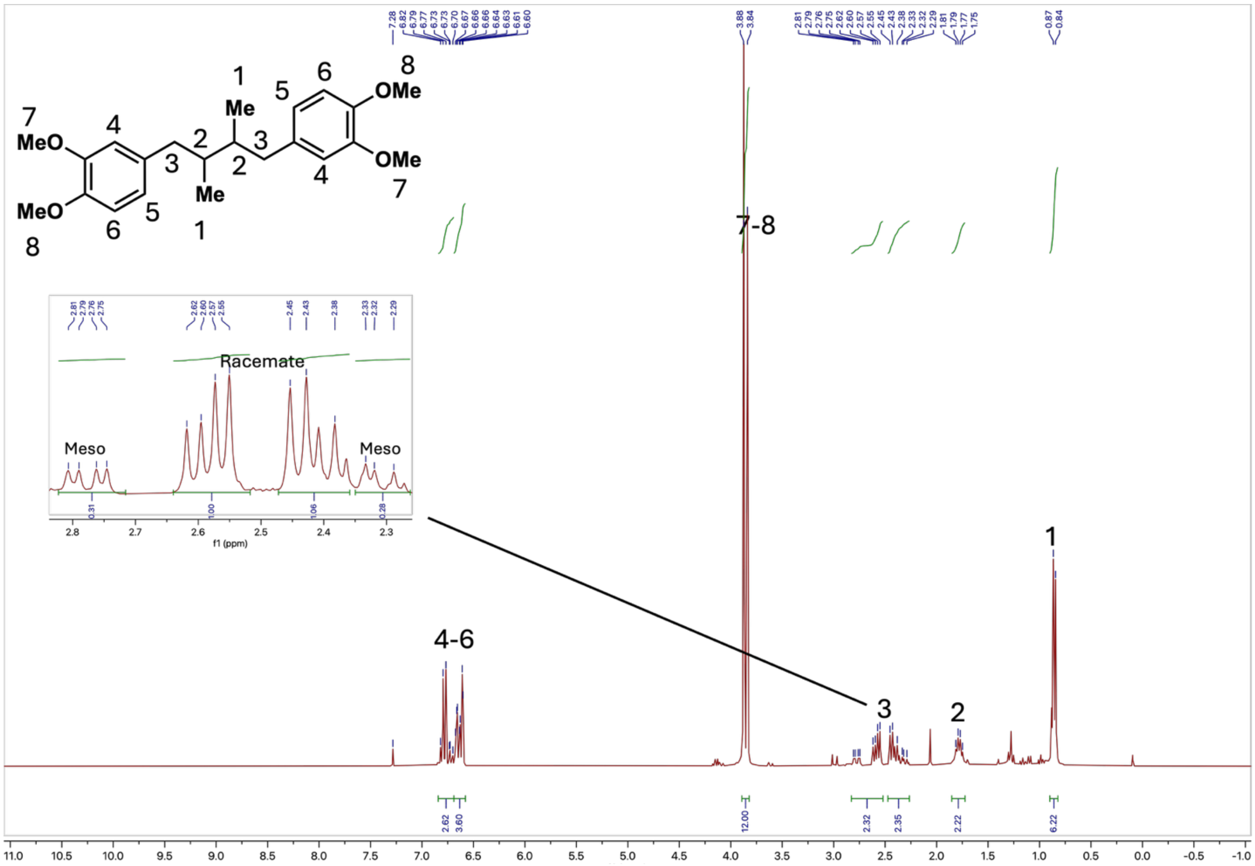

^{13}C NMR (75 MHz, $CDCl_3$) of compound **7b**

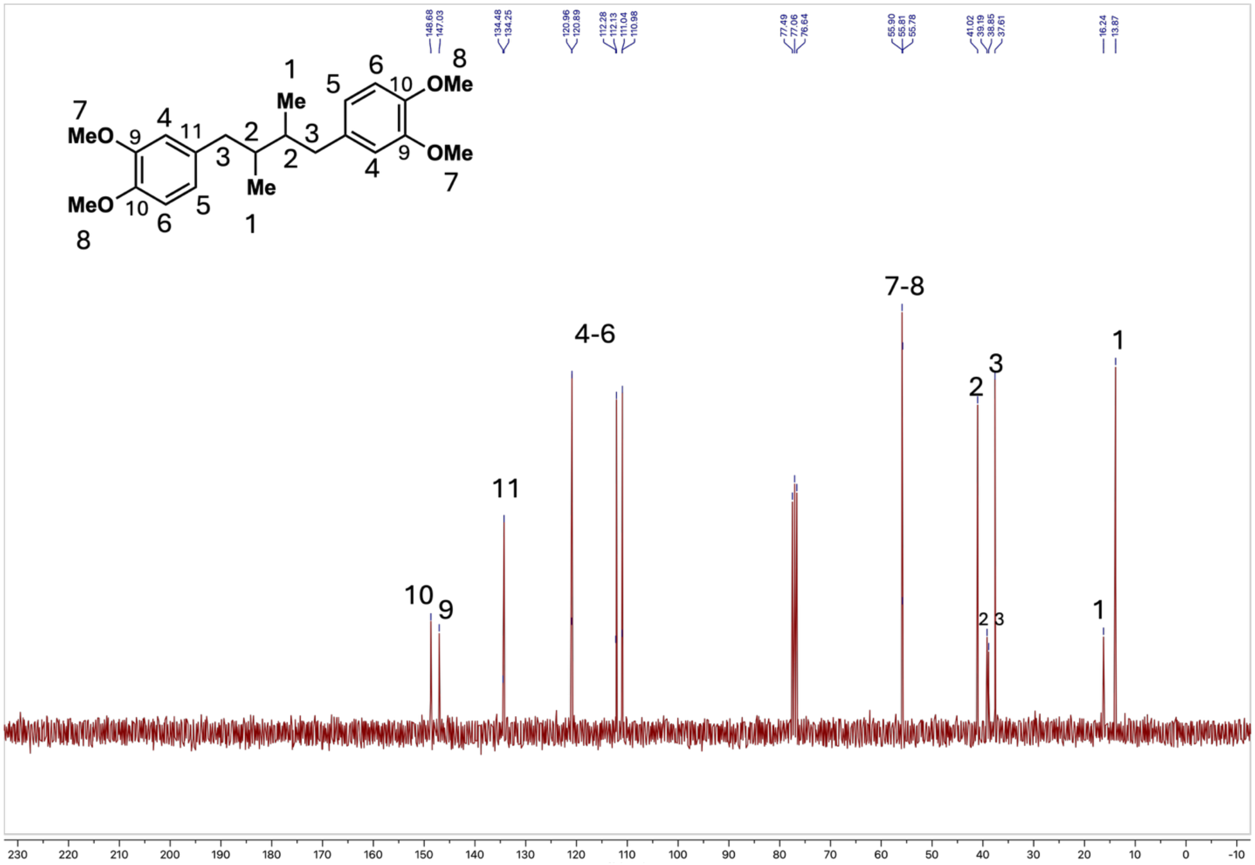

HRMS (ESI-TOF) of compound **7b**

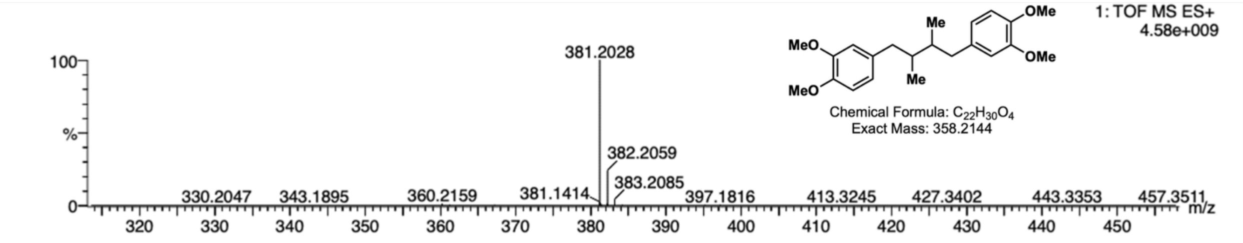

Minimum:				-1.5				
Maximum:		5.0	50.0	50.0				
Mass	Calc. Mass	mDa	PPM	DBE	i-FIT	Norm	Conf(%)	Formula
381.2028	381.2042	-1.4	-3.7	7.5	1704.4	n/a	n/a	C22 H30 O4 Na

LC/MS comparison Linear vs Branch

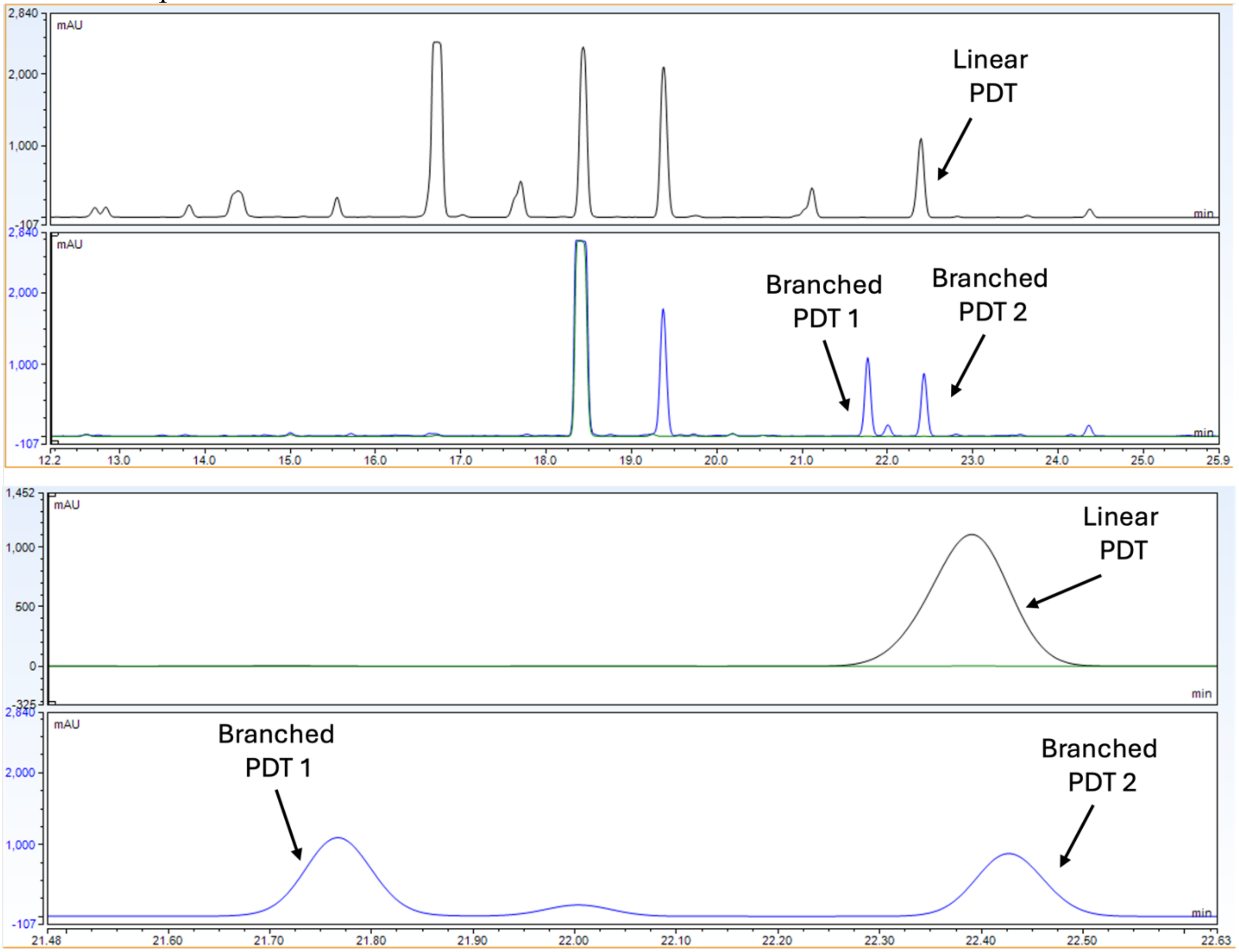

^{1}H NMR (300 MHz, $CDCl_3$) of compound **8a**

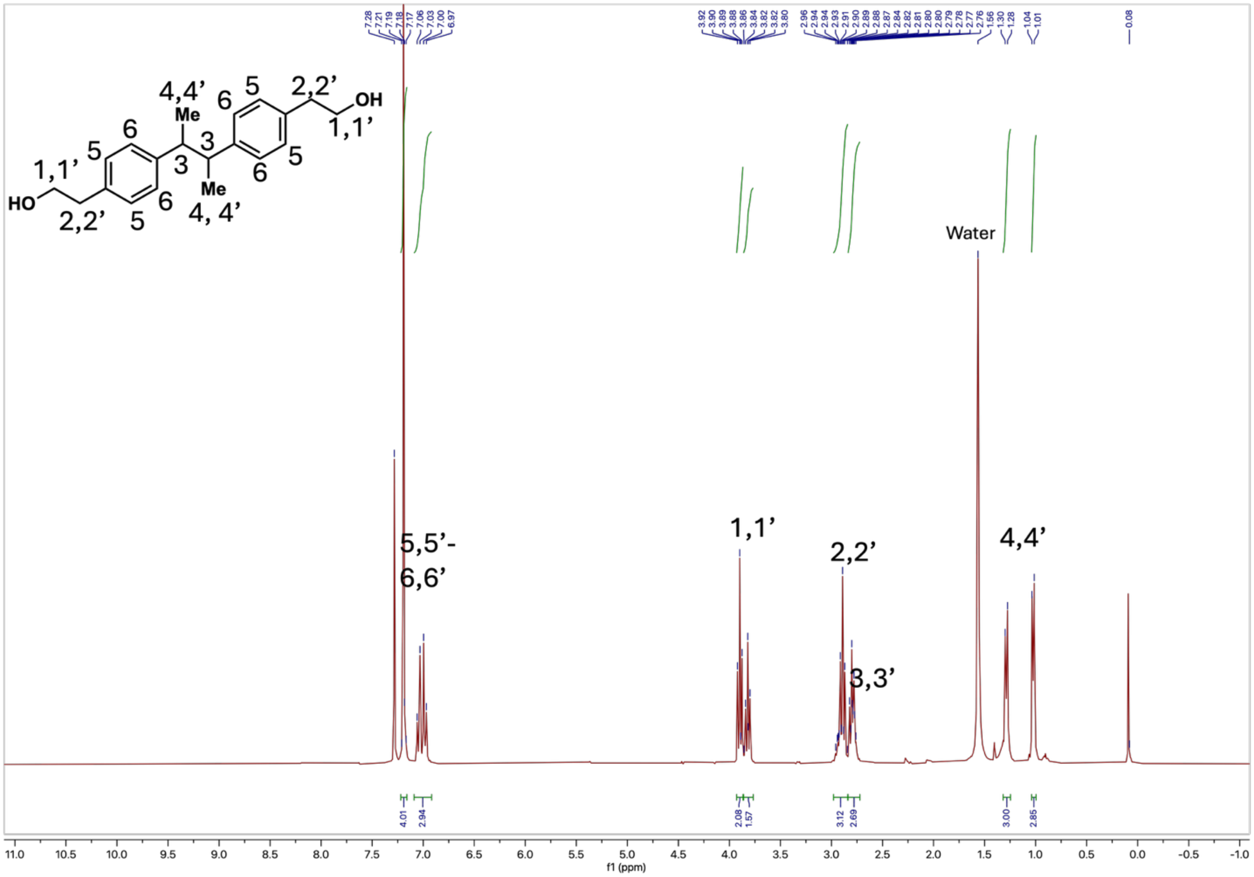

^{13}C NMR (151 MHz, $CDCl_3$) of compound **8a**

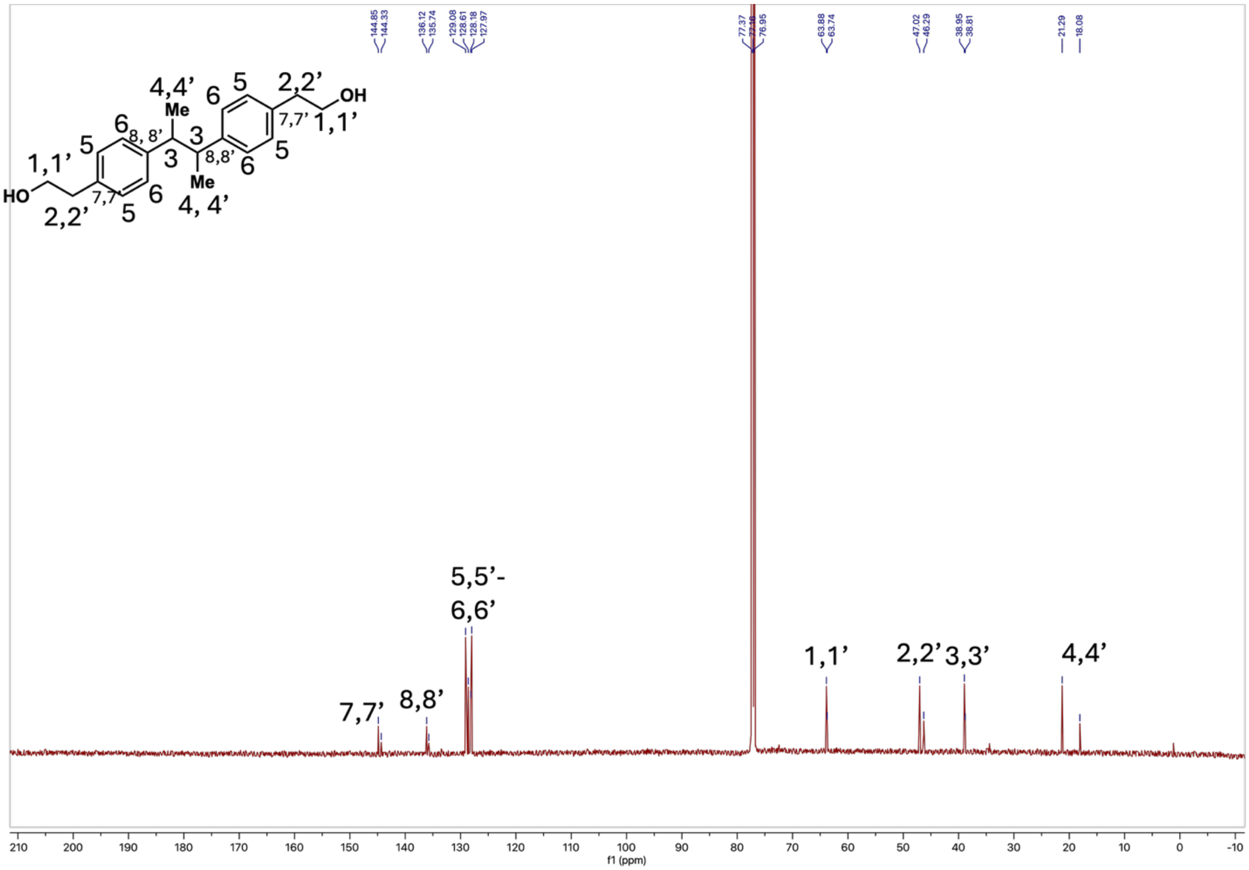

HRMS (ESI-TOF) of compound **8a**

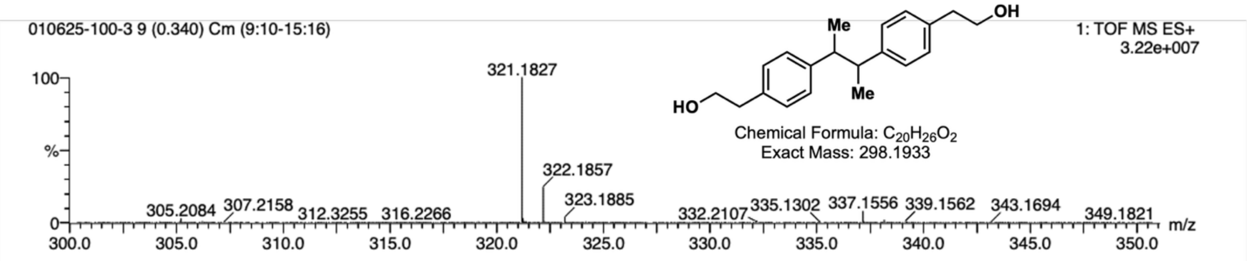

Minimum:				-1.5				
Maximum:		5.0	50.0	50.0				
Mass	Calc. Mass	mDa	PPM	DBE	i-FIT	Norm	Conf(%)	Formula
321.1827	321.1830	-0.3	-0.9	7.5	975.3	n/a	n/a	C20 H26 O2 Na

^{1}**H NMR** (800 MHz, $CDCl_3$) of compound **8b**

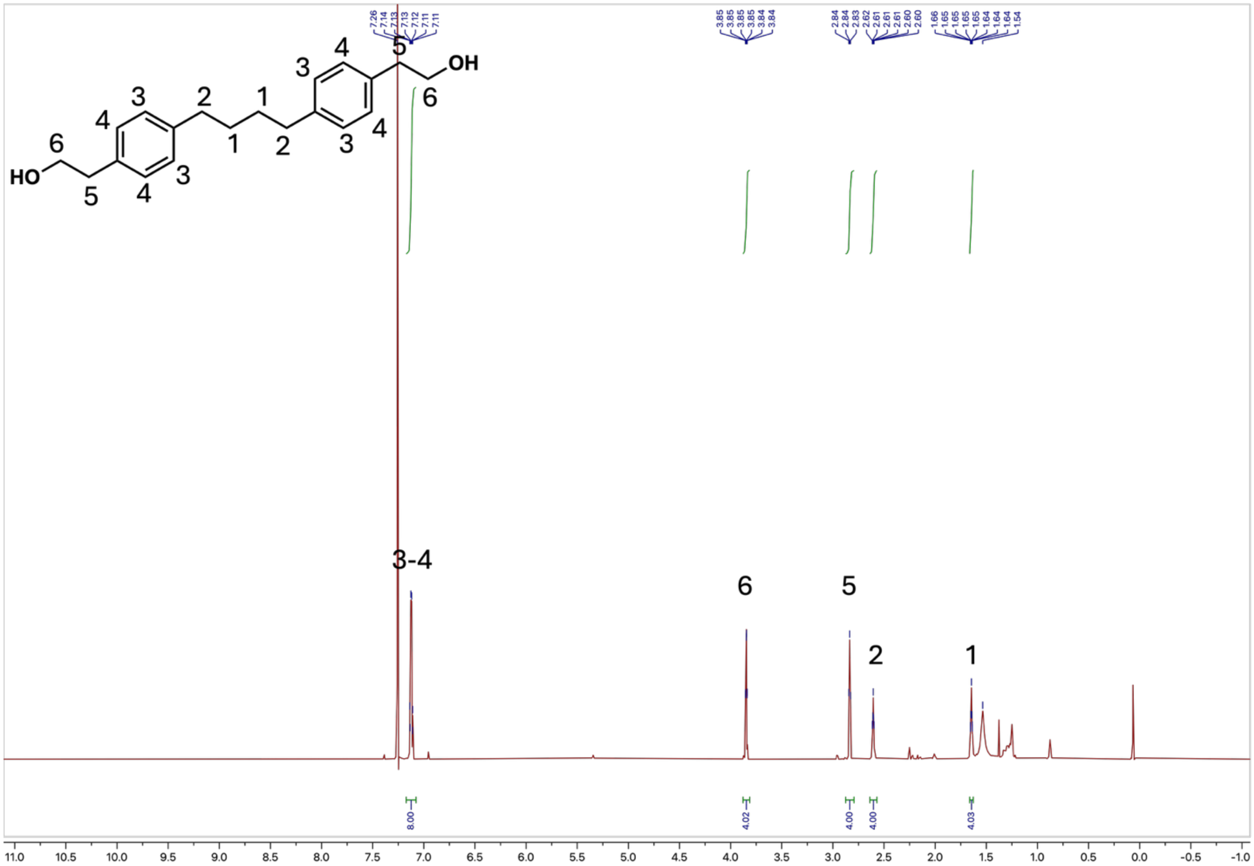

^{13}**C NMR** (201 MHz, $CDCl_3$) of compound **8b**

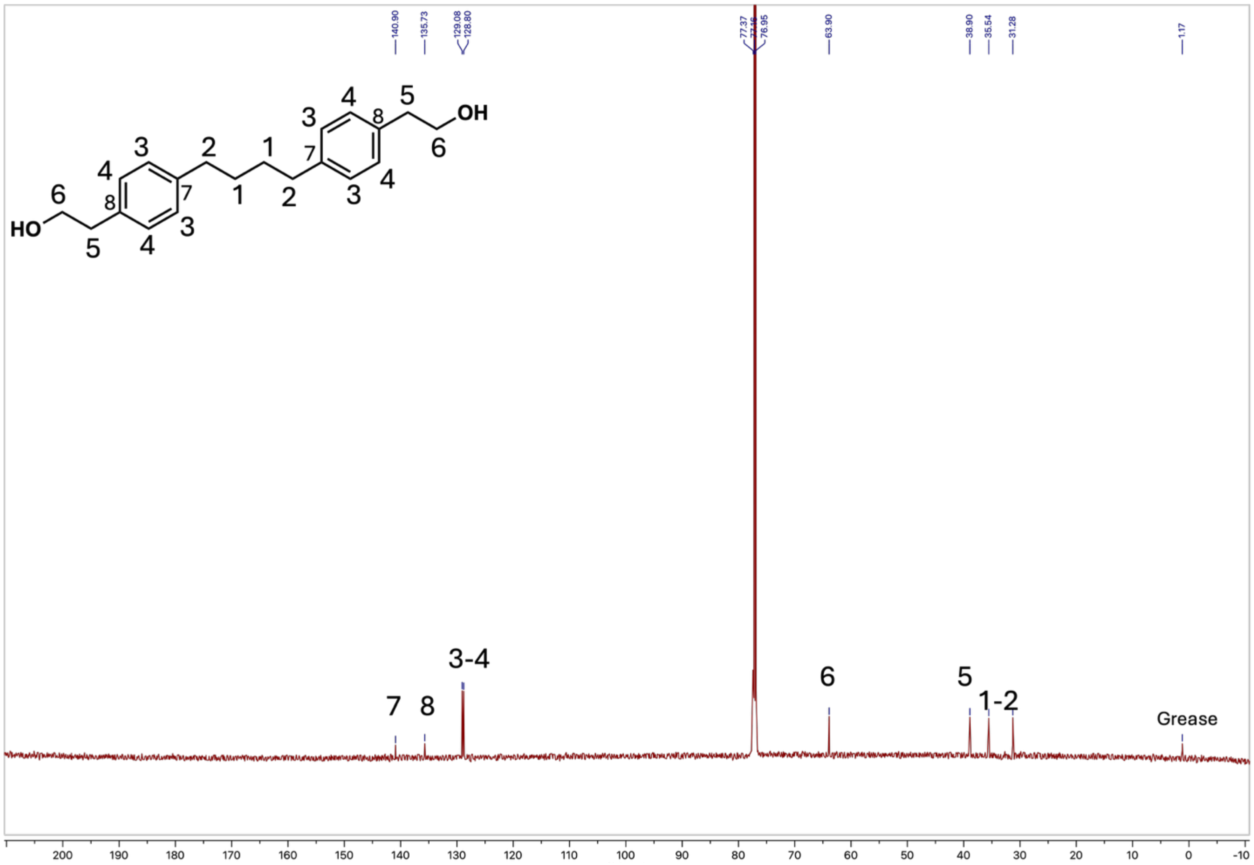

HRMS (ESI-TOF) of compound **8b**

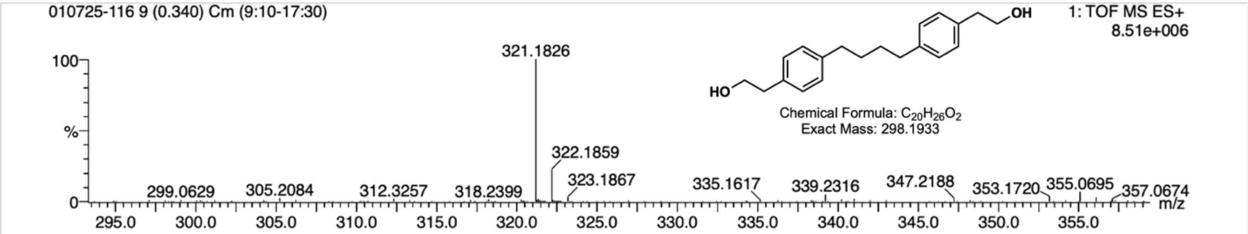

Minimum:				-1.5				
Maximum:		5.0	5.0	50.0				
Mass	Calc. Mass	mDa	PPM	DBE	i-FIT	Norm	Conf(%)	Formula
321.1826	321.1830	-0.4	-1.2	7.5	445.6	n/a	n/a	C20 H26 O2 Na

^{1}H NMR comparison linear vs branch

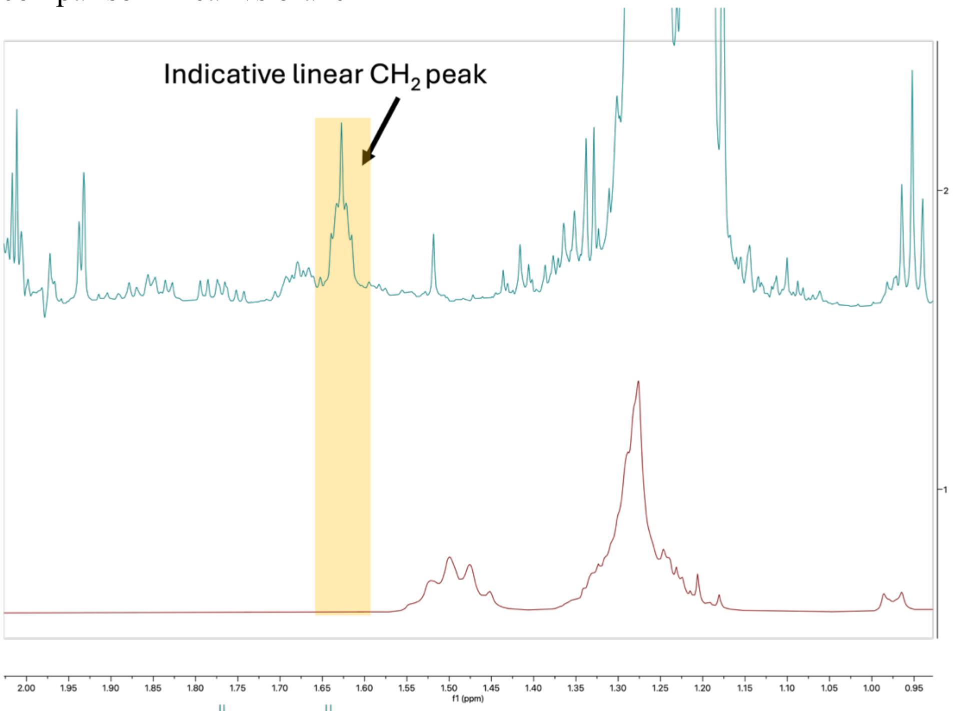

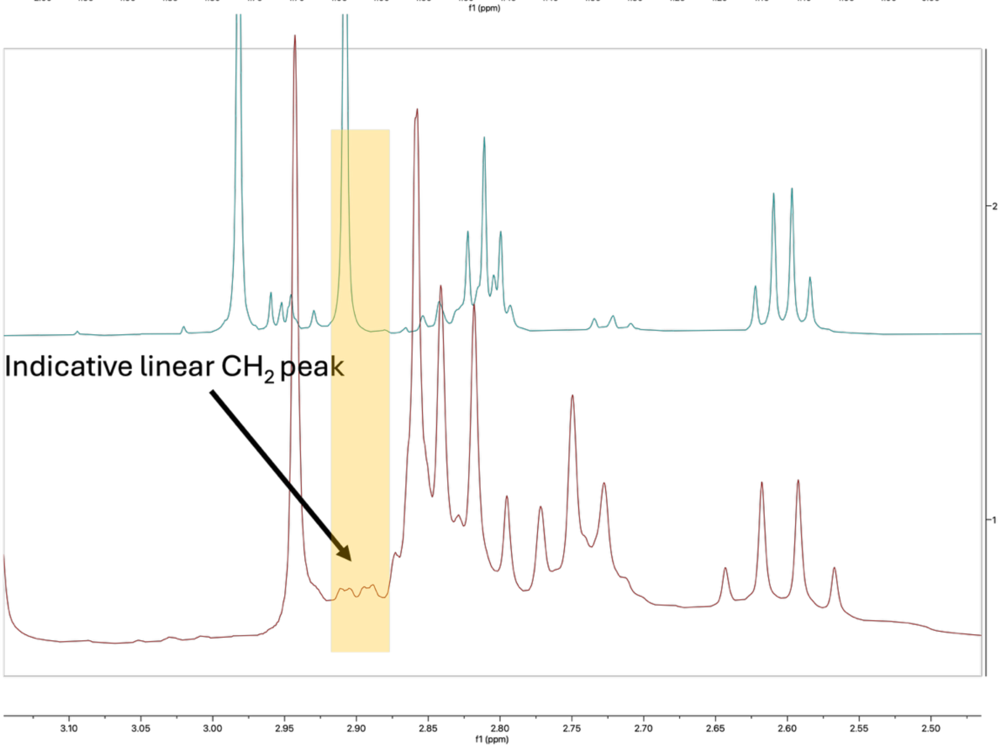

^{1}H NMR (800 MHz, $CDCl_3$) of compound **9a**

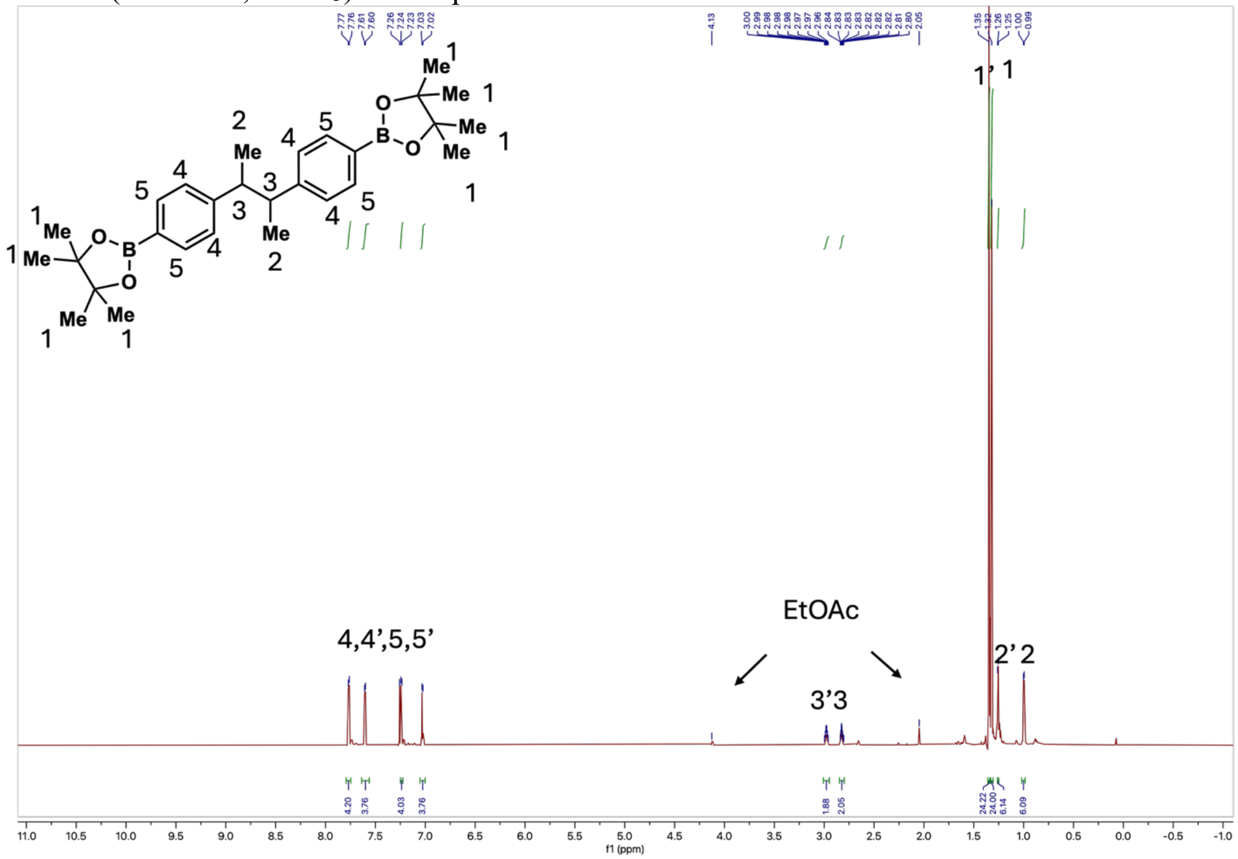

^{13}C NMR (151 MHz, CDCl$_3$) of compound **9a**

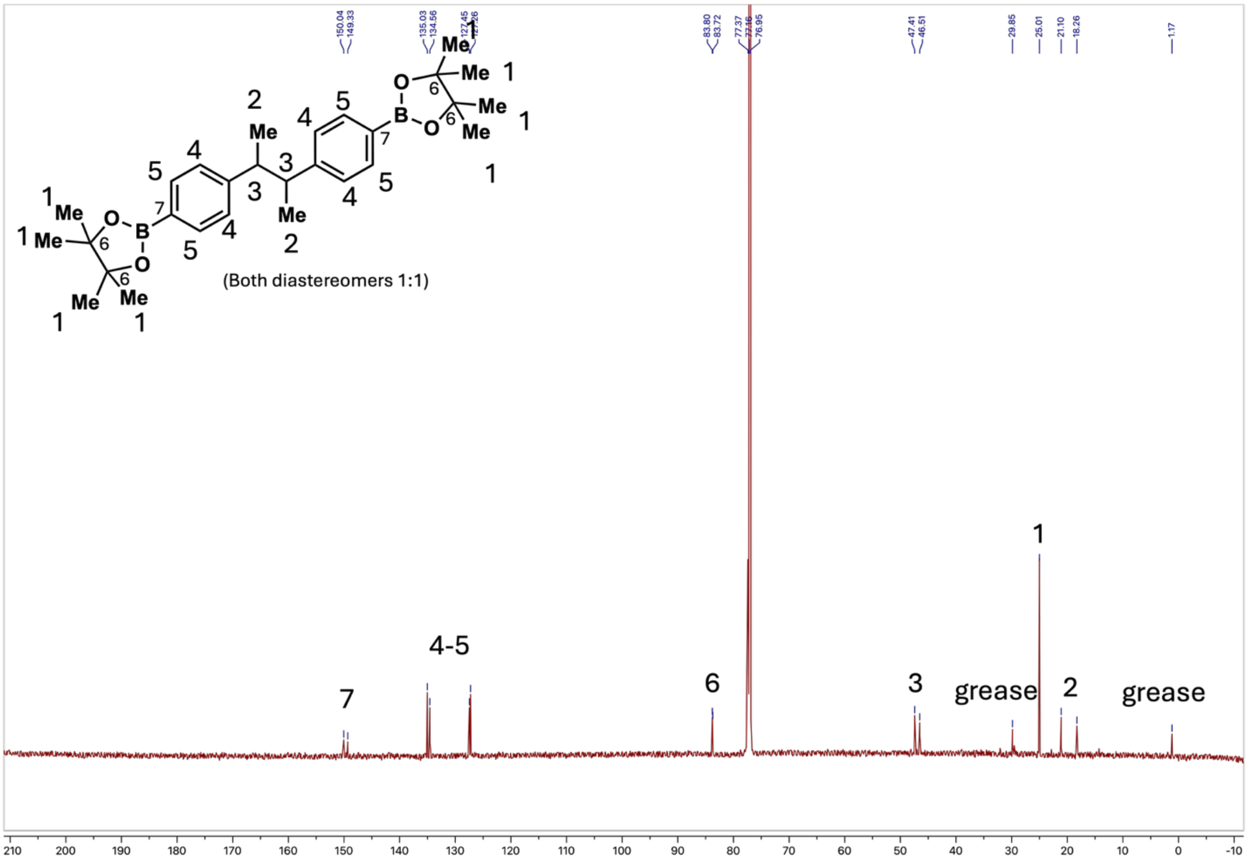

^{11}B NMR (96 MHz, $CDCl_3$) of compound **9a**

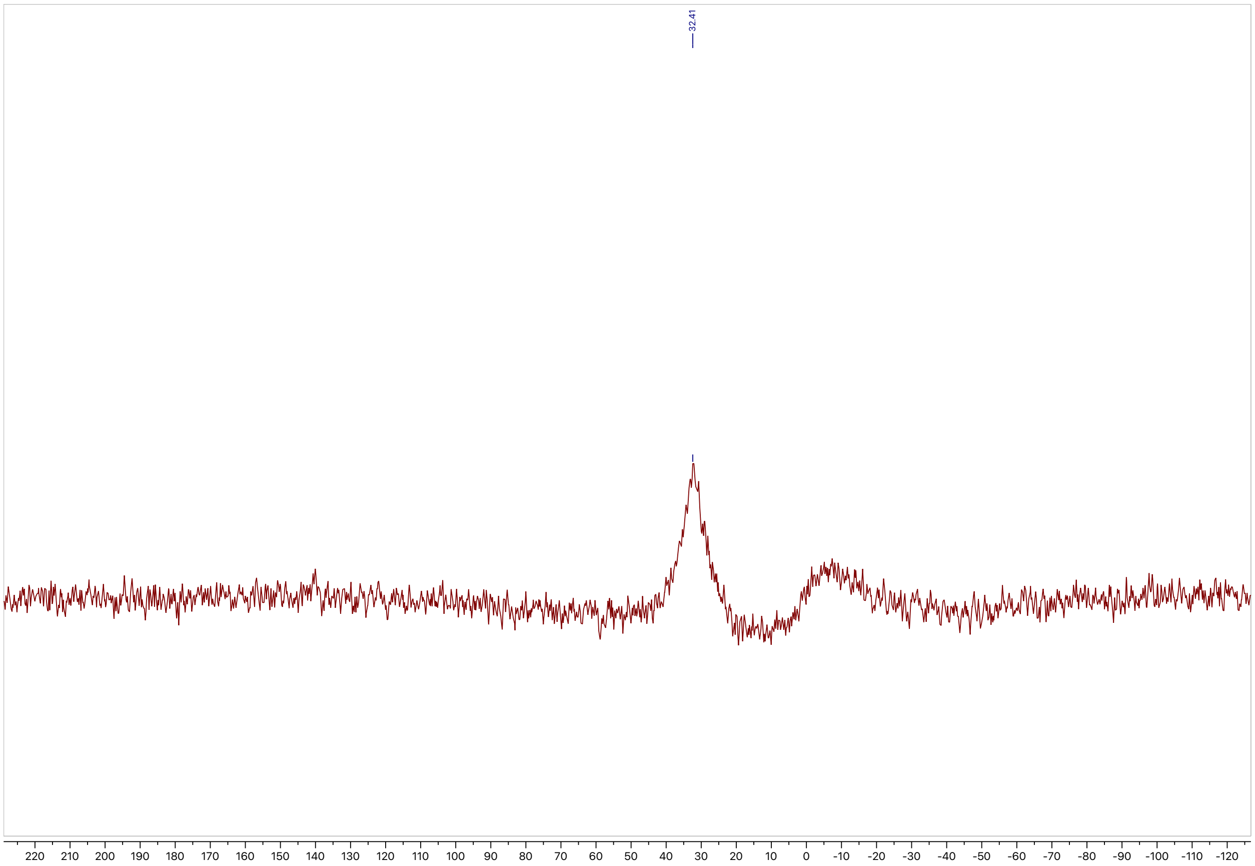

HRMS (ESI-TOF) of compound **9a**

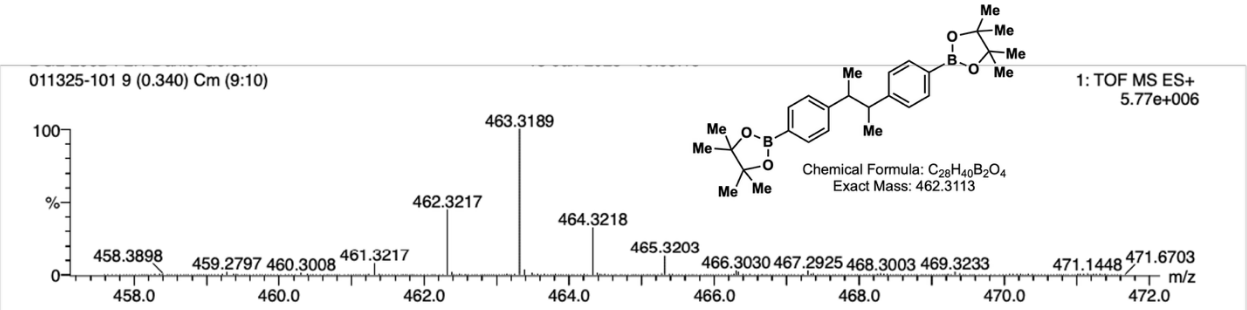

Minimum:				-1.5				
Maximum:		5.0	5.0	50.0				
Mass	Calc. Mass	mDa	PPM	DBE	i-FIT	Norm	Conf(%)	Formula
463.3189	463.3191	-0.2	-0.4	9.5	1037.3	n/a	n/a	C28 H41 O4 B2

^{1}H NMR (800 MHz, $CDCl_3$) of compound **9b**

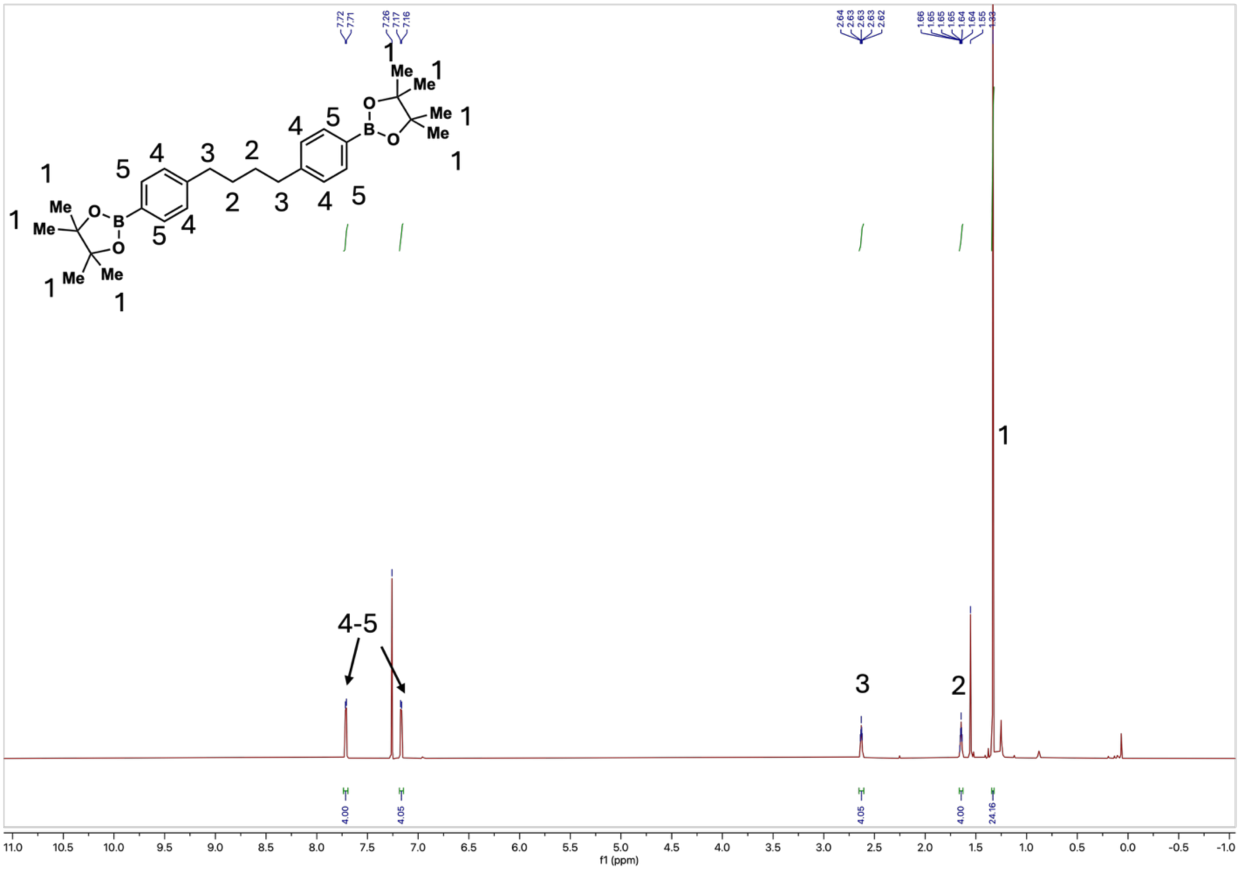

^{13}C NMR (151 MHz, CDCl$_3$) of compound **9b**

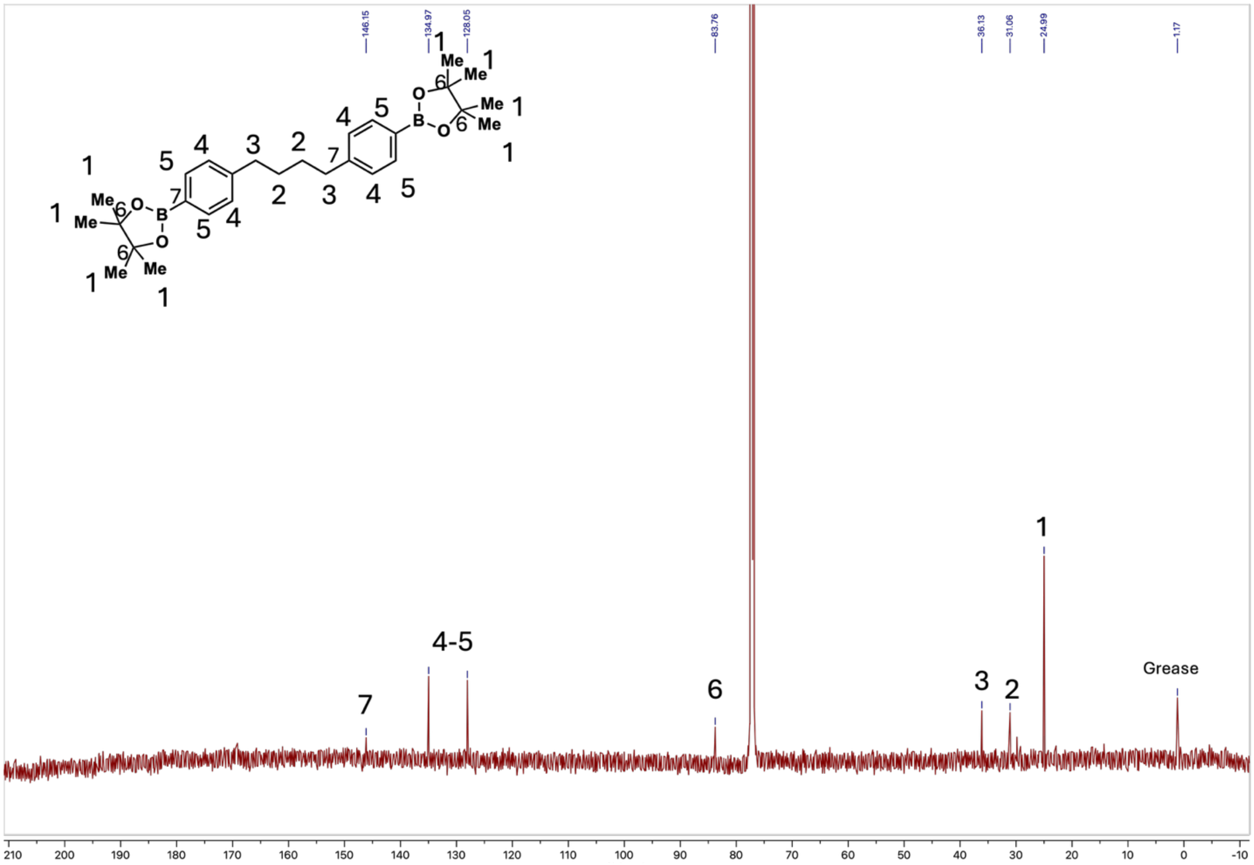

^{11}B NMR (96 MHz, $CDCl_3$) of compound **9b**

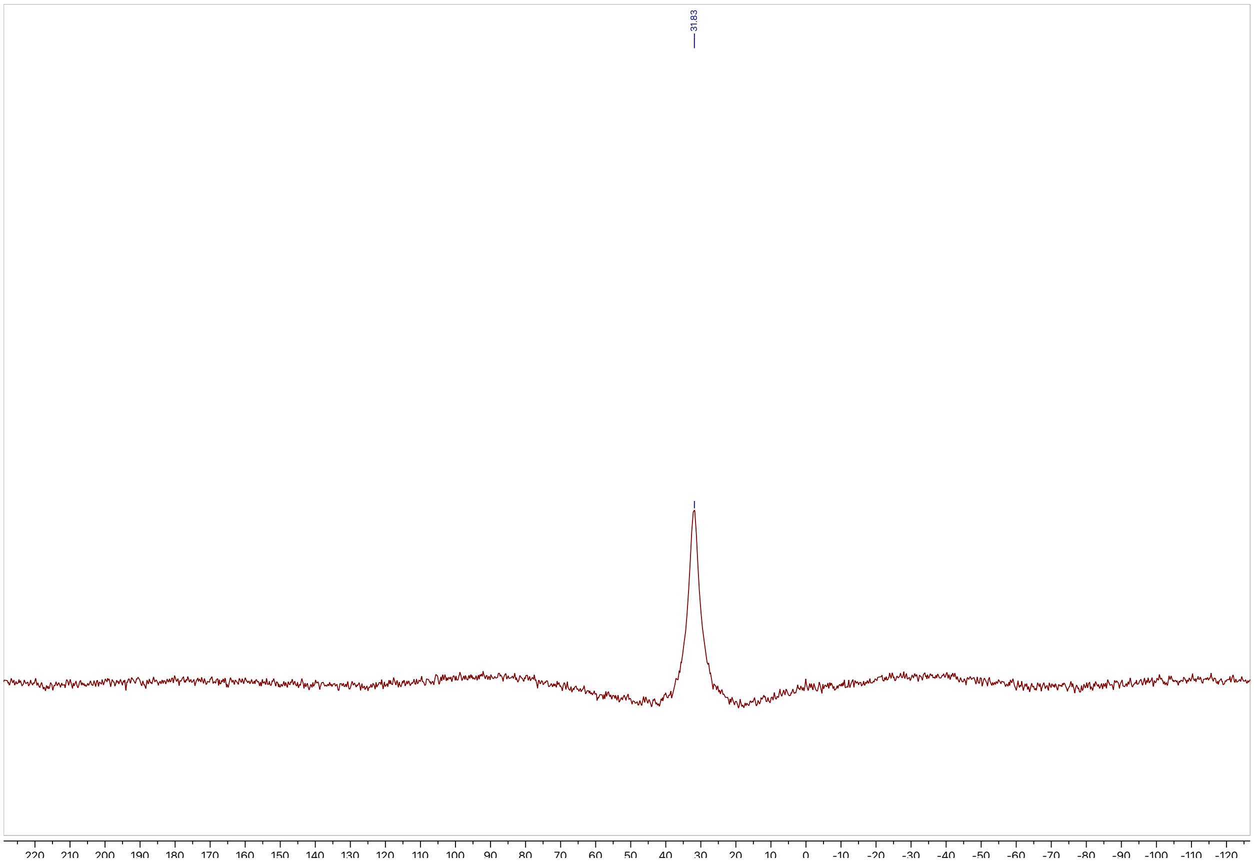

HRMS (ESI-TOF) of compound **9b**

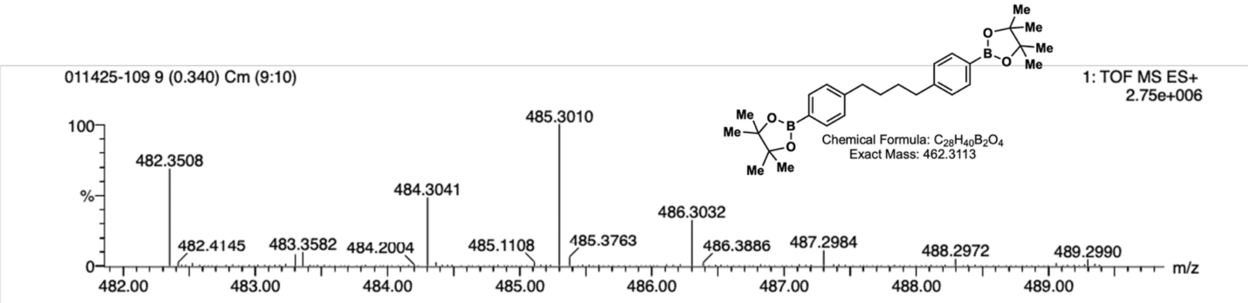

Minimum:				-1.5				
Maximum:		5.0	5.0	50.0				
Mass	Calc. Mass	mDa	PPM	DBE	i-FIT	Norm	Conf(%)	Formula
485.3010	485.3010	0.0	0.0	9.5	899.3	n/a	n/a	C28 H40 O4 Na B2

Crude ^{1}H NMR comparison linear (top) vs branched (bottom)

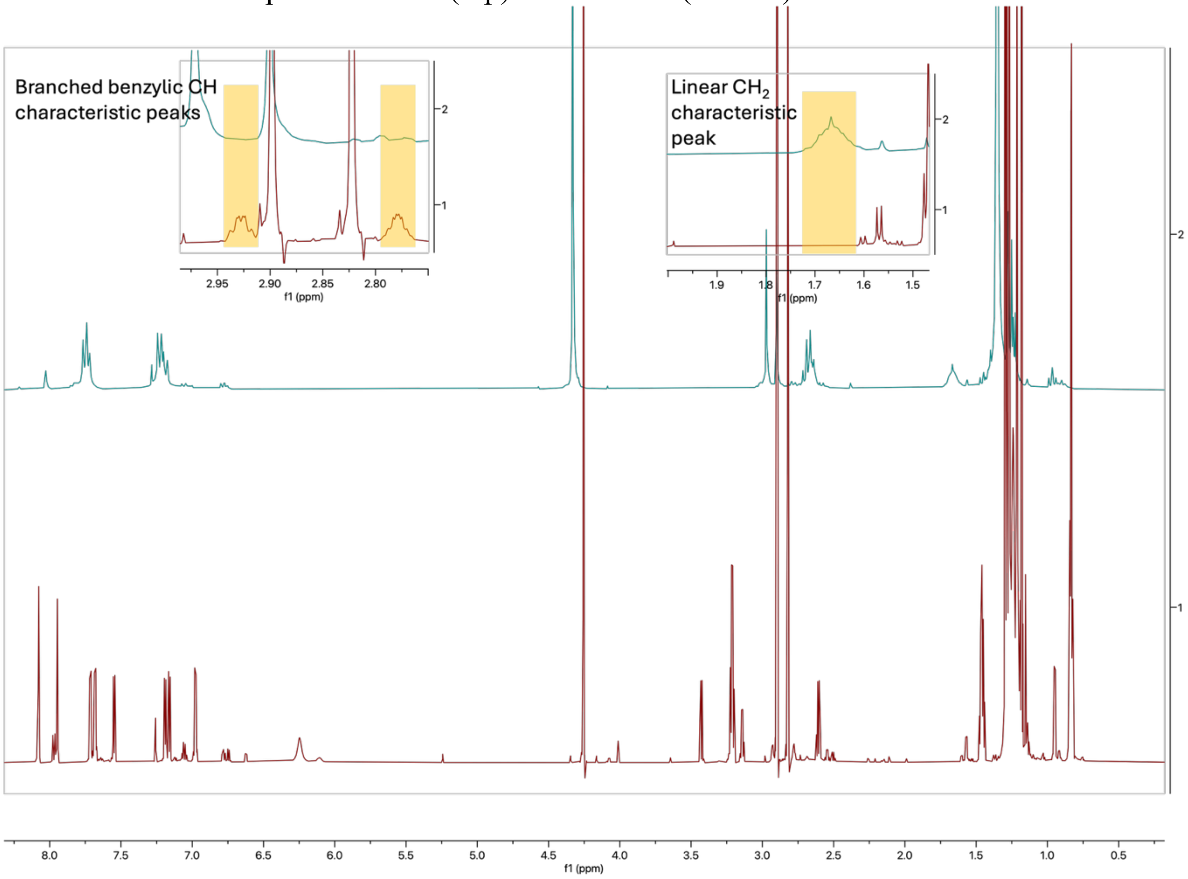

^{1}H NMR (400 MHz, $CDCl_3$) of compound **10a**

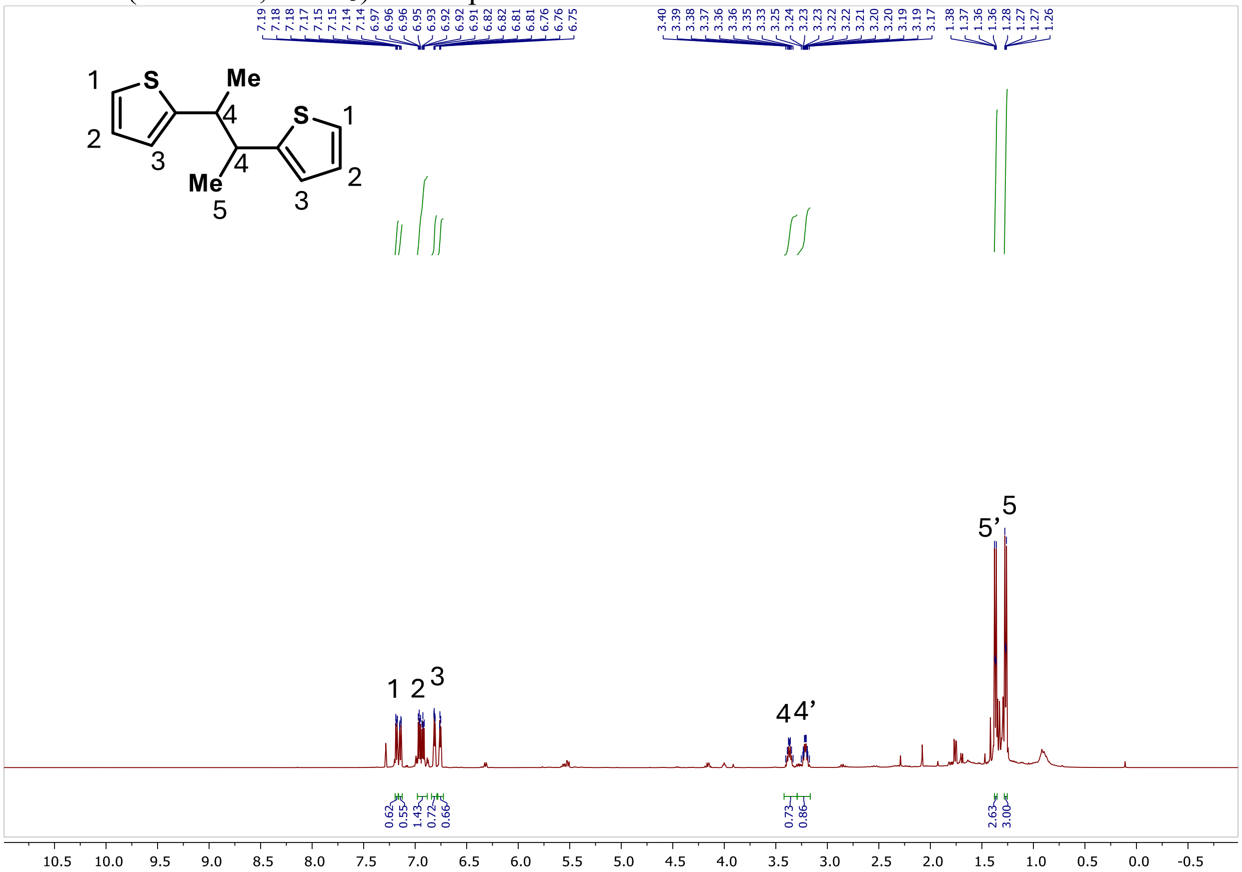

^{13}C NMR (151 MHz, $CDCl_3$) of compound **10a**

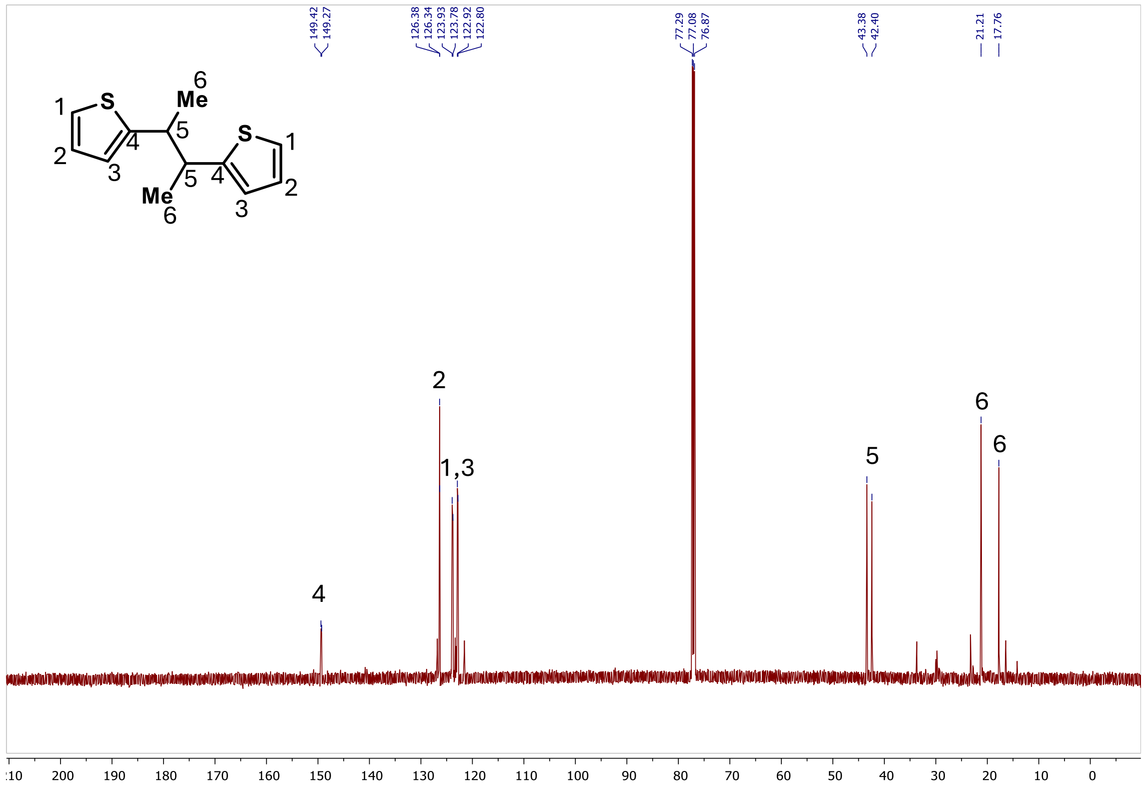

GC-MS (EI) of compound **10a**

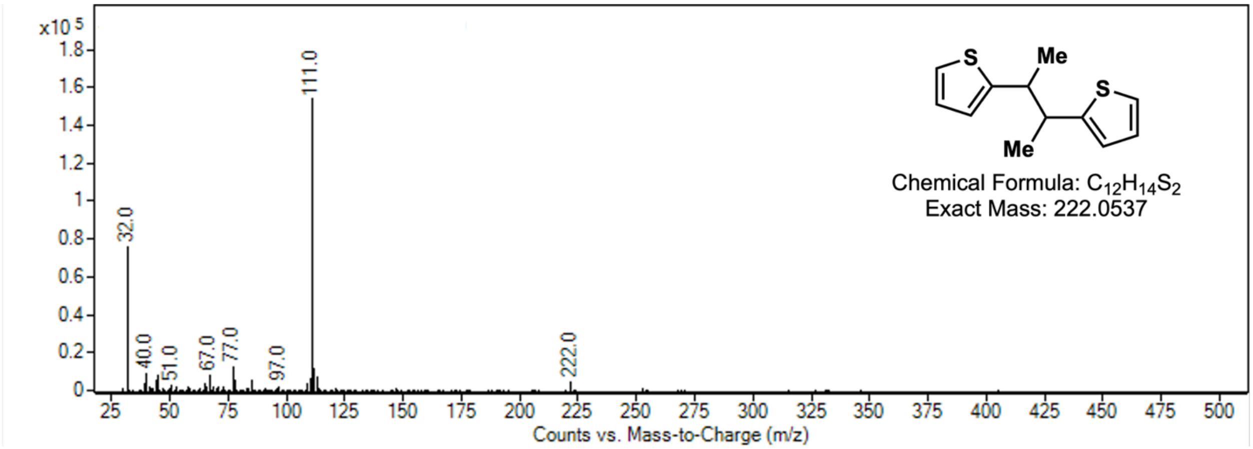

^{1}H NMR (500 MHz, $CDCl_3$) of compound **10b**

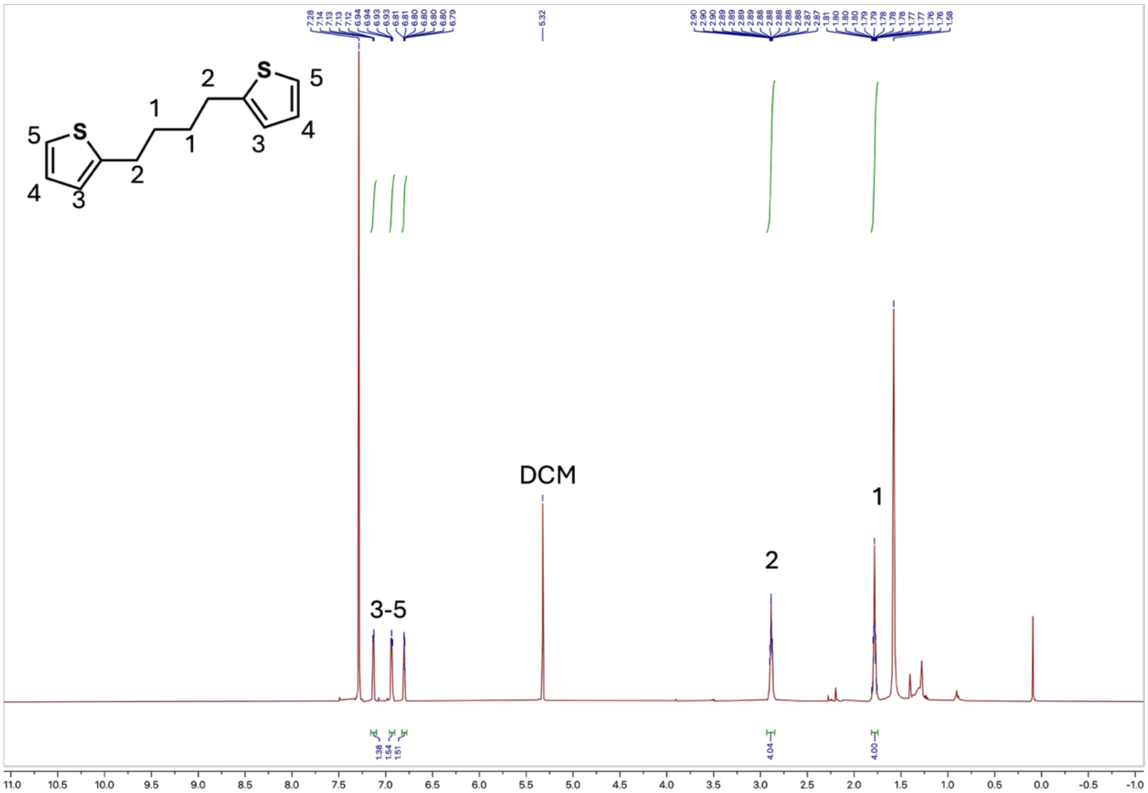

^{13}C NMR (151 MHz, $CDCl_3$) of compound **10b**

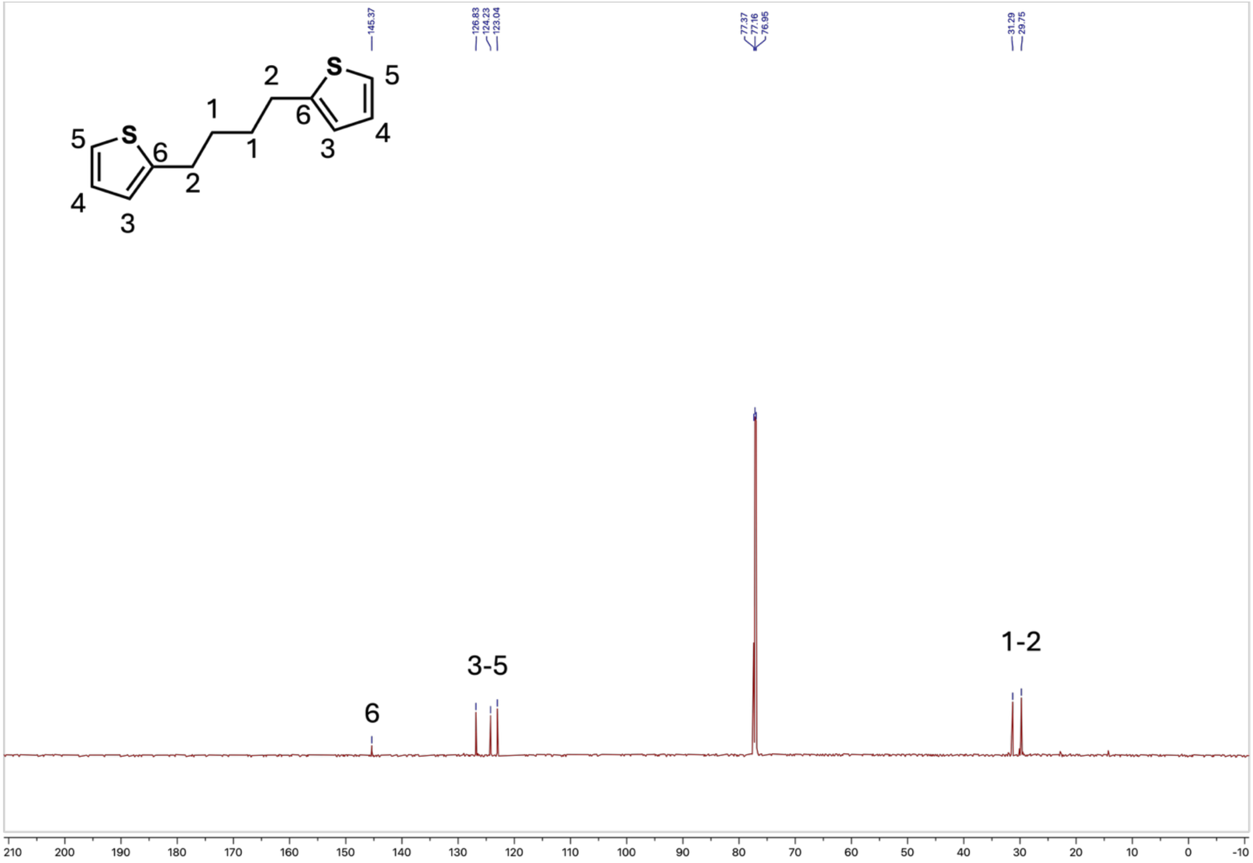

GC-MS (EI) of compound **10b**

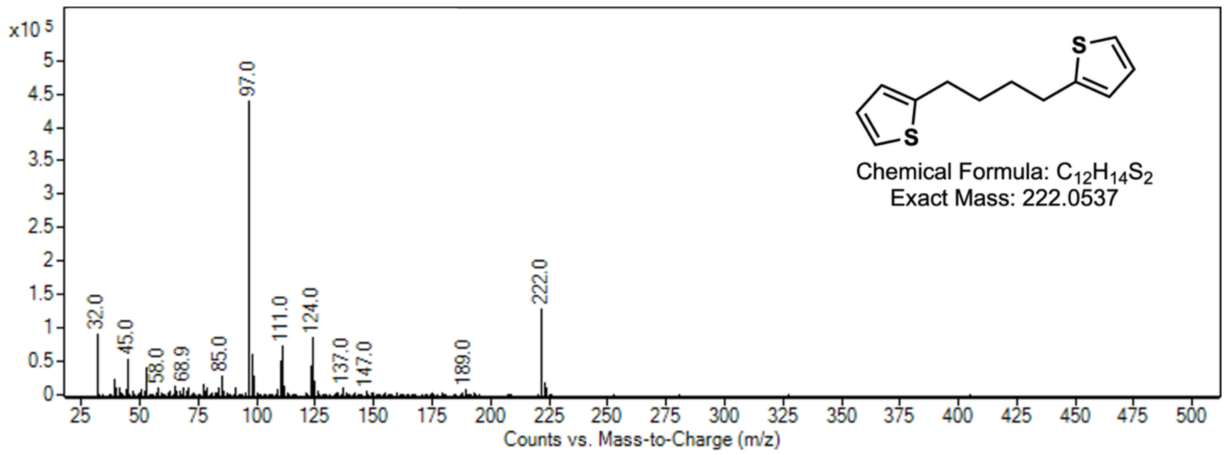

GC/MS Linear (top) vs Branched (bottom)

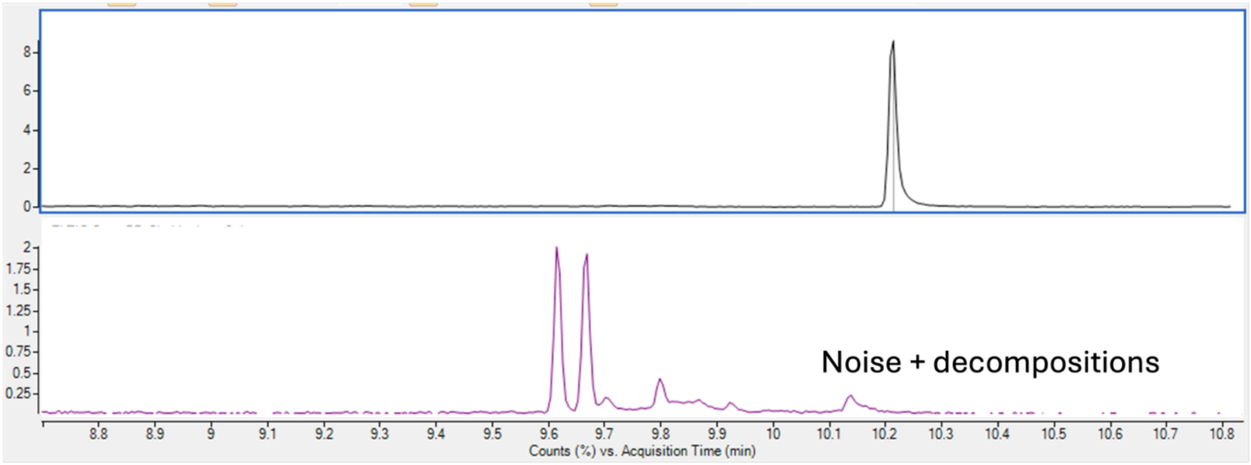

^{1}H NMR (600 MHz, $CDCl_3$) of compound **11a**

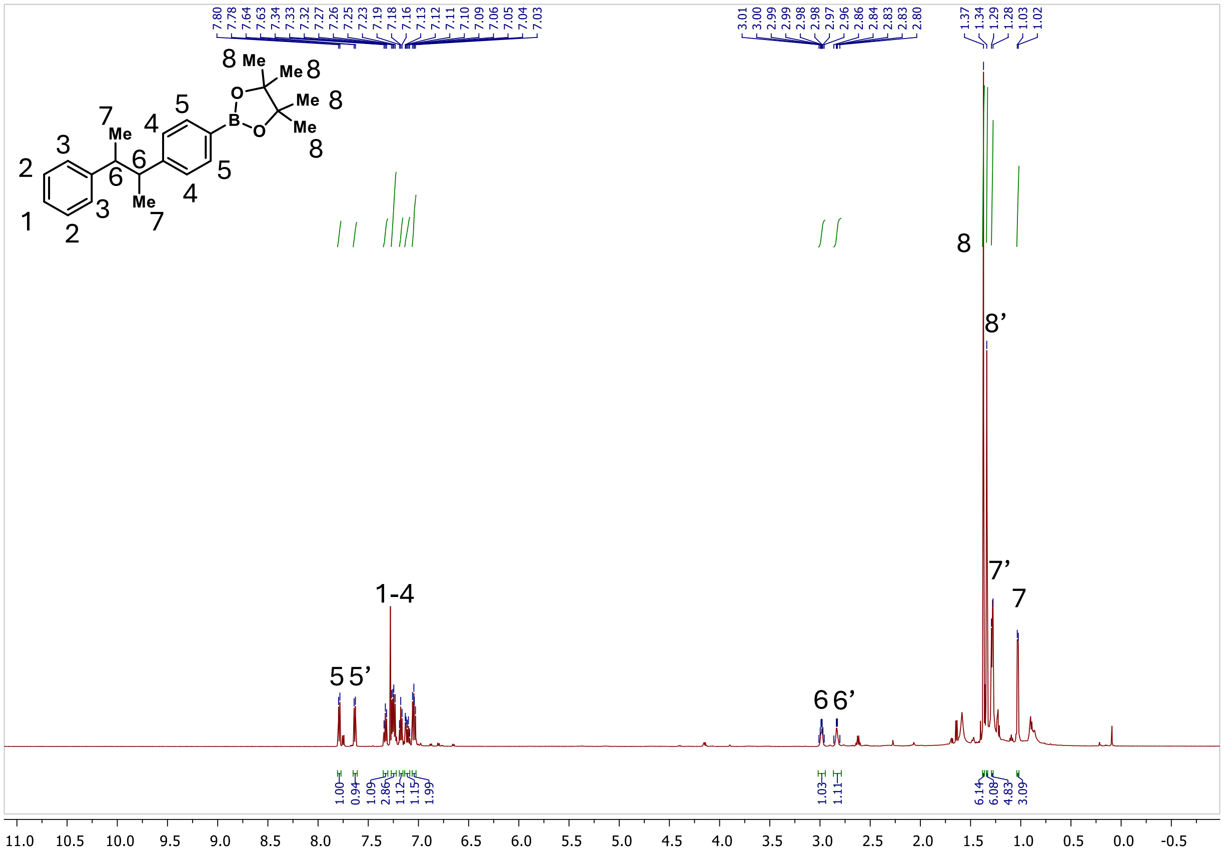

^{13}C NMR (151 MHz, $CDCl_3$) of compound **11a**

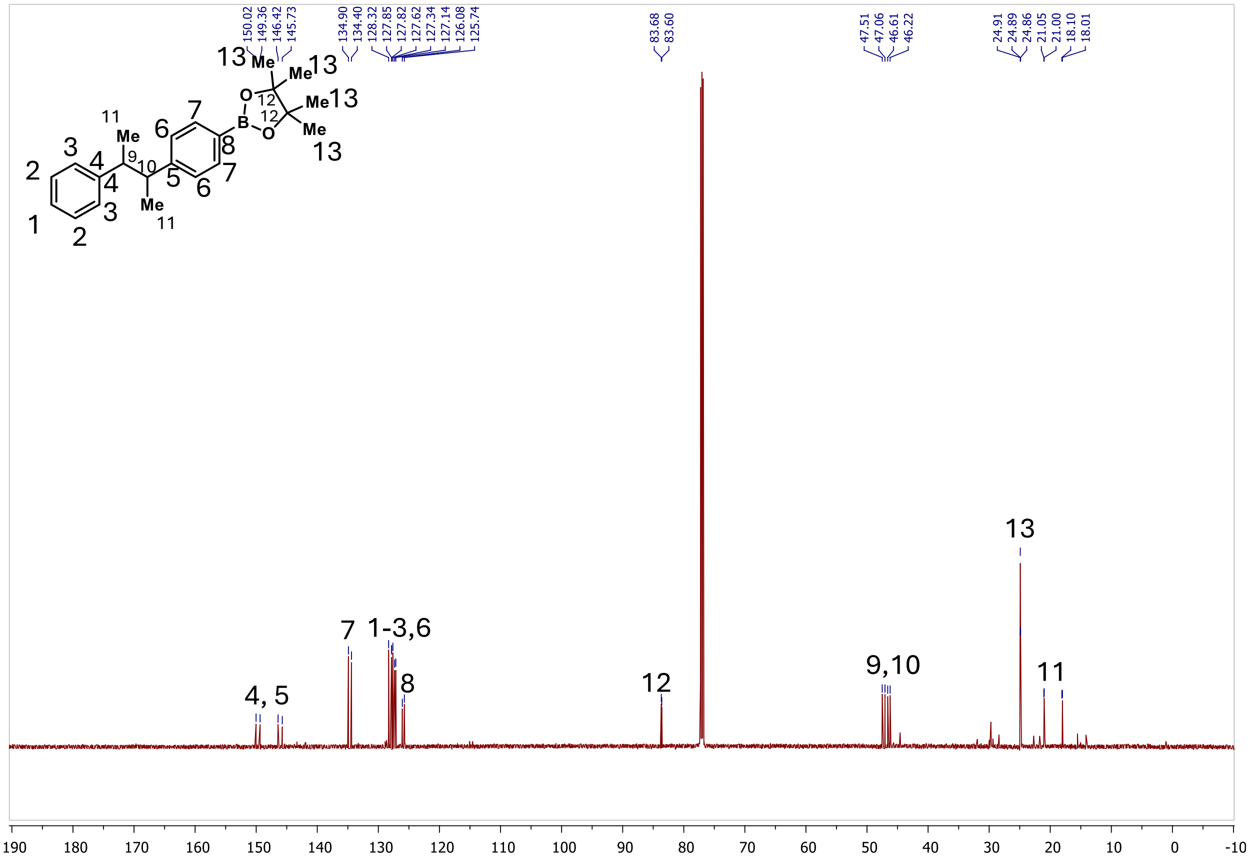

^{11}B NMR (96 MHz, $CDCl_3$) of compound **11a**

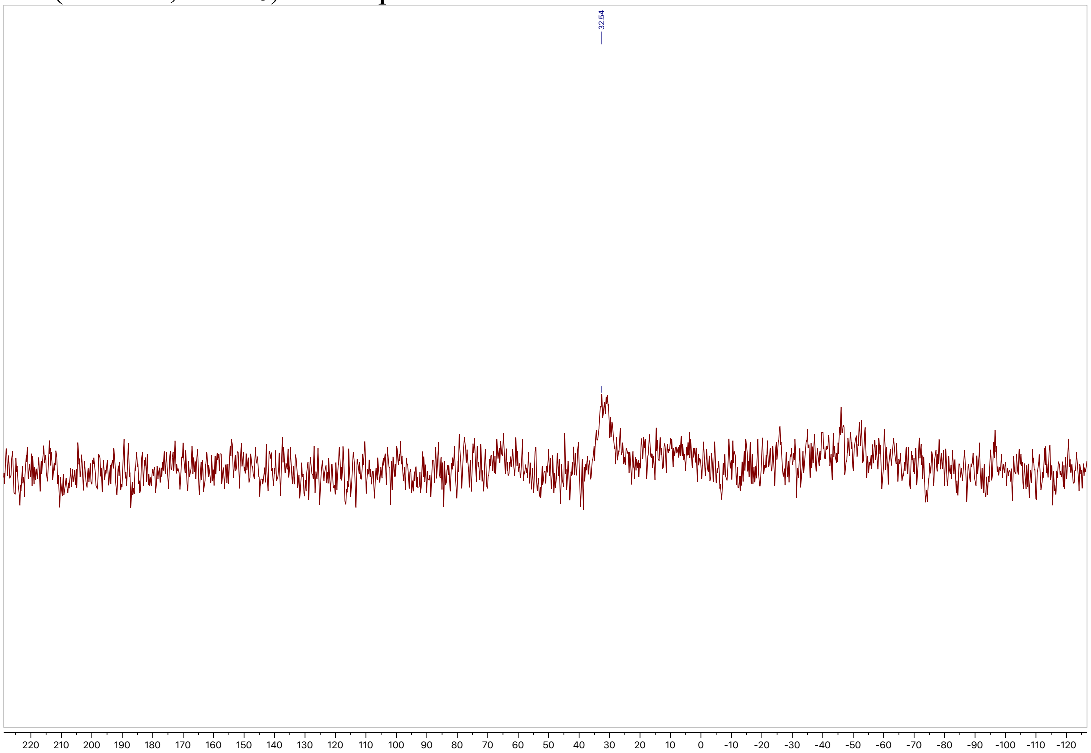

HRMS (ASAP-TOF) of compound **11a**

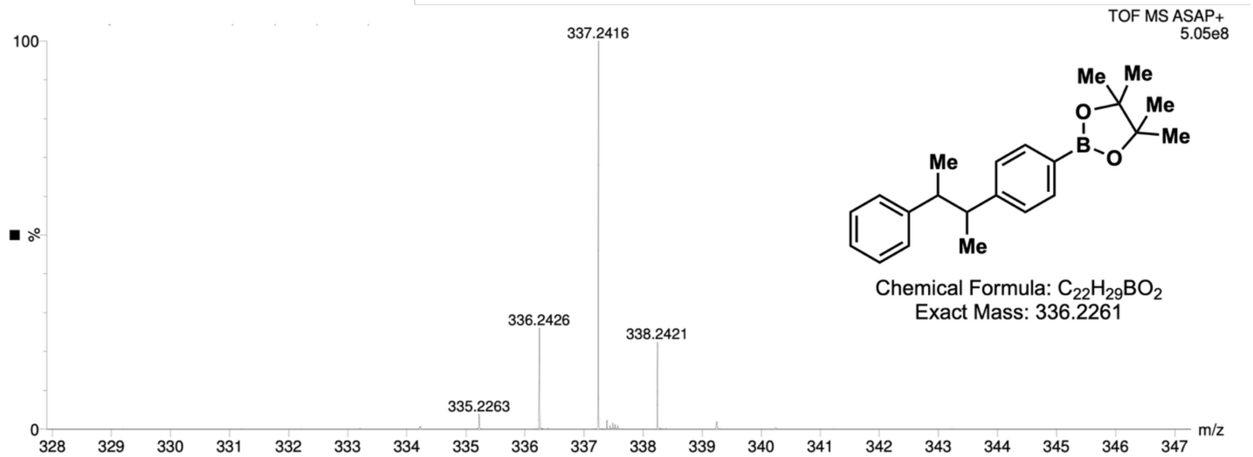

^{1}H NMR (600 MHz, $CDCl_3$) of compound **11b**

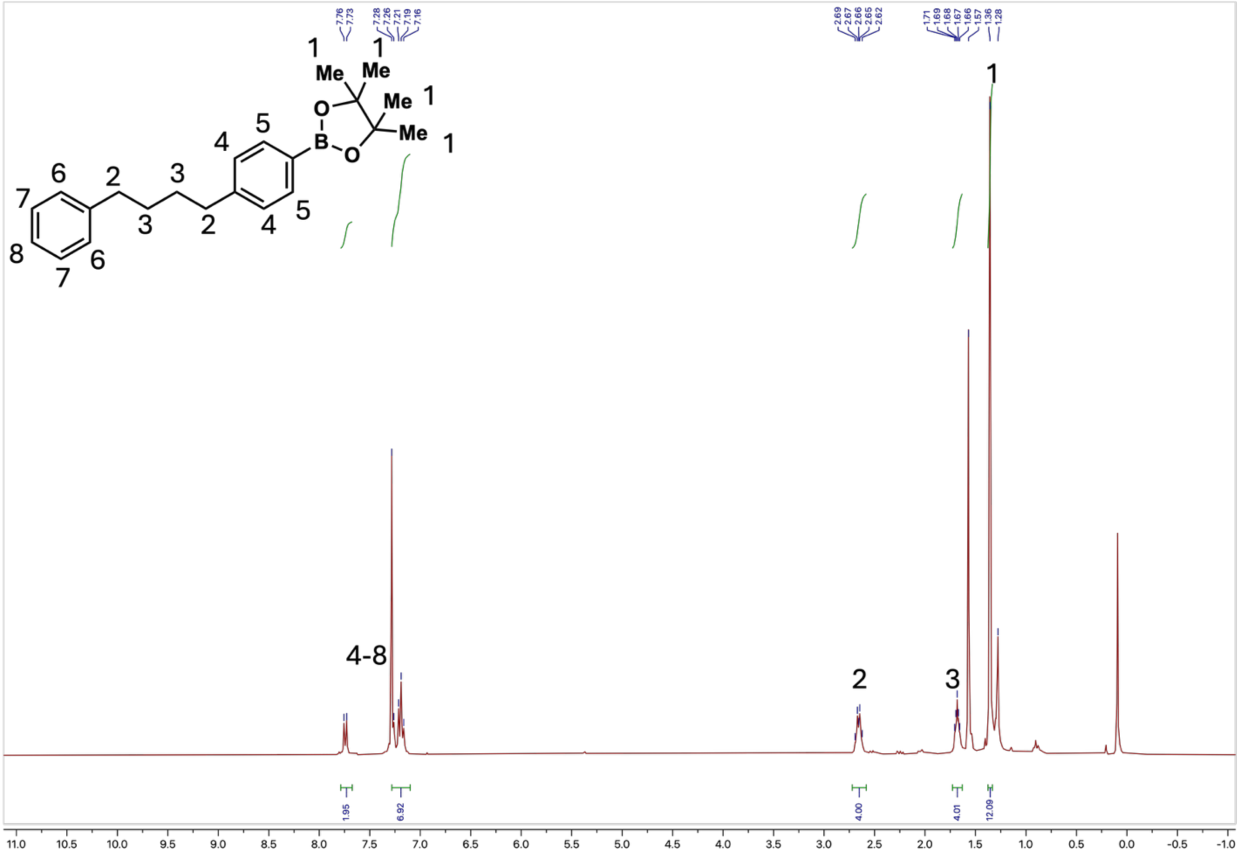

^{13}C NMR (151 MHz, $CDCl_3$) of compound **11b**

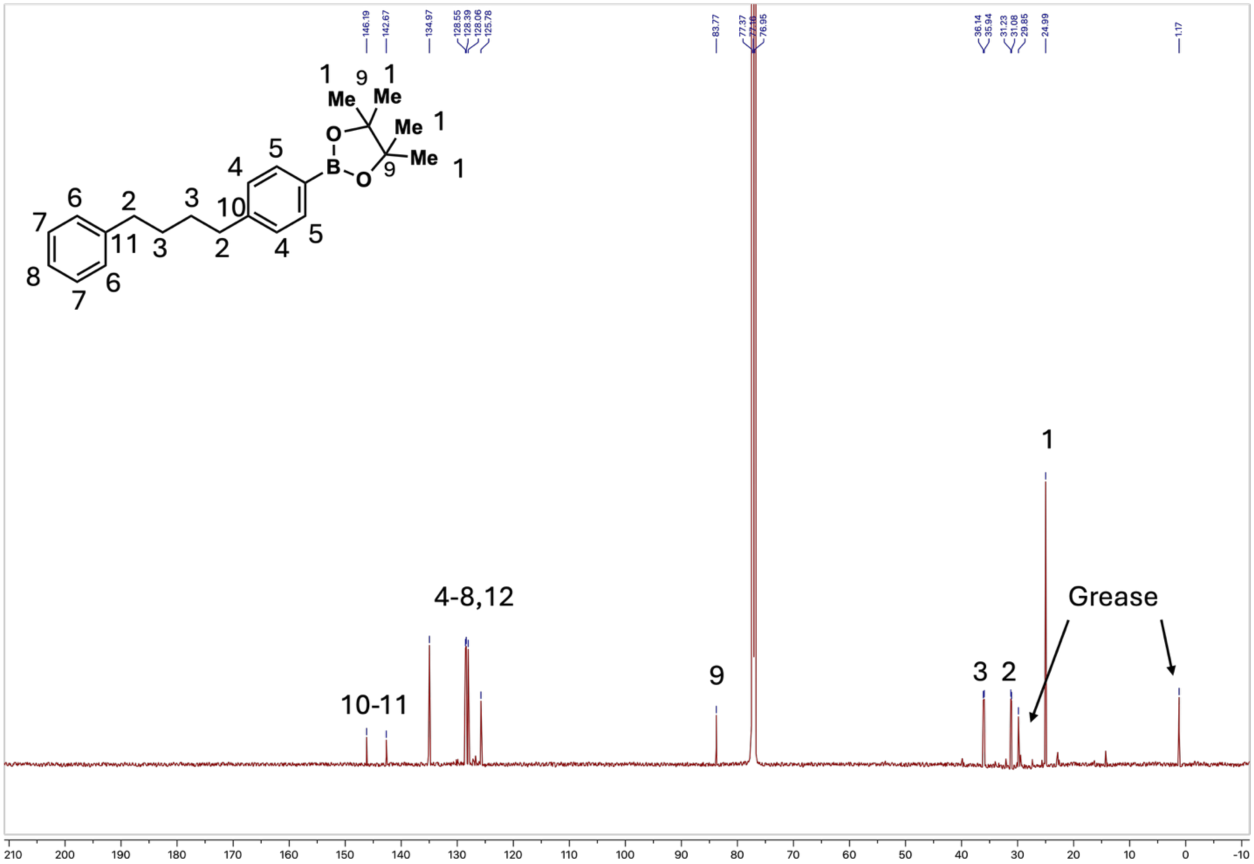

^{11}B NMR (96 MHz, $CDCl_3$) of compound **11b**

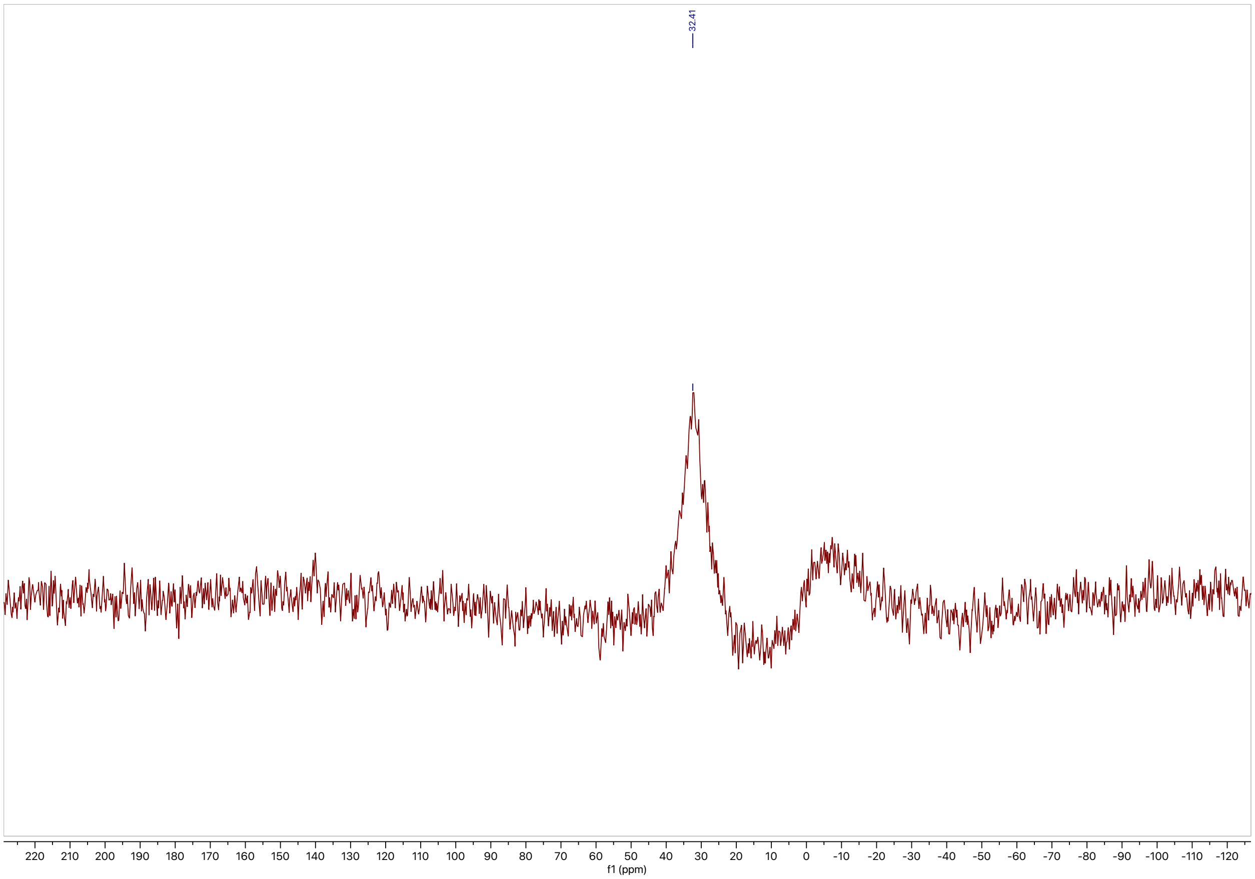

HRMS (ASAP-TOF) of compound **11b**

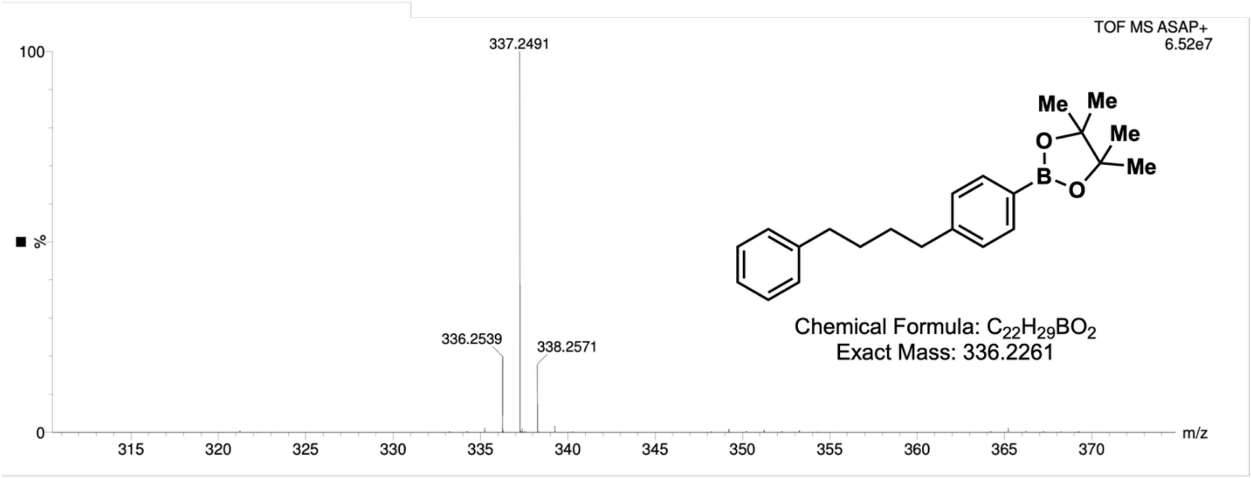

^{1}H NMR (400 MHz, $CDCl_3$) of compound **12**

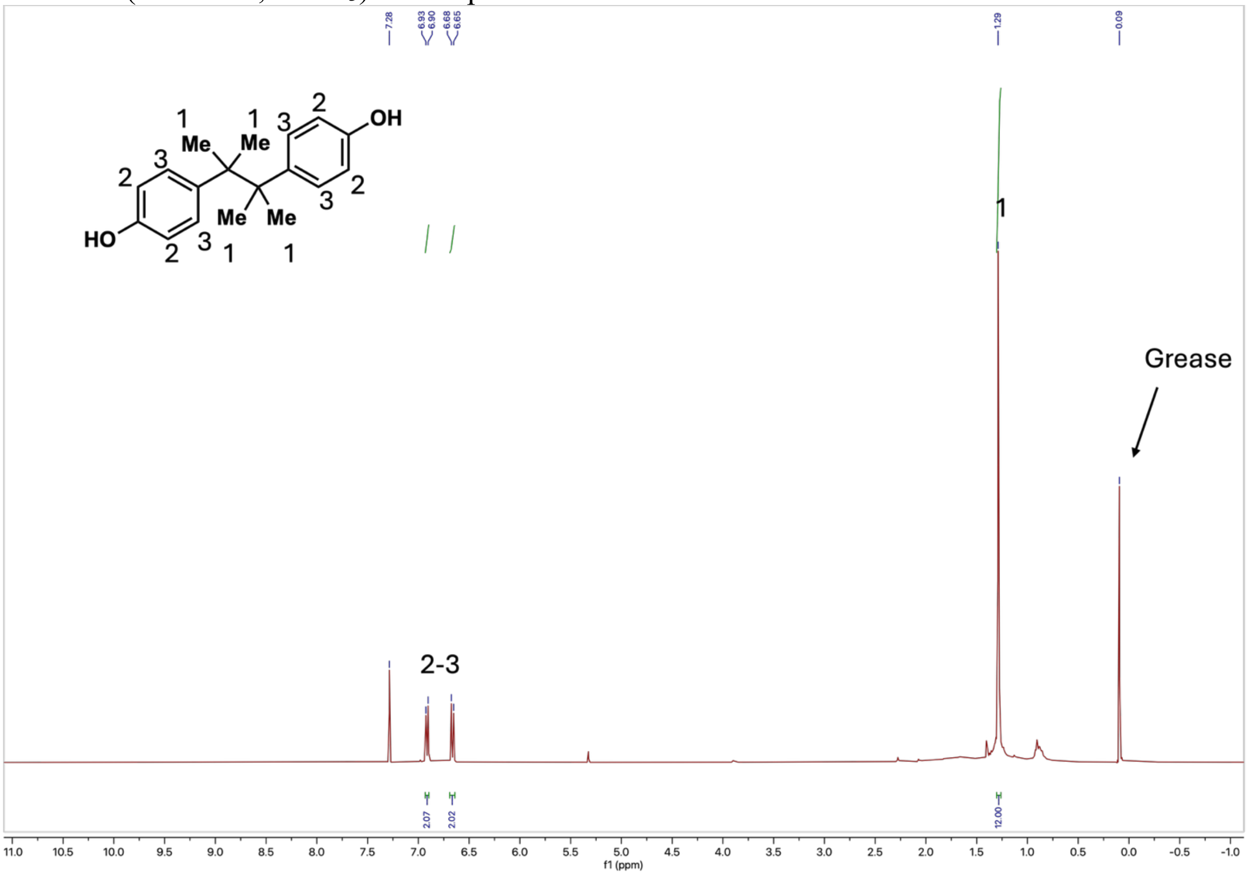

^{13}C NMR (151 MHz, $CDCl_3$) of compound **12**

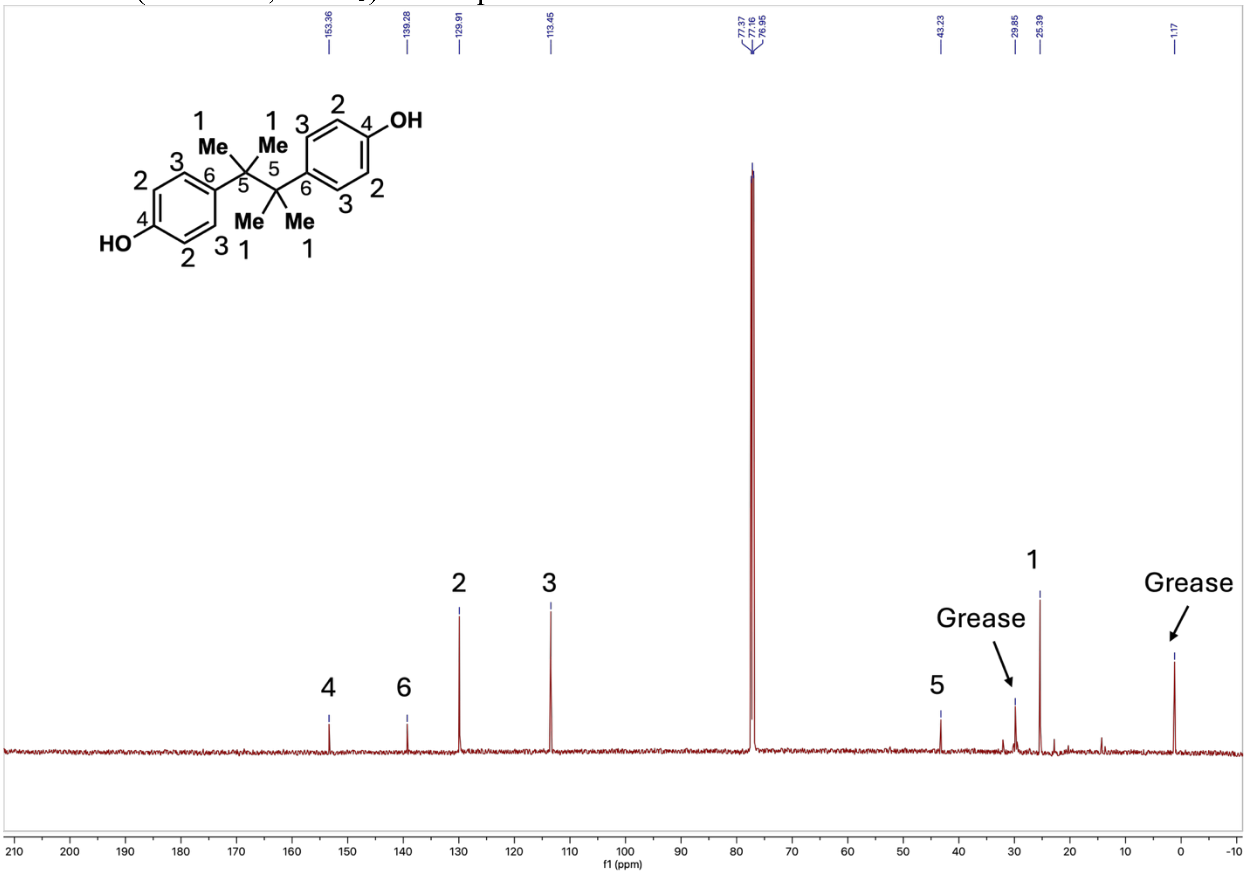

HRMS (ESI-TOF) of compound **12**

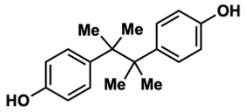

Chemical Formula: $C_{18}H_{22}O_2$
Exact Mass: 270.1620

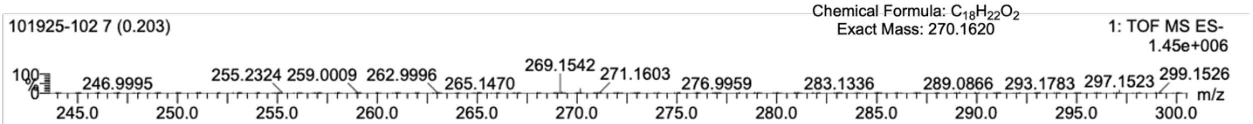

Minimum:				-1.5				
Maximum:		5.0	50.0	50.0				
Mass	Calc. Mass	mDa	PPM	DBE	i-FIT	Norm	Conf(%)	Formula
269.1542	269.1542	0.0	0.0	8.5	415.7	n/a	n/a	C18 H21 O2

^{1}H NMR (400 MHz, $CDCl_3$/MeOD) of compound **13**

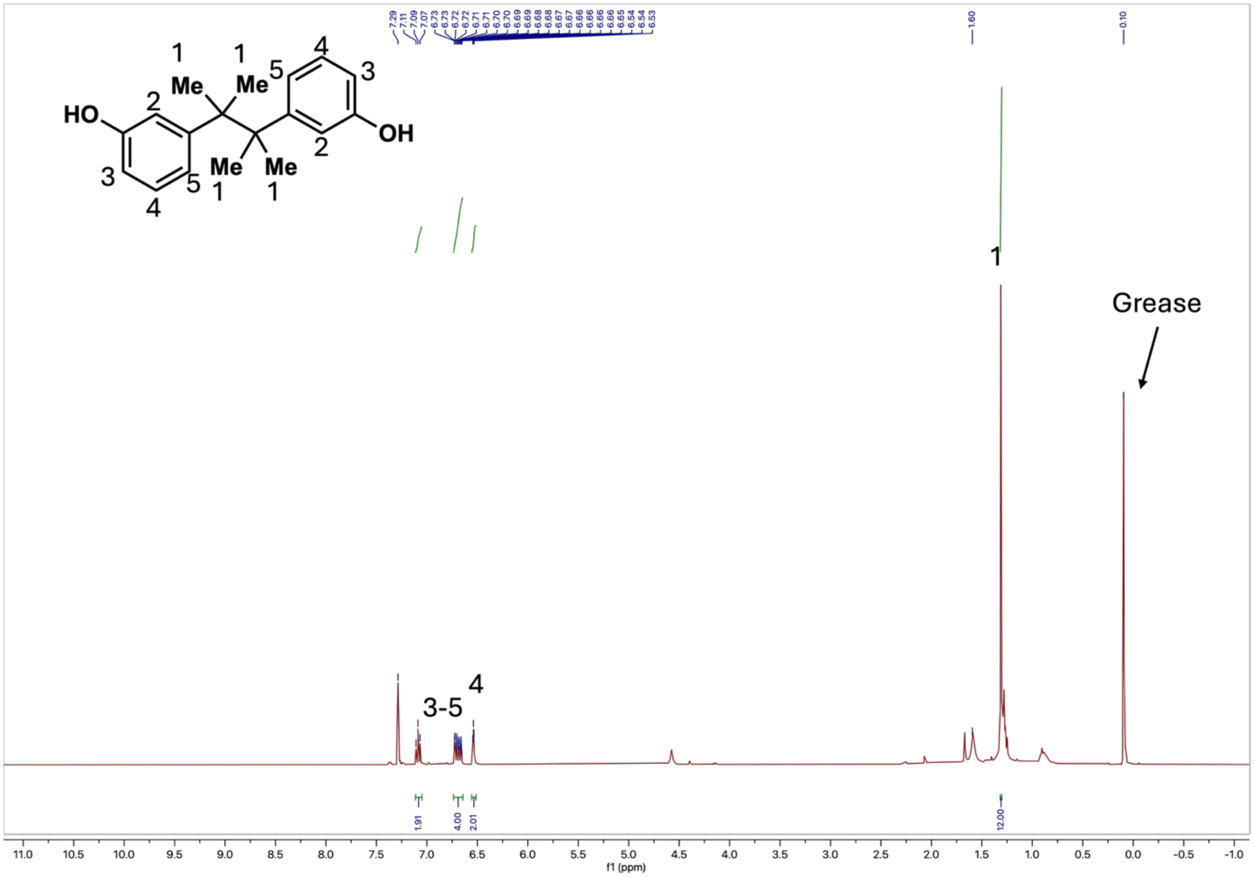

^{13}C NMR (151 MHz, $CDCl_3$/MeOD) of compound **13**

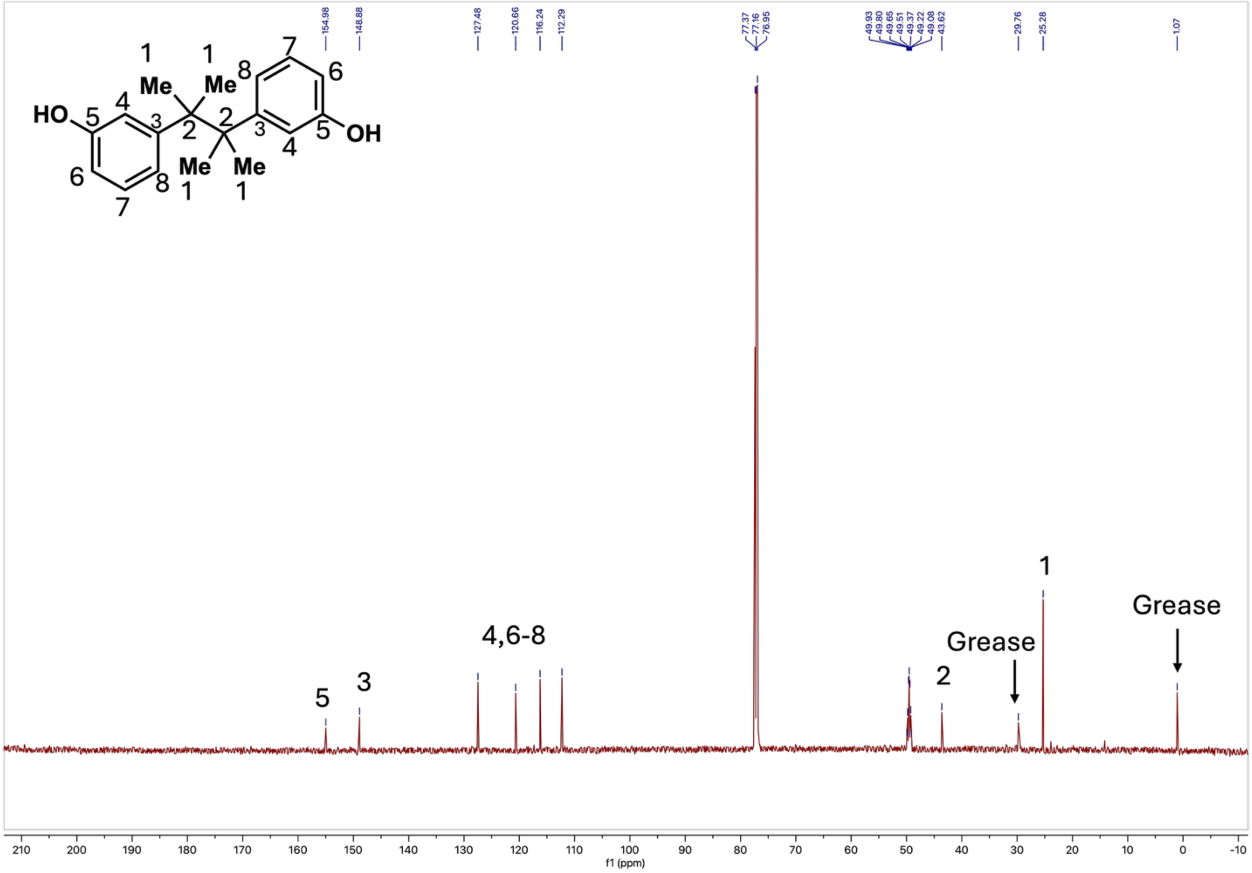

HRMS (ESI-TOF) of compound **13**

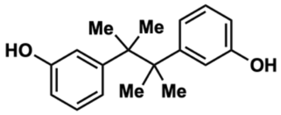

Chemical Formula: $C_{18}H_{22}O_2$
Exact Mass: 270.1620

101925-105 5 (0.140) Cm (5:6)

1: TOF MS ES-
9.59e+005

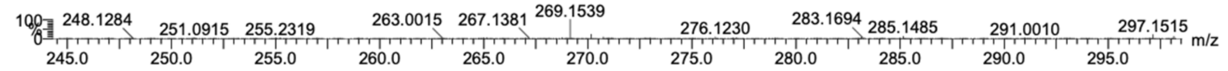

Minimum:				-1.5				
Maximum:		5.0	50.0	50.0				
Mass	Calc. Mass	mDa	PPM	DBE	i-FIT	Norm	Conf(%)	Formula
269.1539	269.1542	-0.3	-1.1	8.5	631.3	n/a	n/a	C18 H21 O2

^1H NMR (600 MHz, $CDCl_3$) of compound **14**

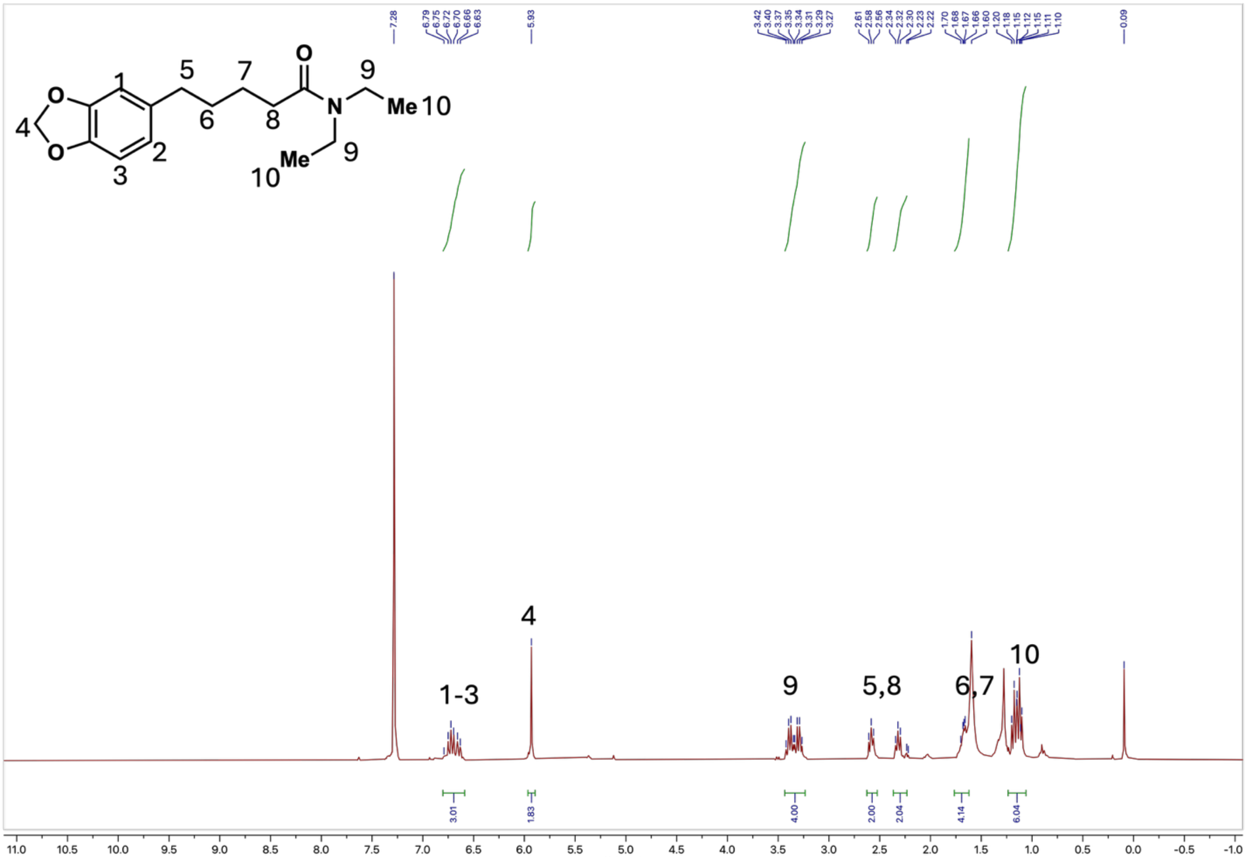

^{13}C NMR (151 MHz, $CDCl_3$) of compound **14**

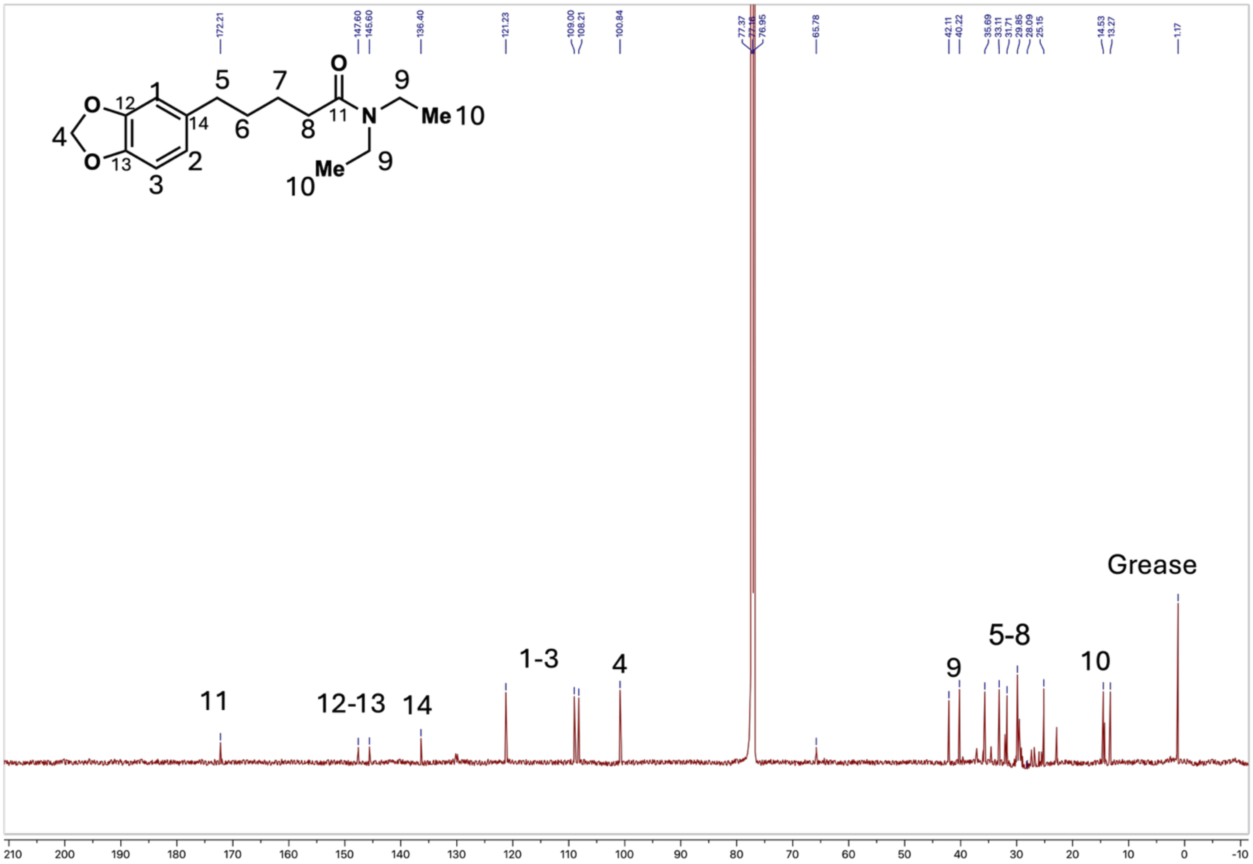

GC-MS (EI) of compound **14**

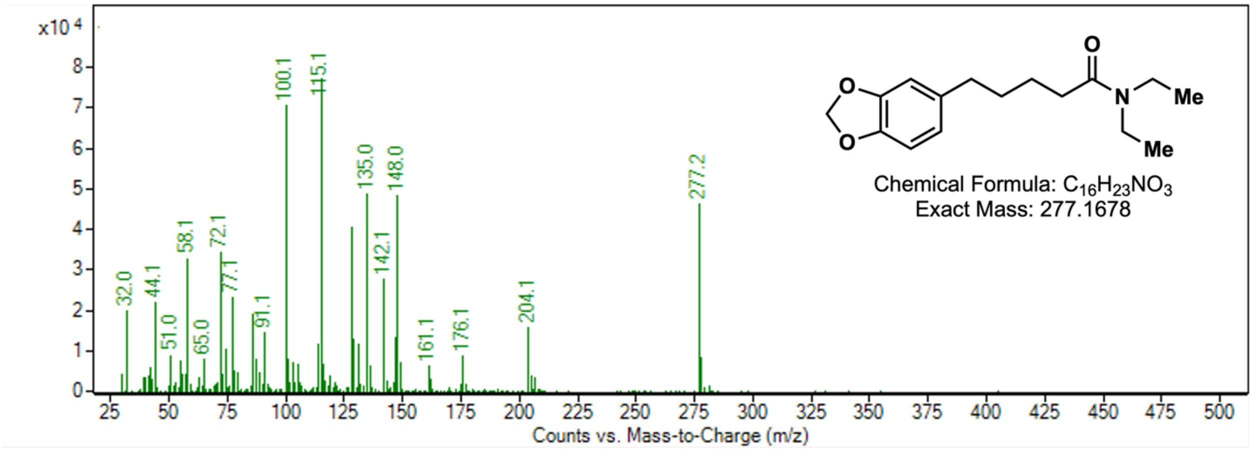

^{1}H NMR (300 MHz, $CDCl_3$) of compound **15**

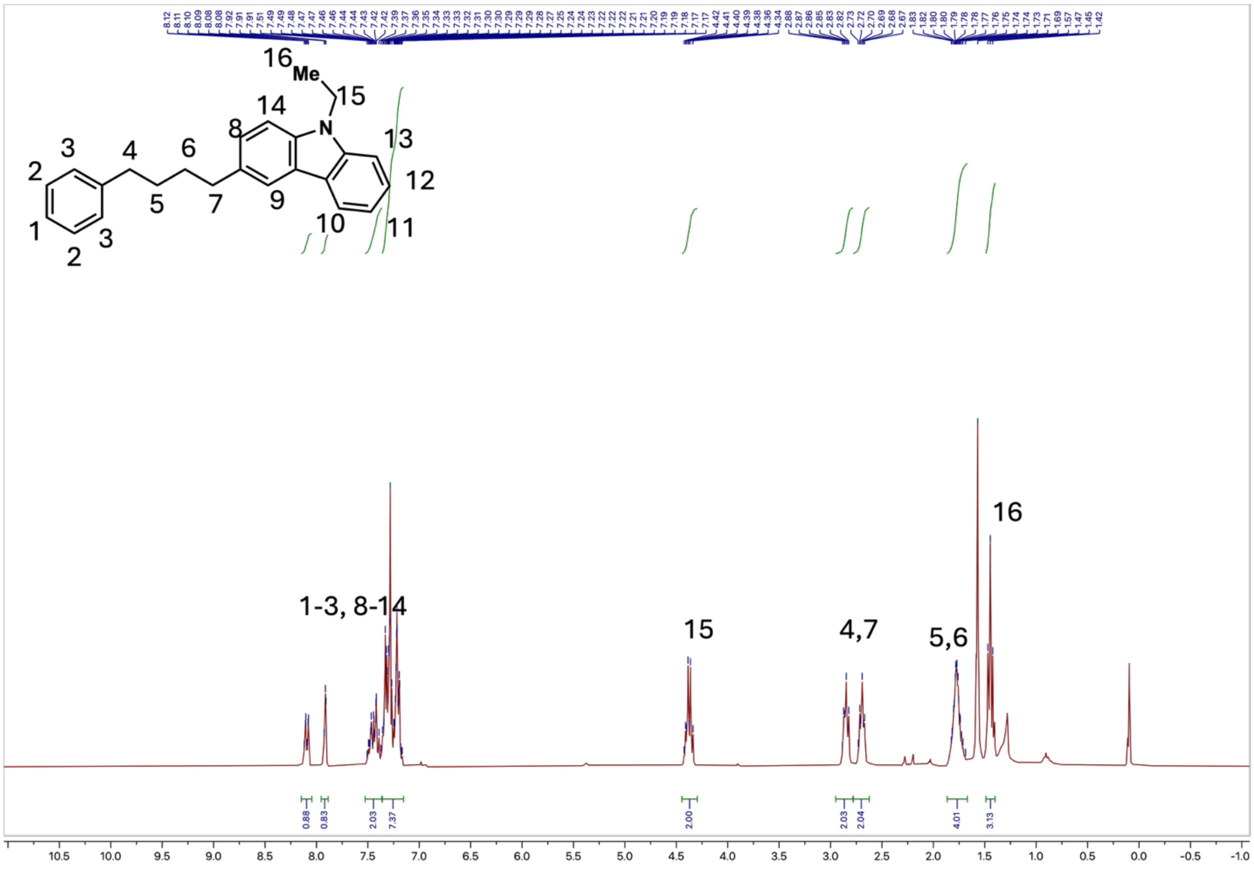

^{13}C NMR (126 MHz, $CDCl_3$) of compound **15**

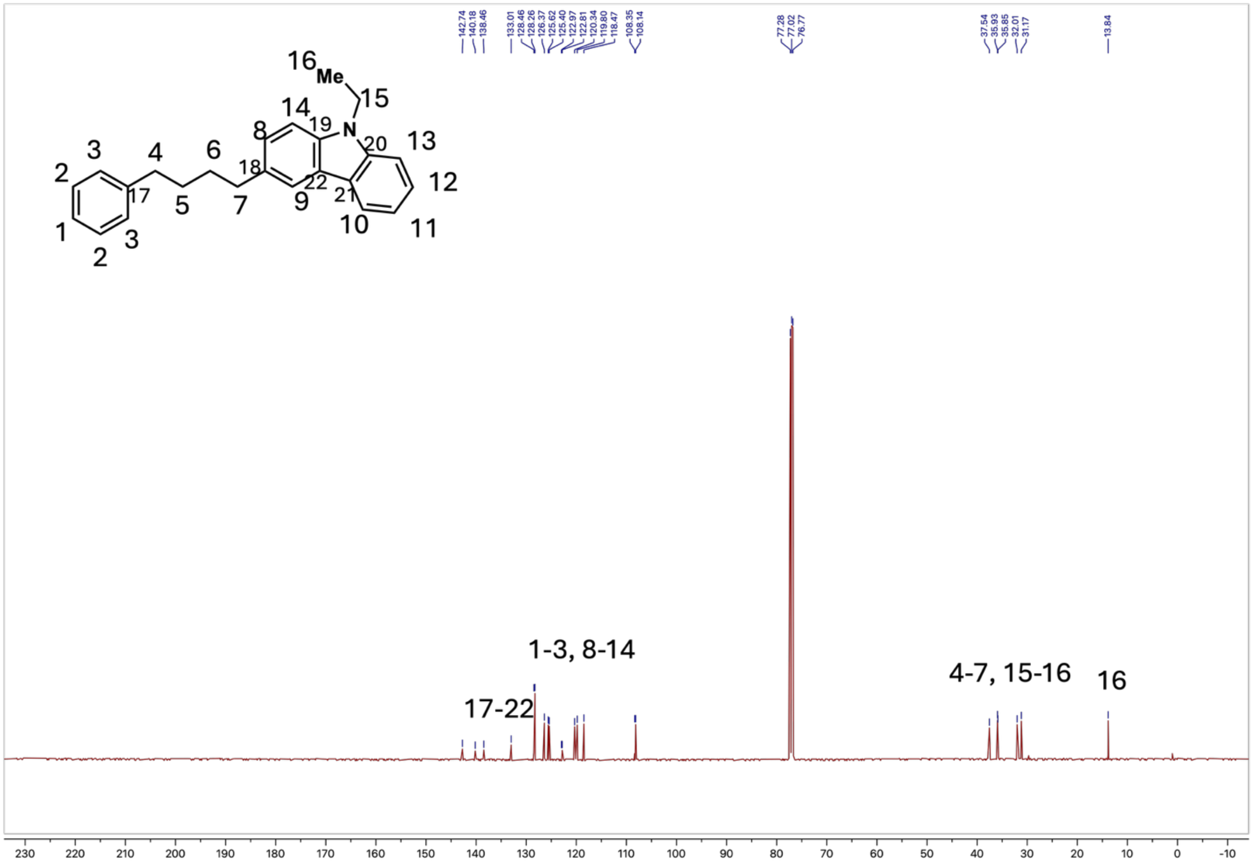

HRMS (ASAP-TOF) of compound **15**

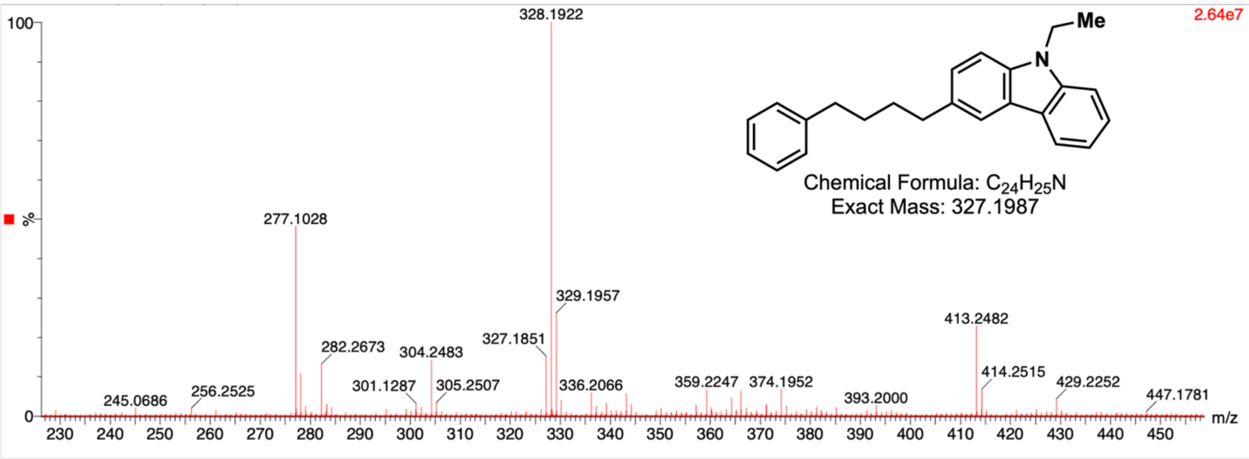

^1H NMR (400 MHz, $CDCl_3$) of compound **16**

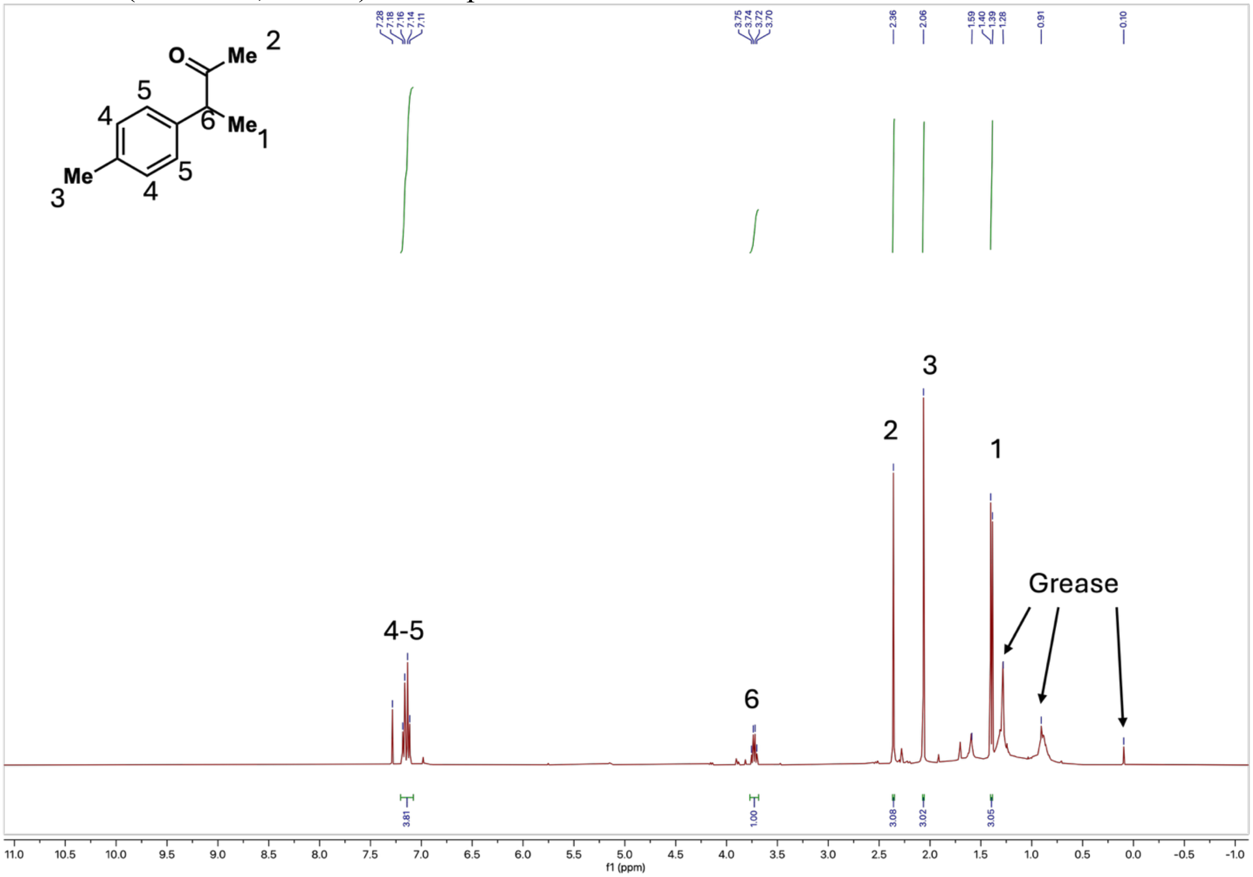

^{13}C NMR (151 MHz, $CDCl_3$) of compound **16**

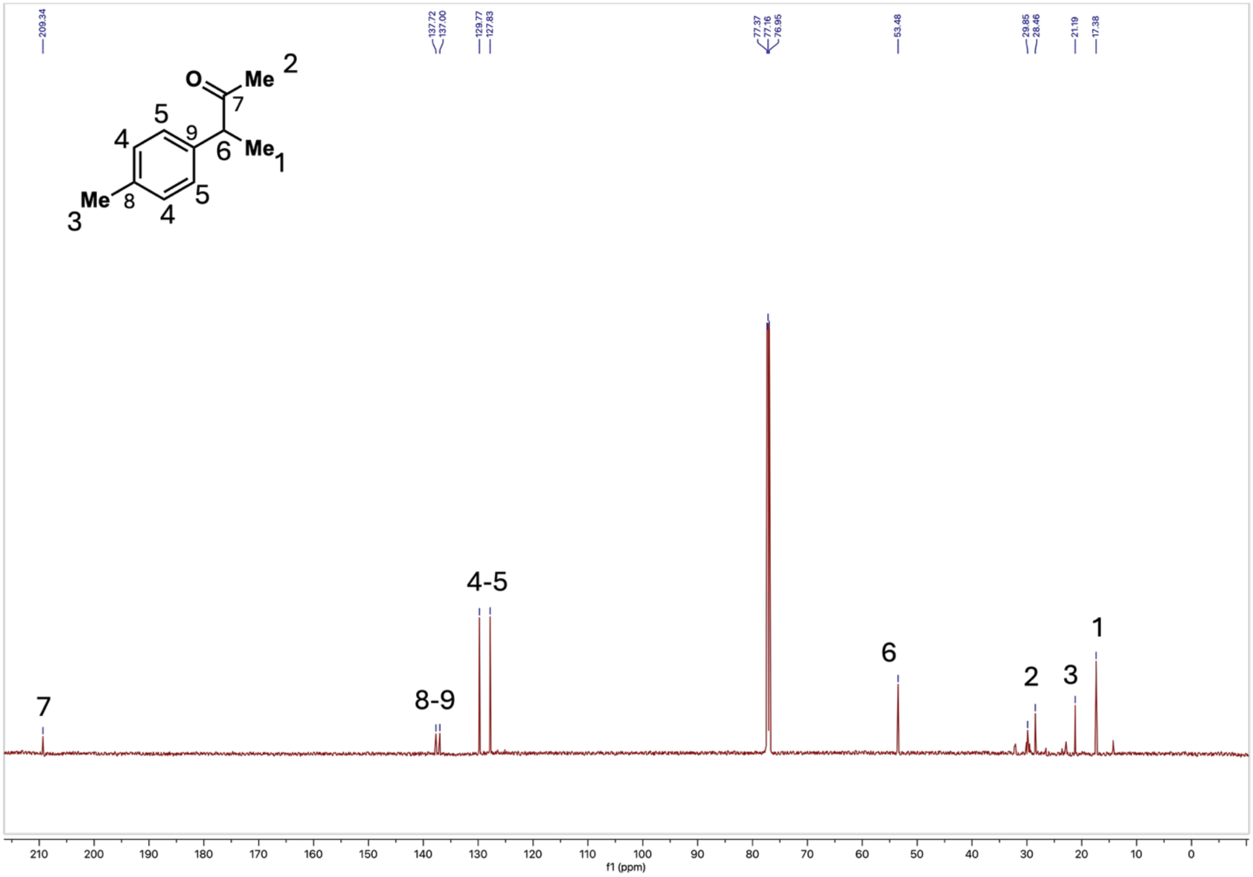

GC-MS (EI) of compound **16**

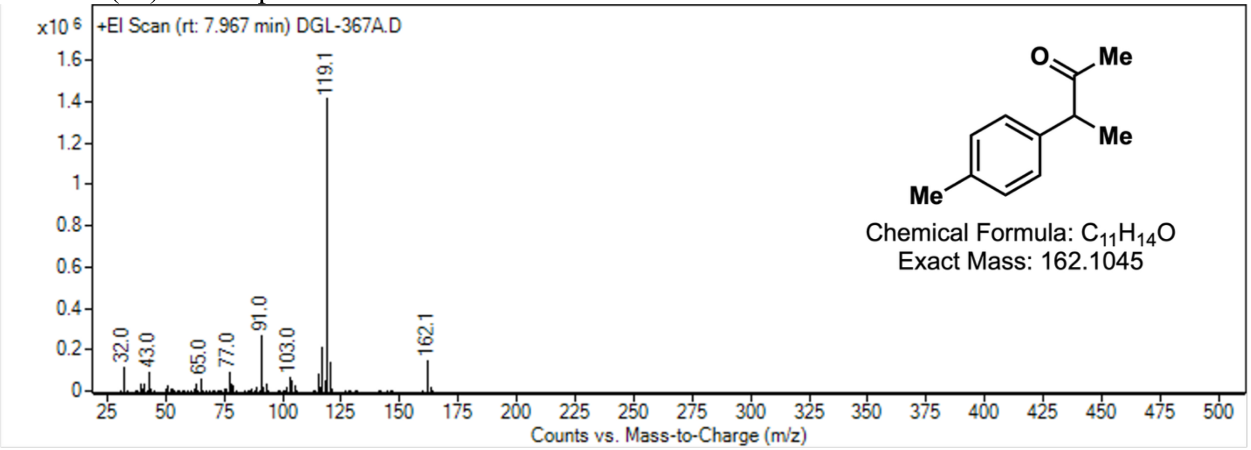

^{1}H NMR (400 MHz, $CDCl_3$) of compound **17**

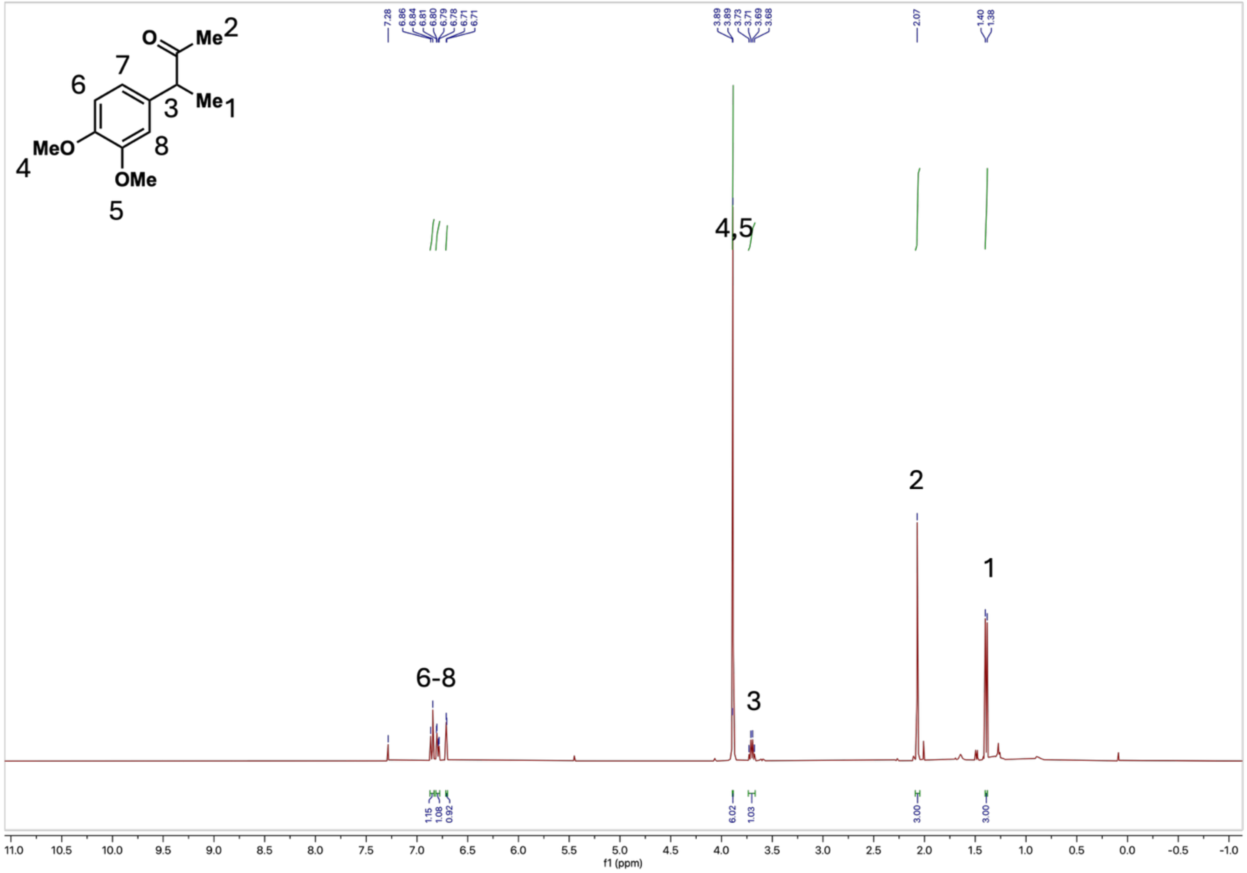

^{13}C NMR (151 MHz, $CDCl_3$) of compound **17**

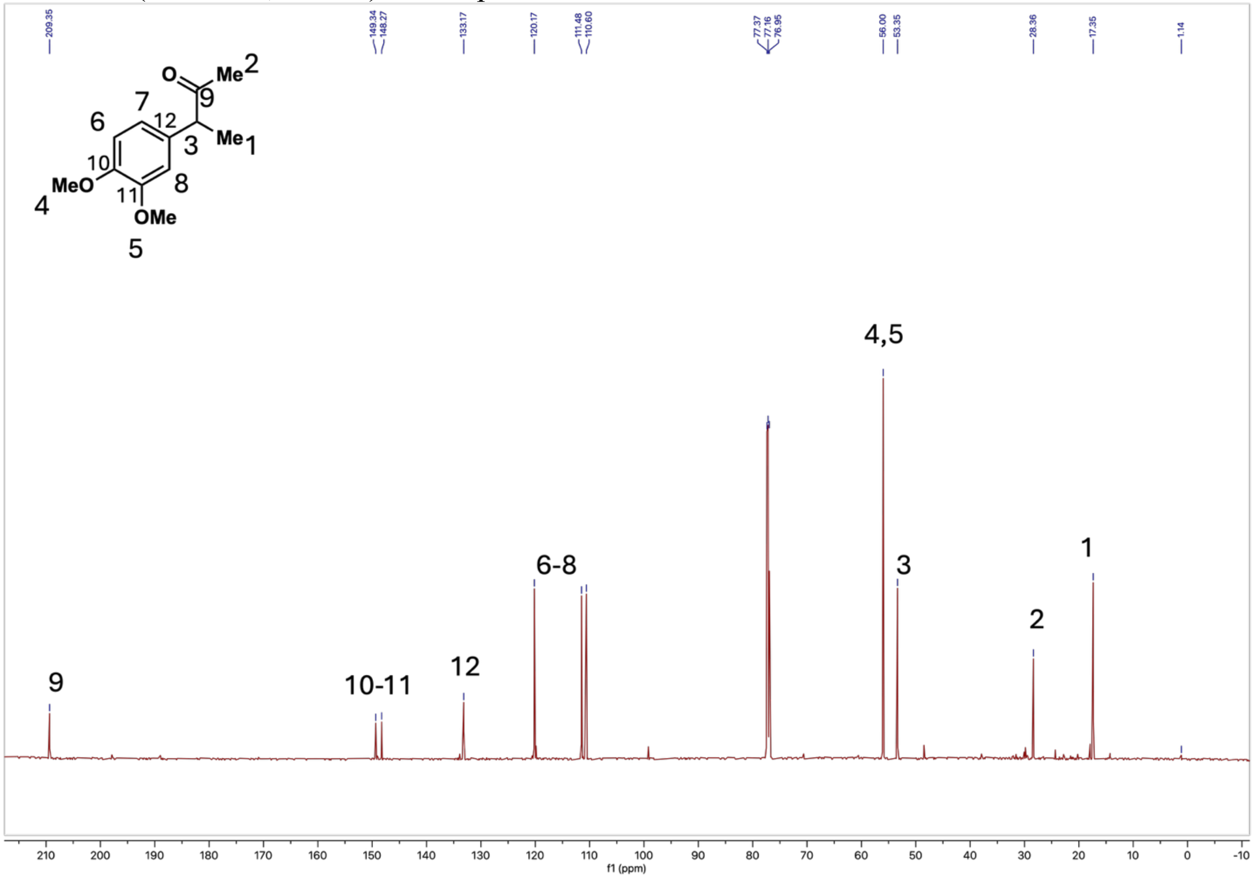

GC-MS (EI) of compound **17**

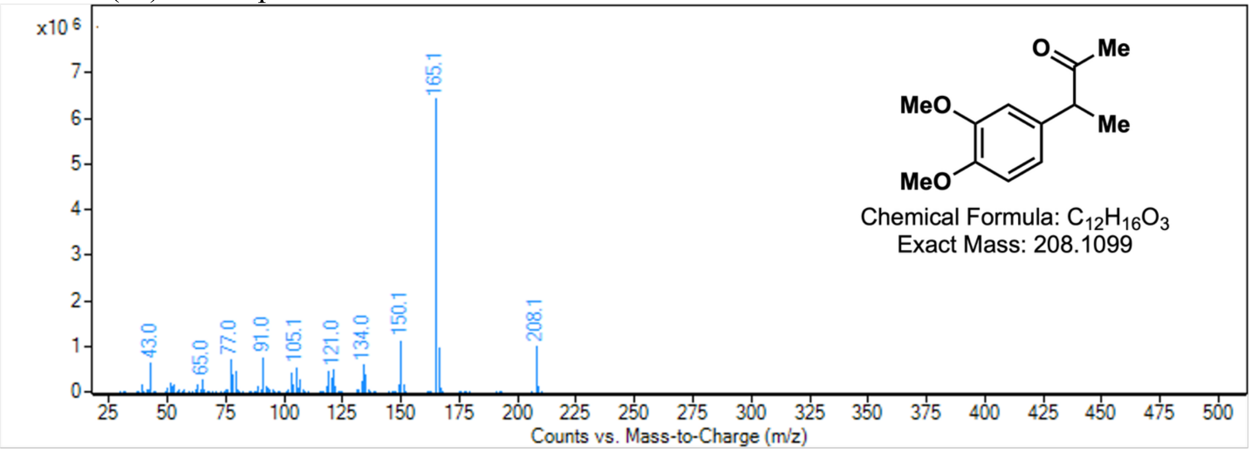

^{1}H NMR (500 MHz, $CDCl_3$) of compound **18**

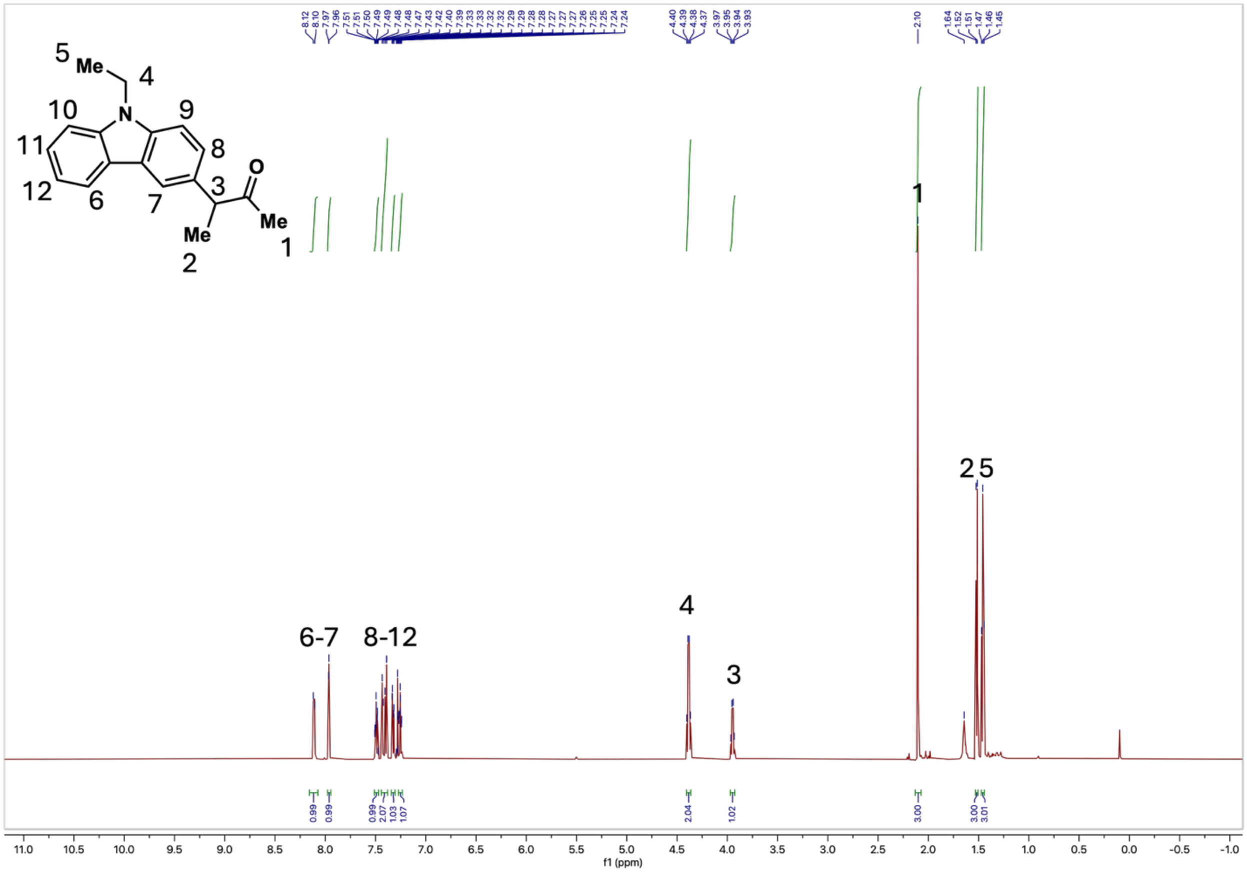

^{13}C NMR (151 MHz, $CDCl_3$) of compound **18**

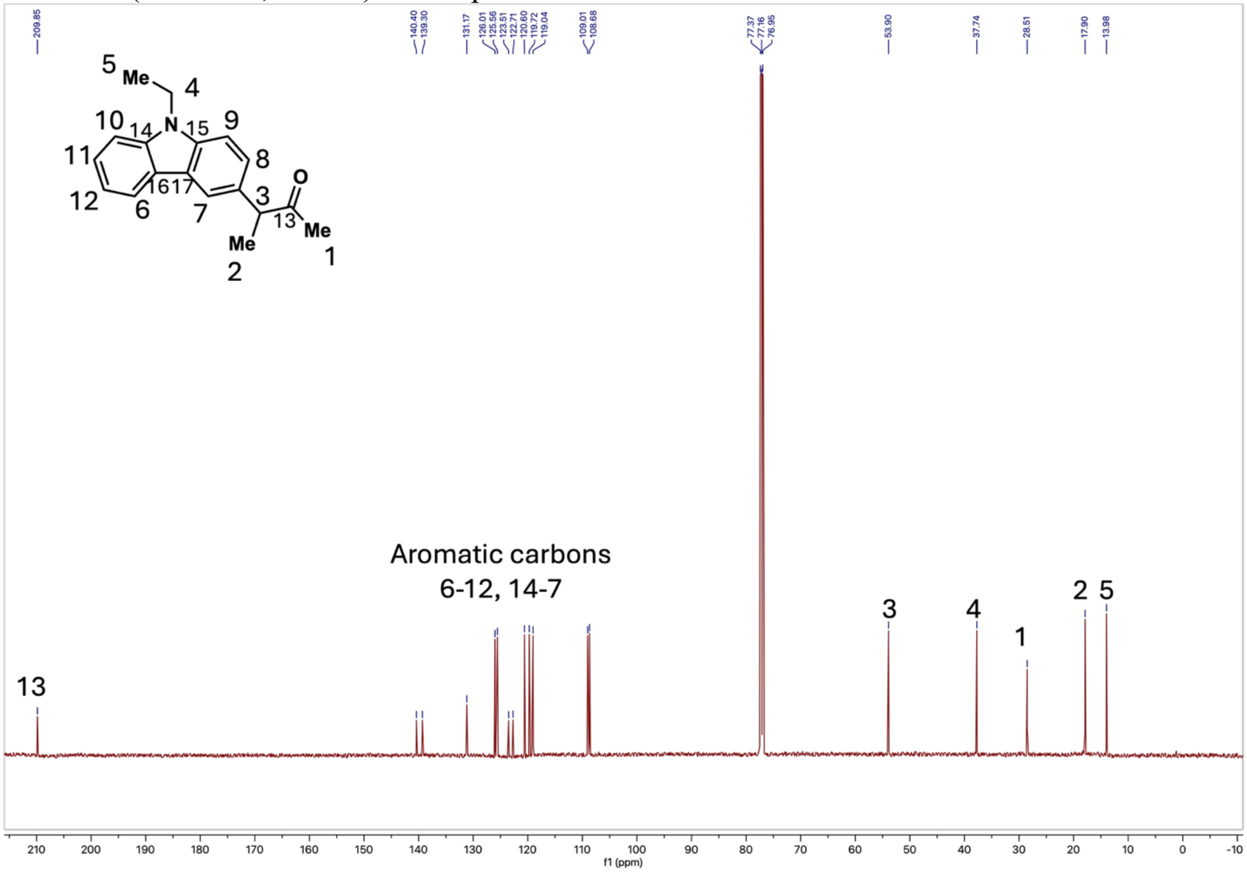

HRMS (ESI-TOF) of compound **18**

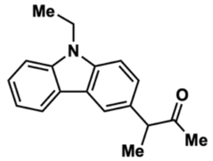

Chemical Formula: $C_{18}H_{19}NO$
Exact Mass: 265.1467

C: 18-18 H: 10-22 N: 1-1 O: 1-7 Na: 0-1
27-Oct-2025 11:38:51
102725-112 8 (0.229) Cm (7:12)

1: TOF MS ES+
1.48e+007

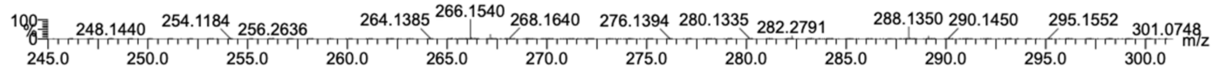

		mDa	PPM	DBE				
Minimum:				-1.5				
Maximum:		5.0	5.0	50.0				
Mass	Calc. Mass	mDa	PPM	DBE	i-FIT	Norm	Conf(%)	Formula
266.1540	266.1545	-0.5	-1.9	9.5	584.7	n/a	n/a	C18 H20 N O

^{1}H NMR (800 MHz, $CDCl_3$) of compound **19**

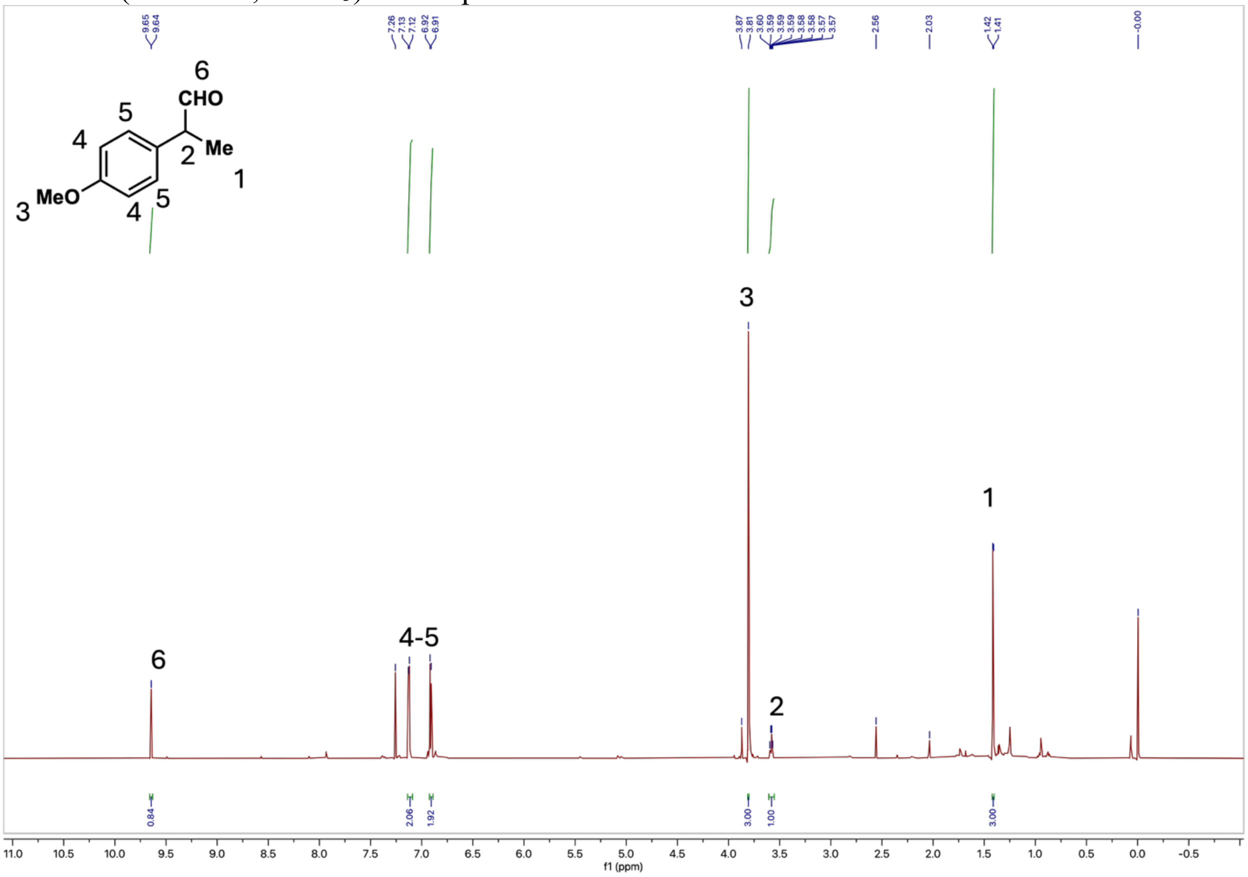

^{13}C NMR (201 MHz, CDCl$_3$) of compound **19**

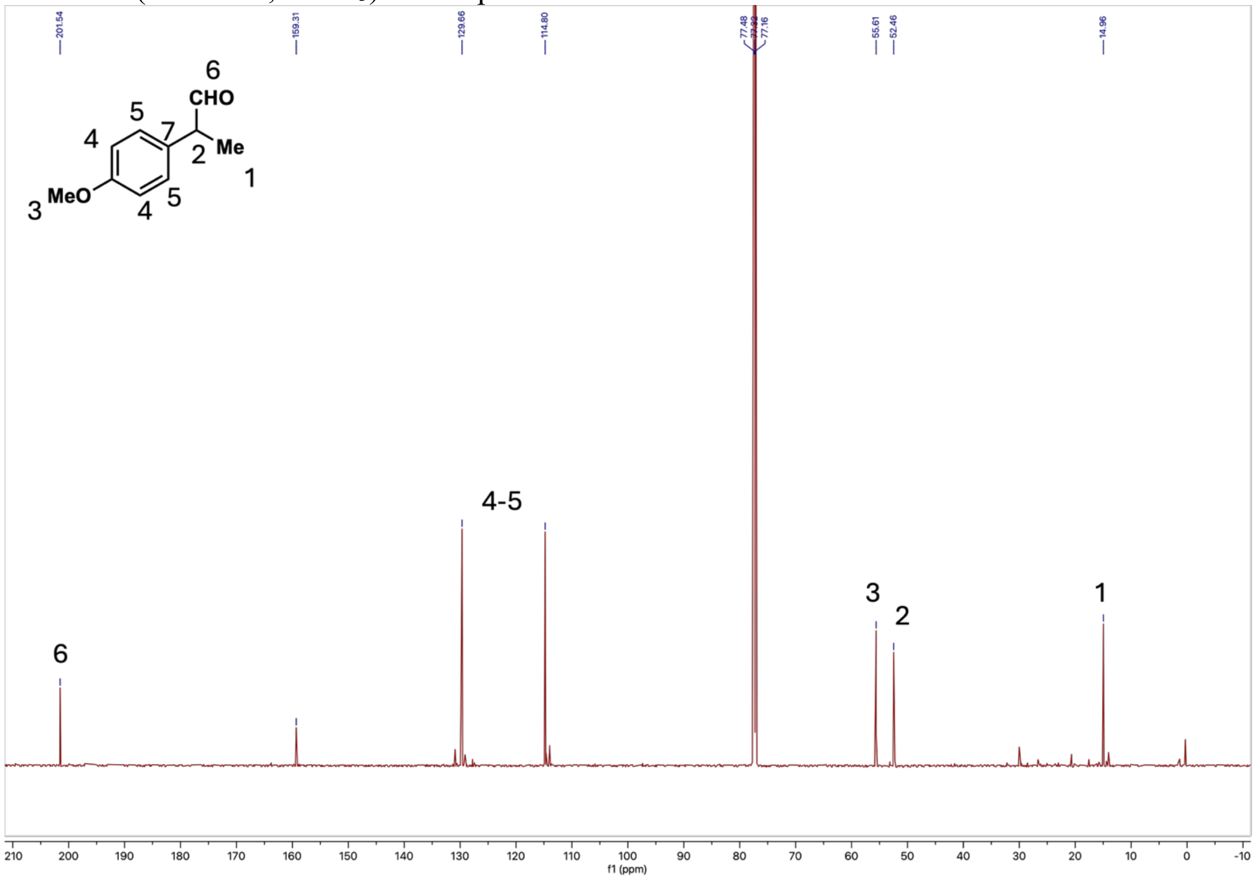

GC-MS (EI) of compound **19**

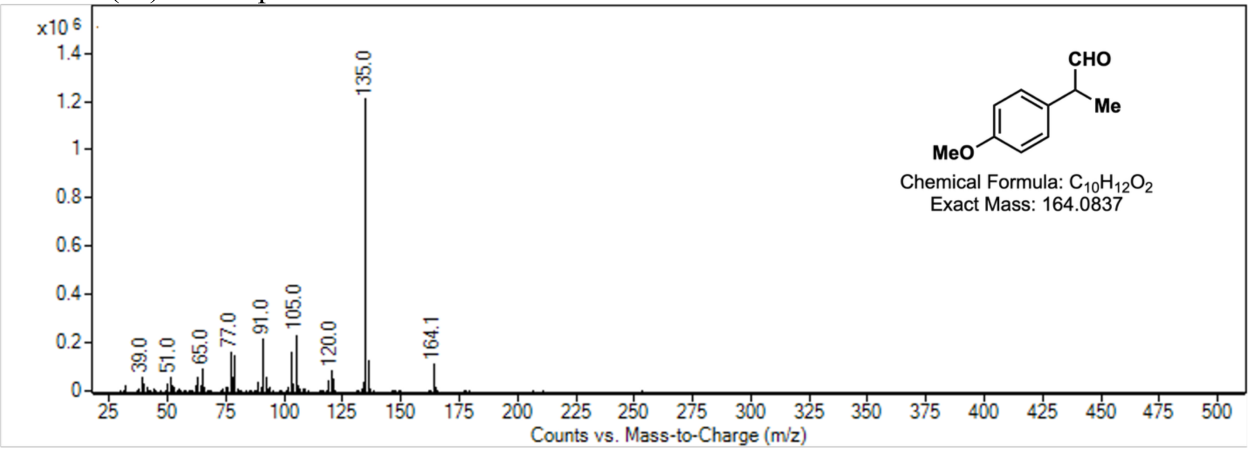

^{1}H NMR (500 MHz, $CDCl_3$) of compound **3a**

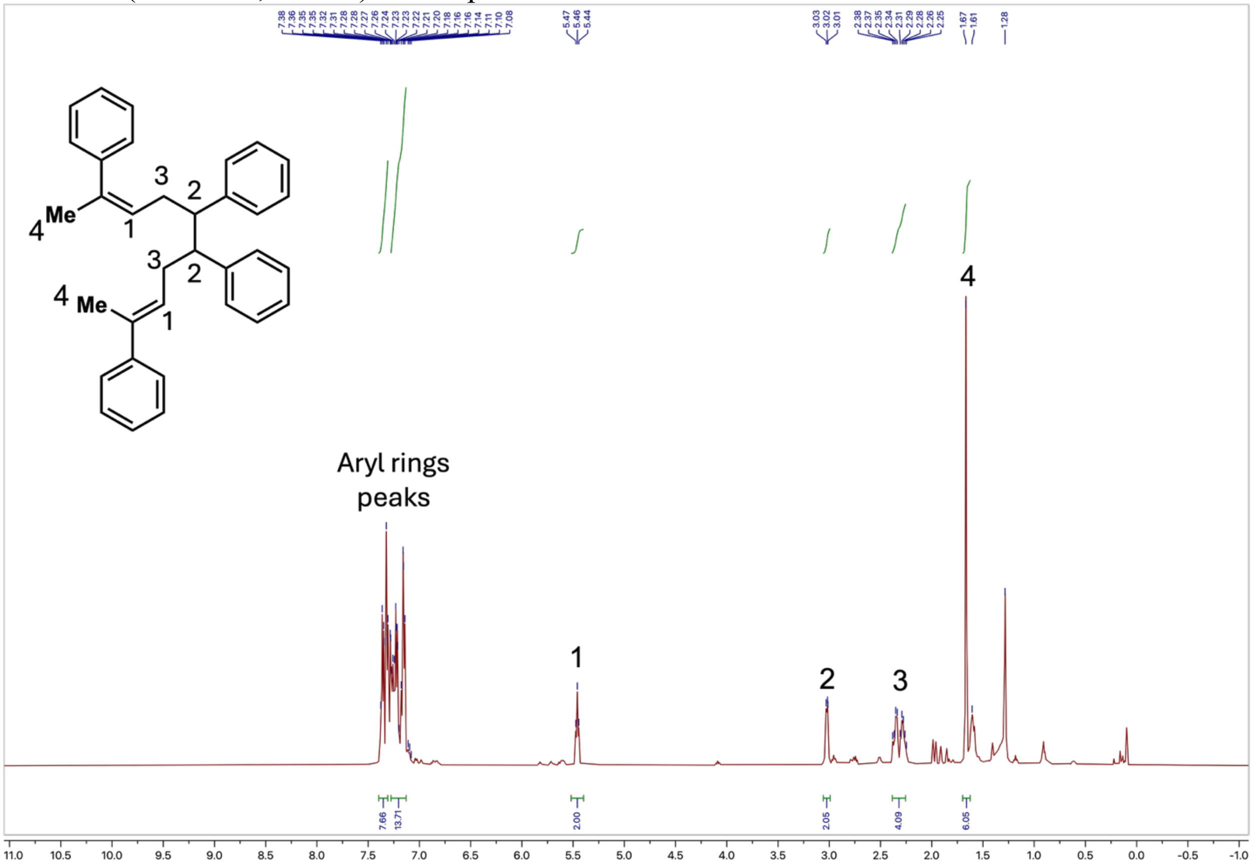

^{13}C NMR (151 MHz, $CDCl_3$) of compound **3a**

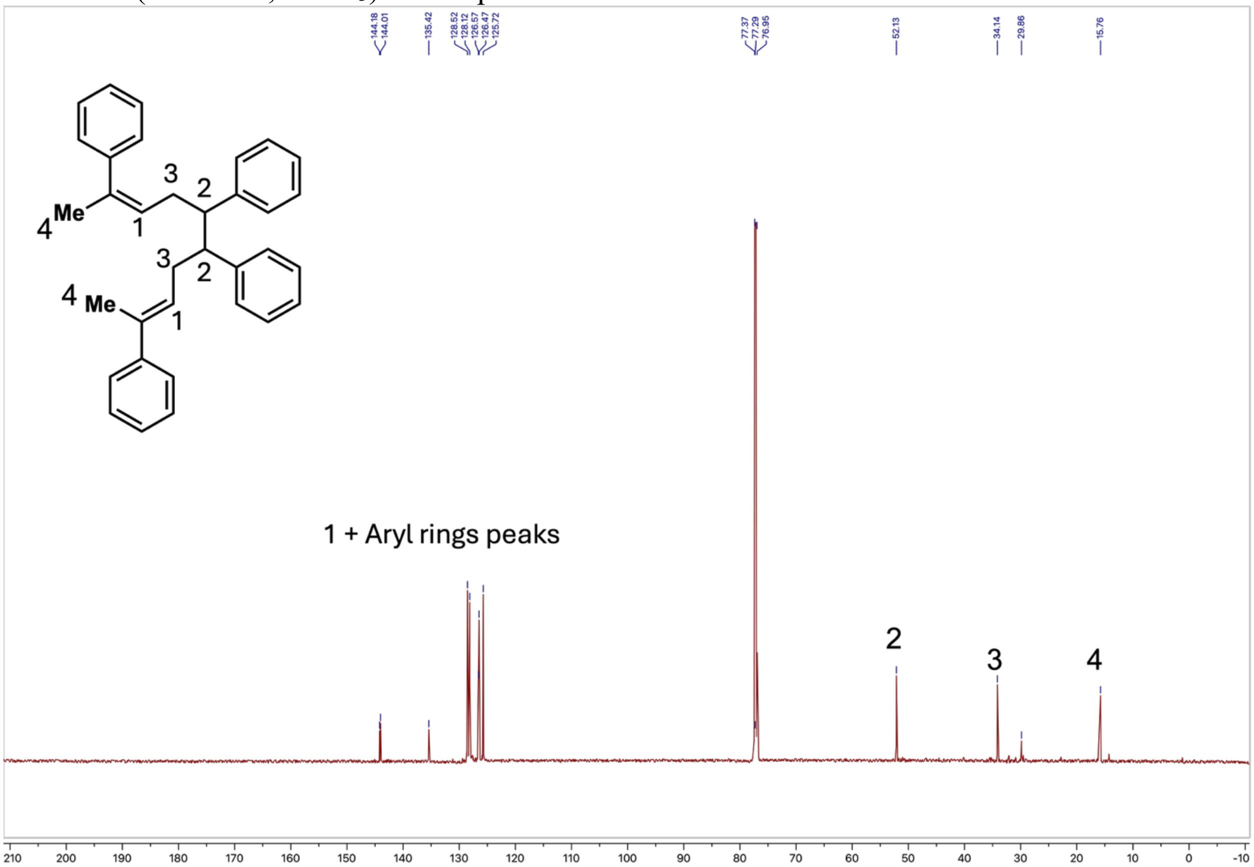

HRMS (ESI-TOF) of compound **3a**

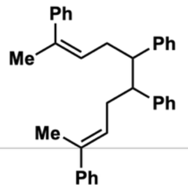

Monoisotopic Mass, Even Electron Ions
1 formula(e) evaluated with 1 results within limits (up to 10 closest results for each mass)
Elements Used:
C: 34-34 H: 30-40 Na: 0-1

Chemical Formula: $C_{34}H_{34}$
Exact Mass: 442.2661

15-Oct-2025 14:29:27
101525-116 9 (0.255) Cm (2:24)

DGL-360A F0 P2 Daniel Gordon

1: TOF MS ES+
9.79e+005

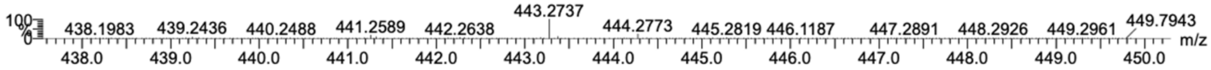

Minimum:				-1.5				
Maximum:		5.0	5.0	50.0				
Mass	Calc. Mass	mDa	PPM	DBE	i-FIT	Norm	Conf(%)	Formula
443.2737	443.2739	-0.2	-0.5	17.5	779.8	n/a	n/a	C34 H35

^{1}H NMR (800 MHz, $CDCl_3$) of compound **3b**

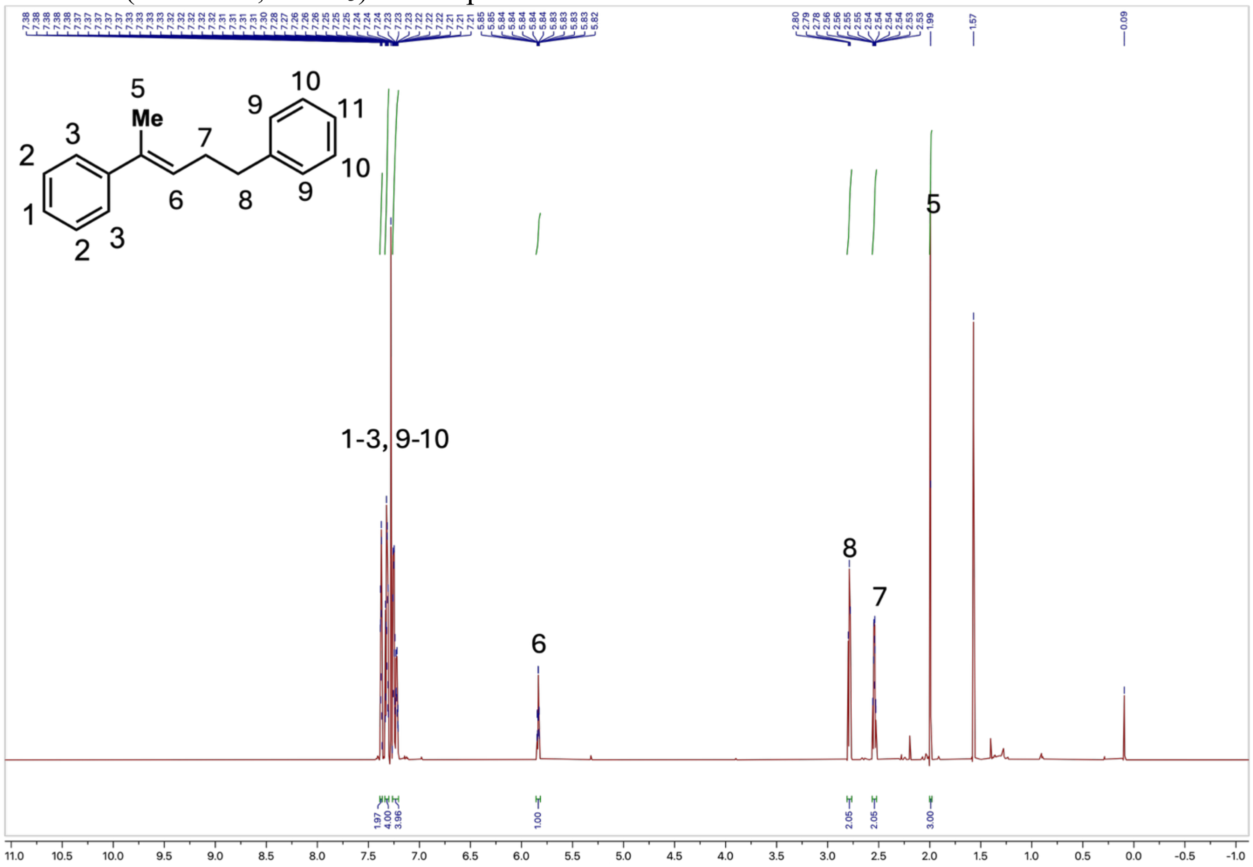

^{13}C NMR (151 MHz, $CDCl_3$) of compound **3b**

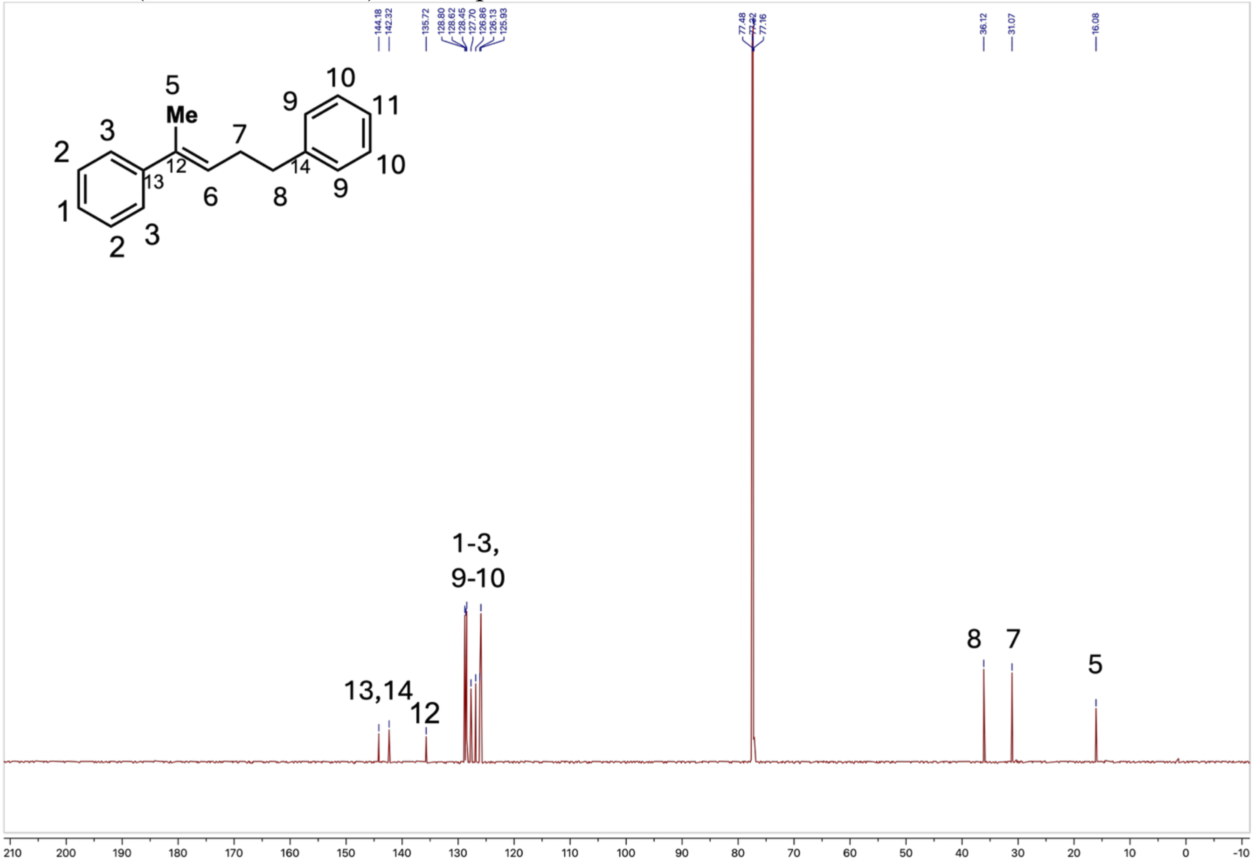

GC-MS (EI) of compound **3b**

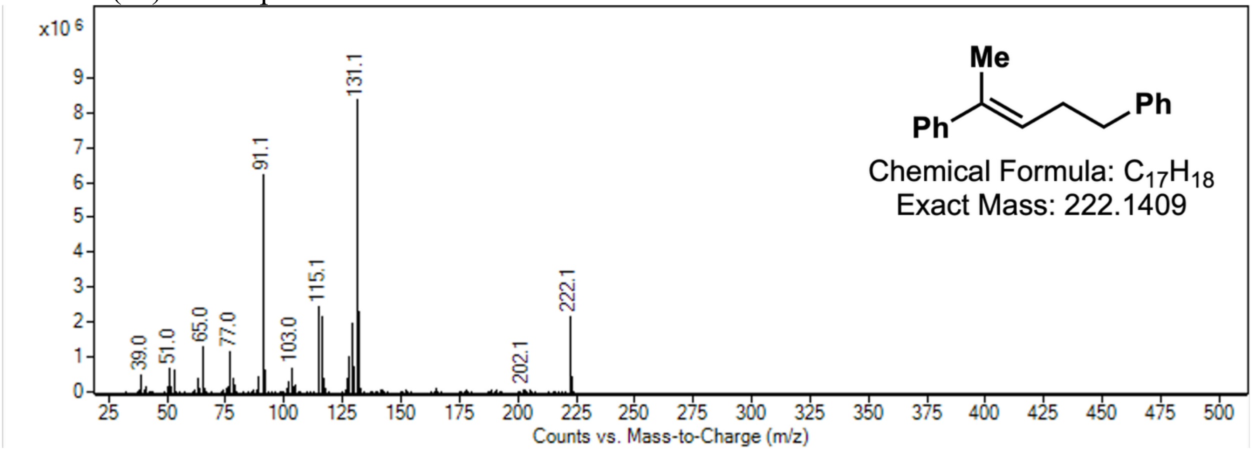

^{1}H NMR (600 MHz, $CDCl_3$) of compound **2e**

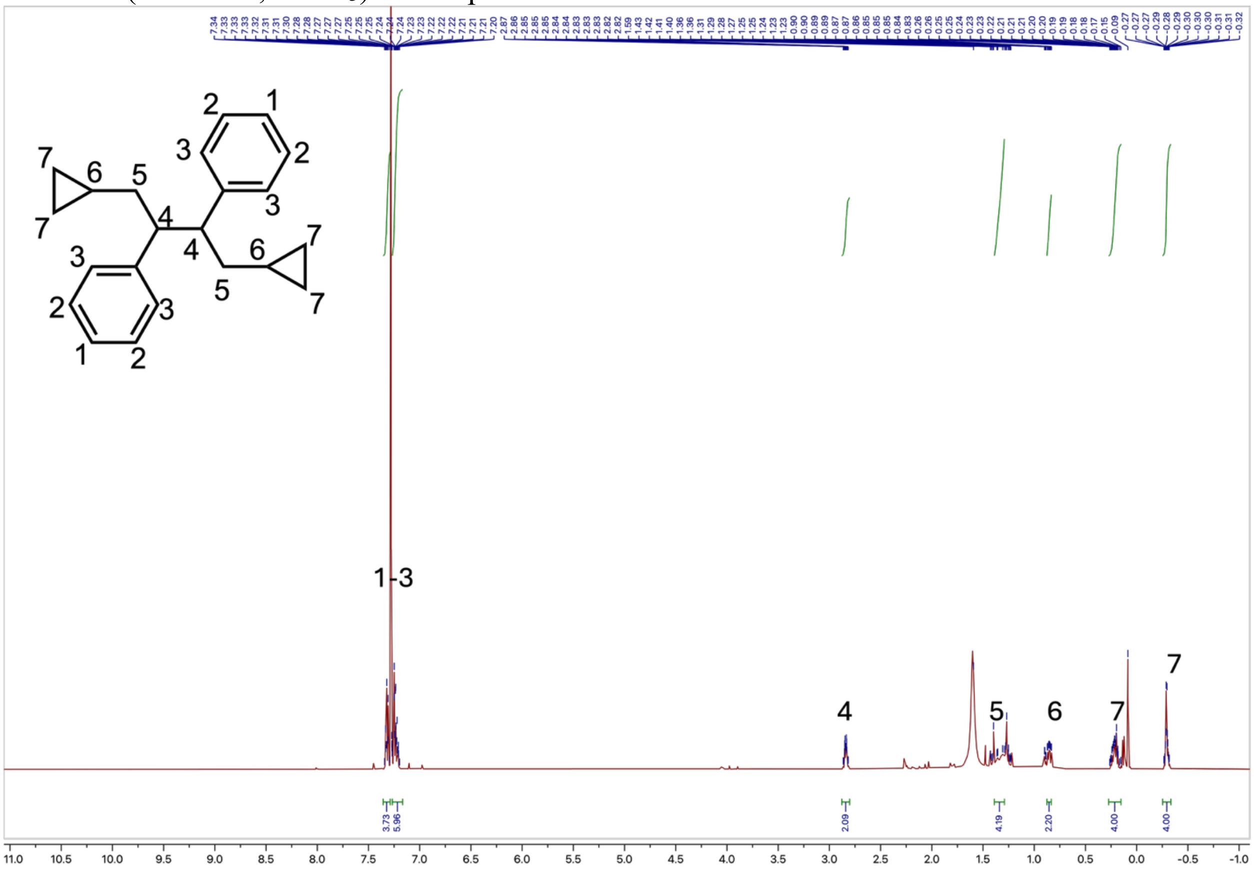

^{13}C NMR (151 MHz, $CDCl_3$) of compound **2e**

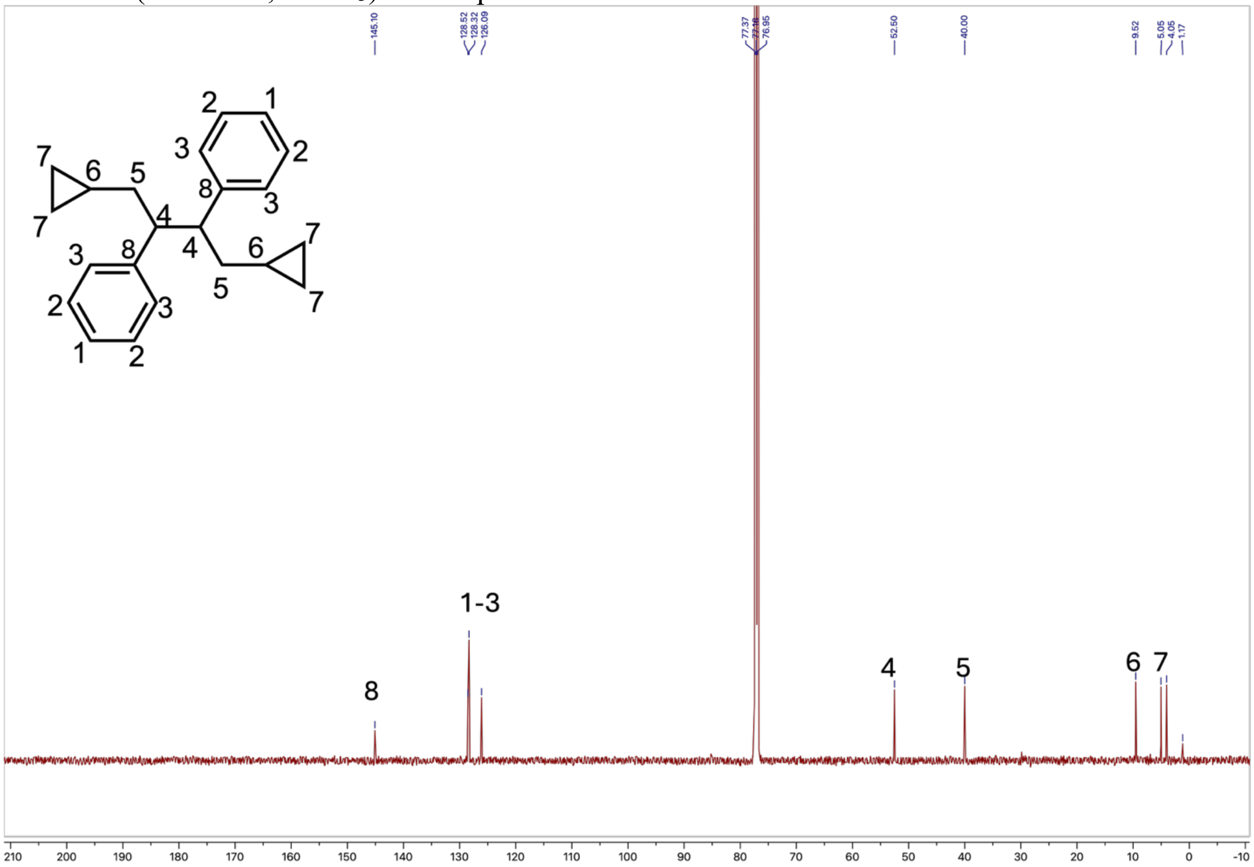

GC-MS (EI) of compound **2e**

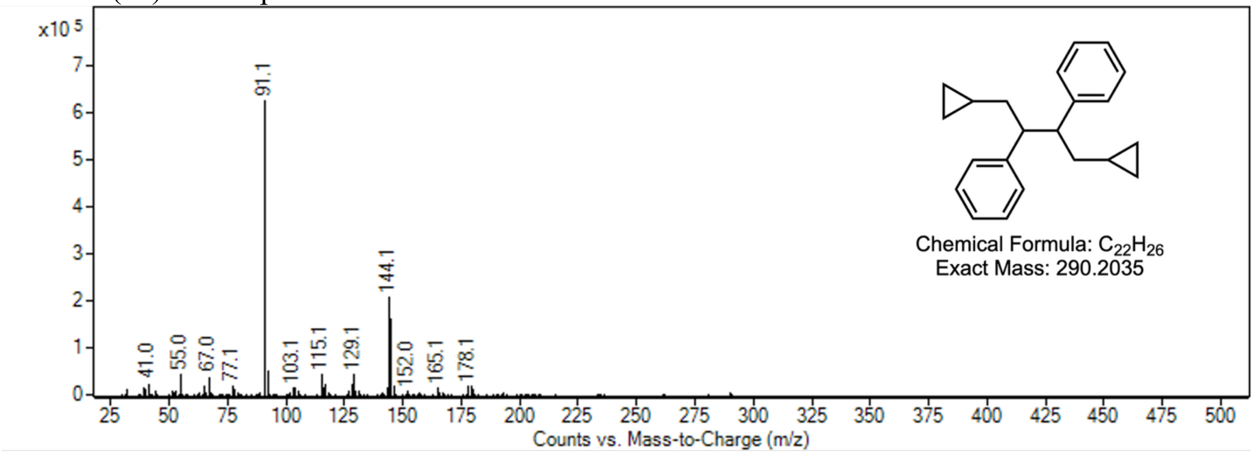

^{1}H NMR (300 MHz, $CDCl_3$) of compound **2c**

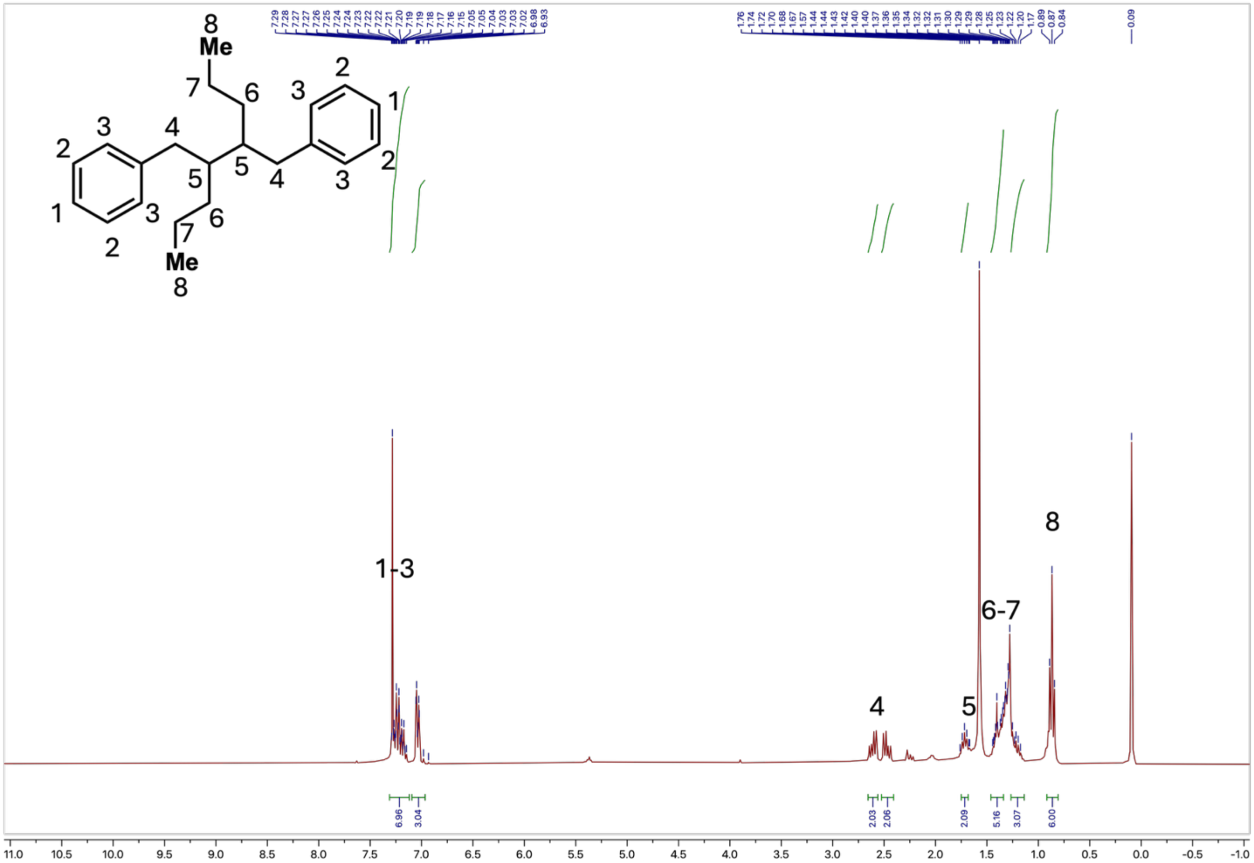

^{13}C NMR (151 MHz, $CDCl_3$) of compound **2c**

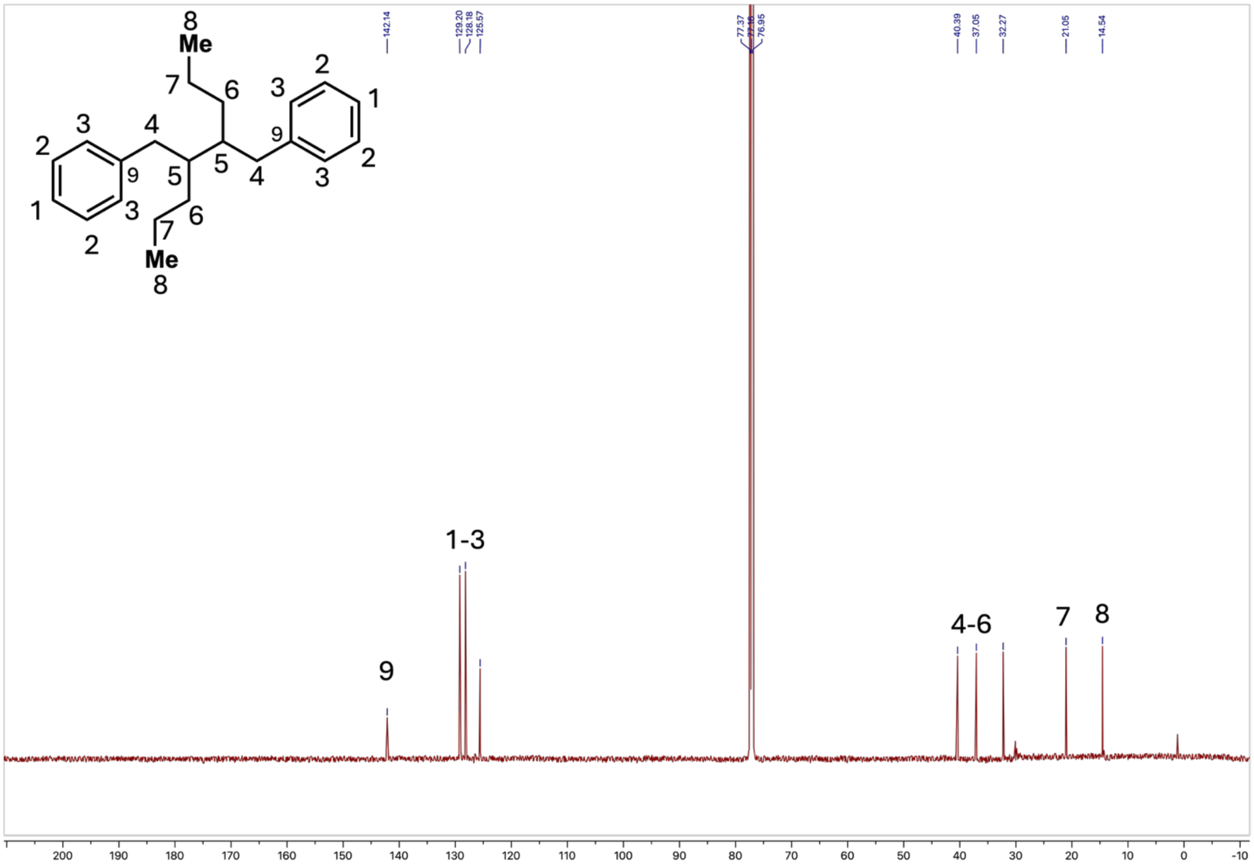

GC-MS (EI) of compound **2c**

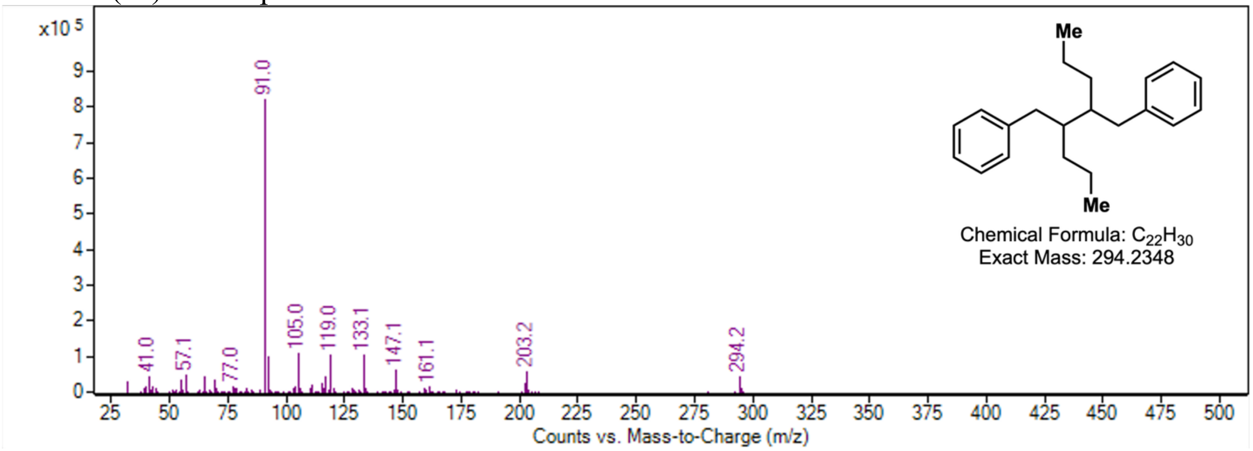

7. References

(S1) Wu, H.; Chen, W.; Deng, W.; Yang, L.; Li, X.; Hu, Y.; Li, Y.; Chen, L.; Huang, Y. Cathodic Regioselective Coupling of Unactivated Aliphatic Ketones with Alkenes. *Org Lett* **2022**, *24* (6), 1412–1417. https://doi.org/10.1021/acs.orglett.2c00314.

(S2) Cooper, P.; Crisenza, G. E. M.; Feron, L. J.; Bower, J. F. Iridium-Catalyzed A-Selective Arylation of Styrenes by Dual C−H Functionalization. *Angewandte Chemie International Edition* **2018**, *57* (43), 14198–14202. https://doi.org/10.1002/anie.201808299.

(S3) Hostier, T.; Neouchy, Z.; Ferey, V.; Gomez Pardo, D.; Cossy, J. Nickel-Catalyzed System for the Cross-Coupling of Alkenyl Methyl Ethers with Grignard Reagents under Mild Conditions. *Org Lett* **2018**, *20* (7), 1815–1818. https://doi.org/10.1021/acs.orglett.8b00313.

(S4) Arai, Y.; Tomita, R.; Ando, G.; Koike, T.; Akita, M. Oxydifluoromethylation of Alkenes by Photoredox Catalysis: Simple Synthesis of CF_2H-Containing Alcohols. *Chemistry – A European Journal* **2016**, *22* (4), 1262–1265. https://doi.org/10.1002/chem.201504838.

(S5) Li, X.; Gu, Q.; Dong, X.; Meng, X.; Liu, X. A Copper Catalyst with a Cinchona-Alkaloid-Based Sulfonamide Ligand for Asymmetric Radical Oxytrifluoromethylation of Alkenyl Oximes. *Angewandte Chemie International Edition* **2018**, *57* (26), 7668–7672. https://doi.org/10.1002/anie.201804315.

(S6) Bahou, K. A.; Braddock, D. C.; Meyer, A. G.; Savage, G. P.; Shi, Z.; He, T. A Relay Strategy Actuates Pre-Existing Trisubstituted Olefins in Monoterpenoids for Cross-Metathesis with Trisubstituted Alkenes. *J Org Chem* **2020**, *85* (7), 4906–4917. https://doi.org/10.1021/acs.joc.0c00067.

(S7) Bhawal, B. N.; Reisenbauer, J. C.; Ehinger, C.; Morandi, B. Overcoming Selectivity Issues in Reversible Catalysis: A Transfer Hydrocyanation Exhibiting High Kinetic Control. *J Am Chem Soc* **2020**, *142* (25), 10914–10920. https://doi.org/10.1021/jacs.0c03184.

(S8) Wang, G.-Z.; Shang, R.; Cheng, W.-M.; Fu, Y. Irradiation-Induced Heck Reaction of Unactivated Alkyl Halides at Room Temperature. *J Am Chem Soc* **2017**, *139* (50), 18307–18312. https://doi.org/10.1021/jacs.7b10009.

(S9) Zhang, Y.; Li, S.; Chen, H.; Song, W.; Ning, Y.; Liu, Z.; Wu, Y.; Murali, K.; Sivaguru, P.; Zhang, X. Dimethoxyacetaldehyde- *N* -Triftosylhydrazone: Preparation and Carbene Reactivity in Cyclopropanation and Doyle–Kirmse Reactions. *Org Lett* **2025**, *27* (8), 1941–1948. https://doi.org/10.1021/acs.orglett.5c00223.

(S10) Templ, J.; Schnürch, M. High-Energy Ball Milling Enables an Ultra-Fast Wittig Olefination Under Ambient and Solvent-free Conditions. *Angewandte Chemie International Edition* **2024**, *63* (49). https://doi.org/10.1002/anie.202411536.

(S11) Gloria, S. M.; Winfrey, L.; Gao, Y.; Barysevich, M.; Gillions, J. P.; Yun, L.; Patel, H.; Shah, A.; Alvarez-Montoya, A.; Thomas, D.; Khan, A.; Harb, H. Y.; Stuart, A. M.; Pulis, A. P. $B(C_6F_5)_3$-Catalyzed Coupling of *N* -Alkyl

Arylamines and Alkenes for the Synthesis of Tetrahydroquinolines. *Org Lett* **2025**, *27* (36), 9993–9999. https://doi.org/10.1021/acs.orglett.5c03023.
(S12) Mu, X.; Yu, H.; Peng, H.; Xiong, W.; Wu, T.; Tang, W. Construction of Various Bridged Polycyclic Skeletons by Palladium-Catalyzed Dearomatization. *Angewandte Chemie International Edition* **2020**, *59* (21), 8143–8147. https://doi.org/10.1002/anie.202000953.
(S13) Mori, H.; Nakano, S.; Endo, T. Controlled Synthesis of Poly(*N* -Ethyl-3-Vinylcarbazole) and Block Copolymers via RAFT Polymerization. *Macromolecules* **2005**, *38* (20), 8192–8201. https://doi.org/10.1021/ma050918g.
(S14) Fu, M.; Chen, L.; Jiang, Y.; Jiang, Z.-X.; Yang, Z. Copper-Catalyzed Intermolecular Chloro- and Bromotrifluoromethylation of Alkenes. *Org Lett* **2016**, *18* (3), 348–351. https://doi.org/10.1021/acs.orglett.5b03080.
(S15) Falk, A.; Göderz, A.; Schmalz, H. Enantioselective Nickel-Catalyzed Hydrocyanation of Vinylarenes Using Chiral Phosphine–Phosphite Ligands and TMS-CN as a Source of HCN. *Angewandte Chemie International Edition* **2013**, *52* (5), 1576–1580. https://doi.org/10.1002/anie.201208082.
(S16) Sato, K.; Inoue, Y.; Mori, T.; Sakaue, A.; Tarui, A.; Omote, M.; Kumadaki, I.; Ando, A. Csp ^{3} –Csp ^{3} Homocoupling Reaction of Benzyl Halides Catalyzed by Rhodium. *Org Lett* **2014**, *16* (14), 3756–3759. https://doi.org/10.1021/ol501619w.
(S17) Lazib, Y.; Retailleau, P.; Saget, T.; Darses, B.; Dauban, P. Asymmetric Synthesis of Enantiopure Pyrrolidines by C(Sp ^{3})−H Amination of Hydrocarbons. *Angewandte Chemie International Edition* **2021**, *60* (40), 21708–21712. https://doi.org/10.1002/anie.202107898.
(S18) Sandeep; Venugopalan, P.; Kumar, A. Metal Free, Direct and Selective Deoxygenation of Α-Hydroxy Carbonyl Compounds: Access to α,Α-Diaryl Carbonyl Compounds. *European J Org Chem* **2020**, *2020* (17), 2530–2536. https://doi.org/10.1002/ejoc.202000142.
(S19) Harada, N.; Nishikata, T.; Nagashima, H. Vinyl Polymerization versus [1,3] O to C Rearrangement in the Ruthenium-Catalyzed Reactions of Vinyl Ethers with Hydrosilanes. *Tetrahedron* **2012**, *68* (15), 3243–3252. https://doi.org/10.1016/j.tet.2012.02.025.
(S20) Xu, H.; Zhao, C.; Qian, Q.; Deng, W.; Gong, H. Nickel-Catalyzed Cross-Coupling of Unactivated Alkyl Halides Using Bis(Pinacolato)Diboron as Reductant. *Chem Sci* **2013**, *4* (10), 4022. https://doi.org/10.1039/c3sc51098k.
(S21) Tran, H.; McCallum, T.; Morin, M.; Barriault, L. Homocoupling of Iodoarenes and Bromoalkanes Using Photoredox Gold Catalysis: A Light Enabled Au(III) Reductive Elimination. *Org Lett* **2016**, *18* (17), 4308–4311. https://doi.org/10.1021/acs.orglett.6b02021.
(S22) Lv, J.; Chen, S.; Xu, Z.; Zhang, S.; Li, Y.; Zhao, Y. Synthesis and Characterization of Diastereoisomeric Polyesters Derived from Bisphenols Bearing Vicinal Trifluoromethyl Groups. *Macromolecules* **2021**, *54* (8), 3716–3724. https://doi.org/10.1021/acs.macromol.0c02762.
(S23) Sangwan, P. L.; Koul, J. L.; Koul, S.; Reddy, M. V.; Thota, N.; Khan, I. A.; Kumar, A.; Kalia, N. P.; Qazi, G. N. Piperine Analogs as Potent

Staphylococcus Aureus NorA Efflux Pump Inhibitors. *Bioorg Med Chem* **2008**, *16* (22), 9847–9857. https://doi.org/10.1016/j.bmc.2008.09.042.

(S24) Kong, C.; Jana, N.; Jones, C.; Driver, T. G. Control of the Chemoselectivity of Metal *N* -Aryl Nitrene Reactivity: C–H Bond Amination versus Electrocyclization. *J Am Chem Soc* **2016**, *138* (40), 13271–13280. https://doi.org/10.1021/jacs.6b07026.

(S25) Specht, A.; Thomann, J.; Alarcon, K.; Wittayanan, W.; Ogden, D.; Furuta, T.; Kurakawa, Y.; Goeldner, M. New Photoremovable Protecting Groups for Carboxylic Acids with High Photolytic Efficiencies at Near-UV Irradiation. Application to the Photocontrolled Release of <scp>L</Scp> -Glutamate. *ChemBioChem* **2006**, *7* (11), 1690–1695. https://doi.org/10.1002/cbic.200600111.

(S26) Cruz, F. A.; Dong, V. M. Stereodivergent Coupling of Aldehydes and Alkynes via Synergistic Catalysis Using Rh and Jacobsen's Amine. *J Am Chem Soc* **2017**, *139* (3), 1029–1032. https://doi.org/10.1021/jacs.6b10680.

(S27) Wu, Z.; Sun, X.; Potter, K.; Cao, Y.; Zakharov, L. N.; Blakemore, P. R. Stereospecific Synthesis of Alkenes by Eliminative Cross-Coupling of Enantioenriched Sp^{3} -Hybridized Carbenoids. *Angewandte Chemie International Edition* **2016**, *55* (40), 12285–12289. https://doi.org/10.1002/anie.201606641.

(S28) Schade, P.; Schäfer, T.; Müllen, K.; Bender, D.; Knoll, K.; Bronstert, K. Reductive Transformations, 19 [(2)] Electron-Transfer-Induced Dimerization of Β-Alkylstyrenes and the Structures of the Resulting 1,4-Dilithiobutanes [(1)]. *Chem Ber* **1991**, *124* (12), 2833–2841. https://doi.org/10.1002/cber.19911241227.

(S29) Lin, Y.-S.; Li, G.-D.; Mao, S.-P.; Chai, J.-D. Long-Range Corrected Hybrid Density Functionals with Improved Dispersion Corrections. *J Chem Theory Comput* **2013**, *9* (1), 263–272. https://doi.org/10.1021/ct300715s.

(S30) Dunning, T. H. Gaussian Basis Sets for Use in Correlated Molecular Calculations. I. The Atoms Boron through Neon and Hydrogen. *J Chem Phys* **1989**, *90* (2), 1007–1023. https://doi.org/10.1063/1.456153.

(S31) Kendall, R. A.; Dunning, T. H.; Harrison, R. J. Electron Affinities of the First-Row Atoms Revisited. Systematic Basis Sets and Wave Functions. *J Chem Phys* **1992**, *96* (9), 6796–6806. https://doi.org/10.1063/1.462569.

(S32) Peterson, K. A.; Woon, D. E.; Dunning, T. H. Benchmark Calculations with Correlated Molecular Wave Functions. IV. The Classical Barrier Height of the H+H2→H2+H Reaction. *J Chem Phys* **1994**, *100* (10), 7410–7415. https://doi.org/10.1063/1.466884.

(S33) Woon, D. E.; Dunning, T. H. Gaussian Basis Sets for Use in Correlated Molecular Calculations. III. The Atoms Aluminum through Argon. *J Chem Phys* **1993**, *98* (2), 1358–1371. https://doi.org/10.1063/1.464303.

(S34) Neese, F. Software Update: The ORCA Program System—Version 6.0. *WIREs Computational Molecular Science* **2025**, *15* (2). https://doi.org/10.1002/wcms.70019.

(S35) Halkier, A.; Helgaker, T.; Jørgensen, P.; Klopper, W.; Koch, H.; Olsen, J.; Wilson, A. K. Basis-Set Convergence in Correlated Calculations on Ne, N2, and H2O. *Chem Phys Lett* **1998**, *286* (3–4), 243–252.
(S36) Abraham, M. J.; Murtola, T.; Schulz, R.; Páll, S.; Smith, J. C.; Hess, B.; Lindahl, E. GROMACS: High Performance Molecular Simulations through Multi-Level Parallelism from Laptops to Supercomputers. *SoftwareX* **2015**, *1–2*, 19–25. https://doi.org/10.1016/j.softx.2015.06.001.
(S37) Abraham, M.; Alekseenko, A.; Andrews, B.; Basov, V.; Bauer, P.; Bird, H.; Briand, E.; Brown, A.; Doijade, M.; Fiorin, G.; Fleischmann, S.; Gorelov, S.; Gouaillardet, G.; Gray, A.; Irrgang, M. E.; Jalalypour, F.; Johansson, P.; Kutzner, C.; Łazarski, G.; Lemkul, J. A.; Lundborg, M.; Merz, P.; Miletić, V.; Morozov, D.; Müllender, L.; Nabet, J.; Páll, S.; Pasquadibisceglie, A.; Pellegrino, M.; Piasentin, N.; Rapetti, D.; Sadiq, M. U.; Santuz, H.; Schulz, R.; Shirts, M.; Shugaeva, T.; Shvetsov, A.; Turner, P.; Villa, A.; Wingbermühle, S.; Hess, B.; Lindahl, E. GROMACS 2025.3 Source Code. Zenodo August 2025. https://doi.org/10.5281/zenodo.16992621.

(S38) Smith, D. G. A.; Burns, L. A.; Simmonett, A. C.; Parrish, R. M.; Schieber, M. C.; Galvelis, R.; Kraus, P.; Kruse, H.; Di Remigio, R.; Alenaizan, A.; James, A. M.; Lehtola, S.; Misiewicz, J. P.; Scheurer, M.; Shaw, R. A.; Schriber, J. B.; Xie, Y.; Glick, Z. L.; Sirianni, D. A.; O'Brien, J. S.; Waldrop, J. M.; Kumar, A.; Hohenstein, E. G.; Pritchard, B. P.; Brooks, B. R.; Schaefer, H. F.; Sokolov, A. Yu.; Patkowski, K.; DePrince, A. E.; Bozkaya, U.; King, R. A.; Evangelista, F. A.; Turney, J. M.; Crawford, T. D.; Sherrill, C. D. Psi4 1.4: Open-Source Software for High-Throughput Quantum Chemistry. *J Chem Phys* **2020**, *152* (18). https://doi.org/10.1063/5.0006002.
(S39) He, X.; Man, V. H.; Yang, W.; Lee, T.-S.; Wang, J. A Fast and High-Quality Charge Model for the next Generation General AMBER Force Field. *J Chem Phys* **2020**, *153* (11). https://doi.org/10.1063/5.0019056.
(S40) Heinz, H.; Vaia, R. A.; Farmer, B. L.; Naik, R. R. Accurate Simulation of Surfaces and Interfaces of Face-Centered Cubic Metals Using 12−6 and 9−6 Lennard-Jones Potentials. *The Journal of Physical Chemistry C* **2008**, *112* (44), 17281–17290. https://doi.org/10.1021/jp801931d.
(S41) Li, P.; Song, L. F.; Merz, K. M. Systematic Parameterization of Monovalent Ions Employing the Nonbonded Model. *J Chem Theory Comput* **2015**, *11* (4), 1645–1657. https://doi.org/10.1021/ct500918t.
(S42) Jo, S.; Kim, T.; Iyer, V. G.; Im, W. CHARMM-GUI: A Web-based Graphical User Interface for CHARMM. *J Comput Chem* **2008**, *29* (11), 1859–1865. https://doi.org/10.1002/jcc.20945.

(S43) Hodgkins, J. E.; Megarity, E. D. A Study of the Benzyl Free Radical and Substituted Benzyl Free Radicals. *J. Am. Chem. Soc.* **1965**, 87 (23), 5322–5326.

(S44) Cozens, F. L.; Ortiz, W.; Schepp, N. P. Direct Observation of the Benzyl Radical and the Benzyl Anion within Cation-Exchanged Zeolites. A Nanosecond Laser Study. *J. Am. Chem. Soc.* **1998**, 120 (51), 13543–13544.

(S45) Jockusch, S.; Hirano, T.; Liu, Z.; Turro, N. J. A Spectroscopic Study of Diphenylmethyl Radicals and Diphenylmethyl Carbocations Stabilized by Zeolites. *J. Phys. Chem. B* **2000**, 104 (6), 1212–1216.

(S46) Tseng, K. L.; Michl, J. MCD Spectra of Diphenylmethyl Cation and Anion. A Test of the Pairing Theorem. *J. Am. Chem. Soc.* **1976**, 98 (20), 6138–6141.
